# Transcriptional Profiling of Chondrodysplasia Growth Plate Cartilage Reveals Adaptive ER-Stress Networks That Allow Survival but Disrupt Hypertrophy

**DOI:** 10.1371/journal.pone.0024600

**Published:** 2011-09-15

**Authors:** Trevor L. Cameron, Katrina M. Bell, Liliana Tatarczuch, Eleanor J. Mackie, M. Helen Rajpar, Ben T. McDermott, Raymond P. Boot-Handford, John F. Bateman

**Affiliations:** 1 Murdoch Childrens Research Institute, Parkville, Victoria, Australia; 2 School of Veterinary Science, University of Melbourne, Parkville, Victoria, Australia; 3 Wellcome Trust Centre for Cell-Matrix Research, Faculty of Life Sciences, University of Manchester, Manchester, United Kingdom; 4 Department of Biochemistry and Molecular Biology, University of Melbourne, Parkville, Victoria, Australia; University of Western Ontario, Canada

## Abstract

Metaphyseal chondrodysplasia, Schmid type (MCDS) is characterized by mild short stature and growth plate hypertrophic zone expansion, and caused by collagen X mutations. We recently demonstrated the central importance of ER stress in the pathology of MCDS by recapitulating the disease phenotype by expressing misfolding forms of collagen X (Schmid) or thyroglobulin (Cog) in the hypertrophic zone. Here we characterize the Schmid and Cog ER stress signaling networks by transcriptional profiling of microdissected mutant and wildtype hypertrophic zones. Both models displayed similar unfolded protein responses (UPRs), involving activation of canonical ER stress sensors and upregulation of their downstream targets, including molecular chaperones, foldases, and ER-associated degradation machinery. Also upregulated were the emerging UPR regulators *Wfs1* and *Syvn1*, recently identified UPR components including *Armet* and *Creld2*, and genes not previously implicated in ER stress such as *Steap1* and *Fgf21*. Despite upregulation of the *Chop*/*Cebpb* pathway, apoptosis was not increased in mutant hypertrophic zones. Ultrastructural analysis of mutant growth plates revealed ER stress and disrupted chondrocyte maturation throughout mutant hypertrophic zones. This disruption was defined by profiling the expression of wildtype growth plate zone gene signatures in the mutant hypertrophic zones. Hypertrophic zone gene upregulation and proliferative zone gene downregulation were both inhibited in Schmid hypertrophic zones, resulting in the persistence of a proliferative chondrocyte-like expression profile in ER-stressed Schmid chondrocytes. Our findings provide a transcriptional map of two chondrocyte UPR gene networks *in vivo*, and define the consequences of UPR activation for the adaptation, differentiation, and survival of chondrocytes experiencing ER stress during hypertrophy. Thus they provide important insights into ER stress signaling and its impact on cartilage pathophysiology.

## Introduction

Many inherited connective tissue diseases are caused by mutations in genes encoding structural components of the extracellular matrix (ECM), or enzymes that regulate their post-translational modification and assembly [1]. Often the mutations introduce premature termination codons (PTCs), leading to nonsense-mediated decay and haploinsufficiency in the ECM, or interfere with ECM protein folding and assembly, inhibiting their secretion from the cell and disrupting their activity in the ECM when they are secreted, in a dominant-negative manner [1]. Therefore the prevailing paradigm for inherited diseases of the ECM has involved a predominantly extracellular molecular pathology.

More recently it has become clear that intracellular consequences may influence the pathology of these conditions as well. Studies investigating effects of disease-causing missense mutations on assembly and secretion of several ECM components have shown that a common consequence of misfolding and intracellular accumulation of mutant ECM proteins is induction of endoplasmic reticulum (ER) stress [1], [2], [3]. While the role of ER stress and the resulting unfolded protein response (UPR) is well known in the pathology of diseases involving professional secretory tissues, such as pancreas or liver [4], [5], [6], [7], the relative contribution of the UPR versus dominant extracellular effects to the pathology of ECM protein disorders has been a matter of recent debate. In the case of collagen X misfolding mutations, which cause metaphyseal chondrodysplasia, Schmid type (MCDS), this has been resolved by our recent study characterising two mouse models of the human growth plate disease [8]. The first model (*Col10a1* p.Asn617Lys, or Schmid) was generated by knocking in a disease-causing p.N617K mutation in the trimerization-controlling NC1 domain of endogenous collagen X. The second model (*ColXTg^cog^*, or Cog) carried a *Col10a1* promoter-driven transgene encoding a misfolding and ER stress-inducing, Tg^cog^ form of thyroglobulin. Both models displayed essential phenotypic hallmarks of MCDS, which include mild short stature and hypertrophic zone elongation [9], and exhibited ER stress due to constitutive expression of misfolding proteins during chondrocyte hypertrophy. These data demonstrated that ER stress targeted to the hypertrophic zone was sufficient to induce the MCDS phenotype, irrespective of the misfolded protein, and thus highlighted the central importance of the UPR in the pathology of this disease [8].

Classically, the UPR has been understood to alleviate ER stress by enhancing the protein-folding capacity of the ER by upregulating molecular chaperones and foldases, by increasing the cells ability to dispose of irreparably misfolded proteins by upregulating catabolic mechanisms such as the proteasome-mediated ER-associated degradation (ERAD) pathway, and by reducing the ER protein load through translational attenuation [10], [11], [12]. The UPR is initiated when the ER-resident chaperone immunoglobulin-heavy-chain-binding protein (BiP) is sequestered by misfolded proteins from the ER-luminal domains of transmembrane ER stress sensors, including activating transcription factor 6 (Atf6), inositol requiring enzyme 1 (Ire1), and double stranded RNA-activated protein kinase-like ER kinase (Perk), rendering the sensors active. Activated Atf6 is cleaved by proteolysis in the Golgi complex yielding a 50 kDa fragment which drives the transcription of ER stress-responsive genes, including X-box binding protein 1 (*Xbp1*) [13], [14]. Activated Ire1 has both kinase and endoribonuclease activities, catalysing the unconventional cytoplasmic cleavage and splicing of *Xbp1* mRNA, converting it into a potent transcription factor (Xbp1_s_) which regulates the expression of a host of ER-resident molecular chaperones [15], [16], [17]. Ire1 also degrades multiple transcripts encoding components of the secretory pathway, providing rapid alleviation of the ER protein load, and allowing reconfiguration of the secretory pathway molecular machinery to enable an optimal response to ER stress conditions [18], [19]. Activated Perk undergoes dimerization and trans-autophosphorylation, and is then able to phosphorylate the eukaryotic translation initiation factor 2-alpha (Eif2α), preventing formation of the translational initiation complex [20]. In the event of prolonged, unresolved ER stress, the UPR may initiate apoptosis [12], [21], [22], [23]. It has also been suggested recently that cells may alleviate ER stress by cellular reprogramming, or dedifferentiation. Specifically, it was reported that hypertrophic chondrocytes of a transgenic mouse model of MCDS responded to ER stress induced by expression of misfolding collagen X by deploying a “reprogram, recover, and survive” adaptive mechanism, in which collagen X expression is reduced at both the RNA and protein levels by dedifferentiating hypertrophic chondrocytes to a prehypertrophic chondrocyte-like state [24].

Here, we took a holistic approach to resolve how hypertrophic chondrocytes in the Schmid and Cog mice respond to ER stress. Our analyses revealed surprisingly similar UPRs in the Schmid and Cog mice involving upregulation of highly specific subsets of molecular chaperones and foldases, upregulation of ERAD genes, and downregulation of genes encoding secreted proteins. Despite the severity and duration of ER stress, and upregulation of Chop/Cebpb signalling, a widely recognized marker of ER stress-induced apoptosis [12], [21], [22], [23], apoptosis was not elevated in the Schmid hypertrophic zone. Electron microscopic analysis of Schmid and wildtype growth plate hypertrophic zones revealed chondrocytes in the mutants characterized by both engorged ER, indicative of mutant protein retention, and several ultrastructural features more consistent with proliferative chondrocytes, suggesting that mutant chondrocytes undergo developmental arrest as a result of misfolded protein-induced ER stress, failing to become fully hypertrophic. Additional transcriptional profiling analyses measuring the expression of wildtype growth plate zone gene signatures in mutant and wildtype hypertrophic zones were then performed to establish the differentiation status of ER-stressed chondrocytes in the hypertrophic zones of the mutant mice, by determining the extent to which they express wildtype proliferative and hypertrophic zone genes. This unbiased approach confirmed that Schmid and Cog growth plate chondrocytes undergo developmental arrest characterized by impaired expression of many hypertrophic zone genes and retained expression of many proliferative zone genes.

This work provides important insights into ER stress signaling and its impact on cartilage pathophysiology. Specifically, our findings reveal the highly complex changes in gene expression which take place when the UPR is initiated in response to different misfolding proteins in hypertrophic cartilage, and what effects this has on the developmental programming of hypertrophic chondrocytes. Moreover, they provide for the first time global transcriptional maps of two mammalian disease-model UPRs *in vivo*, characterized in each case by the activation of all three ER stress sensors, and the upregulation of highly comparable and specific suites of molecular chaperones and foldases, as well as ERAD machinery and the *Chop*/*Cebpb* pathway, without resultant apoptosis. That many genes were co-regulated in the Schmid and Cog mice identifies them as core components of the chondrocyte UPR.

## Results

### Canonical ER stress sensors are activated in Schmid and Cog hypertrophic zones

**Figure 1:**
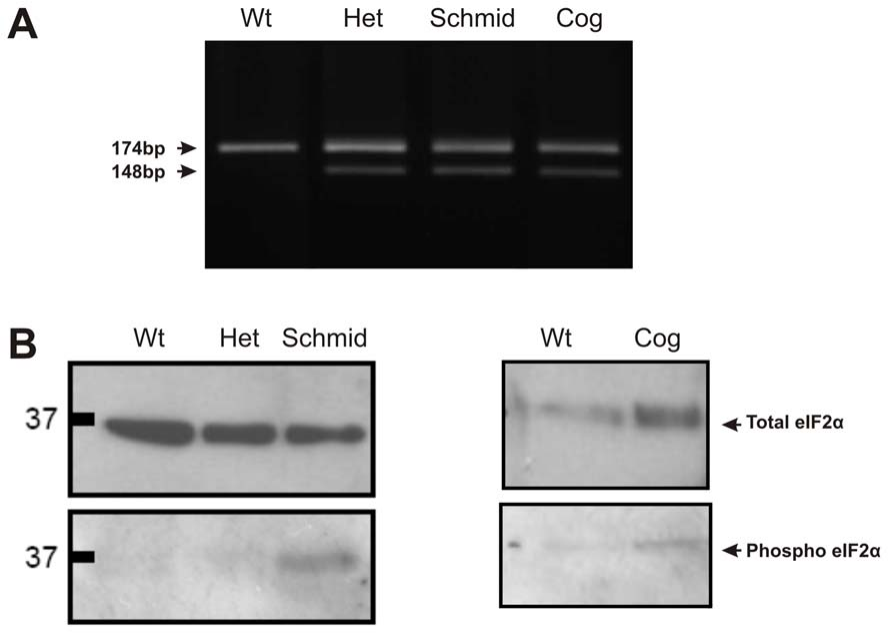
*Xbp1* splicing and Eif2α phosphorylation in Schmid and Cog mouse growth plate hypertrophic zones. (A) RT-PCR performed on aRNA derived from 14 day old wildtype (Wt), heterozygous Schmid (Het), homozygous Schmid (Schmid), and Tg^cog^ (Cog) mouse tibial growth plate hypertrophic zones, using primers specific for sequences flanking the mouse *Xbp1* ER stress-responsive splice site [17]. The 174 bp RT-PCR product represents the unspliced form of *Xbp1*. The 148 bp RT-PCR product represents the spliced form of *Xbp1*. (B) Western blots from SDS-PAGE gels run under reducing conditions with 20 micrograms of whole rib cartilage extracts from Wt, Het, Schmid, and Cog mice, and probed with Eif2α and phospho-Eif2α antibodies.

Previously we showed that Atf6 is proteolytically cleaved in Schmid and Cog cartilages [8], indicating its involvement in the UPRs of these mice. Here we set out to determine whether the remaining canonical ER stress sensors, Ire1 and Perk, are also involved. To determine the activity of Ire1 in the Schmid and Cog UPRs, PCR was performed using primers flanking the *Xbp1* ER stress-responsive splice site, on cDNA derived from microdissected wildtype and mutant hypertrophic zones. In wildtype samples, a single 174 bp RT-PCR product representing unspliced *Xbp1* was detected (Figure 1A). In the heterozygous Schmid (Het), homozygous Schmid (Schmid), and Tg^cog^ (Cog) mice, a smaller, 148 bp RT-PCR product representing spliced *Xbp1* was detected, in addition to the 174 bp product seen in the wildtype samples (Figure 1A). These results confirmed that Ire1 was activated in both mutants. To assay Perk activity in the mutant mice, we performed Western blot analysis of whole cartilage extracts from wildtype and mutant mice using an antibody specific for phosphorylated Eif2α. Increased quantities of phosphorylated Eif2α were detected in Schmid compared with wildtype, but not in Cog (Figure 1B). Taken together, our results for Atf6, Ire1, and Perk indicate that each of the canonical ER stress sensors are active in the UPRs of the Schmid mice, and that at least two are active in the Cog mice.

Next, microarray analyses were performed in triplicate on amplified RNA (aRNA) derived from wildtype, Schmid, and Cog hypertrophic zones, comparing each mutant genotype against wildtype. Each microarray dataset was filtered for probes showing differential expression greater than two-fold, and an adjusted *p* value of ≤0.05. For the Schmid versus wildtype analysis, 1927 probes (1645 genes) registered a fold change of ≥2.00, and 1595 probes (1423 genes) recorded a fold change of ≤−2.00 (Table S1). For the Cog versus wildtype analysis, 424 probes (375 genes) recorded a fold change of ≥2.00, while 141 probes (134 genes) registered a fold change of ≤−2.00 (Table S2). Cog mice carrying the transgene on one chromosome (hemizygous) versus those carrying the transgene on two chromosomes (homozygous) were not discriminated for these analyses; thus gene expression data from hemizygous and homozygous Cog mice were most likely included. Experimental noise generated under these circumstances could explain the smaller number of statistically significant upregulated and downregulated genes observed in the Cog versus wildtype analysis, by comparison with the Schmid versus wildtype analysis. Importantly however, such noise could produce false negative results, but is unlikely to produce false positive results. The full Schmid and Cog microarray datasets are available from NCBI's Gene Expression Omnibus [25] and are accessible through GEO series accession number GSE30628 (http://www.ncbi.nlm.nih.gov/geo/query/acc.cgi?acc=GSE30628).

**Figure 2:**
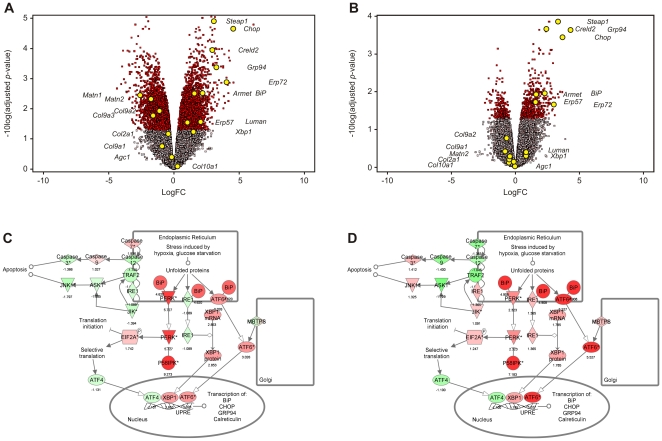
Volcano plots describing differential gene expression in Schmid and Cog mouse growth plate hypertrophic zones. aRNA derived from 14 day old wildtype (Wt), homozygous Schmid (Schmid), and Tg^cog^ (Cog) tibial growth plate hypertrophic zones was labelled with Cy3 and subjected to microarray analysis. (A) Schmid versus Wt and (B) Cog versus Wt analyses expressed as volcano plots showing differential expression plotted along the *x*-axis in LogFC, versus statistical significance plotted along the *y*-axis in −10log(adjusted *p*-value). Genes with adjusted *p* values ≤0.05 are coloured red; genes with adjusted *p* values ≥0.05 are shaded grey. Selected cartilage extracellular matrix and unfolded protein response (UPR) markers are highlighted with yellow spots. (C,D) Schematic diagrams generated using Ingenuity Pathways Analysis (Ingenuity® Systems, www.ingenuity.com), depicting the relationship, and differential expression of key UPR markers in the (C) Schmid versus Wt and (D) Cog versus Wt analyses.

Volcano plots were then generated in which expression data for each microarray probe were plotted according to relative fold change (logFC, mutant versus wildtype) along the x-axis, versus adjusted *p* value (−10log(adjusted *p* value)) along the y-axis (Figure 2). As expected, in the Schmid versus wildtype analysis (Figure 2A) UPR markers including *BiP*, *Grp94*, and *Chop* were upregulated in the mutant (Figure 2A) while genes encoding secreted ECM cartilage markers such as *Col9a2*, *Col9a3*, *Matn1*, and *Matn2* were downregulated. Similar overall patterns were observed in the Cog versus wildtype analysis (Figure 2B), although statistical significance was not reached for a number of these. Microarray analyses were also depicted by uploading the differential expression data into the Endoplasmic Reticulum Stress Canonical Pathway from the Ingenuity Pathways Analysis (Ingenuity Systems®, www.ingenuity.com) library of canonical pathways, further revealing similarity between the UPRs of the Schmid (Figure 2C) and Cog mice (Figure 2D).

### Validation of Schmid and Cog microarray data

**Figure 3:**
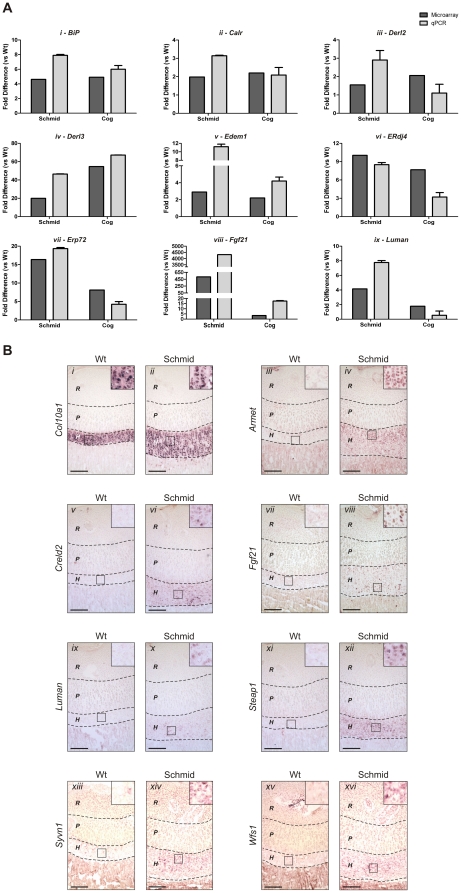
Validation of differential gene expression profiles determined by microarray analysis of aRNA derived from Schmid and Cog mouse growth plate hypertrophic zones. (A) qPCR performed in triplicate on the aRNA samples described in Figure 2, using selected markers of the unfolded protein response (UPR) and endoplasmic reticulum associated degradation pathway, including (*i*) *BiP*, (*ii*) *Calr*, (*iii*) *Derl2*, (*iv*) *Derl3*, (*v*) *Edem1*, (*vi*) *ERdj4*, (*vii*) *Erp72*, *(viii*) *Fgf21*, and (*ix*) *Luman*. Expression profiles are expressed as fold difference for homozygous Schmid (Schmid) or Tg^cog^ (Cog) compared with wildtype (Wt), with profiles determined by the microarray analyses described in Figure 2 shaded dark grey, and profiles determined by qPCR shaded light grey. Error bars indicate standard deviation around the mean. (B) *In situ* analyses performed on 7 day old Wt and Schmid tibial growth plate cryosections using digoxigenin-labelled riboprobes specific for (*i*,*ii*) *Col10a1* as well as novel markers of the UPR including (*iii*,*iv*) *Armet*, (*v*,*vi*) *Creld2*, (*vii*,*viii*) *Fgf21*, (*ix*,*x*) *Luman*, (*xi*,*xii*) *Steap1*, (*xiii*,*xiv*) *Syvn1*, and (*xv*,*xvi*) *Wfs1*. Dashed lines demarcate approximate growth plate zone boundaries: *R* – Resting Zone, *P* – Proliferative Zone, *H* – Hypertrophic Zone. Boxes inset show magnified representative areas of the hypertrophic zones, to highlight the extent of riboprobe hybridization in these zones. Scale bars = 500 µm.

Using cDNA derived from the aRNA interrogated by microarray analysis, we performed qPCR on selected genes as a technical validation of the microarray data (Figure 3A). qPCR was performed on *BiP* (Figure 3A*i*), *Calr* (Figure 3A*ii*), *Derl2* (Figure 3A*iii*), *Derl3* (Figure 3A*iv*), *Edem1* (Figure 3A*v*), *ERdj4* (Figure 3A*vi*), *Erp72* (Figure 3A*vii*), *Fgf21* (Figure 3A*viii*), and *Luman* (Figure 3A*ix*). For each marker, a close correlation was observed between the expression profiles determined by either technique. As further validation, we performed *in situ* analysis on sagittal sections from 7 day old wildtype and Schmid tibiae (Figure 3B). To demarcate hypertrophic zones of wildtype and mutant growth plates, we performed *in situ* analysis for *Col10a1* (Figure 3B*i*,*ii*). Novel gene expression was validated using probes specific for *Armet* (Figure 3B*iii*,*iv*), *Creld2* (Figure 3B*v*,*vi*), *Fgf21* (Figure 3B, *vii*,*viii*), *Steap1* (Figure 3B*xi*,*xii*), *Syvn1* (Figure 3B*xiii*, *xiv*), and *Wfs1* (Figure 3B*xv*, *xvi*), all of which were found by microarray analysis to be highly upregulated in the Schmid and Cog hypertrophic zones compared with wildtype (Tables S1,S2). Additionally, *in situ* analysis was performed using a probe specific for *Luman* (Figure 3B*ix*,*x*), which was upregulated in Schmid only (Figure 3A*ix*). Gene expression detected by ISH confirmed the expression profiles determined by microarray analysis, and resolved the spatial distribution of expression for these genes within the mutant growth plate. *Armet*, *Creld2*, *Fgf21*, *Luman*, *Steap1*, *Syvn1*, and *Wfs1* expression was not observed in the wildtype growth plate, whereas in the Schmid growth plate each gene was highly expressed throughout the hypertrophic zone (Figure 3B*iii*–*xvi*).

### Gene ontology analyses reveal the adaptive nature of the UPR in ER-stressed hypertrophic chondrocytes

Gene ontology (GO) analyses were conducted to identify clusters of functionally related genes being co-ordinately expressed in the hypertrophic chondrocytes of the Schmid and Cog mice. Genes were filtered such that those with a signal intensity of ≥11.0, fold change of ≥2.00 or ≤−2.00 versus wildtype, and adjusted *p* value of ≤0.05 were selected for ontological analysis. The resultant gene lists were interrogated online using DAVID 6.7 (http://david.abcc.ncifcrf.gov/) to generate GO clusters, of which those with enrichment scores of ≥1.3 [26] are shown in Tables S3 and S4. For the Schmid ontological analysis (Table S3), several GO terms identified using genes significantly upregulated in mutant versus wildtype related to protein misfolding, assembly, trafficking, and secretion pathways, while those identified using genes significantly downregulated in mutant versus wildtype related to secreted proteins – such as those involved in skeletal development and extracellular matrix maturation, cytoskeletal organization, and vasculature development. Many of the genes encoding secreted proteins are shown in Table S5, which details the expression profiles of cartilage markers in Schmid, Cog, and wildtype. Interestingly, *Col10a1* was not found to be significantly differentially expressed in either of the mutant mice compared with wildtype (Table S5). *In situ* analysis (Figure 3B*i*,*ii*) on Schmid, Cog, and wildtype, and qPCR performed on cDNA derived from microdissected mutant and wildtype hypertrophic zones (Figure S1) subsequently demonstrated that *Col10a1* was not differentially expressed between mutant and wildtype. The Cog ontological analysis (Table S4) yielded similar results as for Schmid, but with fewer GO terms. Overall nevertheless, the Schmid and Cog ontological analyses both appear to reflect alterations in gene expression designed to restore ER homeostasis in ER-stressed chondrocytes, by enhancing the efficiency of protein folding and trafficking, as well by attenuating transcription of genes encoding secreted proteins in order to reduce the rate at which proteins enter the ER for post-translational modification.

### Similar gene networks are involved in the unfolded protein responses of the Schmid and Cog hypertrophic zones

To explore the gene networks involved in these UPRs in greater detail, we examined the Schmid versus wildtype and Cog versus wildtype microarray datasets with respect to the differential expression of genes involved in protein folding, ER-stress, and its downstream consequences (Table S6). Of the canonical ER stress sensors, *Atf6* was upregulated in both mutants, in addition to being activated (Fig. 1B) [1]. *Perk* was significantly upregulated in Schmid, but we could not detect upregulation in Cog. While *Ire1* was not differentially expressed in the hypertrophic zones of either the Schmid or Cog mice (Table S6), *Xbp1* splicing demonstrated Ire1 activation (Figure 1A). Key downstream targets of each sensor were also upregulated (Table S6). Striking similarity was observed between both mutants for which known targets of the stress sensors were activated. In both models, transcriptional targets of Atf6 including *Armet* [27] and *Grp94* [28], of Ire1 such as *BiP* [29], *ERdj4* [30], and *Syvn1* [31], and of Perk including *Chop*, *Gadd34* [30], and *Wfs1* [32] were all upregulated. In addition to activation of canonical UPR sensors in Schmid and Cog, we also revealed the expression profiles of the other known transmembrane bZIP transcription factors with homology to *Atf6* – *Aibzip*, *Bbf2h7*, *Crebh*, *Luman* (as noted above), and *Oasis*. Only *Bbf2h7* and *Luman* were expressed at appreciable levels in the mutant cartilages; and both were expressed more highly than *Atf6*. *Bbf2h7* however, was not found to be differentially expressed, whereas *Luman* was significantly upregulated in Schmid but not Cog (Table S6).

We observed remarkable similarity between the two strains regarding which molecular chaperones, protein disulphide isomerases, and ERAD components were upregulated. Among the molecular chaperones, a specific subset of genes was upregulated in both models, although many were not found to be differentially expressed. In addition to *BiP*, five more molecular chaperones (*ERdj3*, *ERdj4*, *Grp94*, and *p58IPK*) were upregulated in both Schmid and Cog. Interestingly, a further 9 molecular chaperones (*Dnaja3*, *Dnajb1*, *Dnajc13*, *Hspa2*, *Hspa4*, *Hspb1*, *Hspb8*, *Hspd1*, *and Hsph1*) were highly upregulated in the Schmid mouse but not the Cog mouse, whereas no molecular chaperones were upregulated in the Cog mouse alone (Table S6). Of the protein disulphide isomerases and ERAD components, *Derl3* was most highly upregulated in both models, followed by *Erp72*, *Ero1lb*, *P5*, and *Herpud1*. *Ero1l* was highly upregulated in Schmid, but was not found to be differentially expressed in Cog (Table S6). Having established broad similarity between the Schmid and Cog mutant mice, we focussed primarily on the Schmid mouse for further analysis due to its greater medical relevance.

### Chondrocytes in mutant hypertrophic zones express multiple components of the *Chop* pathway but do not undergo apoptosis

**Figure 4:**
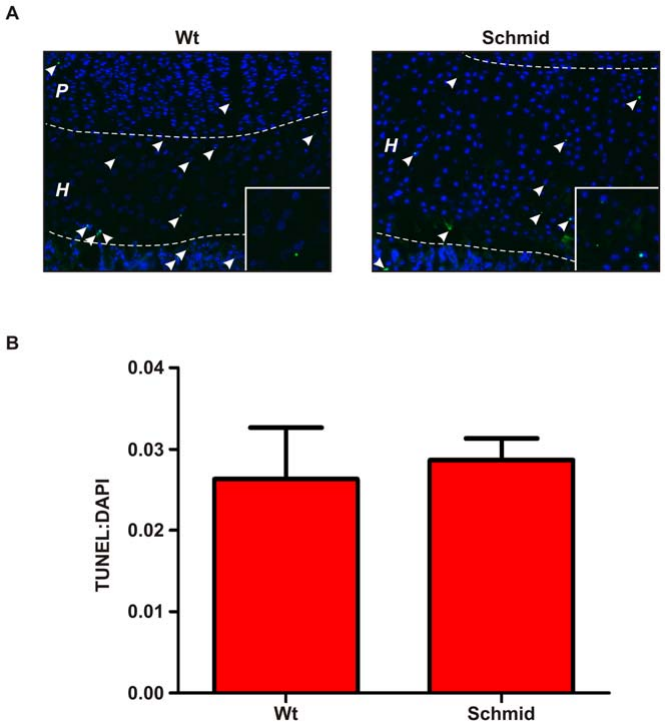
TUNEL analysis of wildtype and Schmid tibial growth plates. (A) Representative 7 day old tibial growth plate cryosections analysed by Terminal deoxynucleotidyl transferase dUTP nick end labelling (TUNEL; green) with 4′,6-diamidino-2-phenylindole (DAPI) counterstaining (blue). TUNEL-positive cells are indicated with white arrowheads. Dashed lines demarcate approximate growth plate zone boundaries: *P* – Proliferative Zone, *H* – Hypertrophic Zone. Boxes inset show magnified representative areas of the hypertrophic zones containing TUNEL-positive chondrocytes. (C) TUNEL analysis of at least 6 tibial growth plate sections from each of 3 Wt and homozygous Schmid mice, expressed as the ratio of TUNEL-positive cells to DAPI-stained nuclei within the hypertrophic zone (TUNEL:DAPI), and showing standard deviation around the mean. Statistical analysis performed using Student's *t*-test, p≤0.05.

Amongst the genes most highly upregulated in both mutants was *Chop*, which has been widely reported to promote ER stress-induced apoptosis [12], [21], [22], [23]. Its dimerization partner *Cebpb* and their transcriptional targets including *Car6* [22] and *Trib3* [33] were also upregulated in Schmid and Cog (Table S6). Therefore, we performed TUNEL analysis on wildtype and Schmid growth plates to determine whether apoptosis is a feature of MCDS ER stress. No statistically significant differences were observed with respect to the relative number or spatial distribution of TUNEL-positive chondrocytes between wildtype and Schmid mice (Figure 4A), with the average rate of TUNEL-positive chondrocytes in wildtype hypertrophic zones being 2.63% compared with 2.90% in the Schmid hypertrophic zones (Figure 4C).

### ER stress in the Schmid and Cog growth plates disrupts the maturation from proliferative chondrocyte to hypertrophic chondrocyte

**Figure 5:**
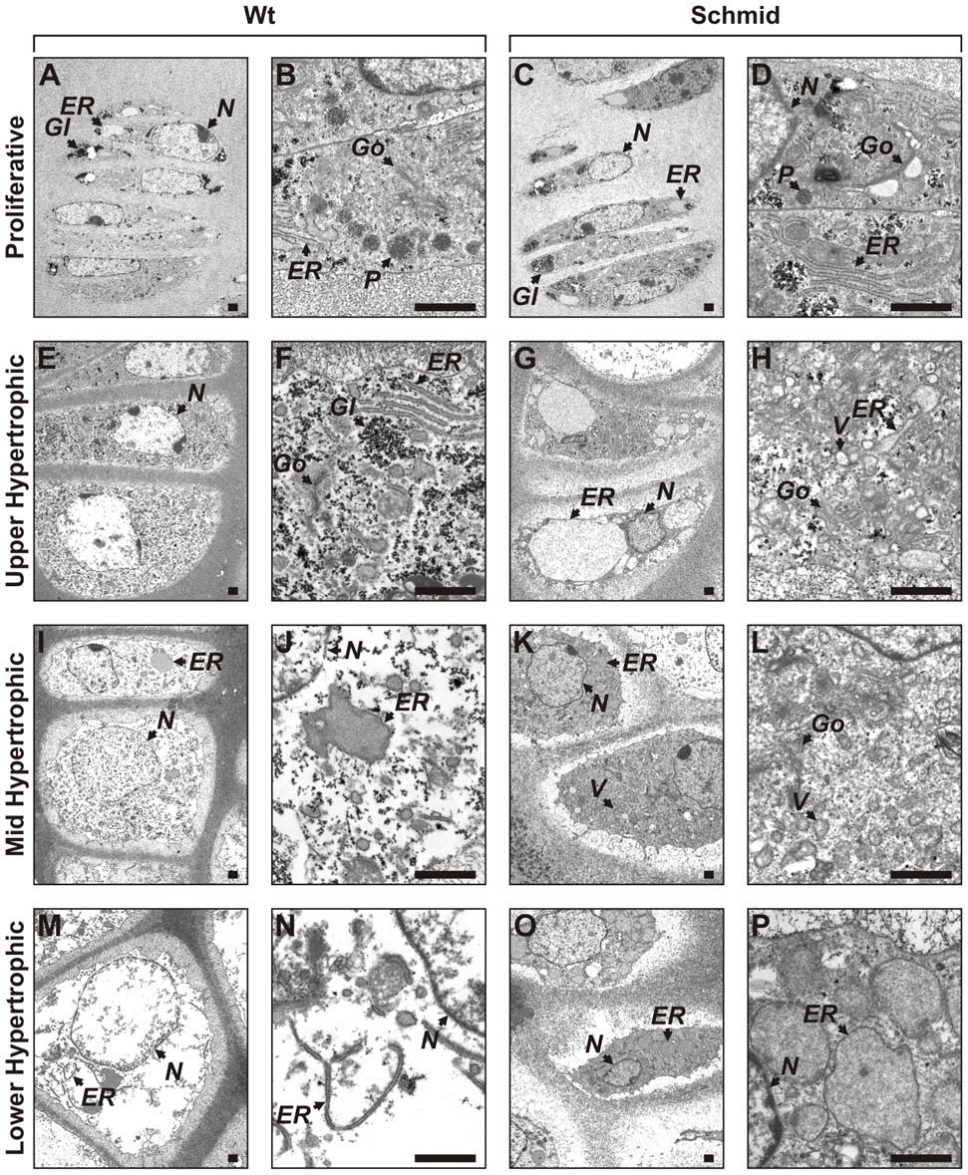
Ultrastructural analysis of wildtype and Schmid tibial growth plates. Transmission electron micrographs of representative chondrocytes from the (A–D) proliferative, (E–H) upper hypertrophic, (I–L) mid-hypertrophic, and (M–P) lower hypertrophic zones of 7 day old wildtype (Wt) and homozygous Schmid (Schmid) tibial growth plates. Italicized letters indicate intracellular features as follows: *ER* – endoplasmic reticulum, *Gl* – glycogen, *Go* – Golgi complex, *N* – nucleus, *P* – proteoglycan, *V* – vesicles. Scale bars = 1 µm.

Our TUNEL analysis revealed that the nuclei of wildtype hypertrophic chondrocytes were refractory to DAPI, whereas the nuclei of chondrocytes in Schmid hypertrophic zones stained as strongly with DAPI as proliferative chondrocytes (Figure 4A,B). Moreover, haematoxylin and eosin staining (Figure S2) displayed a similar pattern as DAPI, as the intensity of haematoxylin staining of chondrocytes throughout the Schmid hypertrophic zones was much greater than that of wildtype hypertrophic chondrocytes, and equivalent to that of proliferative chondrocytes. Subsequent ultrastructural analysis of chondrocytes throughout the wildtype and Schmid growth plates by transmission electron microscopy (TEM) further defined similarities between chondrocytes in the mutant hypertrophic zones and proliferative chondrocytes (Figure 5). As expected, mutant proliferative chondrocytes were indistinguishable from wildtype proliferative chondrocytes (Figure 5A–D), prior to the expression of collagen X and the onset of ER stress in the mutant hypertrophic zone. In the Schmid upper (Figure 5G,H), mid (Figure 5K,L), and lower (Figure 5O,P) hypertrophic zones most chondrocytes were characterized by grossly distended ER, typical of cells expressing misfolded proteins with compromised secretion. Most chondrocytes throughout the Schmid hypertrophic zone were further typified by a relative paucity of glycogen when compared with wildtype, presumably reflecting the significant energetic demands involved in responding to ER stress. Also, chondrocytes in the mutant hypertrophic zone displayed several subcellular hallmarks of proliferative chondrocytes, including having a smaller, flattened appearance, proliferative chondrocyte-like nuclear ultrastructure, and highly developed protein secretory machinery including abundant Golgi complexes and vesicles presumably involved in protein transport and secretion. A smaller proportion of chondrocytes in the Schmid hypertrophic zone displayed a more typical hypertrophic ultrastructure. Therefore most, but not all chondrocytes throughout the hypertrophic zones of the Schmid growth plate displayed signs of severe ER stress and developmental arrest, resembling proliferative chondrocytes on the basis of nuclear staining and TEM ultrastructural analysis.

To characterize the disruption to chondrocyte maturation in the mutant growth plates further, we took a gene expression profiling approach. Thus we microdissected hypertrophic zones and proliferative zones from two week old wildtype mouse tibiae and linearly amplified the isolated RNA. To validate the RNA, we performed qPCR using hypertrophic zone markers *Adamts1*, *Col10a1*, and *Mmp9* [34], [35], and proliferative zone markers *Fmod*, *Gdf10*, and *Prelp* [35], [36], [37] (Figure S3). Each marker was significantly more highly expressed in the expected growth plate zone, validating the samples. Gene expression microarray analyses were then performed on each sample. To establish gene expression signatures which define wildtype hypertrophic and proliferative zones based on these microarray analyses, genes were regarded as defining the hypertrophic zone if they were ≥2-fold more highly expressed in the hypertrophic zone than the proliferative zone with an adjusted *p* value ≤0.05, and defining the proliferative zone if they were ≥2-fold more highly expressed in the proliferative zone than the hypertrophic zone with an adjusted *p* value ≤0.05. A cohort of 510 genes comprised the wildtype hypertrophic zone gene expression signature (Table S7), while a cohort of 773 genes comprised the wildtype proliferative zone gene expression signature (Table S8). Ontological analysis of each cohort using DAVID 6.7 software authenticated the gene expression signatures, yielding GO terms related to programmed cell death, vasculature development, and skeletal system development for the hypertrophic zone signature (Table S9), and cell cycle regulation, DNA metabolism, and chromosomal structure for the proliferative zone signature (Table S10).

**Figure 6:**
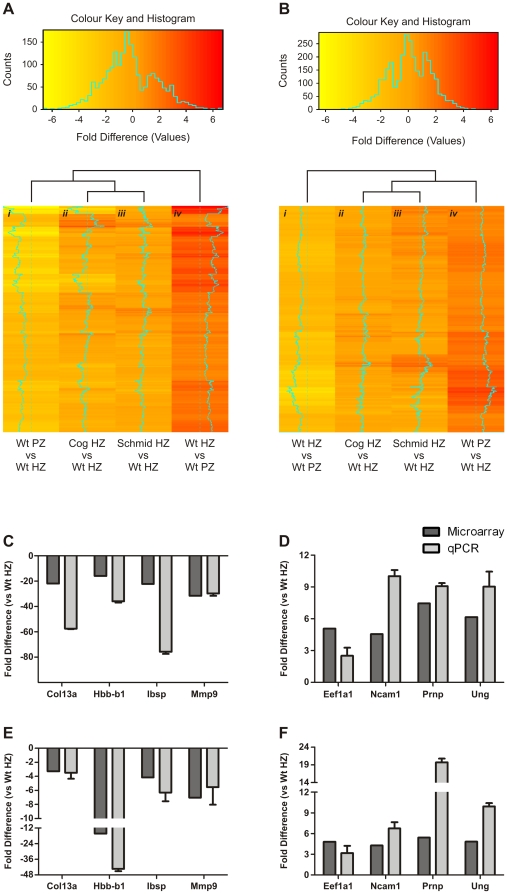
Expression of wildtype hypertrophic and proliferative growth plate zone gene signatures in Schmid and Cog hypertrophic zones. (A) Heatmap depicting the relative fold difference (log fold change) of 510 wildtype (Wt) hypertrophic zone (HZ) signature genes following the comparison of datasets generated by microarray analyses of Wt proliferative zone (PZ), Wt HZ, homozygous Schmid (Schmid) HZ, or Tg^cog^ (Cog) HZ aRNA (N = 3). (B) Heatmap depicting the relative expression (log fold change) of 773 Wt PZ signature genes following the comparison of datasets generated by microarray analyses of Wt PZ, Wt HZ, Schmid HZ, or Cog HZ aRNA (N = 3). For both heatmaps, each Wt growth plate zone signature gene is represented by a single bar, colour-coded according to relative expression as indicated, with downregulated genes coloured yellow, and upregulated genes coloured red. (C–F) Validation of (A) and (B) by quantitative PCR (qPCR) using (C) Wt PZ and Wt HZ cDNA for markers of the Wt HZ gene signature, (D) Wt PZ and Wt HZ cDNA for markers of the Wt PZ gene signature, (E) Schmid HZ and Wt HZ cDNA for the Wt HZ markers used in (C), and (F) Schmid HZ and Wt HZ cDNA for the Wt PZ markers used in (D); N = 3, expression profiles expressed as Fold Difference versus Wt HZ, microarray data shaded dark grey, qPCR data shaded light grey, qPCR error bars indicate standard deviation around the mean.

Next, we used these signatures as references for determining the differentiation status of chondrocytes in the mutant hypertrophic zones. Thus, we performed two analyses. In the first, we analysed our existing Schmid and Cog microarray datasets for the relative expression (log fold change) of the wildtype hypertrophic zone gene signature, to determine the extent to which wildtype hypertrophic zone markers were upregulated in the mutant hypertrophic zones. The results of this analysis are represented by the heatmap in Figure 6A, which indicates that in terms of the expression of the wildtype hypertrophic zone gene signature, chondrocytes in the Schmid and Cog hypertrophic zones resemble each other more closely than those of either wildtype sample, but more closely resemble wildtype proliferative chondrocytes than wildtype hypertrophic chondrocytes. Gene set tests were highly significant for both Schmid versus wildtype and Cog versus wildtype (*p*≤0.001 in each case). Therefore, a significant number of hypertrophic zone signature genes were expressed at abnormally low levels in the Schmid and Cog hypertrophic zones by comparison with the wildtype hypertrophic zone. In the second analysis, we re-examined the same microarray datasets, this time for the relative expression of the wildtype proliferative zone gene signature, to determine the extent to which wildtype proliferative zone markers were downregulated in the mutant hypertrophic zones. The results of this analysis are represented by the heatmap in Figure 6B, which indicates that in terms of the expression of the wildtype proliferative zone gene signature, chondrocytes in the Schmid and Cog hypertrophic zones resemble each other more closely than either wildtype sample. Unlike the first analysis however, the gene set test for Schmid versus wildtype was significant (*p*≤0.001), but the gene set test for Cog versus wildtype was not significant (*p*≥0.1). Therefore, a significant number of proliferative zone signature genes were expressed abnormally highly in chondrocytes in the Schmid hypertrophic zone by comparison with the wildtype hypertrophic zone, however the same genes were not found to be significantly abnormally expressed in the Cog hypertrophic zone.

To validate the microarray analyses, qPCR was performed on wildtype and Schmid growth plate zone cDNA for a cohort of highly differentially expressed genes. *Col13a1*, *Hbb-b1*, *Ibsp*, and *Mmp9* (Figure 6C,E) were found by microarray analysis to be more highly expressed in the wildtype hypertrophic zone compared with the wildtype proliferative zone, and *Eef1a1*, *Ncam1*, *Prnp*, and *Ung* (Figure 6D,F) were more highly expressed in the wildtype proliferative zone compared with the wildtype hypertrophic zone. Consistent with the microarray data, each hypertrophic zone marker gene was found by qPCR to be expressed very lowly in the proliferative zone compared with the wildtype hypertrophic zone (Figure 6C), and in the Schmid hypertrophic zone compared with the wildtype hypertrophic zone (Figure 6E). Conversely, each proliferative zone marker gene was found by qPCR to be expressed very highly in the proliferative zone compared with the wildtype hypertrophic zone (Figure 6D), and in the Schmid hypertrophic zone compared with the wildtype hypertrophic zone (Figure 6F). Thus we confirmed that in the Schmid hypertrophic zone, the wildtype hypertrophic zone gene signature is not fully upregulated, while the proliferative zone gene signature is not fully downregulated.

**Figure 7:**
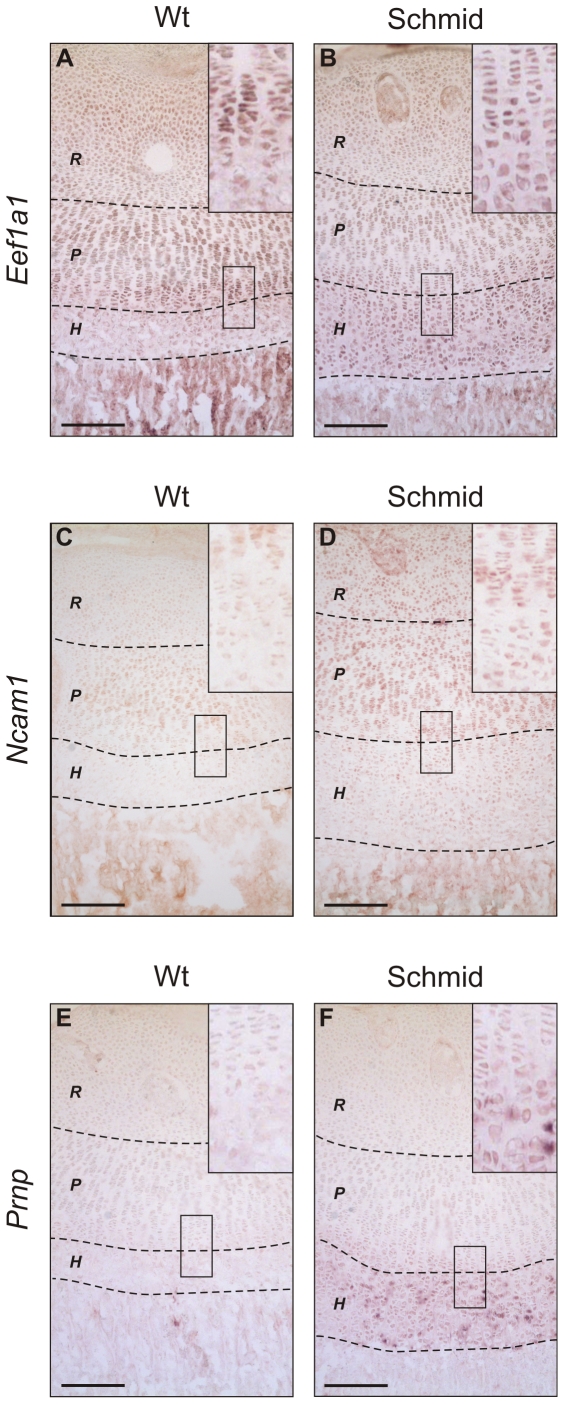
*In situ* analysis of growth plate proliferative zone gene signature markers. *In situ* analyses performed on 7 day old wildtype (Wt) and homozygous Schmid (Schmid) tibial growth plate cryosections using digoxigenin-labelled riboprobes specific for the selected growth plate proliferative zone gene signature markers (A,B) *Eef1a1*, (C,D) *Ncam1*, and (E,F) *Prnp*. Dashed lines demarcate approximate growth plate zone boundaries: *R* – Resting Zone, *P* – Proliferative Zone, *H* – Hypertrophic Zone. Boxes inset show magnified representative areas of the hypertrophic zones, to highlight the extent of riboprobe hybridization in these zones. Scale bars = 500 µm.

Next, the disrupted differentiation of ER-stressed growth plate chondrocytes was further explored by *in situ* analyses, using probes specific for *Eef1a1* (Figure 7A,B), *Ncam1* (Figure 7C,D), and *Prnp* (Figure 7E,F), to show the spatial distribution of proliferative zone marker expression in wildtype and mutant tibial growth plates. In the wildtype growth plate, expression of each gene was observed in the non-hypertrophic zones, and most strongly in the proliferative zone, but excluded from the wildtype hypertrophic zone (Figure 7A,C,E). In Schmid, the expression pattern for each gene observed in the non-hypertrophic zones of the wildtype growth plate was maintained, but unlike wildtype the domain of expression extended in the mutant into the hypertrophic zone, and as far as the ossification front (Figure 7B,D,F). Thus our *in situ* analyses further validated the abnormal growth plate zone marker expression patterns determined by microarray analysis and qPCR in Schmid and Cog, and confirmed that chondrocytes in the mutant growth plates undergo developmental arrest, failing to undergo full hypertrophy, and retaining significant elements of the proliferative chondrocyte phenotype.

## Discussion

Several studies investigating inherited connective tissue disorders have implicated the UPR as a feature of the disease mechanism. Our recent work, in which the MCDS phenotype was recapitulated by expressing different misfolding proteins in the mouse growth plate hypertrophic zone, was the first study to demonstrate unequivocally the central importance of the UPR in the molecular pathology of such diseases [8]. Here we used a gene expression profiling approach to characterize the UPRs of our mutant mice, identify novel components of these regulatory networks in chondrocytes, and define consequences of the UPRs for the differentiation of chondrocytes in the hypertrophic zone.

### The Schmid and Cog UPRs are characterized by activation of the canonical ER stress-signalling pathways

We showed previously that chondrocytes in the hypertrophic zones of Schmid and Cog mice upregulate and proteolytically cleave Atf6 in response to ER stress [8]. Here we show that *Atf6* gene expression was increased in these mutants as well (Table S6). *Ire1* was not found to be differentially expressed between mutant and wildtype, but *Xbp1* splicing in the Schmid and Cog hypertrophic zones confirmed Ire1 activity in the UPR of both mutants (Figure 1A). *Perk* expression and activity was found to be increased in the Schmid hypertrophic zone, but in Cog we were unable to detect significant upregulation or activation of *Perk* (Table S6; Figure 1B). The lack of observed *Perk* mRNA upregulation and lack of apparent Eif2α phosphorylation may indicate that Tg^cog^ thyroglobulin elicits less ER stress in growth plate cartilage than p.N617K collagen X. This is consistent with the finding that the hypertrophic zone expansion, apparent in both mice at younger ages, is resolved in the Cog mice alone by 6 weeks of age [8], suggesting a milder pathological effect. Regardless, each of the canonical ER stress sensors was activated in Schmid, and at least two of these sensors were also activated in Cog mouse hypertrophic cartilage.

This is the first study to implicate all three sensors in the molecular pathology of a skeletal disease. While *Xbp1* splicing was confirmed previously in a transgenic mouse model of MCDS [24], neither Atf6 proteolysis nor Eif2α phosphorylation were demonstrated in that study. In contrast, mild ER stress underpinning a p.T583M *Comp* mutant model of pseudoachondroplasia was found to involve Atf6 cleavage and Eif2α phosphorylation, but not *Xbp1* splicing [38]. It remains unknown whether *Xbp1* splicing, Atf6 proteolysis, or Eif2α phosphorylation occur in other mouse models of skeletal dysplasias characterized by ER stress, including the p.V194D *Matn3* mutant model of multiple epiphyseal dysplasia [39], and an *Aga2*-mutant model of osteogenesis imperfecta [40]. Nevertheless, activation of all three sensors is understood to be the common response of ER-stressed cells, and the combination and kinetics of sensor activation is thought to influence cell fate [41].

Another putative chondrocyte ER-stress sensor, *Luman*, was upregulated in Schmid, although it was not found to be differentially expressed in the Cog hypertrophic zone (Table S6; Figure 3A*ix*, 3B*ix*,*x*). *Luman* encodes an ER membrane-resident [42] type II transmembrane glycoprotein [43], within the cytoplasmic portion of which resides a basic domain leucine zipper transcription factor [44]. As with its structural homologs Atf6 [45], Bbf2h7 [46], and Oasis [47], Luman may be cleaved and thereby activated via S1P proteolysis [43]. Activated Luman targets the ERSE consensus sequence in the promoters of multiple UPR genes, including ERAD markers *Edem* [45] and *Herp* [48]. It is unclear why *Luman* was upregulated in Schmid but no significant differential expression was observed in Cog, or whether proteolytic activation of Luman occurs in Schmid. Therefore, despite its transcriptional upregulation we cannot confirm that *Luman* contributes functionally to the Schmid UPR. Another Atf6 homolog, *Aibzip* [49], was also upregulated specifically in Schmid, although at a much lower level than other Atf6 homologs (Table S6). *Bbf2h7*, which modulates physiological ER stress in chondrocytes by regulating components of the protein secretory pathway during early chondrogenesis [50], and *Oasis* [49], were not differentially expressed in either model. Importantly however, as with Luman, their roles in modulating pathological ER stress in chondrocytes cannot be ruled out until proteolytic cleavage has been tested in suitable mouse models.

### The Schmid and Cog UPRs are surprisingly similar

Consistent with activation of the canonical ER stress sensors, our microarray analyses also revealed upregulation of several of their gene targets (Figure 2, Table S6). In both Schmid and Cog, we saw upregulation of specific and highly comparable subsets of molecular chaperones including *BiP*, *ERdj4*, and *Grp94*, foldases such as *Erp57*, *Erp72*, and *Ero1lb*, and components of the ERAD pathway such as *Derl3* and *Syvn1* (Table S6). Erp57 is a glycoprotein-specific protein disulphide isomerase, and is important in the formation of disulphide bonding intermediates during thyroglobulin biosynthesis [51], [52]. It is known that *Ero1lb* expression may be induced in mammalian cells in response to chemical inducers of ER stress [53]. This study however, is the first to confirm upregulation of *Ero1lb* in response to ER stress in an *in vivo* mammalian disease model. Syvn1 is an E3 ubiquitin ligase which has recently been shown to co-operate with Wfs1 (also significantly upregulated in both Schmid and Cog; Table S6) in suppressing the UPR by enhancing the proteasomal degradation of Atf6 [54]. Thus it is an intriguing possibility that the deleterious effects of chronic ER stress in Schmid and Cog chondrocytes are modulated in part by the Syvn1/Wfs1-mediated degradation of Atf6.

Wildtype collagen X and thyroglobulin contrast significantly in their biosynthesis. Mature collagen X consists of three α1(X) chains, each containing a helical, collagenous domain flanked by N- and C-terminal non-collagenous domains [55], [56]. Collagen X biosynthesis has been extensively reviewed [57], [58]. Briefly, α1(X) monomers become aligned via their NC1 domains within the ER lumen for homotrimer assembly, which occurs through triple helix formation at the alpha-helical collagenous domain. Except for bovine collagen X, post-translational modification and assembly of wildtype collagen X does not involve disulphide bond formation [59]. In contrast, thyroglobulin is a 660 kDa homodimeric glycoprotein, whose intracellular assembly requires extensive post-translational modifications including glycosylation, the formation of 60 disulfide bonds, phosphorylation, proteolysis, and iodination [60], [61]. In view of this, the similarity between the Schmid and Cog UPRs was particularly surprising. This unexpected similarity may be reconciled by the finding that while intracellular assembly of wildtype collagen X does not involve disulphide bond formation, MCDS mutations including p.N617K can cause aberrant disulphide bond formation to occur in mutant collagen X assembly [62]. Therefore, the molecular machinery involved in the post-translational modification of the mutant proteins may differ from those involved in biosynthesis of their wildtype counterparts, making it potentially difficult to predict the UPRs based on knowledge of wildtype biosynthetic pathways. The differences between Schmid and Cog UPRs are an important area of future study and further detailed molecular comparisons may reveal additional complexities in UPR regulation and downstream signalling.

However, the extent of co-regulation observed between the Schmid and Cog UPRs suggests that the UPR is largely not protein-specific, and that most UPR target genes are part of a generic, or default response to ER stress. This possibility raises the hope that new, “generic” treatment strategies may be developed which can ameliorate the deleterious consequences of unresolved chondrocyte-specific UPRs by targeting components of common pathways.

### Expression of novel UPR markers revealed by microarray analysis of Schmid and Cog hypertrophic zones

Our microarray analyses also identified several genes recently implicated in the UPR, including *Armet* and *Creld2*, and other genes never previously associated with ER stress, such as *Steap1* and *Fgf21*. Each of these genes was highly upregulated in Schmid and Cog compared with wildtype (Table S6; Figure 3). *Armet* (arginine-rich, mutated in early stage tumours; also called *Manf* – mesencephalic astrocyte derived neurotrophic factor) is a soluble 18 kDa protein [63] which localizes to the ER but may also be secreted [64]. *Armet* is a robust UPR marker, inducible *in vitro* with chemical ER stressors [63], [64], [65], as well as in the brain following experimentally induced ischemia [64], or by misfolding Matn3 expressed in a mouse model of multiple epiphyseal dysplasia [39]. *Armet* expression is regulated by Xbp1_s_ and Atf6 [65], and mediated by an ERSE-II element in its promoter [63]. The function of Armet is not fully resolved, though it is known to impair cell proliferation and protect against ER stress-induced cell death [64]. Intrastriatal injection of exogenous Armet has proven efficacious in the treatment of an experimental model of Parkinson's disease, a neurodegenerative disorder related to ER stress in, as well as loss of, dopaminergic neurons [66], [67]. Thus, *Armet* appears to have a prominent role in the UPR, and it will be important to determine whether its manipulation can ameliorate not only Parkinson's disease, but other ER stress disorders as well, including MCDS.

*Creld2* (cysteine rich with EGF-like domains 2) encodes an ER-resident [68], [69], 60 kDa glycoprotein [68]. Like *Armet*, *Creld2* is inducible *in vitro* using chemical ER stressors, and is regulated by Atf6 via an ERSE element in its promoter [68]. The function of *Creld2* is unknown, however it was shown to be highly upregulated, along with *Armet*, in a *Matn3* mutant model of multiple epiphyseal dysplasia [39]. Thus, the upregulation of *Creld2* observed in the Schmid and Cog UPRs here represents the second time this gene has been implicated in the UPR of a mouse model of an ER stress-related disease. Upregulation in response to various *in vitro* and *in vivo* stressors strongly implicates *Creld2* as having an important role in the UPR which is neither stimulus- nor cell type-specific, and highlights the need for further research into the function of this conspicuous, novel ER stress marker.

Less is known about *Steap1* (six transmembrane epithelial antigen of the prostate 1), a protein which is widely expressed and which has recently been identified marker of various types of cancer [70]. *Steap1* bears greater than 60% homology to three other family members – *Steap2*, *Steap3*, and *Steap4* [71], none of which were upregulated in either Schmid or Cog (Table S6). In addition to their structural homology, all four Steap proteins have been found to localize to endosomes [71]. This is the first study, to our knowledge, to demonstrate the upregulation of *Steap1* in response to any kind of ER stress. Therefore *Steap1* may constitute a novel, chondrocyte-specific component of the UPR.

Fibroblast growth factor 21 (*Fgf21*) belongs to the endocrine-acting Fgf19 subfamily of the Fgf superfamily [72]. Under normal conditions, *Fgf21* is widely expressed in metabolically important tissues including liver, fat, muscle, and pancreas, and accordingly is involved in multiple metabolic processes including adaptation to starvation [73]. Fgf21 signalling is known to be mediated by either of Fgfr1, -2, or -3 in conjunction with the co-regulator β-klotho [72]. To our knowledge, this is the first study to report the upregulation of *Fgf21* in response to ER stress. Intriguingly, we also found a putative ERSE element in the mouse *Fgf21* promoter positioned 310 bp upstream of the *Fgf21* start codon (Figure S4). This sequence closely resembles the previously reported consensus sequence for ERSE I elements, which are *cis*-acting regulatory motifs that favour the upregulation of UPR target genes and glucose-regulated proteins [74]. It is unclear what role Fgf21 has in the UPR of the Schmid or Cog mice. While it is possible that Fgf21 may play a currently unrecognised direct role in the UPR, it may also be that *Fgf21* is upregulated in ER-stressed Schmid and Cog chondrocytes as an adaptive response to “starvation” caused by the significant energetic cost of UPR activation. Such costs are reflected by the appreciable depletion of glycogen deposits in the ER-stressed chondrocytes of the upper hypertrophic zone compared with wildtype (Figures 5F,H).

### Downregulation of genes encoding secreted proteins in ER-stressed Schmid chondrocytes

Genes encoding secreted proteins were widely downregulated in Schmid (Table S3), including cartilage ECM components such as *Matn1*, *Matn2*, *Col9a2*, *Col9a3*, *Col11a2*, *Chad*, as well as proteases, including *Mmp9* (Table S5). ECM integrity is critical for providing structural support, as well as signalling information during normal tissue development [1]. Thus, while the downregulation of cartilage ECM and protease genes observed in ER-stressed Schmid chondrocytes would significantly reduce their ER protein load, favouring their survival, we anticipate it having deleterious effects on mutant growth plate function as well. It is known for example that Vegf, which mediates vascularisation during endochondral ossification [75], may be stored bioactively bound to the ECM, and released as a soluble angiogenic factor in response to protease activity [76]. Mmp9 and Mmp13 may be important for Vegf release from the hypertrophic zone to mediate growth plate vascularisation, and loss of either protease from the growth plate results in hypertrophic zone elongation [77], [78]. Therefore impaired vascularisation of the mutant growth plates may be caused not only by reduced *Vegf* expression, as we observed previously in the lower half of Schmid hypertrophic zones [8], but also by reduced bioavailability of Vegf and other growth factors, due to disrupted development of mutant hypertrophic cartilage matrices caused by UPR-mediated transcriptional attenuation of ECM and protease genes. That the pathology of MCDS involves impaired growth plate vascularisation is further supported by the much lower expression of *Hbb-b1* [34] (Table S1, Figure 6C,E), along with other genes related ontologically to vasculature development (Table S3), in the mutant hypertrophic zones compared with wildtype.

Interestingly, *Col10a1* was not found to be differentially expressed in our microarray analyses of 2 week old Schmid and Cog tibiae compared to wildtype (Table S6). That *Col10a1* is not differentially expressed between Schmid and wildtype in 1 week old tibial growth plates was then demonstrated by *in situ* analysis (Figure 3B*i*,*ii*). By contrast, we [8] and others [24] previously observed downregulation of mutant *Col10a1* in the hypertrophic zones of 3 week old MCDS mouse growth plates. The growth rate of wildtype and Schmid mice is maximal between 3–4 weeks [8]. Accordingly, it may be that the level of mutant collagen X passing through the ER of Schmid mice is also maximal at 3 weeks, resulting in a more severe ER stress than at 1–2 weeks. Thus, the different *Col10a1* expression patterns observed in MCDS growth plates here and previously may reflect different levels of ER stress severity during the time course of the disease.

### Apoptosis is not a feature of the UPR in Schmid or Cog ER-stressed chondrocytes

Apoptosis was not increased in the Schmid hypertrophic zone compared with wildtype (Figure 4), despite upregulation of *Chop*, *Cebpb*, and key transcriptional targets in mutant hypertrophic zones by comparison with wildtype (Table S6). *Chop* is widely regarded as a marker of ER-stress induced apoptosis. Numerous studies have shown that its over-expression promotes apoptosis, while its inhibition favours cell survival [79], [80], [81]. More recently however, others have demonstrated that Chop can play pro-survival roles in the UPR [82]. As already noted, the fate of ER-stressed cells is thought to depend on the combination and kinetics of the stress sensors they activate. ER-stressed cells activate at least Ire1, Atf6, and Perk, however Ire1 (and Atf6) activity may be transient [41]. Indeed Tsang et al previously demonstrated *Xbp1* splicing only in the upper portion of the hypertrophic zone in their transgenic model of MCDS [24], suggesting transient Ire1 activity in ER-stressed chondrocytes in this disease. The kinetics and duration of Ire1 activity is important, but controversial. Some suggest that its activity favours cell survival, while its loss, coupled with persistent Perk activation, favours apoptosis [41]. Others however, have correlated the *Xbp1*-independent kinase activity of Ire1 with increased apoptosis [19]. These studies, and ours, highlight the complexities involved in trying to understand how the regulation of cell fate during ER stress is modulated.

### ER-stressed Schmid and Cog chondrocytes undergo developmental arrest and retain ultrastructural and molecular features of proliferative chondrocytes

We have revealed significant histological and ultrastructural similarities between chondrocytes in the Schmid hypertrophic zone and proliferative chondrocytes (Figure 5). Moreover, our gene expression profiling analyses revealed that chondrocytes in the Schmid hypertrophic zone do not fully upregulate wildtype hypertrophic zone markers (Figure 6A,C,E), or downregulate proliferative zone markers (Figure 6B,D,F). Crucially, the abnormal nuclear staining (Figure S2; Figure 4A), proliferative chondrocyte-like ultrastructural features (Figure 5), and growth plate zone marker expression (Figure 7) were present in chondrocytes throughout the hypertrophic zones of the mutant mice. These findings differ from those of Tsang et al, who reported loss of proliferative and pre-hypertrophic (late proliferative) zone marker expression in the upper portion of the hypertrophic zone of their MCDS mouse model, followed by resumed expression in the lower hypertrophic zone, and who argued on this basis that MCDS growth plate chondrocytes become hypertrophic before de-differentiating to a pre-hypertrophic chondrocyte-like state, to ameliorate mutant collagen X-induced ER stress [24]. Rather, our data on the Schmid mutant suggest that with the onset of hypertrophy, expression of misfolding collagen X and the subsequent onset of ER stress disrupts further maturation of growth plate chondrocytes due to UPR-mediated dysregulation of normal gene expression. Consequently, ER-stressed mutant chondrocytes in this MCDS model undergo developmental arrest, retaining significant elements of the proliferative chondrocyte gene signature, failing to acquire the full gene signature of wildtype hypertrophic chondrocytes, and continuing to display several key subcellular ultrastructural hallmarks of proliferative chondrocytes. As a result, it is likely that they are unable to provide vital microenvironmental cues required to facilitate growth plate remodelling and vascularisation such as proteases and pro-angiogenic signaling molecules (discussed above), accounting for the hypertrophic zone elongation characteristic of the MCDS growth plate.

## Materials and Methods

### Ethics Statement

*Col10a1* p.Asn617Lys mice (Schmid) and *ColXTg^cog^* mice (Cog), which were generated as described previously [8], as well as wildtype littermates, and Swiss white mice were sacrificed in accordance with Institutional Animal Ethics guidelines at one week or two weeks post-partum, as indicated.

### Western Blotting

Whole cartilage extracts and Western blots were generated as described previously [8].

### Microdissection of mutant and wildtype growth plate zones

**Figure 8:**
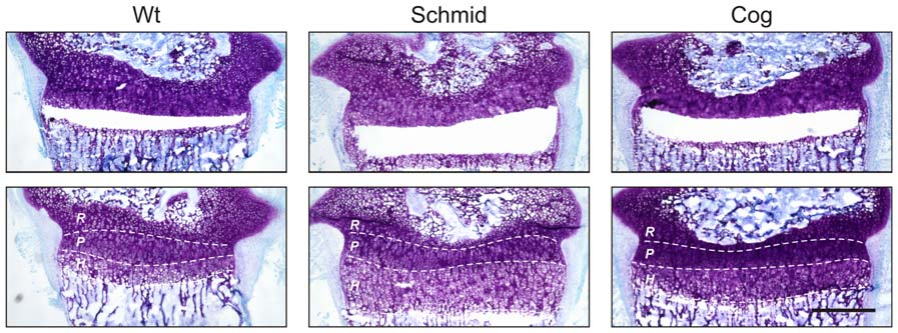
Microdissection of growth plate hypertrophic zones from wildtype and mutant mice. Representative 10 µm tibial growth plate cryosections from 14 day old wildtype (Wt), Schmid homozygous (Schmid), or Tg^cog^ (Cog) mice, stained with Toluidine Blue and Fast Green, and following microdissection of hypertrophic zones. Microdissected sections are shown in the upper panel; non-microdissected serial sections are shown in the lower panel. Dashed lines demarcate approximate growth plate zone boundaries: *R* – Resting Zone, *P* – Proliferative Zone, *H* – Hypertrophic Zone. Scale bar = 500 µm.

One tibia from each of three two week old Schmid, wildtype (Schmid background), Cog, and wildtype (Cog background) mice was used for hypertrophic zone microdissection (Figure 8). One tibia from each of three two week old Swiss White mice was used to microdissect hypertrophic and proliferative zones for wildtype growth plate zone gene signature analyses. Tibiae were dissected and embedded in Tissue-Tek OCT (Sakura Fine Technical), snap-frozen in isopentane, and stored at −80°C. 10 µm tibial sections were prepared using a cryostat (Leica CM1850), mounted on RNAse-free SuperFrost Plus slides (Biolab Scientific), and stored at −80°C. Immediately prior to microdissection, sections were fixed in 70% ethanol, rinsed in RNAse-free water, and dehydrated in 70%, 95%, and 100% ethanol for 30 seconds each, and air-dried. Schmid, Cog, and wildtype hypertrophic zones, and Swiss White hypertrophic and proliferative zones were microdissected from slides immobilized on the stage of an inverted light microscope (Leica DM IL) using an ophthalmic scalpel (Feather) fixed to the scanning xy-object guide. Light microscopy was performed using an Eclipse 80i light microscope (Nikon).

### RNA Preparation

Microdissected tissues were collected in TRIzol reagent (Invitrogen) for RNA extraction and purification, which was performed following the manufacturer's specifications. All total RNA samples were subjected to two rounds of linear amplification using the MessageAmp II aRNA Amplification Kit (Ambion), following the manufacturer's protocol. 100–150 ng of first-round amplified aRNA was used as template for each second round amplification. Following total RNA extraction and amplification, the yield, purity, and integrity of all RNA samples were validated by capillary electrophoresis with a Bioanalyzer 2100 (Agilent Technologies), using a Series II RNA 6000 Pico Kit (Agilent Technologies), according to the manufacturer's specifications. Amplified RNA samples (2 µg) were labelled with Cy3 using the ULS Fluorescent Labeling Kit for Agilent Arrays (Kreatech Diagnostics), according to the manufacturer's specifications. Fluorophore incorporation and yield were determined by spectrophotometry using a Nanodrop ND-1000 spectrophotometer (Thermo Fisher Scientific).

### PCR Analysis

Polymerase chain reaction (PCR) analysis was used to detect *Xbp1* splicing, and to generate templates for *in situ* hybridization (see below) riboprobes. To generate cDNA for PCR reactions, reverse transcriptions were performed on equal quantities of aRNA, using the Transcriptor High Fidelity cDNA Synthesis Kit (Roche Applied Science), according to the manufacturer's specifications. For *Xbp1* splicing, PCR was performed on equal quantities wildtype, Schmid, and Cog hypertrophic zone cDNA using primers flanking the *Xbp1* ER stress-responsive splice site [17]. To synthesize *in situ* hybridization riboprobes, PCR product were generated from cDNA as appropriate using primers spanning 3′-biased regions of the gene of interest ranging from 500–700 bp in length. Quantitative PCR (qPCR) was used for quantitative validation of the differential expression of specific genes between wildtype and mutant hypertrophic zones, and between wildtype proliferative and hypertrophic zones. qPCR was performed using the LightCycler 480 Probes Master kit (Roche Applied Science) in 10 µl reactions comprised of 5 µl LightCycler 480 Probes Master 2× concentrate, 50 ng cDNA, 100 nM UPL Probe (Roche Applied Science), and 200 nM each primer. qPCR primers were designed online (https://www.roche-applied-science.com/sis/rtpcr/upl/adc.jsp). Thermal cycling was conducted on a LightCycler 480 II qPCR machine (Roche Applied Science), as follows: initial denaturation at 95°C for 10 minutes, followed by 50 cycles of denaturation at 95°C for 30 seconds, annealing and polymerization at 60°C for one minute. qPCR data were analysed using LightCycler480 Software release 1.5.0 (Roche). All primer sequences are available on request.

### Microarray hybridizations and Bioinformatic Analyses

All aRNA samples were interrogated by microarray analysis using single-colour hybridizations to 44 K whole mouse genome microarrays, according to the manufacturer's specifications (Agilent Technologies). The arrays were then scanned at 5 µm resolution on a G2565BA DNA Microarray Scanner (Agilent Technologies), and the features extracted using Feature Extraction 9.5.3 software (Agilent Technologies). The raw data were then processed in statistical language R, using the limma package [83], [84], performing Normexp (offset = 50) for background correction and quantile normalization, with control probes removed and duplicate spots averaged. One array (ID 251486826813_3 (Proliferative)) was removed from the analysis due to quality control issues. Data were adjusted for multiple testing using the Benjamini and Hochberg's method to control false discovery rate. Gene set tests were performed within limma.

### *In Situ* Hybridization

*In situ* hybridisation was used to investigate the expression of candidate genes identified by microarray analyses. 10 µm tibial cryosections from one week old Schmid, Cog, and wildtype mice prepared as above were fixed in 4% paraformaldehyde (PFA) in PBS, and subjected to *in situ* hybridization, as briefly follows. DIG-labelled antisense RNA probes were prepared from PCR products subcloned into pGEMT-Easy cloning vector (Promega). Probes were hybridized to cryosections overnight at 65°C in hybridization buffer, comprised of 1× salts (0.2 M NaCl, 10 mM Tris-HCl pH 7.5, 1 mM Tris Base, 5 mM NaH_2_PO_4_.2H_2_O, 5 mM Na_2_HPO_4_, 50 mM EDTA), 1 mg/ml tRNA, 1× Denhardt's solution, 10% dextran sulphate, 50% deionized formamide. Hybridized cryosections were washed three times for 30 minutes at 65°C in washing solution (1× SSC, 50% formamide), then twice for 30 minutes at room temperature in TBTX (50 mM Tris-HCl pH 7.5, 150 mM NaCl, 0.1% Triton X-100). Cryosections were then treated with blocking solution (TBTX, 2% blocking reagent (Boehringer), 20% sheep serum) for ≥1 hour at room temperature, followed by antibody solution, comprised of 1∶1000 DIG anti-Fab fragments (Roche) in blocking solution overnight at room temperature. Next, cryosections were washed four times for 20 minutes in TBTX, and stained using 4.5 µl/mL nitro blue tetrazolium chloride (NBT) and 3.5 µl/mL 5-Bromo-4-chromo-3-indolyl phosphate (BCIP) in alkaline phosphatase staining buffer (100 mM NaCl, 50 mM MgCl_2_, 100 mM Tris pH 9.5) until strong, specific signal was detected.

### Histology

Mouse tibial growth plate sections were characterized histologically with Harris's Haematoxylin and Eosin Y (CliniPure, HD Scientific Supplies Pty Ltd), and for proteoglycans with Toluidine Blue and Fast Green (BDH Laboratory Supplies) counterstaining as previously described [85], but with the following exceptions. Because OCT-embedded cryosections were used rather than paraffin-embedded microtome sections, de-waxing steps involving xylene treatments were discarded. Instead, sections which had already been fixed in ethanol in preparation for microdissection were washed in water prior to staining, while fresh sections were fixed in 4% PFA for 10 minutes, and washed in water. For H&E staining, following haematoxylin treatment, sections were treated for 30 seconds each in 1% HCl and 1% ammonia water, and washed for 1 minute each under running tap water between and following each of these three treatments. For Toluidine Blue/Fast Green staining, following Toluidine Blue treatment and tap water rinse, sections were counterstained with Fast Green for three minutes, rinsed in tap water, and differentiated twice for one minute each in 100% isopropanol, before preparing for mounting in Pertex mounting medium (HD Scientific Pty Ltd) by treatment with xylene.

### TUNEL Staining

Terminal deoxynucleotidyl transferase dUTP nick end labelling (TUNEL) was used to detect cells undergoing DNA fragmentation at the end stages of apoptosis. Six tibial growth plate cryosections from each of three wildtype and three Schmid mice were assayed by TUNEL using the *In Situ* Cell Death Detection Kit, Fluorescein (Roche) according to the manufacturer's specifications. Wildtype cryosections treated with recombinant DNase I (Roche) for 20 minutes at room temperature prior to TUNEL staining were used as positive controls. 4′,6-diamidino-2-phenylindole (DAPI) was added to TUNEL reactions as a nuclear counterstain at a concentration of 200 ng/ml. Fluorescent microscopy was performed using an Axio Imager M1 fluorescence microscope (Zeiss). A ratio was calculated for the number of TUNEL-positive nuclei to the total number of nuclei per hypertrophic zone, to normalize for the length differential between wildtype and Schmid hypertrophic zones.

### Transmission Electron Microscopy

Specimens of growth cartilage collected from tibiae of 1 week old mice were fixed in Karnovsky's fixative supplemented with 0.7% (v/v) safranin O, a modified procedure used to enhance structural preservation and stabilization of cartilage proteoglycans [86]. After decalcification in 0.15 M EDTA samples were post-fixed in 1% osmium tetroxide/1.5% potassium ferrocyanide and embedded in Spurr's resin. Semi-thin sections (0.5 µm) were stained with 1% methylene blue. Ultra-thin sections were contrasted with uranyl acetate and Reynold's lead citrate and examined with a Philips 300 transmission electron microscope at 60 kv.

## Supporting Information

Figure S1 **qPCR analysis of** ***Col10a1*** **expression in wildtype and mutant hypertrophic zones.** qPCR performed for *Col10a1* on cDNA derived from hypertrophic zones microdissected from wildtype (Wt), homozygous Schmid (Schmid), or Tg^cog^ (Cog) tibial growth plates; N = 3, expression profiles expressed as Fold Difference versus Wt, error bars indicate standard deviation around the mean.
(TIF)

Figure S2 **Haematoxylin and eosin staining of wildtype and Schmid growth plates.** Representative 10 µm tibial growth plate cryosections from 7 day old wildtype (Wt) or Schmid homozygous (Schmid) mice, stained with haematoxylin and eosin. Dashed lines demarcate approximate growth plate zone boundaries: *R* – Resting Zone, *P* – Proliferative Zone, *H* – Hypertrophic Zone. Boxes inset show magnified representative areas of the HZs, to highlight the differential staining between Wt and Schmid in these zones. Scale bars = 500 µm.
(TIF)

Figure S3 **qPCR validating aRNA derived from wildtype mouse growth plate hypertrophic and proliferative zones.** qPCR performed on cDNA derived from hypertrophic zones (HZ) or proliferative zones (PZ) microdissected from wildtype (Wt) mouse tibial growth plates, using (A) HZ markers *Adamts1*, *Col10a1*, and *Mmp9*, and (B) PZ markers *Fmod*, *Gdf10*, and *Prelp*, and expressed as Fold Difference for Wt PZ versus Wt HZ; N = 3, error bars indicate standard deviation around the mean.
(TIF)

Figure S4 **Putative ERSE I element in the** ***Fgf21*** **promoter.** Portion of *Fgf21* genomic DNA sequence, from 334 bp upstream of the start codon, to 40 bp downstream of the start codon. Non-coding sequence denoted by lower-case font; coding sequence denoted by upper-case font. Start codon denoted by bold, blue font. Putative *Fgf21* ERSE I sequence denoted by bold, red font, with nucleotides matching the ERSE I consensus sequence underlined.
(TIF)

Table S1 **Schmid versus wildtype differentially expressed genes.**
(PDF)

Table S2 **Cog versus wildtype differentially expressed genes.**
(PDF)

Table S3 **Schmid versus wildtype GO analysis.**
(DOCX)

Table S4 **Cog versus wildtype GO analysis.**
(DOCX)

Table S5 **Cartilage-specific collagens and ECM components in schmid or cog versus wildtype.**
(DOCX)

Table S6 **ER stress sensors, their targets, and downstream pathways.**
(DOCX)

Table S7 **Wildtype hypertrophic zone gene expression signature.**
(DOCX)

Table S8 **Wildtype proliferative zone gene expression signature.**
(DOCX)

Table S9 **Wildtype hypertrophic zone gene expression signature GO analysis.**
(DOCX)

Table S10 **Wildtype proliferative zone gene expression signature GO analysis.**
(DOCX)
