# Supplementary material for: Transcriptional Profiling of Chondrodysplasia Growth Plate Cartilage Reveals Adaptive ER-Stress Networks That Allow Survival but Disrupt Hypertrophy
Source: PLoS One. 2011 Sep 15;6(9):e24600. doi: 10.1371/journal.pone.0024600 (PMC3174197; doi:10.1371/journal.pone.0024600)
Supplement: Table S1 — Schmid versus wildtype differentially expressed genes. (PDF) [file pone.0024600.s005.pdf]

Table S1 - Schmid versus Wildtype Differentially Expressed Genes

GenBank Accession	GeneName	Fold Diff	A	adj.P.Val
NM_020013	*Fgf21*	518.91	11.87	0.000
NM_009802	*Car6*	62.41	13.14	0.000
NM_001008424	*Cdsn*	31.66	12.08	0.000
NM_007837	*Chop*	23.52	13.94	0.000
NM_024440	*Derl3*	19.99	13.73	0.000
NM_053115	*Acox2*	19.54	10.70	0.000
XM_001002573	*4930438A08Rik*	18.66	9.41	0.000
NM_029639	*1600029D21Rik*	18.57	13.01	0.000
NM_008491	*Lcn2*	18.22	10.75	0.005
NM_010400	*H60*	17.96	10.71	0.003
NM_010924	*Nnmt*	17.55	12.04	0.002
NM_197986	*1110007F12Rik*	16.46	12.21	0.001
NM_009787	*Erp72*	16.37	12.43	0.001
NM_011858	*Odz4*	15.20	11.97	0.006
NM_008199	*H2-Bl*	14.64	10.64	0.000
NM_008382	*Inhbe*	14.28	11.69	0.004
BC060276	*Flnc*	13.83	13.01	0.000
NM_175096	*D5Ertd593e*	13.80	12.16	0.000
AK004394	*1110067I12Rik*	12.84	12.10	0.000
NM_008132	*Glrp1*	11.06	9.30	0.000
NM_145512	*Sft2d2*	10.91	12.62	0.005
NM_020519	*Slurp1*	10.70	9.45	0.003
NM_009626	*Adh7*	10.37	8.15	0.000
NM_007515	*Slc7a3*	10.28	14.10	0.000
NM_145953	*Cth*	10.08	12.40	0.007
NM_007752	*Cp*	10.01	11.33	0.002
NM_022018	*Niban*	9.91	11.23	0.000
NM_008630	*Mt2*	9.87	15.13	0.008
NM_010362	*Gsto1*	9.80	13.35	0.000
NM_011631	*Grp94*	9.61	16.49	0.000
NM_011819	*Gdf15*	9.35	12.28	0.000
NM_010884	*Ndrg1*	9.28	14.66	0.000
NM_009434	*Phlda2*	9.11	12.34	0.000
NM_201410	*Ugt1a6b*	8.94	10.76	0.006
AK017440	*Rian*	8.92	9.29	0.000
NM_028057	*Cyb5r1*	8.77	14.48	0.000
NM_008485	*Lamc2*	8.76	10.07	0.000
NM_024262	*1200011M11Rik*	8.52	8.68	0.003
NM_025505	*Blzf1*	8.49	13.99	0.001
NM_027399	*Steap1*	8.34	15.77	0.000
NM_178920	*Mal2*	8.26	11.16	0.000
NM_144554	*Trib3*	8.22	14.84	0.000
NM_145154	*Angptl6*	8.16	12.92	0.010
NM_026184	*Ero1lb*	8.15	10.65	0.004
NM_007498	*Atf3*	8.10	14.53	0.002
AK051789	*Hook1*	8.10	10.48	0.002
NM_001033189	*C77080*	8.06	8.53	0.000
NM_145535	*Sdcbp2*	7.93	11.60	0.000
NM_013760	*ERdj4*	7.88	12.90	0.000
NM_054087	*Slc19a2*	7.75	11.00	0.003
NM_022324	*Sdf2l1*	7.73	14.44	0.000
NM_177260	*Tmem154*	7.71	9.61	0.000
NM_008125	*Gjb2*	7.70	10.39	0.010
NM_029720	*Creld2*	7.66	17.30	0.000
NM_173866	*Gpt2*	7.64	13.63	0.003
NM_145599	*Tmem34*	7.59	11.61	0.019

NM_010479	*Hspa1a*	7.54	10.22	0.011
NM_010849	*Myc*	7.43	11.09	0.009
NM_001033302	*Gm129*	7.39	11.74	0.000
NM_008471	*Krt19*	7.33	10.19	0.000
NM_025860	*Ddx18*	7.33	11.07	0.009
NM_029278	*2610033H07Rik*	7.24	9.18	0.010
NM_008344	*Igfbp6*	7.13	11.20	0.001
AK035515	*1110002L01Rik*	7.01	7.87	0.000
NAP030221-1	*NAP030221-1*	7.00	14.74	0.020
NM_009988	*Cxadr*	6.98	9.15	0.000
NM_011905	*Tlr2*	6.90	10.94	0.001
NM_178595	*Ptrh1*	6.76	13.59	0.000
NM_001034851	*1810015C04Rik*	6.70	10.41	0.005
AK018789	*Ntrk2*	6.54	10.19	0.004
NM_001001446	*Cyp2c44*	6.41	10.79	0.001
AK089283	*Slc7a11*	6.41	10.43	0.001
AK031330	*AK031330*	6.35	8.83	0.004
NM_013512	*Epb4.1l4a*	6.35	12.74	0.004
NM_001033336	*Abcc4*	6.31	10.78	0.005
NM_177420	*Psat1*	6.28	12.40	0.006
AK032098	*2610042O14Rik*	6.26	8.02	0.002
NM_134111	*Eaf2*	6.26	10.40	0.002
NM_008393	*Irx3*	6.26	7.65	0.002
NM_026577	*Arl13b*	6.21	9.52	0.021
NM_172768	*Gramd1b*	6.21	9.90	0.001
NM_133743	*Lypd3*	6.16	9.64	0.000
NM_010800	*Bhlhb8*	6.16	10.90	0.001
AK138387	*1700001L19Rik*	6.11	8.88	0.000
NM_133930	*Creld1*	6.07	12.08	0.006
NM_025345	*0610037P05Rik*	6.07	8.41	0.011
AK010728	*Unc13a*	6.03	9.63	0.022
NM_145460	*Oxnad1*	6.02	12.48	0.003
NM_134086	*Slc38a1*	6.02	11.35	0.001
AK145182	*AI838057*	5.99	11.04	0.001
BC030317	*Lrrc17*	5.97	11.84	0.040
NM_134102	*Pla1a*	5.96	9.49	0.023
NM_026159	*Retsat*	5.92	10.54	0.012
NM_134052	*Adi1*	5.87	11.08	0.023
AK173292	*Nek1*	5.85	11.86	0.007
AK047398	*D10Bwg1379e*	5.85	11.36	0.013
AK042501	*AK042501*	5.83	7.87	0.008
NM_001005509	*Eif2a*	5.83	11.25	0.015
NM_207279	*Plcxd1*	5.83	10.28	0.000
NM_011851	*Nt5e*	5.78	13.04	0.015
NM_030704	*Hspb8*	5.78	11.43	0.047
XM_993103	*LOC667885*	5.77	7.87	0.004
NM_145835	*Lctl*	5.73	9.21	0.005
NM_010121	*Perk*	5.73	12.64	0.048
NM_007479	*Arf4*	5.66	14.49	0.005
NM_027545	*Cwf19l2*	5.63	11.55	0.002
NM_173876	*Clcn3*	5.55	9.55	0.005
NM_178772	*Aadacl1*	5.55	11.55	0.002
NM_153543	*Aldh1l2*	5.53	12.02	0.001
NM_001017983	*Foxred2*	5.52	8.70	0.015
NM_008242	*Foxd1*	5.51	11.43	0.006
NM_133943	*Hsd3b7*	5.49	12.68	0.007
NM_026929	*Chac1*	5.49	16.09	0.002
NM_009939	*Cops2*	5.44	8.57	0.004
NM_030685	*D3Ucla1*	5.43	16.99	0.036

NM_178747	*Gulo*	5.42	11.32	0.004
NM_011170	*Prnp*	5.42	14.33	0.005
NM_013562	*Ifrd1*	5.39	15.52	0.009
NM_139292	*Reep6*	5.39	11.06	0.010
NM_010442	*Hmox1*	5.38	14.84	0.001
NM_011081	*Piga*	5.36	11.51	0.000
XM_001002157	*Akap11*	5.36	10.91	0.013
XM_286373	*1810010H24Rik*	5.32	9.64	0.001
NM_011019	*Osmr*	5.32	11.60	0.009
NM_030138	*Centb2*	5.31	10.52	0.040
NM_013509	*Eno2*	5.30	9.54	0.000
XM_982330	*LOC675467*	5.30	10.39	0.019
NM_009210	*Smarca3*	5.28	7.98	0.007
NM_013560	*Hspb1*	5.27	13.81	0.004
XM_125867	*Utp20*	5.27	9.66	0.036
NM_009278	*Ssb*	5.27	11.52	0.029
AK220437	*BC016423*	5.25	9.77	0.049
XM_125902	*Xpot*	5.25	13.37	0.001
NM_021525	*Rcl1*	5.23	10.76	0.005
AK085838	*AK085838*	5.23	7.42	0.013
NM_010481	*Hspa9a*	5.19	15.48	0.004
BB657751	*BB657751*	5.16	11.80	0.001
NM_019812	*Sirt1*	5.13	10.06	0.048
AK086294	*Tcfcp2l1*	5.11	10.87	0.000
NM_027514	*Pvr*	5.11	13.52	0.000
NM_010475	*Hsd17b1*	5.10	9.67	0.000
NM_145354	*Nsun2*	5.09	15.26	0.012
NAP071006-1	*NAP071006-1*	5.08	13.23	0.044
NM_172727	*D330028D13Rik*	5.05	10.34	0.012
NM_026390	*Ubxd2*	5.05	8.50	0.044
NM_010324	*Got1*	5.03	14.47	0.000
NM_027959	*P5*	4.97	12.87	0.008
NM_008922	*Prim2*	4.97	9.88	0.022
XM_972868	*Erbb3*	4.96	10.80	0.005
NM_027296	*Trnt1*	4.96	13.02	0.018
NM_053086	*Nolc1*	4.95	11.47	0.039
NM_026643	*2410017P07Rik*	4.93	9.97	0.031
NM_030720	*Gpr84*	4.93	11.47	0.001
NM_028623	*Cst6*	4.89	9.86	0.001
NM_011348	*Sema3e*	4.89	8.71	0.000
NM_172713	*Sdad1*	4.87	11.00	0.005
AK015570	*4930474A20Rik*	4.86	9.49	0.035
NM_026358	*4930583H14Rik*	4.85	12.44	0.005
NM_019687	*Slc22a4*	4.85	10.63	0.021
NM_023184	*Klf15*	4.85	10.64	0.023
NM_001040005	*D11Ertd759e*	4.84	12.31	0.000
NM_173753	*A730024A03Rik*	4.84	10.19	0.010
NM_145450	*BC022687*	4.82	11.25	0.011
AK049625	*AK049625*	4.82	9.17	0.003
NM_025682	*Pspc1*	4.82	11.20	0.025
AK053583	*Ube1l2*	4.82	7.48	0.000
AK003980	*Lysmd3*	4.82	11.64	0.025
NM_011677	*Ung*	4.82	14.40	0.004
NM_007791	*Csrp1*	4.80	11.93	0.000
NM_008301	*Hspa2*	4.79	12.04	0.000
NM_145381	*Lactb2*	4.78	12.12	0.005
NM_011812	*Fbln5*	4.78	12.85	0.000
XM_973419	*5430411K18Rik*	4.76	11.77	0.008
NM_009731	*Akr1b7*	4.75	11.95	0.008

AK018172	*A230083H22Rik*	4.73	12.20	0.004
NM_021395	*Hyou1*	4.72	14.22	0.002
AK036738	*Aars*	4.70	9.54	0.032
NM_018813	*Cpsf3*	4.70	12.41	0.006
NM_001033525	*Kcnk6*	4.67	11.91	0.019
NM_024225	*Snx5*	4.66	14.83	0.002
NM_133829	*2210010L05Rik*	4.66	10.82	0.012
NM_183019	*Arhgef4*	4.66	9.17	0.001
NM_001039562	*Ankrd37*	4.66	11.01	0.045
BC007479	*Vps39*	4.64	11.96	0.043
NM_007981	*Acsl1*	4.63	12.60	0.001
NM_022310	*BiP*	4.62	17.53	0.003
AK013700	*2900055J20Rik*	4.62	9.87	0.001
AK020213	*Ddx3y*	4.61	9.28	0.017
NM_146133	*Golph3l*	4.61	11.71	0.018
AK037746	*Ywhaq*	4.59	7.80	0.004
NM_007404	*Adam9*	4.59	13.49	0.029
NM_022331	*Herpud1*	4.56	16.66	0.000
BY762059	*AW215868*	4.56	12.49	0.004
NM_015736	*Galnt3*	4.56	11.39	0.000
NM_011899	*Srp54*	4.55	14.63	0.029
AK084451	*AK084451*	4.55	9.24	0.015
NM_175329	*Ndg2*	4.54	13.82	0.002
AK083630	*Tom1l1*	4.54	9.80	0.000
NM_144885	*BC005624*	4.52	13.85	0.000
AK089994	*Fbxl2*	4.51	9.11	0.004
NM_026580	*Otub2*	4.51	10.61	0.014
NM_001039089	*Sel1h*	4.50	11.18	0.006
NM_024207	*Derl1*	4.50	14.87	0.005
NM_001039488	*4933421E11Rik*	4.49	8.57	0.011
NM_018748	*Golga4*	4.48	13.94	0.018
NM_178376	*Rraga*	4.48	14.31	0.006
NM_025969	*1700034H14Rik*	4.48	12.86	0.009
NM_019770	*Tmed2*	4.47	14.44	0.047
NM_145502	*Spfh1*	4.47	13.18	0.003
NM_172308	*Mthfd1l*	4.45	14.84	0.001
NAP028543-1	*NAP028543-1*	4.45	11.82	0.050
NM_019551	*Ttrap*	4.45	12.18	0.046
NM_029023	*Scpep1*	4.45	11.46	0.000
NM_027533	*Tspan2*	4.42	13.42	0.001
NM_011670	*Uchl1*	4.41	12.02	0.024
NM_145516	*Plekhb2*	4.41	10.55	0.001
NM_011901	*Taf7*	4.41	10.23	0.005
NM_172451	*Galnt6*	4.38	9.57	0.000
AK047419	*AK047419*	4.38	9.84	0.029
AK220437	*BC016423*	4.38	11.79	0.005
NM_133878	*Rcc1*	4.38	11.44	0.020
NM_175193	*Golph4*	4.37	12.29	0.034
BC052851	*Tor3a*	4.36	10.08	0.005
NM_027460	*5730438N18Rik*	4.36	13.34	0.002
NM_033398	*Ptdsr*	4.35	11.40	0.006
NM_025522	*Dhrs7*	4.33	16.42	0.005
XM_001001886	*4933401B06Rik*	4.32	9.96	0.008
NM_026545	*Psmd8*	4.30	14.68	0.010
NM_011113	*Plaur*	4.29	12.84	0.002
NM_013646	*Rora*	4.28	10.14	0.047
AK090134	*Anapc1*	4.28	12.13	0.006
NM_027139	*Taf9*	4.27	9.70	0.013
X15052	*Ncam1*	4.27	13.96	0.003

NM_146200	*Eif3s8*	4.27	10.54	0.007
NM_026149	*Nudcd1*	4.26	9.75	0.027
AK046738	*C030017B01Rik*	4.26	8.35	0.007
NM_139295	*Mcfd2*	4.25	15.03	0.017
NM_025443	*1810003N24Rik*	4.24	14.40	0.001
NM_145950	*BC010311*	4.23	10.45	0.018
AK029888	*Ankrd42*	4.22	10.05	0.037
NM_007416	*Adra1b*	4.20	9.58	0.001
NM_178397	*Ubxd8*	4.20	11.12	0.038
NM_001013825	*LOC435366*	4.19	9.32	0.002
NM_008378	*Impact*	4.18	13.24	0.016
NM_207659	*Hook3*	4.18	10.15	0.020
BC052532	*BC006779*	4.18	10.04	0.002
NM_153591	*Nars2*	4.18	9.45	0.031
NM_028133	*Egln3*	4.18	9.77	0.015
AK083983	*AK083983*	4.16	10.43	0.016
AK122422	*Unc13b*	4.15	10.35	0.006
NM_007836	*Gadd45a*	4.15	12.74	0.012
AK085741	*Cln5*	4.15	12.66	0.025
NM_024250	*Phf10*	4.15	16.76	0.001
NM_024258	*Usp16*	4.14	13.62	0.005
BC066082	*Wdr59*	4.14	9.18	0.002
NM_024208	*Echdc3*	4.12	10.09	0.006
NM_153075	*Catsper2*	4.11	9.84	0.008
NM_175088	*Mdfic*	4.11	10.40	0.020
NM_027208	*Bdh2*	4.11	12.32	0.009
NM_008654	*Myd116*	4.11	10.20	0.000
NM_016966	*Phgdh*	4.10	14.83	0.001
AK049968	*Anapc7*	4.10	7.86	0.010
NM_009230	*Soat1*	4.10	8.12	0.021
NM_177861	*Tmem67*	4.09	9.31	0.014
NM_001013768	*LOC272350*	4.08	10.44	0.000
NM_001013376	*Rpp38*	4.08	11.72	0.011
NM_178626	*Cdc42se2*	4.08	11.73	0.001
NM_025472	*1810032O08Rik*	4.08	13.37	0.010
NM_001015046	*Garnl4*	4.08	8.83	0.002
NM_172628	*Sh3tc2*	4.07	12.46	0.003
NM_001038592	*Glrx2*	4.07	7.60	0.001
NM_170728	*Ank3*	4.07	10.09	0.000
NM_028994	*Pck2*	4.06	14.57	0.000
NM_025289	*Tbrg1*	4.06	14.83	0.005
NM_029789	*Lass2*	4.05	13.75	0.007
NM_024201	*0610011N22Rik*	4.04	12.16	0.019
AK052759	*Dcn*	4.03	8.63	0.006
NM_008773	*P2ry2*	4.02	9.95	0.039
NM_029688	*Srxn1*	4.02	14.07	0.000
NAP103757-1	*NAP103757-1*	4.01	9.27	0.016
BC023820	*Fvt1*	4.00	12.82	0.011
NM_027285	*1700029I01Rik*	4.00	10.83	0.008
NM_008886	*Pms2*	4.00	10.47	0.022
NM_178640	*B3galnt2*	3.99	11.34	0.017
NM_029998	*6030458C11Rik*	3.99	10.66	0.009
NM_027896	*Coasy*	3.98	10.75	0.015
XM_486227	*Tln2*	3.98	8.49	0.018
NM_177462	*Zmym6*	3.98	10.64	0.034
NM_019631	*Tmem45a*	3.98	15.21	0.017
NM_025447	*1500031M22Rik*	3.98	12.68	0.007
NM_008929	*Dnajc3*	3.98	14.73	0.000
NM_029508	*Pcgf5*	3.98	11.91	0.020

NM_018868	*Nol5*	3.97	14.60	0.000
XM_980542	*Rab3gap2*	3.97	10.83	0.015
AK079181	*AK079181*	3.96	9.65	0.000
NM_007992	*Fbln2*	3.96	11.28	0.000
NM_025904	*1600012F09Rik*	3.95	11.69	0.004
NM_028766	*Tmem43*	3.94	14.73	0.000
BC039753	*Cdcp1*	3.94	9.57	0.001
NM_025706	*Tbc1d15*	3.93	12.47	0.005
NM_025644	*Exosc1*	3.93	12.27	0.001
NM_027569	*Spag9*	3.92	13.14	0.009
AK051509	*Ppm1d*	3.92	7.83	0.025
NM_025816	*Tax1bp1*	3.90	9.11	0.026
NM_029662	*Mfsd2*	3.89	8.88	0.004
NM_029001	*Elovl7*	3.89	11.78	0.007
NM_023646	*Dnaja3*	3.88	12.74	0.003
NM_015803	*Atp8a2*	3.88	10.26	0.011
NM_019437	*Rfk*	3.88	11.22	0.032
XR_002802	*LOC670106*	3.88	15.60	0.011
NAP057018-1	*NAP057018-1*	3.87	10.00	0.019
NM_011196	*Ptger3*	3.87	9.71	0.003
NM_198108	*BC023055*	3.86	10.81	0.002
BC051230	*BC051230*	3.84	13.17	0.001
AK017856	*5730564L20Rik*	3.84	7.51	0.001
AK037118	*B930037P14Rik*	3.84	9.12	0.021
NM_133769	*Cyfip2*	3.84	9.51	0.007
BC012405	*2310001H12Rik*	3.83	10.22	0.007
NM_152895	*Jarid1b*	3.82	9.66	0.013
NM_011464	*Spint2*	3.82	10.91	0.006
AK050117	*AK050117*	3.82	13.06	0.027
NM_008976	*Ptpn14*	3.81	11.75	0.002
NM_019425	*Gnpnat1*	3.81	10.87	0.003
NM_153589	*Tmem16b*	3.81	9.94	0.007
X61450	*Napb*	3.77	11.17	0.001
NM_029809	*2310014L17Rik*	3.77	7.93	0.002
AK136270	*RP23-3N21.1*	3.77	10.69	0.038
NM_028142	*Nsun4*	3.76	10.64	0.008
AV030849	*AV030849*	3.76	14.72	0.018
AK041561	*AK041561*	3.76	7.48	0.004
NM_028233	*Lrpprc*	3.76	12.12	0.017
NM_173395	*BC056923*	3.76	9.75	0.003
NM_021554	*Mettl9*	3.76	14.88	0.015
NM_011716	*Wfs1*	3.76	15.01	0.000
BC048160	*2210418O10Rik*	3.76	7.30	0.000
AK084295	*Thap6*	3.75	9.04	0.040
NM_009761	*Bnip3l*	3.75	14.52	0.008
NM_173052	*Serpinb1b*	3.73	7.76	0.007
NM_173363	*Eif5*	3.72	12.97	0.005
BC034056	*Dsg2*	3.72	9.03	0.000
AV036172	*AV036172*	3.72	13.02	0.035
NM_177806	*Prpf39*	3.71	10.44	0.041
NM_183149	*Zfp598*	3.70	13.24	0.010
NM_133993	*Pwp1*	3.70	13.60	0.024
NM_027439	*Atp6ap2*	3.70	12.99	0.044
NM_028782	*Prss15*	3.70	14.20	0.000
NM_007757	*Cpox*	3.70	14.13	0.000
NM_028958	*Taf7l*	3.69	8.94	0.000
NM_053178	*Acsbg1*	3.69	10.32	0.001
NM_010878	*Nck1*	3.69	10.88	0.006
NM_023719	*Txnip*	3.69	12.49	0.046

NM_008300	*Hspa4*	3.68	12.80	0.009
NM_178883	*Scyl1bp1*	3.68	11.13	0.026
NM_001033158	*Rasl12*	3.68	11.83	0.003
AK085036	*Mtx3*	3.67	8.09	0.005
NM_001029990	*Mett11d1*	3.67	9.46	0.008
NM_027881	*Osbpl3*	3.66	9.17	0.010
NM_026453	*Rbm13*	3.66	10.51	0.043
AK014499	*Exoc4*	3.65	8.43	0.015
NM_183423	*Pla2g12a*	3.64	10.62	0.000
NM_013749	*Tnfrsf12a*	3.64	14.55	0.029
NM_145371	*Eif2b1*	3.64	13.89	0.013
AV004411	*Ttc9c*	3.64	10.91	0.007
NM_028325	*Zcchc12*	3.63	10.54	0.001
NM_026437	*1810055E12Rik*	3.63	12.97	0.007
AK162197	*1190002N15Rik*	3.63	9.01	0.030
NM_029068	*Snx16*	3.63	8.10	0.002
NM_029735	*Eprs*	3.62	13.93	0.020
AK051206	*Dars*	3.62	9.08	0.011
XM_485258	*Mtap9*	3.62	10.03	0.000
NM_010840	*Mthfr*	3.62	12.39	0.003
NM_134133	*2010002N04Rik*	3.61	11.15	0.004
NM_183167	*AI987944*	3.61	10.40	0.023
NM_021461	*Mknk1*	3.61	9.72	0.010
NM_207161	*BC048355*	3.60	9.83	0.011
BC049168	*2300002D11Rik*	3.59	9.78	0.000
NM_029793	*Golga1*	3.59	11.89	0.017
AK050462	*C730049O14Rik*	3.59	8.82	0.002
NM_013898	*Timm8a1*	3.58	13.00	0.000
NM_146112	*Tnrc15*	3.58	9.41	0.020
NM_010395	*H2-T10*	3.58	12.23	0.005
NM_023697	*Rdh14*	3.58	12.28	0.032
NM_007808	*Cycs*	3.58	13.13	0.003
NM_011704	*Vnn1*	3.58	9.97	0.003
NM_021466	*Taf1a*	3.57	10.99	0.014
NM_178218	*Hist3h2a*	3.57	11.60	0.012
NM_029437	*Ckap5*	3.57	9.07	0.038
NM_023781	*1700020D05Rik*	3.55	9.82	0.018
NM_026405	*Rab32*	3.55	11.25	0.013
NM_013609	*Ngfb*	3.55	9.48	0.006
NM_145491	*Rhoq*	3.55	13.84	0.006
NM_033322	*Lztfl1*	3.55	9.80	0.002
NM_007616	*Cav1*	3.55	12.66	0.002
AK145748	*Gle1l*	3.55	13.42	0.000
BC032970	*2810026P18Rik*	3.55	14.56	0.036
XM_127272	*Ppp1r3g*	3.54	9.99	0.009
NM_026609	*Leprotl1*	3.54	12.44	0.006
NM_001024849	*LOC329575*	3.54	12.49	0.014
NM_010113	*Egf*	3.53	9.58	0.007
AK037592	*AK037592*	3.53	7.94	0.012
NM_027748	*Taf3*	3.53	8.82	0.028
NM_013819	*H2-M3*	3.53	10.49	0.007
NM_177203	*A730037C10Rik*	3.53	8.80	0.003
NM_001001932	*Eea1*	3.53	8.23	0.027
NM_008615	*Mod1*	3.51	13.12	0.021
NM_026203	*Ahi1*	3.51	11.08	0.002
NM_021414	*4631427C17Rik*	3.51	8.53	0.004
BC053524	*Ipo7*	3.50	12.80	0.004
AK083028	*Zfp422-rs1*	3.50	9.28	0.013
NM_029419	*9130022K13Rik*	3.50	9.53	0.042

NM_027547	*Prdm5*	3.49	10.34	0.020
NM_175266	*Epm2aip1*	3.49	11.39	0.036
NM_010123	*Eif3s10*	3.49	11.66	0.042
XR_004445	*LOC194985*	3.48	11.32	0.016
AK037051	*Myo1d*	3.48	8.91	0.019
BC080729	*Ddx10*	3.48	10.70	0.007
NM_001037926	*MGC117846*	3.47	9.94	0.013
NM_029802	*Arfip2*	3.47	11.66	0.022
NM_146215	*BC025546*	3.47	11.49	0.024
AK020360	*Ces7*	3.47	7.75	0.006
XM_001001171	*Trip11*	3.47	7.86	0.004
NM_015786	*Hist1h1c*	3.46	14.10	0.007
NM_153794	*4933403F05Rik*	3.46	9.15	0.005
NM_172574	*Pqlc3*	3.46	10.78	0.040
XM_994286	*Parp4*	3.46	8.97	0.009
NM_026345	*Mansc1*	3.46	11.15	0.000
NM_145568	*AA792894*	3.46	11.27	0.042
NM_008362	*Il1r1*	3.46	10.82	0.020
NM_009447	*Tuba4*	3.45	10.32	0.005
BC043044	*Psmc6*	3.45	12.86	0.022
NM_025964	*2310038H17Rik*	3.45	12.27	0.014
NM_001024618	*Cmya3*	3.45	11.33	0.008
NM_026396	*Bxdc2*	3.44	12.64	0.042
NM_023906	*Asb3*	3.44	10.70	0.004
AK034417	*9330188P03Rik*	3.44	10.82	0.030
NM_138589	*D7Wsu128e*	3.44	13.86	0.005
NM_175494	*Zfp367*	3.43	11.15	0.021
NM_011991	*Cops3*	3.43	12.84	0.026
NM_173400	*6230416J20Rik*	3.43	9.23	0.005
NM_010107	*Efna1*	3.43	10.84	0.003
NM_008249	*Tfb2m*	3.43	11.83	0.021
NM_009443	*Tgoln1*	3.43	15.35	0.000
NM_078478	*Ghitm*	3.43	15.48	0.004
XM_972834	*LOC664813*	3.43	12.38	0.028
AK082839	*AK082839*	3.42	8.76	0.011
BC091647	*Ankhd1*	3.42	10.36	0.023
NM_146034	*Mgea6*	3.42	11.47	0.002
NM_026373	*Cdk2ap2*	3.42	13.88	0.001
NM_023579	*Ranbp5*	3.42	11.66	0.009
BC048674	*1700125H20Rik*	3.42	9.66	0.030
NM_024194	*Lrrc40*	3.42	10.48	0.002
NM_144806	*Prpsap2*	3.41	13.05	0.020
NM_019766	*Ptges3*	3.41	9.94	0.038
NM_023788	*Mageh1*	3.41	10.68	0.001
XM_902605	*Ankrd11*	3.41	8.83	0.017
AV025990	*AV025990*	3.40	11.50	0.012
NM_001024806	*Cebpz*	3.40	10.19	0.007
NM_145959	*D15Ertd621e*	3.40	14.27	0.031
NM_028352	*Pgm3*	3.39	13.82	0.020
AK078849	*Mpp7*	3.39	10.24	0.020
NM_172015	*Iars*	3.39	13.33	0.001
NM_012010	*Eif2s3x*	3.39	13.40	0.029
NM_028355	*Tmem48*	3.39	10.57	0.016
NM_023554	*Nol7*	3.38	9.79	0.017
NM_013820	*Hk2*	3.38	16.44	0.008
NM_020491	*Sssca1*	3.38	12.39	0.005
AK037343	*A1300008O04Rik*	3.38	7.28	0.007
NM_008577	*Slc3a2*	3.38	16.97	0.000
AK083890	*D130051D11Rik*	3.37	10.19	0.002

NM_011484	*Stam*	3.37	13.76	0.005
NM_022889	*Pes1*	3.37	13.04	0.004
AK054461	*1700010H15Rik*	3.37	8.72	0.003
NM_021509	*Moxd1*	3.36	10.67	0.036
AK220252	*9130229H14Rik*	3.36	14.68	0.001
NM_175004	*Ptrh2*	3.36	14.04	0.001
NM_023191	*Wdr61*	3.36	14.18	0.012
NM_001017955	*LOC232875*	3.36	9.66	0.002
NM_178701	*Lrrc8d*	3.36	11.56	0.038
NM_026220	*Mfap1*	3.35	12.90	0.008
DV061293	*C330008K14Rik*	3.35	12.24	0.008
NM_134114	*5630401J11Rik*	3.35	8.35	0.008
XM_148904	*Osbp*	3.35	12.42	0.034
NM_011663	*U2af1-rs1*	3.34	10.57	0.026
NM_010897	*Nf1*	3.33	9.37	0.020
AK077950	*Setd5*	3.33	9.71	0.046
NM_198246	*Yars2*	3.33	10.20	0.007
NM_020048	*Trfp*	3.32	11.94	0.025
NM_027251	*2010107G23Rik*	3.32	9.90	0.001
NM_007771	*Cry1*	3.32	8.50	0.000
AK042768	*AK042768*	3.31	9.23	0.025
NM_178712	*Gpr64*	3.31	11.63	0.013
NM_008176	*Cxcl1*	3.31	10.06	0.001
NM_008102	*Gch1*	3.31	10.49	0.001
NM_138592	*Usp39*	3.31	10.05	0.035
NM_172051	*Tmcc3*	3.30	11.22	0.003
NM_013677	*Surf1*	3.30	13.13	0.006
NM_001014397	*LOC433804*	3.29	9.86	0.024
NM_011033	*Pabpc2*	3.29	12.70	0.017
NM_020494	*Ddx24*	3.29	10.10	0.025
NM_028809	*Arpc5l*	3.29	13.05	0.004
NM_026472	*Mki67ip*	3.28	14.79	0.000
NM_015751	*Abce1*	3.28	13.23	0.038
NM_001024708	*LOC436177*	3.28	12.73	0.002
AK030626	*Abcf3*	3.28	11.16	0.036
BC027262	*Mt1*	3.28	10.89	0.000
NM_028119	*Ddb2*	3.28	10.16	0.013
NM_133900	*Psph*	3.27	14.14	0.002
NM_009060	*Rgn*	3.27	10.25	0.002
NM_177382	*Cyp2r1*	3.27	9.46	0.006
NM_027115	*Mapk1ip1*	3.27	11.44	0.032
1110025P21	*1110025P21*	3.27	12.05	0.008
BI102035	*Trpc1*	3.27	10.05	0.020
AK032327	*Pdzd11*	3.26	7.26	0.001
NM_145600	*Zfp330*	3.26	14.97	0.032
NM_175393	*4930555G01Rik*	3.26	9.42	0.007
NM_027495	*Tmem144*	3.25	9.47	0.011
NM_016792	*Txnl1*	3.25	8.78	0.031
NM_146104	*Aph1a*	3.24	9.65	0.010
AK129472	*4930402E16Rik*	3.24	9.58	0.044
BC078630	*Nup98*	3.24	10.94	0.018
BC052715	*Lars*	3.24	16.52	0.000
AK047802	*AK047802*	3.24	8.08	0.045
NM_008214	*Hars*	3.23	13.51	0.047
NM_033325	*Loxl2*	3.23	11.81	0.024
NM_201639	*Dmn*	3.23	9.46	0.014
2210408K08	*2210408K08*	3.23	9.74	0.010
NM_010398	*H2-T23*	3.22	12.26	0.011
NM_172763	*BB114266*	3.22	7.98	0.012

AK164904	*Surf6*	3.22	9.21	0.000
AK037047	*AK037047*	3.22	8.78	0.008
AK018053	*5830474E16Rik*	3.22	10.21	0.035
NM_026030	*Eif2s2*	3.22	15.85	0.005
NM_018742	*Bet1l*	3.21	12.95	0.001
NM_134092	*Mtbp*	3.21	12.21	0.024
AK053595	*AK053595*	3.21	8.86	0.002
BC050879	*Adal*	3.20	10.76	0.028
AK031374	*Huwe1*	3.20	8.74	0.001
NM_026824	*Dus1l*	3.19	14.02	0.004
NM_172447	*A330021E22Rik*	3.19	10.51	0.003
NM_053183	*Ddx50*	3.19	11.46	0.027
NM_001024825	*LOC433520*	3.18	10.18	0.008
NM_177027	*Zcchc7*	3.18	9.84	0.004
NM_009071	*Rock1*	3.17	9.29	0.032
NM_028099	*Dusp11*	3.17	11.26	0.043
XM_129248	*Btaf1*	3.17	10.59	0.031
AK171114	*Krt80*	3.17	8.97	0.000
NM_009444	*Tgoln2*	3.17	14.24	0.003
NAP071064-1	*NAP071064-1*	3.17	14.37	0.034
NM_026276	*Aasdhppt*	3.17	11.74	0.021
NAP108117-1	*NAP108117-1*	3.16	10.70	0.038
NM_021890	*Fads3*	3.16	12.94	0.000
NM_021326	*Rbak*	3.15	10.63	0.045
NM_177342	*Taf5*	3.15	12.00	0.030
NM_011629	*Nr2c1*	3.15	10.65	0.014
NM_172294	*Sulf1*	3.14	15.67	0.008
AK052318	*D330027G24Rik*	3.14	9.58	0.011
NM_020586	*Herpud2*	3.14	12.02	0.004
NM_029963	*Mrps5*	3.14	10.44	0.004
NM_148952	*E2f4*	3.14	8.68	0.012
NM_008604	*Mme*	3.13	11.20	0.019
NM_009883	*Cebpb*	3.13	15.47	0.000
NM_025934	*Riok2*	3.13	11.69	0.011
NM_029017	*Mrpl47*	3.13	10.09	0.003
XM_001006108	*LOC673676*	3.13	8.69	0.017
NM_018829	*Ap3m1*	3.13	13.61	0.022
NM_027427	*Taf15*	3.13	8.29	0.014
NM_007508	*Atp6v1a*	3.13	11.78	0.034
NM_001040026	*Sco1*	3.13	9.62	0.004
NM_177718	*1600021P15Rik*	3.12	11.59	0.031
AK169742	*LOC14433*	3.12	10.39	0.033
AK014174	*Chka*	3.11	11.67	0.012
NM_025794	*Etfdh*	3.11	12.80	0.005
NM_025855	*Echdc1*	3.11	12.29	0.039
NM_144958	*Eif4a1*	3.11	13.61	0.026
NM_027134	*Mtfmt*	3.10	11.44	0.033
NM_133679	*Cryzl1*	3.10	13.85	0.007
NM_013896	*Timm9*	3.10	10.70	0.045
NM_029773	*4921517N04Rik*	3.09	11.01	0.042
NAP032508-1	*NAP032508-1*	3.09	9.96	0.001
BC037640	*Cwf19l1*	3.09	8.40	0.004
NM_172865	*Manea*	3.09	9.52	0.005
NM_026474	*Sugt1*	3.09	11.68	0.018
NM_177148	*B230317F23Rik*	3.08	9.11	0.008
NAP103151-1	*NAP103151-1*	3.08	8.57	0.015
AF191547	*AF191547*	3.08	8.42	0.021
NM_133242	*Rbm39*	3.08	11.86	0.032
NM_146068	*2310008H04Rik*	3.08	12.23	0.045

NM_011940	*Ifi202b*	3.08	10.27	0.014
NM_133349	*Zfand2a*	3.08	14.91	0.001
AK013921	*LOC627905*	3.08	13.19	0.021
AK035049	*9430079B08Rik*	3.08	9.80	0.024
NM_172262	*Aof1*	3.08	11.72	0.006
XM_885832	*LOC621431*	3.08	9.69	0.008
NM_175472	*Zcchc11*	3.07	8.54	0.004
NM_175225	*Tasp1*	3.07	10.51	0.020
NM_026240	*Gramd3*	3.07	10.68	0.020
NM_007439	*Alk*	3.07	7.06	0.013
NM_146074	*Tfb1m*	3.06	11.55	0.011
NM_027652	*D5Wsu178e*	3.06	11.20	0.050
AK044024	*Eml5*	3.06	8.77	0.001
NM_152134	*Homer1*	3.06	8.93	0.046
NM_173752	*1110067D22Rik*	3.06	13.38	0.024
AK084857	*Bsdc1*	3.06	10.21	0.046
NM_026642	*Trmt12*	3.06	9.31	0.046
NM_172310	*Tarsl2*	3.05	10.42	0.007
NM_133225	*Acbd3*	3.05	14.14	0.003
BC051161	*6330505F04Rik*	3.05	8.45	0.009
BC022619	*Phr1*	3.05	11.87	0.044
NM_177356	*Lamp3*	3.05	10.88	0.021
BF535186	*Crebl2*	3.04	9.53	0.006
NM_021347	*Gsdm1*	3.04	8.82	0.028
AK019896	*Invs*	3.04	8.76	0.022
XM_135146	*Dnajc13*	3.04	10.79	0.037
NM_008977	*Ptpn2*	3.03	14.34	0.011
NM_180678	*Gars*	3.03	11.01	0.014
NM_172589	*Lhfpl2*	3.03	9.25	0.008
NM_026932	*Ebna1bp2*	3.03	16.06	0.000
NM_025476	*2410005O16Rik*	3.03	10.69	0.009
NM_134141	*Ciapin1*	3.02	15.19	0.003
AK035848	*AI449023*	3.02	7.92	0.032
NM_013747	*Golga5*	3.02	13.49	0.034
NM_207269	*D330050I23Rik*	3.01	13.53	0.005
AK079702	*AK079702*	3.01	9.11	0.009
XM_894031	*Raph1*	3.01	10.77	0.035
NM_019926	*Mtm1*	3.01	8.94	0.044
NM_172857	*Exdl1*	3.01	9.43	0.008
AK012966	*Mlstd2*	3.00	13.39	0.015
NM_028769	*Syvn1*	3.00	16.29	0.002
NM_172644	*Dars2*	3.00	9.28	0.035
NM_026009	*2610204L23Rik*	3.00	15.13	0.008
NM_146097	*Cbwd1*	3.00	11.99	0.024
NM_027164	*Lrrc27*	3.00	9.53	0.005
NM_027977	*2310001A20Rik*	3.00	13.47	0.045
AK077374	*2310047D13Rik*	2.99	9.93	0.010
NM_029103	*Armet*	2.99	17.28	0.003
NM_026260	*4930521E07Rik*	2.99	10.91	0.008
NM_029478	*Tmem49*	2.99	15.19	0.002
NM_026375	*Ahctf1*	2.99	12.41	0.031
NM_025391	*Nip7*	2.99	14.73	0.009
BC013561	*BC013561*	2.98	14.24	0.005
NM_024221	*Pdhb*	2.98	13.03	0.003
NAP020770-001	*NAP020770-001*	2.98	13.29	0.036
NM_027159	*Ccdc115*	2.98	10.60	0.007
NM_172303	*Phf17*	2.98	11.67	0.018
NM_008187	*Gtl3*	2.98	12.79	0.020
NM_013754	*Insl6*	2.98	9.83	0.041

NM_009760	*Bnip3*	2.97	15.65	0.050
AK036244	*AK036244*	2.97	9.28	0.025
NM_033560	*Vps37a*	2.97	11.65	0.014
NM_001033156	*Fbxo33*	2.97	9.59	0.035
XM_975420	*Arfgef1*	2.97	12.96	0.043
NM_013915	*Zfp238*	2.97	12.05	0.020
AK081751	*AK081751*	2.96	11.19	0.012
NM_016670	*Pknox1*	2.96	11.50	0.017
AK037402	*AK037402*	2.96	8.65	0.020
NM_024124	*Hdac9*	2.96	9.08	0.012
NM_024222	*Stt3b*	2.96	13.96	0.005
NM_023200	*Ppp1r7*	2.95	8.05	0.047
NM_021354	*Drg2*	2.95	12.30	0.003
NM_134129	*Prpf19*	2.95	10.25	0.013
NM_009884	*Cebpg*	2.95	11.86	0.024
BC058687	*Maea*	2.95	14.58	0.000
NM_026337	*Sltm*	2.95	12.61	0.017
NM_025482	*Tpd52l2*	2.94	13.32	0.003
NM_023214	*Slc30a7*	2.94	10.70	0.009
NAP102621-1	*NAP102621-1*	2.94	8.78	0.008
AK054284	*AK054284*	2.94	9.12	0.005
NM_018824	*Slc23a2*	2.94	11.00	0.026
NM_001029876	*AK122209*	2.94	9.84	0.036
NM_008532	*Tacstd1*	2.94	9.76	0.000
NM_133661	*Slc6a12*	2.94	11.60	0.027
NM_175250	*2810007J24Rik*	2.93	12.10	0.017
AK079318	*9630020I17Rik*	2.93	7.77	0.030
XM_130646	*Arfgef2*	2.93	10.44	0.027
AK012573	*Nr2f2*	2.93	8.54	0.022
NM_026487	*Atad1*	2.92	12.96	0.026
NM_016851	*Irf6*	2.92	9.68	0.000
NM_001033293	*Uap1l1*	2.92	12.40	0.017
NM_022019	*Dusp10*	2.92	9.30	0.004
NM_013610	*Ninj1*	2.92	13.79	0.014
NM_212441	*Acsm3*	2.92	8.96	0.004
AK029387	*B230312I18Rik*	2.92	8.88	0.020
NM_009035	*Rbpsuh*	2.92	11.20	0.024
NAP061138-1	*NAP061138-1*	2.92	16.76	0.005
NM_001039483	*Tmco1*	2.91	14.63	0.020
NM_013924	*Abt1*	2.91	12.44	0.003
NM_021330	*Acp1*	2.91	9.84	0.007
NM_025346	*Rmnd5b*	2.91	11.24	0.040
NM_133907	*Ube3c*	2.91	11.86	0.023
AK015074	*AK015074*	2.91	13.34	0.008
NM_146047	*C130052I12Rik*	2.91	16.00	0.033
NM_172405	*Ccdc98*	2.91	8.21	0.014
AK129163	*BC046331*	2.91	9.63	0.020
NM_026574	*Inoc1*	2.90	10.37	0.042
AK088678	*Hnrpc*	2.90	10.62	0.028
NM_010477	*Hspd1*	2.90	16.84	0.025
NM_138677	*Edem1*	2.90	12.65	0.005
NM_178761	*Zfp672*	2.90	10.64	0.007
NM_010391	*H2-Q10*	2.90	12.00	0.008
AK087219	*AK087219*	2.90	9.41	0.011
U73445	*Dld*	2.90	13.13	0.025
NM_008576	*Abcc1*	2.90	10.78	0.007
NM_007916	*Ddx19a*	2.90	9.02	0.028
BC076612	*3110043O21Rik*	2.90	10.64	0.040
AK029366	*2700094F01Rik*	2.90	8.81	0.035

BC064456	*Zfp383*	2.90	10.80	0.014
NM_028112	*Seh1l*	2.89	10.41	0.043
BC089370	*2610034N15Rik*	2.89	11.70	0.014
NM_010171	*F3*	2.89	9.05	0.000
XM_981751	*AA987161*	2.89	9.60	0.022
NM_011455	*Serpinb9g*	2.89	9.07	0.005
NM_028398	*2900006B13Rik*	2.89	12.75	0.004
XM_138712	*Zfp72*	2.89	9.58	0.022
AK043908	*Dedd2*	2.89	8.00	0.001
BC062107	*A430041B07Rik*	2.88	12.11	0.033
XM_144820	*Tigd2*	2.88	9.88	0.011
AK037919	*AK037919*	2.88	8.48	0.010
NM_172572	*Rhbdf2*	2.87	11.69	0.005
NM_133976	*Imp3*	2.87	13.35	0.008
NM_026130	*Srpr*	2.87	10.96	0.010
AK029617	*BB116930*	2.87	9.13	0.036
NM_011890	*Sgcb*	2.87	10.75	0.023
NM_023243	*Ccnh*	2.86	13.41	0.002
NM_026195	*Atic*	2.86	10.83	0.032
NM_199201	*Clm3*	2.86	9.50	0.028
NM_138314	*Nme7*	2.86	9.55	0.008
NM_019564	*Htra1*	2.86	15.50	0.015
NM_008200	*H2-D4*	2.86	9.95	0.001
NM_172665	*Pdk1*	2.86	12.19	0.042
NM_024468	*Trim39*	2.86	11.05	0.011
AK018382	*Zzef1*	2.86	8.41	0.035
AK165551	*Prdx4*	2.86	9.13	0.014
NM_023311	*Yipf5*	2.86	16.14	0.003
NM_145490	*BC011426*	2.86	9.15	0.008
NM_172429	*Smndc1*	2.86	14.55	0.010
AK034071	*AK034071*	2.85	11.51	0.040
NM_145135	*Rnh1*	2.85	10.85	0.023
NM_013912	*Apln*	2.84	11.50	0.049
NM_023671	*Clns1a*	2.84	12.28	0.042
AK004937	*Bbs10*	2.84	10.01	0.010
XM_897688	*D14Abb1e*	2.83	9.11	0.009
AK044799	*AK044799*	2.83	8.92	0.002
NM_008408	*Stt3a*	2.83	10.74	0.004
NM_133752	*Opa1*	2.83	10.83	0.007
NM_008860	*Prkcz*	2.83	11.45	0.012
AK030450	*AK030450*	2.83	8.86	0.030
NM_013559	*Hsp110*	2.83	15.06	0.000
AK043395	*Zfp612*	2.83	9.18	0.007
NAP102548-1	*NAP102548-1*	2.83	9.78	0.024
BC089626	*2310046A06Rik*	2.83	11.60	0.023
NM_177325	*Tsr1*	2.83	11.06	0.004
AK052831	*AK052831*	2.82	8.04	0.040
NM_173747	*Gpkow*	2.82	9.73	0.042
NM_022981	*Zfp110*	2.82	11.72	0.038
NM_134095	*D15Wsu75e*	2.82	12.47	0.003
NM_011274	*C80913*	2.82	10.51	0.011
NM_010145	*Ephx1*	2.82	10.59	0.011
AK085277	*9930033D15Rik*	2.81	10.02	0.019
NM_022022	*Ube4b*	2.81	11.02	0.005
NAP028877-1	*NAP028877-1*	2.81	10.08	0.015
XM_194572	*Etohi1*	2.81	10.32	0.041
NM_053207	*Egln1*	2.80	10.09	0.044
NM_016766	*Mcrs1*	2.80	11.09	0.035
NM_001007590	*LOC433886*	2.80	8.83	0.000

NM_010081	*Dst*	2.80	11.01	0.011
NM_145443	*L2hgdh*	2.80	11.58	0.045
AK162455	*Asxl2*	2.80	11.14	0.032
BC003834	*Fbxw2*	2.80	10.01	0.035
NM_133806	*Uap1*	2.80	12.56	0.047
NM_026194	*1810074P20Rik*	2.80	10.91	0.015
NM_001024602	*AW555464*	2.79	13.87	0.046
NM_145444	*Acot5*	2.79	9.34	0.001
BC026442	*Zbtb8os*	2.79	8.59	0.005
NM_013763	*Tbl2*	2.79	10.39	0.002
NAP028210-1	*NAP028210-1*	2.79	8.60	0.046
NM_024187	*U2af1*	2.79	12.74	0.023
NM_010166	*Eya3*	2.78	11.34	0.012
AK167515	*Mobk1b*	2.78	13.69	0.016
NM_001033167	*3110004L20Rik*	2.78	10.91	0.030
NM_080554	*Psmd5*	2.78	12.77	0.015
NAP046120-1	*NAP046120-1*	2.78	8.93	0.011
XM_146277	*Gm501*	2.78	8.41	0.001
AK053748	*AK053748*	2.77	8.25	0.012
AK040360	*Capn3*	2.77	9.13	0.014
NM_026168	*Ergic2*	2.77	11.51	0.045
NM_023784	*Yipf7*	2.77	9.24	0.003
NM_026157	*Papd1*	2.77	12.53	0.010
NM_138686	*Cys1*	2.76	8.35	0.008
NM_008161	*Gpx3*	2.76	11.18	0.032
NM_011584	*Nr1d2*	2.76	10.97	0.046
NM_198003	*1300003B13Rik*	2.76	9.70	0.013
NM_009635	*Avil*	2.76	9.84	0.020
NM_024180	*Ormdl2*	2.76	11.81	0.020
BC021460	*Lsm1*	2.75	12.72	0.004
NM_178398	*Wipi2*	2.75	10.81	0.048
NM_027266	*Rg9mtd3*	2.75	10.40	0.037
NM_134042	*Aldh6a1*	2.75	14.19	0.041
NM_177251	*D830014E11Rik*	2.74	9.17	0.009
NM_025370	*1110018J18Rik*	2.74	13.62	0.006
NM_080289	*Grhpr*	2.74	14.20	0.002
BB160563	*BB160563*	2.74	9.55	0.014
AK021082	*C030014O09Rik*	2.74	9.15	0.016
NM_181406	*Rarsl*	2.73	10.41	0.042
XM_622789	*C230094A16Rik*	2.73	8.50	0.008
AK041028	*Scfd1*	2.73	11.12	0.008
BC064745	*Wdr33*	2.73	12.36	0.042
NM_023168	*Grina*	2.73	11.02	0.029
NM_027404	*Bag5*	2.72	13.35	0.019
NM_139305	*Car9*	2.72	10.05	0.031
NM_019565	*Zfp386*	2.72	7.82	0.001
NAP058332-1	*NAP058332-1*	2.72	17.94	0.015
AK161694	*AW495713*	2.72	9.92	0.032
NAP007437-001	*NAP007437-001*	2.71	13.79	0.005
NM_026536	*Atp5s*	2.71	10.61	0.049
NM_007805	*Cyb561*	2.71	10.75	0.041
AK015002	*Ppp3cc*	2.71	8.89	0.006
NM_008360	*Il18*	2.71	9.93	0.005
NM_001024917	*B3bp*	2.71	9.50	0.022
NM_194462	*Akap9*	2.71	13.30	0.020
NM_025442	*Alg5*	2.71	14.42	0.007
NM_153567	*5033405K12Rik*	2.70	13.94	0.001
NM_001033261	*Psrc2*	2.70	8.84	0.046
AK038874	*Cpxm2*	2.70	9.00	0.001

NM_019703	*Pfkp*	2.70	10.66	0.005
NM_013911	*Fbxl12*	2.70	11.68	0.034
NM_026641	*Ift80*	2.70	8.31	0.046
NM_138607	*D0HXS9928E*	2.70	11.80	0.049
NM_008537	*Amacr*	2.70	11.37	0.045
NM_030013	*Cyp20a1*	2.70	10.74	0.020
XM_620516	*Rkhd3*	2.70	10.95	0.021
XM_892824	*LOC628101*	2.69	8.96	0.009
NM_013703	*Vldlr*	2.69	13.84	0.001
NM_023493	*Cml5*	2.69	8.58	0.007
NM_054057	*Prosc*	2.69	13.32	0.022
NM_172267	*Phyhd1*	2.69	10.91	0.044
NM_001003910	*Gnn*	2.69	8.89	0.001
XM_001005092	*Wdr43*	2.69	12.44	0.006
NM_010120	*Eif1a*	2.69	13.55	0.018
NM_211358	*Slc35c1*	2.69	13.94	0.048
NM_133995	*Upb1*	2.69	9.23	0.020
BC043315	*3230401M21Rik*	2.68	10.17	0.003
NM_010760	*Magoh*	2.68	16.00	0.017
NM_025962	*Mmachc*	2.68	10.17	0.030
NM_033320	*Glce*	2.68	9.10	0.009
NM_023733	*Crot*	2.68	10.51	0.036
NM_008484	*Lamb3*	2.68	10.49	0.003
NM_172722	*C330023M02Rik*	2.67	12.92	0.010
NM_010394	*H2-Q7*	2.67	11.88	0.008
NM_027870	*Armcx3*	2.67	14.05	0.006
NM_026538	*Ddx56*	2.67	13.03	0.019
NM_019920	*Map2k1ip1*	2.67	16.72	0.040
NM_178736	*Elmod2*	2.67	12.37	0.025
NAP027441-1	*NAP027441-1*	2.67	9.21	0.013
NM_023256	*Krt20*	2.67	9.87	0.008
NM_011826	*Hax1*	2.67	16.00	0.000
AK012596	*Tdrkh*	2.67	10.67	0.029
AK043732	*4833446K15Rik*	2.66	8.62	0.003
NM_027101	*Polr2d*	2.66	15.38	0.036
NM_011603	*Tbpl1*	2.66	14.00	0.014
NM_009257	*Serpinb5*	2.66	8.79	0.000
NM_018808	*Dnajb1*	2.66	11.70	0.046
NM_026483	*Mphosph10*	2.66	10.93	0.005
XM_355303	*1700029F09Rik*	2.66	10.50	0.046
NM_026933	*Triap1*	2.65	13.37	0.030
NM_153566	*Yrdc*	2.65	11.49	0.011
NM_008536	*Tm4sf1*	2.65	14.14	0.018
NM_172684	*Rsbn1*	2.65	12.21	0.042
NM_011710	*Wars*	2.65	13.51	0.004
NM_178392	*Snapc1*	2.65	12.15	0.029
NM_025804	*Tcf25*	2.65	15.18	0.002
NM_011802	*Clpx*	2.65	12.57	0.037
XM_001005515	*Fpgt*	2.65	9.33	0.020
NM_133905	*Papd4*	2.65	14.24	0.020
NM_026504	*Coq5*	2.64	9.55	0.006
NM_001039209	*LOC195531*	2.64	9.87	0.016
NM_178939	*Pdrg1*	2.64	16.30	0.007
XR_003524	*LOC435752*	2.64	13.13	0.032
NM_010392	*H2-Q2*	2.64	12.63	0.004
BC029127	*BC029127*	2.64	8.13	0.012
XM_902907	*LOC628308*	2.64	9.59	0.005
NM_201600	*Myo5b*	2.64	8.17	0.007
AK043633	*AK043633*	2.64	7.68	0.001

NM_178605	*D13Wsu177e*	2.64	11.58	0.025
NM_029394	*Snx24*	2.64	10.72	0.027
NM_027654	*Pcgf6*	2.64	10.14	0.020
AK161163	*Gria3*	2.64	8.94	0.006
NM_013692	*Klf10*	2.63	12.00	0.014
NM_198606	*Wdsof1*	2.63	12.95	0.005
NM_009834	*Ccrn4l*	2.63	11.48	0.011
NM_080467	*Atp6v0a4*	2.63	8.90	0.013
NM_011538	*Tbx6*	2.62	10.68	0.043
NM_028356	*Zbtb25*	2.62	11.28	0.041
NM_153568	*BC031901*	2.62	8.55	0.025
NM_053158	*Mrpl1*	2.62	10.35	0.025
NM_007573	*C1qbp*	2.62	15.86	0.028
NM_009085	*Rpo1-1*	2.62	14.75	0.008
BC004022	*BC004022*	2.62	10.92	0.017
BC055860	*2600001A11Rik*	2.62	8.72	0.005
NM_001033300	*Gmps*	2.62	9.32	0.044
NM_001001978	*LOC380687*	2.62	17.47	0.001
NM_153780	*2610044O15Rik*	2.62	10.45	0.014
NM_173350	*Osbpl9*	2.61	15.72	0.030
NM_009829	*Ccnd2*	2.61	12.83	0.010
NM_145138	*Nek9*	2.61	14.28	0.035
AK033525	*AK033525*	2.61	9.19	0.006
NM_024478	*Grpel1*	2.61	17.79	0.001
NM_009196	*Slc16a1*	2.61	13.67	0.048
NM_024284	*Hagh*	2.61	13.35	0.001
AK079958	*AK079958*	2.61	7.74	0.047
BC008626	*Icam1*	2.61	10.63	0.001
NM_008667	*Nab1*	2.61	10.73	0.029
XM_986525	*LOC666173*	2.61	10.97	0.010
BC062197	*Sirpa*	2.61	15.00	0.008
NM_028584	*Marveld3*	2.60	9.39	0.006
NM_025334	*Txndc12*	2.60	11.76	0.024
NM_026489	*Hormad1*	2.60	9.05	0.033
NM_026368	*5830433M19Rik*	2.60	9.99	0.037
XR_004886	*LOC676153*	2.60	11.60	0.016
XM_618738	*Lats1*	2.60	9.39	0.040
AK163685	*Pcyt1a*	2.60	11.09	0.002
NM_178795	*Hisppd2a*	2.60	9.91	0.021
AK147179	*Strap*	2.60	12.34	0.007
NM_028930	*Tmc5*	2.60	8.44	0.005
NM_011498	*Bhlhb2*	2.59	11.74	0.017
NM_145124	*Mib2*	2.59	11.79	0.008
NM_007983	*Faf1*	2.59	14.46	0.006
NM_011541	*Tcea1*	2.59	12.48	0.006
AK035797	*Adamts18*	2.59	10.24	0.036
NM_025854	*1700023B02Rik*	2.59	11.84	0.035
NM_152812	*Otud6b*	2.59	10.96	0.040
NM_015734	*Col5a1*	2.59	16.28	0.041
NM_207650	*Dtna*	2.59	9.46	0.008
NM_025780	*Thap2*	2.59	9.62	0.027
NM_053069	*Atg5*	2.58	13.24	0.009
NM_021511	*Rrs1*	2.58	12.18	0.015
NM_028292	*Ppme1*	2.58	12.70	0.015
AY590892	*Dmxl1*	2.58	9.22	0.024
BC056501	*Slc45a4*	2.58	14.31	0.001
NM_008650	*Mut*	2.58	11.53	0.005
NM_172402	*Slc25a32*	2.58	11.01	0.046
NM_023290	*Mkrn2*	2.58	11.97	0.007

NM_026440	*Rnmt*	2.58	11.91	0.016
NM_024439	*H47*	2.57	16.45	0.041
AK145080	*4933421O10Rik*	2.57	11.16	0.033
NM_001034893	*LOC435970*	2.57	9.06	0.002
NM_181411	*Aftph*	2.57	10.99	0.034
NM_026198	*2010200O16Rik*	2.57	11.56	0.006
AK159148	*Slc35d2*	2.57	10.06	0.016
NM_001010825	*D5Ertd40e*	2.57	9.82	0.004
NM_019490	*Vdp*	2.56	11.98	0.004
NM_028817	*Acsl3*	2.56	11.47	0.033
NM_175252	*6720457D02Rik*	2.56	8.67	0.014
AK034645	*Mterfd1*	2.56	7.65	0.004
NM_009142	*Cx3cl1*	2.56	10.16	0.002
XM_987722	*LOC665775*	2.56	9.58	0.007
NM_178069	*Lsg1*	2.56	10.01	0.019
NM_018886	*Lgals8*	2.56	13.74	0.025
AK052753	*Ncor1*	2.56	7.57	0.020
AK054249	*Lrrc8b*	2.55	9.85	0.016
AK147204	*Cyld*	2.55	9.98	0.022
NM_023429	*Ociad1*	2.55	12.37	0.008
NM_053075	*Rheb*	2.55	15.94	0.039
NM_172552	*Tdg*	2.55	14.65	0.009
NM_024251	*2010301N04Rik*	2.55	10.84	0.042
AK050568	*C79248*	2.55	9.28	0.001
NM_027478	*5730494N06Rik*	2.55	14.42	0.026
NM_027269	*1110034A24Rik*	2.55	9.23	0.007
NM_027642	*Phf6*	2.55	9.02	0.038
AK015077	*AK015077*	2.54	7.21	0.007
NM_019667	*Stam2*	2.54	11.36	0.043
NM_012011	*Eif2s3y*	2.54	14.44	0.020
AK161721	*Tlcd1*	2.54	9.33	0.007
NM_007570	*Btg2*	2.53	13.08	0.008
NM_172383	*6330530A05Rik*	2.53	10.31	0.025
AK078860	*Hoxb9*	2.53	10.51	0.001
BC087944	*Saal1*	2.53	10.47	0.043
NM_173745	*Dusp18*	2.53	10.68	0.035
NM_178690	*Rab3gap1*	2.53	10.20	0.019
XM_001004952	*9430008C03Rik*	2.53	13.41	0.041
NM_029775	*Dcun1d5*	2.53	14.33	0.016
NM_013470	*Anxa3*	2.53	15.18	0.023
NM_178632	*Ints7*	2.52	9.82	0.005
AK052864	*D830012I24Rik*	2.52	8.27	0.023
NM_011656	*Tuft1*	2.52	10.86	0.032
NM_134025	*Pex12*	2.52	11.23	0.013
AK045871	*4932415D10Rik*	2.52	8.99	0.009
NM_199199	*AI316787*	2.52	10.41	0.043
NM_021274	*Cxcl10*	2.52	9.45	0.041
NM_009289	*Slk*	2.52	10.22	0.033
NM_019909	*LOC56628*	2.52	14.61	0.001
AK039955	*AK039955*	2.52	9.67	0.041
NM_021388	*Extl2*	2.52	11.87	0.048
NM_016748	*Ctps*	2.52	13.39	0.002
NM_026344	*Dph2*	2.52	11.07	0.028
NM_019738	*Nupr1*	2.52	17.70	0.002
NM_030080	*Aibzip*	2.52	9.48	0.027
AK014074	*3110023G01Rik*	2.51	9.71	0.002
NM_029582	*Txndc11*	2.51	10.28	0.023
AK085374	*AK085374*	2.51	8.83	0.004
NM_174868	*C030011O14Rik*	2.51	9.46	0.000

NM_172054	*Txndc9*	2.51	13.70	0.003
NM_080553	*Itpr3*	2.51	13.80	0.018
NM_009507	*Vhlh*	2.51	10.94	0.017
NM_173867	*Rcc2*	2.51	14.02	0.002
AK090393	*Tgfbr2*	2.51	8.60	0.025
NM_008162	*Gpx4*	2.51	17.62	0.003
NM_172621	*Clic5*	2.50	9.30	0.013
XM_139814	*LOC240038*	2.50	10.04	0.037
NM_144545	*Eif3s1*	2.50	14.83	0.001
NM_011404	*Slc7a5*	2.50	15.54	0.030
NM_178608	*Reep1*	2.49	12.21	0.002
AK079499	*AK079499*	2.49	9.71	0.034
AK033409	*Gstcd*	2.49	12.43	0.036
NM_029467	*Tcam1*	2.49	8.69	0.001
AK083849	*AK083849*	2.49	7.34	0.018
NM_001013375	*Utp18*	2.49	11.50	0.005
NM_019987	*Ick*	2.49	11.02	0.021
NAP026836-1	*NAP026836-1*	2.49	8.69	0.017
NM_011421	*Smpd1*	2.49	14.12	0.021
NM_133794	*Qars*	2.49	10.94	0.024
NM_011032	*P4hb*	2.49	18.13	0.050
NM_025546	*Rsl1d1*	2.48	9.20	0.001
AK047913	*C130021H21Rik*	2.48	9.19	0.019
NM_008209	*Mr1*	2.48	9.07	0.017
NM_024223	*Crip2*	2.48	14.88	0.016
NM_001013028	*AI597468*	2.48	15.00	0.036
NM_001024712	*LOC544988*	2.48	9.38	0.023
NM_053229	*V1rb8*	2.48	9.12	0.020
NM_026892	*Eif1b*	2.48	15.58	0.002
NM_026312	*2610029G23Rik*	2.48	6.75	0.001
NM_008799	*Pdcd2*	2.48	12.01	0.028
NM_001013792	*BC087945*	2.48	12.99	0.007
NM_009273	*Srp14*	2.48	17.50	0.001
NM_023158	*Cxcl16*	2.47	8.77	0.000
NM_031999	*Gpr137b*	2.47	13.55	0.012
NM_177474	*D19Bwg1357e*	2.47	13.06	0.003
NM_009866	*Cdh11*	2.47	13.34	0.014
NM_010880	*Ncl*	2.47	13.75	0.016
NM_001034900	*LOC545471*	2.46	8.92	0.023
NM_145984	*Prepl*	2.46	12.46	0.007
NM_152815	*Lins2*	2.46	11.19	0.022
AK028505	*Rad18*	2.46	9.28	0.029
XM_484785	*LOC433230*	2.46	10.91	0.041
NM_011281	*Rorc*	2.46	10.60	0.015
BC010717	*Jarid1a*	2.46	9.32	0.039
AK007130	*1700106N22Rik*	2.46	7.69	0.035
NM_009679	*Ap2m1*	2.45	9.76	0.009
NM_011650	*Tsn*	2.45	14.25	0.031
NM_028136	*Dhx36*	2.45	11.82	0.024
NM_020560	*Mrps31*	2.45	12.39	0.004
XM_129027	*Cep76*	2.45	9.01	0.024
NM_010072	*Dpm1*	2.45	10.24	0.032
NM_013716	*RP23-336J1.4*	2.45	9.56	0.007
NM_001024468	*Bcat1*	2.45	14.46	0.002
NM_027156	*Ddx51*	2.45	11.39	0.025
NM_144924	*Tbc1d17*	2.45	12.12	0.011
NM_009252	*Serpina3n*	2.45	9.19	0.002
NM_028314	*2700097O09Rik*	2.45	12.78	0.013
NM_022032	*Perp*	2.45	13.09	0.013

NM_018740	*Rai12*	2.44	13.88	0.014
NM_148942	*Serpinb6c*	2.44	9.97	0.039
NAP062835-1	*NAP062835-1*	2.44	10.03	0.038
NAP102122-1	*NAP102122-1*	2.44	9.04	0.007
NM_011774	*Slc30a4*	2.44	11.69	0.017
NM_133692	*Pold3*	2.44	11.00	0.021
AK085552	*Tbca*	2.44	6.79	0.001
NM_026465	*2010316F05Rik*	2.44	11.86	0.023
NM_029810	*Nt5c2*	2.44	10.05	0.016
NM_016865	*Htatip2*	2.44	11.89	0.026
NM_026452	*Coq9*	2.44	11.05	0.045
NM_021463	*Prps1*	2.44	13.61	0.006
AK081464	*AK081464*	2.44	8.71	0.025
NM_144905	*6330416G13Rik*	2.44	9.73	0.003
NM_213729	*AI842396*	2.44	8.87	0.012
NM_025646	*Crls1*	2.44	13.20	0.026
NM_019465	*Crtam*	2.44	9.73	0.034
NM_026095	*Snrpd3*	2.44	15.17	0.024
NM_146248	*Cchcr1*	2.44	9.98	0.033
AK046867	*4932438A13Rik*	2.44	8.14	0.003
BC025885	*BC025885*	2.43	14.55	0.013
NM_145505	*AI450540*	2.43	11.63	0.017
NM_138590	*D4Wsu132e*	2.43	14.25	0.029
NM_030706	*Trim2*	2.43	10.26	0.016
NM_009282	*Stag1*	2.43	9.46	0.006
NM_025671	*Ogfod2*	2.43	10.50	0.041
NM_026719	*Lmbrd1*	2.43	12.37	0.026
AK154222	*4833409A17Rik*	2.43	11.74	0.034
NM_144882	*2810022L02Rik*	2.42	11.11	0.022
BC027278	*BC027278*	2.42	16.24	0.014
DQ077804	*BC003331*	2.42	10.08	0.004
AK037574	*H2-Q1*	2.42	14.60	0.004
NM_033552	*Slc4a10*	2.42	9.23	0.000
NM_019814	*Higd1a*	2.42	10.02	0.003
BC060722	*Ibtk*	2.42	14.06	0.041
NM_172428	*2310042L06Rik*	2.42	13.70	0.001
NM_001040399	*1700108L22Rik*	2.42	11.39	0.008
AK082178	*3000002C10Rik*	2.42	16.32	0.005
NM_001025102	*2700007P21Rik*	2.42	15.19	0.010
NM_025901	*Polr3k*	2.41	9.19	0.034
AK037524	*AK037524*	2.41	9.45	0.027
BC049236	*Slc4a4*	2.41	6.89	0.014
NM_031179	*Sf3b1*	2.41	10.24	0.038
NM_010591	*Jun*	2.41	11.15	0.014
AK122556	*BC057079*	2.41	11.89	0.011
AK081044	*AK081044*	2.41	7.91	0.026
NM_009412	*Tpd52*	2.41	13.63	0.012
BC037681	*Polr2e*	2.40	16.78	0.003
NM_001004436	*Wapal*	2.40	13.39	0.027
NM_026229	*Gpr89*	2.40	8.58	0.002
AK085615	*AK085615*	2.40	7.31	0.011
NM_019466	*Dscr1*	2.40	10.21	0.027
NM_197993	*Rbm21*	2.40	13.06	0.008
NM_211355	*1110034C04Rik*	2.40	10.38	0.039
AK140255	*C330018D20Rik*	2.40	6.99	0.001
NM_010397	*H2-T22*	2.40	10.79	0.030
NM_001012309	*Ccdc55*	2.40	9.06	0.047
NM_008054	*Fyn*	2.40	9.67	0.003
AB019028	*Crsp2*	2.40	9.23	0.028

AK018344	*Vamp4*	2.40	11.76	0.037
XM_976816	*A630089N07Rik*	2.39	9.72	0.006
BC030908	*Lrp2bp*	2.39	7.86	0.045
NAP093832-001	*NAP093832-001*	2.39	11.11	0.028
NM_133216	*Xpnpep1*	2.39	10.56	0.018
NM_175112	*Rae1*	2.39	12.28	0.020
NM_023232	*Diablo*	2.39	13.43	0.028
NM_021312	*Wdr12*	2.39	10.79	0.038
NM_178119	*Centg2*	2.38	11.71	0.003
NAP027859-1	*NAP027859-1*	2.38	12.22	0.018
NM_016784	*Plrg1*	2.38	10.80	0.046
NM_001013581	*Pard3*	2.38	10.04	0.026
NM_011550	*Mlx*	2.38	10.08	0.002
AK038372	*AK038372*	2.38	9.27	0.024
NM_007670	*Cdkn2b*	2.38	11.40	0.005
NM_144515	*Zfp52*	2.38	9.45	0.043
NM_138599	*Tomm70a*	2.38	14.02	0.014
NM_033370	*Copb1*	2.37	13.38	0.030
NM_011304	*Ruvbl2*	2.37	10.96	0.001
BC004016	*Uck2*	2.37	13.80	0.012
AK134274	*Ddx27*	2.37	12.13	0.009
NM_025898	*Napa*	2.37	14.30	0.009
NM_145822	*Cd3eap*	2.37	11.16	0.033
NM_053093	*Tac4*	2.37	8.65	0.017
NM_018758	*Apba3*	2.37	13.69	0.006
NM_183027	*Ap1s3*	2.37	10.05	0.037
BC066073	*AA407452*	2.37	9.73	0.022
NM_008765	*Orc2l*	2.37	10.60	0.020
NAP092588-001	*NAP092588-001*	2.37	15.95	0.012
NM_153787	*Bclaf1*	2.37	8.98	0.022
BC006965	*BC006965*	2.37	9.45	0.015
NM_025423	*1110059E24Rik*	2.37	12.73	0.036
NM_026247	*Glt28d1*	2.36	10.19	0.020
NM_023485	*Sync*	2.36	8.98	0.003
NAP112590-1	*NAP112590-1*	2.36	11.35	0.013
NM_009533	*Xrcc5*	2.36	11.31	0.006
NM_172562	*Tada2l*	2.36	8.89	0.019
NM_027872	*1200006F02Rik*	2.36	10.78	0.003
BC036957	*Pax6*	2.36	8.98	0.000
NM_011752	*Zfp259*	2.36	10.43	0.019
NM_015799	*Trfr2*	2.36	10.61	0.028
AK172893	*Ube4a*	2.36	10.49	0.005
AK051891	*D230015P20Rik*	2.35	6.77	0.011
NM_027519	*6330406I15Rik*	2.35	9.79	0.038
NM_146978	*Olfr1258*	2.35	8.84	0.017
AV132191	*Ptcd1*	2.35	12.76	0.014
NM_013899	*Timm10*	2.35	14.21	0.023
NM_013745	*Nufip1*	2.35	13.62	0.007
NM_172274	*5730509K17Rik*	2.35	8.92	0.045
NM_175428	*Zfp295*	2.35	8.12	0.005
AK037098	*Cast*	2.35	9.84	0.042
NM_133991	*Ftsj1*	2.35	12.22	0.006
AK077389	*Golga3*	2.35	13.32	0.005
NM_198619	*MGC67181*	2.35	8.09	0.000
NM_145944	*Ccdc25*	2.35	10.51	0.020
BC079668	*Whsc1*	2.35	9.65	0.014
NM_178935	*4932441K18Rik*	2.35	10.59	0.015
NM_001008427	*LOC434179*	2.34	16.81	0.033
NM_026048	*2810452K22Rik*	2.34	10.90	0.027

NM_177214	*Ascc3l1*	2.34	13.05	0.007
NM_175391	*2210421G13Rik*	2.34	9.10	0.008
NM_011255	*Rbp4*	2.34	16.08	0.018
NM_183146	*A530054K11Rik*	2.34	10.51	0.007
NM_177096	*B430203M17Rik*	2.34	7.85	0.008
NM_011324	*Scnn1a*	2.34	10.29	0.017
NM_018791	*Zfp108*	2.33	10.32	0.008
NM_001001180	*BC066028*	2.33	9.85	0.011
M31690	*Ass1*	2.33	10.67	0.003
NM_026409	*Ddx55*	2.33	9.91	0.021
NM_009359	*Tex9*	2.33	9.09	0.019
AK032000	*9930017N22Rik*	2.33	8.78	0.000
AK054054	*AK054054*	2.33	9.75	0.009
AK035943	*AK035943*	2.33	9.94	0.012
XM_001006141	*Jrkl*	2.33	8.94	0.029
NM_153156	*Stoml3*	2.33	8.90	0.006
NAP094515-001	*NAP094515-001*	2.33	11.15	0.018
NM_013556	*Hprt1*	2.32	16.13	0.002
AK047788	*C030040A22Rik*	2.32	6.63	0.002
NM_013887	*Opn4*	2.32	10.00	0.026
XM_001001527	*LOC630499*	2.32	14.31	0.012
NM_178672	*Scfd2*	2.32	8.89	0.014
NM_025535	*Sar1b*	2.32	14.74	0.013
BC094565	*Fbxo10*	2.32	12.02	0.022
NM_012000	*Cln8*	2.32	9.27	0.012
AK042179	*AK042179*	2.32	7.38	0.005
NM_009593	*Abcg1*	2.32	11.16	0.028
NM_173383	*Dnd1*	2.32	10.12	0.050
AK087532	*E130314M14Rik*	2.32	9.80	0.045
NM_007608	*Car5a*	2.32	8.41	0.036
BC082558	*Tbccd1*	2.31	10.44	0.033
NM_016773	*Nucb2*	2.31	13.63	0.040
NM_010918	*Nktr*	2.31	13.56	0.008
NM_145528	*D2Ertd391e*	2.31	11.31	0.024
NM_145591	*BC003267*	2.31	9.31	0.024
AK083758	*AK083758*	2.31	7.08	0.026
AK158756	*Rasgef1a*	2.31	9.05	0.004
NM_172693	*Galnt12*	2.31	10.23	0.036
XM_128374	*Zfp294*	2.31	9.43	0.022
BC094221	*BC094221*	2.31	11.43	0.020
BC034085	*Rmnd5a*	2.31	8.60	0.011
NM_177323	*Rint1*	2.31	12.20	0.027
BC048176	*E430034L04Rik*	2.31	13.37	0.019
NM_133898	*B230342M21Rik*	2.30	9.62	0.032
NM_172295	*BC037703*	2.30	9.27	0.000
NM_025951	*Pi4k2b*	2.30	10.27	0.026
NM_175360	*Obfc1*	2.30	9.42	0.021
NM_177186	*A530082C11Rik*	2.30	13.39	0.005
NM_019493	*Btg4*	2.30	9.35	0.047
BC080756	*H2-K1*	2.30	15.59	0.002
NM_017476	*Akap8l*	2.30	10.33	0.015
AK082305	*Pex1*	2.30	9.47	0.011
XM_888686	*Atf6*	2.30	11.41	0.003
NM_027299	*Degs2*	2.30	9.60	0.002
NM_172627	*Pggt1b*	2.30	9.55	0.007
NM_029607	*2310003C23Rik*	2.30	9.76	0.046
AK041120	*Rfx3*	2.30	8.08	0.032
NM_028599	*Wdr75*	2.29	10.68	0.011
NM_026584	*Gtf2e2*	2.29	14.61	0.025

NM_011342	*Sec22b*	2.29	14.44	0.031
BC030415	*Col20a1*	2.29	9.92	0.012
AF037454	*Itch*	2.29	13.92	0.020
BC014761	*Lrrfip2*	2.29	10.02	0.016
AK088830	*Braf*	2.29	11.23	0.040
NAP030437-1	*NAP030437-1*	2.29	10.94	0.042
AK033612	*Yipf6*	2.29	7.48	0.001
AK011292	*2610002J23Rik*	2.29	10.77	0.048
AK037684	*AK037684*	2.29	8.22	0.010
XM_981786	*Gtl2*	2.29	11.29	0.034
NM_026792	*Agpat5*	2.29	11.97	0.005
NM_145975	*Ddx46*	2.29	12.38	0.003
AK085795	*AK085795*	2.28	7.49	0.008
NM_022884	*Bhmt2*	2.28	7.63	0.016
NM_010259	*Gbp1*	2.28	8.56	0.042
XM_001006017	*LOC382161*	2.28	8.48	0.002
NAP108362-1	*NAP108362-1*	2.28	9.35	0.010
NM_009775	*Tspo*	2.28	12.59	0.036
NM_011233	*Rad17*	2.28	11.36	0.023
NM_027212	*Thrap6*	2.28	13.12	0.010
NM_009864	*Cdh1*	2.28	8.04	0.002
NM_025845	*Prpf38b*	2.27	9.32	0.038
NM_007647	*Entpd5*	2.27	11.89	0.034
NM_145552	*Gnl2*	2.27	12.71	0.032
AK018564	*9030622M22Rik*	2.27	7.64	0.014
NM_023153	*0610040D20Rik*	2.27	15.35	0.011
NM_027649	*Spats1*	2.27	8.37	0.008
NM_145479	*Klhl22*	2.27	12.80	0.011
NAP057123-1	*NAP057123-1*	2.27	9.28	0.037
NM_025840	*Bzw2*	2.27	8.83	0.015
NM_007661	*Cdc2l1*	2.27	11.90	0.035
AK013141	*2810423A18Rik*	2.26	14.86	0.003
NM_019787	*Sec23b*	2.26	14.07	0.001
NM_010893	*Neu1*	2.26	10.75	0.002
AB010352	*Mszf33*	2.26	10.16	0.034
NM_025344	*Eif3s5*	2.26	13.61	0.029
NM_011480	*Srebf1*	2.26	14.42	0.005
NM_023503	*Ing2*	2.26	9.48	0.033
NM_144558	*Bivm*	2.26	8.53	0.006
AK081256	*9030617O03Rik*	2.26	9.79	0.025
NM_178357	*Tieg3*	2.25	11.14	0.020
NM_027000	*Gtpbp4*	2.25	9.86	0.024
NM_026400	*ERdj3*	2.25	16.47	0.006
NM_177235	*B230209C24Rik*	2.25	9.72	0.032
NM_027561	*4632415L05Rik*	2.25	9.39	0.017
NM_028431	*Pmpcb*	2.25	13.69	0.014
NM_133750	*3110048E14Rik*	2.25	9.31	0.025
NM_138584	*Spg21*	2.25	10.38	0.003
BC052637	*Mtap7*	2.25	8.97	0.012
NM_138680	*Luc7l2*	2.25	14.19	0.040
NM_025859	*Arl1*	2.25	15.39	0.007
NM_019695	*Pard6a*	2.25	11.11	0.033
NM_172511	*Abhd10*	2.24	9.57	0.027
AK137302	*AK137302*	2.24	9.43	0.020
XM_983620	*6720475J19Rik*	2.24	10.47	0.014
NM_172697	*Prpf38a*	2.24	13.85	0.001
NM_172276	*Sfrs8*	2.24	13.41	0.048
NM_145397	*BC002059*	2.24	9.28	0.023
AK028852	*D030022P07Rik*	2.24	10.60	0.015

NM_133941	*Dhx32*	2.24	14.49	0.014
NM_144521	*1110031B06Rik*	2.24	13.76	0.010
BC043690	*Abhd13*	2.24	10.32	0.042
AK020820	*Ttyh1*	2.24	8.93	0.021
AK003999	*Gtf2f2*	2.24	12.38	0.010
XM_132143	*Srp72*	2.24	14.84	0.015
NAP027924-1	*NAP027924-1*	2.23	12.21	0.009
AI427529	*AI427529*	2.23	8.85	0.014
BC094938	*MGC107533*	2.23	8.45	0.001
NM_028862	*3732413I11Rik*	2.23	12.64	0.035
AK165240	*Slc16a10*	2.23	9.96	0.015
NM_177358	*A630033E08Rik*	2.23	12.05	0.007
NM_008049	*Ftl2*	2.23	16.59	0.028
NM_028811	*Elp3*	2.23	13.53	0.023
NM_153134	*Irgq*	2.23	10.24	0.029
XM_994427	*Sh3d19*	2.23	8.51	0.025
NM_012055	*Asns*	2.23	16.44	0.017
AK017313	*5430416B10Rik*	2.23	8.49	0.003
NM_146249	*BC031441*	2.23	11.30	0.028
BC069869	*Wdr36*	2.23	10.46	0.025
NM_019442	*Stk19*	2.22	11.66	0.045
NM_026171	*Nvl*	2.22	8.75	0.015
NM_177239	*Mysm1*	2.22	10.63	0.036
NM_146078	*Ubr2*	2.22	8.65	0.004
NM_008715	*Ints6*	2.22	11.14	0.034
AK134636	*2610005L07Rik*	2.22	11.49	0.006
NAP042167-1	*NAP042167-1*	2.22	8.17	0.004
NM_009051	*Rex2*	2.22	9.32	0.008
NM_203507	*Rwdd4a*	2.22	13.77	0.005
AK042419	*AK042419*	2.22	7.79	0.006
NM_145943	*BC031781*	2.22	12.66	0.016
AK018439	*8430425A16Rik*	2.22	8.79	0.006
BC023155	*Dnahc1*	2.22	9.69	0.049
NM_007483	*Rhob*	2.22	10.15	0.009
NM_133740	*Prmt3*	2.22	9.83	0.044
NM_009187	*Cox7a2l*	2.22	12.67	0.015
XM_141392	*Kif16b*	2.22	9.57	0.011
AK018277	*6430500C12Rik*	2.21	7.71	0.044
AK029235	*A930040G15Rik*	2.21	8.85	0.045
AK040392	*AK040392*	2.21	8.19	0.020
AK037158	*Osbpl8*	2.21	7.70	0.050
AK161694	*AW495713*	2.21	10.98	0.048
XM_982400	*2610207F23Rik*	2.21	9.42	0.002
NM_028097	*Tmem68*	2.21	11.43	0.029
NAP058254-1	*NAP058254-1*	2.21	9.46	0.028
AW490100	*AW490100*	2.21	7.10	0.012
NM_019961	*Pex3*	2.21	9.05	0.014
NM_010749	*M6pr*	2.21	14.31	0.012
NM_145608	*BC021891*	2.21	11.29	0.012
AK122222	*Appbp2*	2.21	12.20	0.032
NM_009471	*Umps*	2.20	9.77	0.032
NM_028410	*Prkrir*	2.20	8.90	0.002
AK048435	*C130065N10Rik*	2.20	10.21	0.006
NM_021372	*Sertad2*	2.20	9.97	0.034
AK129110	*BC030440*	2.20	10.00	0.035
XM_985259	*LOC626391*	2.20	9.37	0.026
AK052911	*Trim63*	2.20	9.17	0.009
NM_019393	*Exosc9*	2.20	11.94	0.017
NM_153287	*Axud1*	2.19	11.65	0.010

NM_145469	*Npal2*	2.19	10.66	0.029
NM_177008	*4932435O22Rik*	2.19	9.33	0.021
NM_199062	*BC024063*	2.19	9.12	0.043
NM_178065	*1110018G07Rik*	2.19	13.04	0.001
XM_145466	*LOC243881*	2.19	8.31	0.015
NM_007918	*Eif4ebp1*	2.19	16.77	0.026
AK034401	*Sap130*	2.19	9.37	0.002
NM_009549	*Zfp185*	2.19	12.00	0.018
BC038907	*BC038907*	2.19	8.79	0.032
NM_145511	*BC003331*	2.19	9.44	0.013
NM_001033298	*Gm114*	2.19	13.10	0.027
AK034915	*AK034915*	2.19	9.16	0.012
NM_029281	*MGC29393*	2.19	9.73	0.037
NM_175933	*Pex5*	2.19	11.39	0.042
AK016844	*4933417G07Rik*	2.19	10.46	0.038
NM_009000	*Rab24*	2.19	13.93	0.010
AK039166	*5730407K14Rik*	2.19	7.73	0.028
NM_134172	*V1rc17*	2.18	7.64	0.031
NAP062064-1	*NAP062064-1*	2.18	12.05	0.018
NM_033074	*Tars*	2.18	16.67	0.010
NM_012048	*Polk*	2.18	12.62	0.049
NM_021526	*Psmd14*	2.18	15.00	0.022
NAP101988-1	*NAP101988-1*	2.18	15.15	0.045
NM_175352	*AA407659*	2.18	9.69	0.032
NM_026148	*Lims1*	2.18	8.97	0.035
NM_028110	*Dennd2d*	2.18	9.44	0.003
AK009316	*Ski*	2.18	11.52	0.046
CB588406	*CB588406*	2.18	10.28	0.050
NM_023685	*Zfp306*	2.18	9.36	0.038
NM_019719	*Stub1*	2.18	14.74	0.021
NAP036221-1	*NAP036221-1*	2.18	11.50	0.041
NM_010361	*Gstt2*	2.18	10.61	0.010
NM_010726	*Phyh*	2.18	12.37	0.050
NM_028478	*Rassf6*	2.18	8.89	0.003
NM_178388	*4930425N13Rik*	2.17	8.02	0.001
NM_018861	*Slc1a4*	2.17	12.27	0.004
AK037691	*Tmem16f*	2.17	7.83	0.035
AK035387	*9530028C05*	2.17	16.08	0.038
NM_026665	*Cep57*	2.17	10.84	0.012
NM_027011	*Krt5*	2.17	10.38	0.043
NM_172567	*Mettl2*	2.17	9.84	0.009
BC024728	*Slco2a1*	2.17	13.74	0.018
AK133251	*Usp48*	2.17	9.94	0.010
DQ355288	*Vwf*	2.17	10.56	0.020
NM_019966	*Mlycd*	2.17	11.49	0.049
NM_025303	*Stau2*	2.17	12.60	0.044
XM_992169	*D10Ertd438e*	2.16	11.54	0.036
NM_025656	*Sip1*	2.16	11.17	0.040
NM_031249	*Cstf2t*	2.16	12.66	0.002
NM_026959	*Stx18*	2.16	14.05	0.011
BC020137	*Mitd1*	2.16	8.70	0.004
NM_026041	*2810430M08Rik*	2.16	14.24	0.004
NM_025862	*Acad8*	2.16	10.70	0.029
NM_009572	*Zhx1*	2.16	9.63	0.021
NM_024242	*Riok1*	2.16	8.62	0.010
NM_011014	*Oprs1*	2.16	10.34	0.034
NM_016903	*Esd*	2.16	16.74	0.048
AK035750	*Pdcd6ip*	2.16	10.60	0.009
NM_027927	*Ints12*	2.16	10.51	0.039

NM_009498	*Vamp3*	2.16	9.68	0.047
BC033455	*0610007L01Rik*	2.16	13.26	0.024
NM_007697	*Chl1*	2.16	9.07	0.001
NAP038076-1	*NAP038076-1*	2.16	9.37	0.026
BC011329	*Pgd*	2.15	10.32	0.043
NM_009706	*Arhgap5*	2.15	10.87	0.042
NM_025418	*1110059P08Rik*	2.15	12.47	0.012
NM_001004185	*Whdc1*	2.15	10.86	0.043
NAP021083-001	*NAP021083-001*	2.15	9.29	0.005
U64446	*U64446*	2.15	9.14	0.009
NM_026067	*Thex1*	2.15	9.04	0.009
AK052358	*D330037H05Rik*	2.15	10.62	0.043
NM_023668	*Ndel1*	2.15	14.49	0.014
AK037264	*AK037264*	2.15	7.60	0.025
AK076333	*Ganc*	2.15	8.31	0.002
NM_153424	*Nphp4*	2.15	10.06	0.045
AK053084	*AK053084*	2.15	8.48	0.009
NM_023348	*Snap29*	2.15	9.16	0.020
NM_010225	*Foxf2*	2.15	9.14	0.013
NM_152807	*3110023B02Rik*	2.15	10.10	0.011
CO796036	*CO796036*	2.15	11.15	0.005
NM_021713	*Myg1*	2.15	11.53	0.017
AK078133	*6230429P13Rik*	2.14	12.63	0.023
NM_172302	*5730453I16Rik*	2.14	12.21	0.031
AK133661	*Lrrfip1*	2.14	14.17	0.004
NM_023418	*Pgam1*	2.14	17.64	0.014
NAP035212-1	*NAP035212-1*	2.14	9.12	0.018
NM_001013808	*LOC433801*	2.14	8.99	0.045
NM_016897	*Timm23*	2.13	15.26	0.008
NM_172823	*Lmln*	2.13	8.67	0.001
NM_146441	*Olfr933*	2.13	9.56	0.034
NM_027740	*Wdr51b*	2.13	8.58	0.035
NM_080562	*Ubox5*	2.13	10.98	0.048
NM_177124	*Tnrc6b*	2.13	8.88	0.048
AK009669	*Tmem158*	2.13	15.07	0.006
NM_011415	*Snai2*	2.13	14.79	0.028
NM_134226	*V1rj2*	2.13	9.60	0.040
NM_028059	*Zfp654*	2.13	8.62	0.008
NM_025982	*Tspan31*	2.13	15.54	0.002
NM_026102	*Daam1*	2.13	12.15	0.041
NM_021529	*4930511A21Rik*	2.13	11.93	0.033
XM_888349	*LOC433882*	2.13	8.66	0.018
NM_011596	*Atp6v0a2*	2.12	12.11	0.006
AK048881	*AK048881*	2.12	9.61	0.033
BC043710	*Dopey1*	2.12	9.31	0.010
NM_013642	*Dusp1*	2.12	12.58	0.041
NM_175238	*Rif1*	2.12	9.50	0.044
AK135556	*Cdc26*	2.12	7.77	0.004
XM_917294	*LOC640374*	2.12	16.86	0.011
NM_001008705	*Bud31*	2.12	14.06	0.014
NM_025465	*1810029B16Rik*	2.12	10.27	0.041
AK002861	*0610040B10Rik*	2.12	8.44	0.028
NM_177171	*D930036F22Rik*	2.12	12.89	0.019
NM_026425	*Nat5*	2.12	13.73	0.042
NM_172637	*Hectd2*	2.12	8.27	0.027
BI152977	*BI152977*	2.12	11.18	0.033
XM_148244	*Golgb1*	2.12	9.25	0.007
NM_021421	*Angel2*	2.12	12.90	0.005
NM_153407	*BC035295*	2.12	8.59	0.020

NM_011694	*Vdac1*	2.12	14.38	0.035
NM_027314	*38412*	2.12	14.60	0.046
NM_053228	*V1rb7*	2.12	8.72	0.027
NM_146144	*Usp1*	2.11	13.96	0.044
NM_026791	*Fbxw9*	2.11	9.40	0.032
AK005231	*1500012F01Rik*	2.11	13.41	0.039
NM_010786	*Mdm2*	2.11	12.22	0.030
NM_008377	*Lrig1*	2.11	9.67	0.045
NM_172751	*Arhgef10*	2.11	9.38	0.032
NM_172647	*F11r*	2.11	12.84	0.006
NM_199472	*MGC68323*	2.11	17.58	0.003
AK144593	*Ppapdc1*	2.11	13.80	0.033
NM_009005	*Rab7*	2.10	14.33	0.021
NM_029271	*Mrpl32*	2.10	12.44	0.036
NM_175151	*Tatdn1*	2.10	11.95	0.003
NM_028226	*Rbm12b*	2.10	9.21	0.012
XM_977926	*Ggps1*	2.10	11.36	0.020
NM_177663	*Isg20l2*	2.10	13.29	0.045
AK047044	*Phf20l1*	2.10	9.00	0.016
XM_132005	*4933407H18Rik*	2.09	9.75	0.008
NM_178702	*D930005D10Rik*	2.09	8.55	0.032
NM_199476	*Rrm2b*	2.09	9.60	0.007
NM_145743	*Lace1*	2.09	9.12	0.030
NM_178875	*8430426H19Rik*	2.09	9.08	0.008
NM_153395	*Mon2*	2.09	13.99	0.020
NM_177911	*Tgm4*	2.09	9.03	0.006
NM_173016	*AI427515*	2.09	9.63	0.005
NM_023363	*2810426N06Rik*	2.09	9.10	0.004
NM_026352	*Ppid*	2.09	15.08	0.031
NM_025323	*0610009D07Rik*	2.09	16.05	0.005
XM_978218	*1810013L24Rik*	2.09	8.42	0.040
BC050849	*Rbbp8*	2.09	12.27	0.010
AK003545	*St13*	2.09	11.04	0.009
NM_172396	*9130011J15Rik*	2.08	14.26	0.008
NAP112575-1	*NAP112575-1*	2.08	16.86	0.010
AK008216	*D130073L02Rik*	2.08	12.78	0.020
NM_011722	*Dctn6*	2.08	13.22	0.011
NM_009721	*Atp1b1*	2.08	10.63	0.012
NM_008722	*Npm1*	2.08	16.39	0.028
NM_027998	*Cldn23*	2.08	8.62	0.045
NM_016918	*Nudt5*	2.08	11.62	0.049
NM_010832	*Msl31*	2.08	13.29	0.023
NM_010492	*Ica1*	2.08	12.13	0.050
AK040981	*AK040981*	2.08	7.65	0.045
AK082583	*AK082583*	2.08	9.69	0.008
NAP033617-1	*NAP033617-1*	2.08	9.10	0.007
AK088165	*D5Ertd135e*	2.08	9.19	0.019
BC072573	*1300001I01Rik*	2.08	12.92	0.017
NM_172943	*Alkbh5*	2.08	15.16	0.019
BC066818	*Polr3g*	2.07	8.73	0.049
NM_026485	*Trabd*	2.07	13.75	0.021
NAP060490-1	*NAP060490-1*	2.07	13.20	0.032
NM_019639	*Ubc*	2.07	14.92	0.022
NM_028803	*Gbe1*	2.07	14.28	0.041
NM_026301	*Rnf125*	2.07	9.83	0.006
NM_008828	*Pgk1*	2.07	17.64	0.010
NM_146036	*Ahsa1*	2.07	16.21	0.001
NM_183165	*BC027061*	2.07	11.54	0.020
NM_026267	*Necap1*	2.07	13.13	0.030

AK089967	*Rg9mtd2*	2.07	12.37	0.024
NM_026539	*Chd1l*	2.07	9.59	0.004
NM_030066	*Armcx1*	2.07	13.42	0.010
AK168488	*Rbm35b*	2.07	9.39	0.002
NM_175260	*Myh10*	2.07	11.21	0.038
NM_172772	*B230380D07Rik*	2.07	10.70	0.042
NM_197998	*2210021J22Rik*	2.07	12.85	0.017
NM_025485	*Mrps22*	2.07	12.77	0.026
NM_013499	*Crry*	2.06	8.42	0.010
NM_175265	*6720463M24Rik*	2.06	9.44	0.003
NM_015797	*Fbxo6b*	2.06	9.63	0.005
BC020156	*Trim41*	2.06	12.01	0.041
NM_024448	*Rab12*	2.06	9.94	0.044
NM_025590	*Acot11*	2.06	9.64	0.009
XM_132499	*Lmtk2*	2.06	10.14	0.038
NM_080556	*Tm9sf2*	2.06	15.19	0.021
XM_982560	*LOC333830*	2.06	16.19	0.013
NM_147222	*Rdhs*	2.06	9.24	0.032
NM_025692	*Ube1dc1*	2.06	15.24	0.002
NM_172480	*Mtrr*	2.06	9.03	0.002
AK028719	*Copz1*	2.05	8.65	0.008
NM_007691	*Chek1*	2.05	10.32	0.005
NM_008009	*Fgfbp1*	2.05	9.38	0.049
NM_001002771	*A830005F24Rik*	2.05	9.39	0.036
NM_027350	*Nars*	2.05	13.17	0.016
NM_025296	*Wdr39*	2.05	13.44	0.005
AK085487	*AK085487*	2.05	8.33	0.001
NAP071007-1	*NAP071007-1*	2.05	8.12	0.039
NM_011400	*Slc2a1*	2.05	16.46	0.014
NM_016776	*Mybbp1a*	2.05	9.45	0.002
NM_175287	*A430005L14Rik*	2.05	15.90	0.041
BC060121	*Phf20*	2.05	9.44	0.028
NM_022432	*Sirt2*	2.05	11.53	0.020
NM_145415	*AA408296*	2.05	8.99	0.006
XM_001000749	*LOC668299*	2.05	18.00	0.001
NM_199016	*Enpp4*	2.05	10.09	0.005
NM_007952	*Erp57*	2.05	17.25	0.028
NM_172625	*D030070L09Rik*	2.05	10.68	0.028
NM_026509	*2310039E09Rik*	2.05	11.09	0.048
NM_011696	*Vdac3*	2.05	16.37	0.004
AK019726	*4930539E08Rik*	2.05	9.58	0.049
NM_010247	*Xrcc6*	2.04	9.09	0.018
AK079745	*AI449175*	2.04	8.00	0.017
XM_140451	*Lama3*	2.04	9.00	0.003
NM_009967	*Crygs*	2.04	9.03	0.018
NM_030180	*Usp54*	2.04	12.49	0.044
NM_010709	*Lgtn*	2.04	10.40	0.023
NM_145995	*2700050L05Rik*	2.04	11.60	0.033
NM_181853	*Trim66*	2.04	9.03	0.012
NM_026768	*Mrps18a*	2.04	14.27	0.005
NM_175558	*Zfp446*	2.04	8.64	0.003
NM_028284	*Bbs5*	2.03	8.93	0.013
NM_013736	*Tceb3*	2.03	10.02	0.019
NM_175213	*4930486G11Rik*	2.03	8.72	0.005
NM_026043	*Rnpc3*	2.03	11.55	0.012
AK157817	*Spred2*	2.03	10.53	0.030
NM_013601	*Msx2*	2.03	9.79	0.030
BF682133	*BF682133*	2.03	8.91	0.002
NM_010467	*Hoxd1*	2.03	9.35	0.010

NM_009262	*Spock1*	2.03	8.20	0.015
NM_144796	*Susd4*	2.03	8.96	0.006
AK014419	*Nup205*	2.03	8.96	0.013
NM_024270	*Stard3nl*	2.03	11.12	0.021
AK006127	*1700019L03Rik*	2.02	9.14	0.008
NM_022332	*St7*	2.02	13.04	0.029
NM_011315	*Saa3*	2.02	7.57	0.010
NM_028121	*Adpgk*	2.02	12.55	0.012
NM_009201	*Slc1a5*	2.02	16.36	0.007
XM_978638	*LOC384039*	2.02	10.50	0.016
NM_001009947	*Dock11*	2.02	8.97	0.046
NM_001024713	*2610042L04Rik*	2.02	9.15	0.020
NM_015827	*Copb2*	2.02	16.36	0.005
NM_177310	*E430012M05Rik*	2.02	9.35	0.008
XM_126361	*5730455P16Rik*	2.02	8.48	0.032
NM_133749	*2900064A13Rik*	2.02	15.78	0.029
AK048657	*AK048657*	2.02	8.42	0.009
NM_026654	*Toe1*	2.02	12.38	0.022
NM_176828	*C030006K11Rik*	2.02	10.34	0.020
NM_022883	*Lpin3*	2.02	11.14	0.013
NM_011573	*Tex264*	2.02	14.44	0.014
NM_013681	*Syn2*	2.02	9.16	0.016
NM_029128	*Qtrtd1*	2.02	10.39	0.027
NM_178648	*Ubxd6*	2.01	8.90	0.034
AK048052	*AK048052*	2.01	9.56	0.009
NM_008224	*Hcfc1*	2.01	10.16	0.029
NM_018814	*Pcnx*	2.01	9.96	0.037
NM_026528	*2700060E02Rik*	2.01	15.26	0.013
NM_019912	*Ube2d2*	2.01	12.16	0.025
BC018196	*Alkbh3*	2.01	13.64	0.023
NM_053173	*Kifc1*	2.01	9.32	0.007
NM_133808	*Hdlbp*	2.01	8.75	0.004
NM_001033601	*C130038G02Rik*	2.01	9.49	0.047
NM_009510	*Vil2*	2.01	13.46	0.021
XM_916118	*Tlr5*	2.01	9.64	0.005
AK035983	*AK035983*	2.01	8.71	0.018
AK044044	*1700093C20Rik*	2.01	7.29	0.016
NM_172601	*Rab2b*	2.00	13.08	0.023
NM_028731	*D12Ertd551e*	2.00	8.64	0.030
NM_176845	*Ddhd1*	2.00	9.40	0.021
NM_145484	*BC021442*	2.00	9.54	0.018
NM_026529	*2700062C07Rik*	2.00	11.64	0.028
NM_027346	*Ccdc44*	2.00	9.82	0.035
NM_177572	*BC057371*	2.00	9.20	0.028
NM_011176	*St14*	2.00	10.81	0.044
AK031553	*Sec24a*	2.00	9.43	0.007
XM_917083	*Syne2*	2.00	9.19	0.019
NM_007656	*Cd82*	2.00	12.68	0.020
NM_019713	*Rassf1*	2.00	13.76	0.009
AK038374	*AK038374*	2.00	9.03	0.006
AK122521	*Plce1*	2.00	7.76	0.038
NM_145078	*2610305D13Rik*	2.00	8.87	0.025
NM_009296	*Supt4h1*	2.00	15.83	0.020
NM_207202	*Ccdc120*	2.00	9.08	0.014
NM_027420	*2610034B18Rik*	-2.00	10.20	0.012
AK147270	*1700020I14Rik*	-2.00	13.69	0.006
AK052696	*AK052696*	-2.00	11.77	0.027
NM_198615	*Rkhd1*	-2.00	9.99	0.014
NM_008518	*Ltb*	-2.00	10.45	0.045

AK131163	*A630054L15Rik*	-2.00	12.42	0.044
NM_025849	*3110001D03Rik*	-2.00	15.66	0.026
NM_013891	*Spdef*	-2.00	10.27	0.049
NM_146689	*Olfr1459*	-2.00	10.16	0.014
AV152162	*AV152162*	-2.00	9.78	0.009
AK083451	*Rab14*	-2.00	12.99	0.033
NM_009573	*Zic1*	-2.00	9.34	0.044
NM_175437	*A530088I07Rik*	-2.00	10.16	0.009
NM_199222	*Lman1l*	-2.00	10.01	0.003
NM_199470	*Cdh24*	-2.00	10.45	0.014
NM_010049	*Dhfr*	-2.00	10.33	0.032
NM_177033	*A930041G11Rik*	-2.00	9.46	0.006
NM_029101	*1110014J01Rik*	-2.00	11.79	0.006
AK036419	*F730031O20Rik*	-2.01	10.93	0.013
AK006048	*1700016M24Rik*	-2.01	9.85	0.002
NM_078484	*Slc35a2*	-2.01	13.04	0.012
NM_194446	*Cdk10*	-2.01	13.57	0.037
AK083757	*2610028E06Rik*	-2.01	9.48	0.028
NM_198894	*Abr*	-2.01	10.22	0.013
NM_153523	*Tcstv3*	-2.01	10.52	0.014
NM_001039578	*B130050I23Rik*	-2.01	11.03	0.029
NM_010903	*Nfe2l3*	-2.01	12.08	0.016
NM_130861	*Slco1a5*	-2.01	9.80	0.009
NM_177605	*Pdzd7*	-2.01	10.23	0.024
AK029371	*Ablim1*	-2.02	11.09	0.008
NM_146670	*Olfr815*	-2.02	9.11	0.026
NM_176846	*Exph5*	-2.02	9.81	0.003
NM_146807	*Olfr136*	-2.02	10.01	0.011
NM_194059	*Nanos3*	-2.02	10.10	0.006
NM_008519	*Ltb4r1*	-2.02	10.13	0.019
X61497	*X61497*	-2.02	10.36	0.032
NM_010283	*Ggta1*	-2.02	10.73	0.012
NM_009055	*Rfx1*	-2.02	10.49	0.017
NM_213727	*9430069J07Rik*	-2.02	10.08	0.008
NM_007641	*Ms4a1*	-2.02	9.83	0.036
NM_053104	*Rbm9*	-2.02	15.83	0.016
NM_146985	*Olfr1270*	-2.02	9.88	0.014
NM_175511	*A130092J06Rik*	-2.03	9.33	0.006
AK020361	*9230119C12Rik*	-2.03	10.12	0.013
AK006745	*1700049J03Rik*	-2.03	11.50	0.009
NM_024267	*Ipo4*	-2.03	10.71	0.017
NM_029850	*Bcl7a*	-2.03	11.89	0.024
NM_011938	*Gprk6*	-2.03	9.85	0.005
NM_027189	*Gemin7*	-2.03	15.45	0.048
NM_019544	*Msgn1*	-2.03	10.39	0.004
AK053224	*9530036O11Rik*	-2.03	12.70	0.032
NM_177158	*5830482F20Rik*	-2.03	10.42	0.013
XM_001005255	*LOC673307*	-2.03	9.76	0.006
AK154853	*2610029I01Rik*	-2.03	11.17	0.018
NM_013914	*Snai3*	-2.03	10.01	0.014
NM_011711	*Fmnl3*	-2.03	14.02	0.014
NM_008738	*Nrtn*	-2.03	10.89	0.018
NM_016868	*Hif3a*	-2.04	9.51	0.007
BB161836	*BB161836*	-2.04	7.98	0.020
NM_010206	*Fgfr1*	-2.04	10.20	0.010
AK051581	*AK051581*	-2.04	8.30	0.045
AK030305	*Col4a3bp*	-2.04	11.14	0.050
NM_177657	*D630003M21Rik*	-2.04	10.17	0.017
NM_008228	*Hdac1*	-2.04	15.36	0.030

NAP092699-001	*NAP092699-001*	-2.04	12.06	0.026
NM_008427	*Kcnj4*	-2.04	10.11	0.004
NM_025427	*1190002H23Rik*	-2.04	15.42	0.035
NM_016922	*Gal3st1*	-2.04	10.89	0.019
NM_181732	*2610208M17Rik*	-2.04	11.57	0.006
NM_011286	*Rph3a*	-2.04	9.90	0.008
AK054415	*AK054415*	-2.04	9.01	0.042
NM_028937	*Sohlh2*	-2.04	10.56	0.026
CO038890	*AW552889*	-2.04	9.97	0.016
NM_009313	*Tacr1*	-2.04	9.72	0.006
NM_026012	*Nradd*	-2.04	11.73	0.026
NAP053020-1	*NAP053020-1*	-2.05	10.10	0.003
NM_178762	*A930025D01Rik*	-2.05	10.65	0.035
NM_001005425	*Gm1008*	-2.05	10.71	0.006
NM_199058	*Gpr6*	-2.05	9.96	0.009
AF346502	*Fry*	-2.05	9.85	0.018
AK122541	*Zfp451*	-2.05	10.29	0.032
AK005811	*1700010B08Rik*	-2.05	10.34	0.014
NM_019931	*Kcnd3*	-2.05	9.92	0.011
NM_028487	*Gpbp1*	-2.05	10.51	0.003
NM_001013022	*2010001J22Rik*	-2.05	10.52	0.024
NM_133960	*Ces6*	-2.06	9.99	0.024
NM_001033245	*Hk3*	-2.06	10.88	0.003
NM_008595	*Mfng*	-2.06	9.92	0.023
NM_181585	*Pik3r3*	-2.06	10.17	0.035
NM_133192	*Npffr2*	-2.06	8.99	0.007
NM_010781	*Mcpt6*	-2.06	10.33	0.007
NM_011106	*Pkig*	-2.06	14.05	0.018
NM_025493	*1700018B24Rik*	-2.06	9.46	0.012
NM_019934	*Sec1*	-2.06	10.33	0.013
NM_008914	*Ppp3cb*	-2.06	13.10	0.006
NM_028308	*2700078K21Rik*	-2.06	14.26	0.020
S63763	*Ptpn6*	-2.06	9.89	0.018
NM_011149	*Ppib*	-2.06	13.90	0.044
NM_007717	*Cmah*	-2.06	9.79	0.008
NM_022657	*Fgf23*	-2.07	10.21	0.011
NM_011832	*Insrr*	-2.07	10.16	0.007
NM_016874	*Deaf1*	-2.07	10.54	0.008
NM_007431	*Akp2*	-2.07	17.07	0.038
NM_080640	*Baalc*	-2.07	10.35	0.004
NAP050610-1	*NAP050610-1*	-2.07	10.75	0.039
NM_146813	*Olfr651*	-2.07	10.47	0.028
NM_145851	*Cables2*	-2.07	10.02	0.019
NM_026438	*Ppa1*	-2.07	16.51	0.031
NM_198640	*AI324046*	-2.07	10.57	0.002
NM_018741	*Igfbpl1*	-2.07	8.90	0.004
NM_001008700	*Il4ra*	-2.07	10.37	0.006
NM_146697	*Olfr1442*	-2.07	9.34	0.032
NM_178420	*BC034204*	-2.08	10.71	0.018
NM_025968	*Ltb4dh*	-2.08	13.17	0.020
NM_027817	*Grap*	-2.08	9.88	0.002
NM_025439	*Tmem9*	-2.08	13.24	0.036
NM_146356	*Olfr521*	-2.08	11.44	0.025
NM_030082	*Hist3h2ba*	-2.08	9.87	0.047
NM_013866	*Zfp385*	-2.08	12.10	0.012
NM_001033172	*Rab11fip2*	-2.08	11.54	0.018
NM_019911	*Tdo2*	-2.08	10.07	0.030
NM_026797	*Dbndd2*	-2.08	13.99	0.012
NM_147061	*Olfr691*	-2.08	10.21	0.002

NM_013796	*Nagpa*	-2.09	11.86	0.008
NM_029239	*Prkcn*	-2.09	10.03	0.032
NAP053178-1	*NAP053178-1*	-2.09	10.34	0.045
NM_145555	*A330049M08Rik*	-2.09	14.62	0.015
NM_175168	*Ptk7*	-2.09	11.57	0.038
AK017136	*Ptchd3*	-2.09	10.38	0.035
NM_178673	*Fstl5*	-2.09	10.42	0.015
BC110692	*Adora2a*	-2.09	10.51	0.022
NM_133804	*Tmem132a*	-2.09	12.29	0.014
AK038734	*A230058J24Rik*	-2.09	10.46	0.010
NM_025594	*Zmat2*	-2.09	16.35	0.022
NM_031872	*Tas1r3*	-2.10	10.44	0.006
AK002747	*Galnt14*	-2.10	7.62	0.021
NM_207583	*6430517E21Rik*	-2.10	11.06	0.036
NM_011651	*Stk22s1*	-2.10	10.64	0.016
NM_008512	*Lrp1*	-2.10	12.84	0.003
NM_007979	*F9*	-2.10	12.50	0.040
NM_022313	*Eral1*	-2.10	11.15	0.048
NM_133765	*Fbxo31*	-2.10	13.03	0.023
AK036809	*9930013L23Rik*	-2.10	10.63	0.009
AK033799	*Zdhhc14*	-2.11	9.78	0.027
NM_007558	*Bmp8a*	-2.11	15.55	0.028
NM_029600	*Abcc3*	-2.11	10.71	0.011
AJ245904	*Shank3*	-2.11	10.25	0.009
NM_024253	*Nkg7*	-2.11	9.31	0.009
D13903	*Ptprd*	-2.11	13.70	0.036
NM_025581	*2810433K01Rik*	-2.11	9.85	0.007
NM_175162	*Stox2*	-2.11	10.92	0.023
NM_144790	*Ankrd33*	-2.11	10.55	0.008
NM_008492	*Ldhb*	-2.11	13.46	0.004
AK032822	*6720458D17Rik*	-2.11	10.93	0.007
NM_016973	*St6galnac6*	-2.11	10.58	0.002
NM_001011811	*Olfr487*	-2.11	10.36	0.014
NM_007627	*Cckbr*	-2.12	9.98	0.008
NM_144914	*BC019731*	-2.12	10.88	0.041
S74315	*S74315*	-2.12	10.77	0.012
NAP062889-1	*NAP062889-1*	-2.12	11.01	0.014
NM_020498	*Ly6i*	-2.12	10.89	0.018
AK045240	*B130050I23Rik*	-2.12	10.57	0.045
NM_026439	*Ccdc80*	-2.12	18.13	0.006
NM_022317	*Slc28a3*	-2.12	10.27	0.040
AY352586	*AY352586*	-2.12	10.40	0.005
NAP070564-1	*NAP070564-1*	-2.12	11.61	0.019
NM_028533	*1700065D16Rik*	-2.12	10.18	0.004
NM_177322	*Agtr1a*	-2.12	9.93	0.014
NM_007600	*Capn1*	-2.12	10.94	0.014
NM_145532	*Mall*	-2.12	13.88	0.015
AK129420	*Tubgcp6*	-2.13	9.78	0.020
NM_178162	*Hrbl*	-2.13	12.22	0.012
NM_026385	*Pllp*	-2.13	10.89	0.037
NM_008574	*Smcp*	-2.13	9.41	0.005
NM_011226	*Rab19*	-2.13	9.36	0.023
NM_008398	*Itga7*	-2.13	12.08	0.025
NM_182785	*Lypd4*	-2.13	9.21	0.008
NM_013748	*Clnk*	-2.14	11.06	0.029
AK037945	*Zswim6*	-2.14	11.61	0.011
AK034052	*9330151L19Rik*	-2.14	11.70	0.012
NM_008320	*Irf8*	-2.14	9.88	0.039
BG067131	*BG067131*	-2.14	10.48	0.011

NM_146247	*BC024814*	-2.14	10.93	0.021
NAP023145-001	*NAP023145-001*	-2.14	10.77	0.016
AK076088	*Hsd17b12*	-2.14	9.36	0.036
AK014771	*Pnpla5*	-2.14	10.46	0.003
NM_030017	*Rdh12*	-2.14	13.78	0.049
NM_175672	*4930579C12Rik*	-2.15	10.28	0.001
NM_001025612	*Snx22*	-2.15	11.59	0.008
NAP039199-1	*NAP039199-1*	-2.15	10.34	0.015
NM_007741	*Col9a2*	-2.15	17.85	0.012
AK007167	*1700111A04Rik*	-2.15	9.79	0.006
NM_011948	*Map3k4*	-2.15	11.36	0.010
NM_020276	*Nelf*	-2.15	13.60	0.015
NM_177222	*Casc1*	-2.15	10.69	0.017
BC004622	*Gas5*	-2.15	10.86	0.020
XM_620995	*LOC546296*	-2.15	10.01	0.000
NM_013862	*Rabgap1l*	-2.15	9.68	0.005
AK006248	*Gsdmdc2*	-2.15	10.29	0.003
NM_146432	*Olfr1512*	-2.15	9.48	0.009
NM_172859	*2810039F03Rik*	-2.15	9.60	0.042
NM_011784	*Agtrl1*	-2.16	10.09	0.004
NM_001037247	*Defb36*	-2.16	10.05	0.007
AK008024	*Slc7a15*	-2.16	10.00	0.005
NM_019636	*Tbc1d1*	-2.16	11.12	0.020
NM_146308	*Olfr1356*	-2.16	9.74	0.038
NM_028661	*1110006G06Rik*	-2.16	13.55	0.003
NAP052266-1	*NAP052266-1*	-2.16	10.15	0.003
NM_080452	*Mrps2*	-2.16	11.88	0.034
AK017625	*Myl6b*	-2.16	11.32	0.004
XM_131770	*Otud3*	-2.16	14.31	0.038
X98368	*Sox19*	-2.16	10.52	0.019
XM_486328	*6430704N06*	-2.16	10.45	0.009
NM_009482	*Utf1*	-2.16	11.68	0.025
NM_023224	*Cblc*	-2.17	9.95	0.001
NM_146907	*Olfr1282*	-2.17	10.39	0.010
NM_178600	*Vkorc1*	-2.17	17.47	0.005
NM_029416	*Klf17*	-2.17	9.58	0.017
BC028808	*BC028808*	-2.17	10.56	0.006
NM_153062	*Slc37a1*	-2.17	10.97	0.000
NM_008884	*Pml*	-2.17	12.00	0.031
NM_026785	*Ube2c*	-2.17	11.46	0.043
NM_019798	*Pde4a*	-2.17	10.99	0.038
NM_153080	*Tom1l2*	-2.17	10.38	0.010
NM_001039076	*Ldb3*	-2.17	10.75	0.004
NM_018815	*Nup210*	-2.17	11.39	0.014
NM_178072	*Glcci1*	-2.17	10.91	0.014
NM_023637	*Sars2*	-2.18	11.78	0.023
NM_019568	*Cxcl14*	-2.18	10.46	0.037
BC062921	*Zfp335*	-2.18	13.40	0.049
AK006609	*1700036A12Rik*	-2.18	10.20	0.014
BC027311	*2310007F21Rik*	-2.18	13.84	0.005
AK051169	*D130009I18Rik*	-2.18	10.41	0.005
NM_008172	*Grin2d*	-2.18	10.98	0.012
BQ033444	*BQ033444*	-2.18	10.28	0.010
NM_019874	*Dnajb5*	-2.18	10.63	0.013
NM_146289	*Olfr113*	-2.19	9.43	0.036
NM_146536	*Olfr313*	-2.19	10.22	0.001
AK049822	*Synj1*	-2.19	10.18	0.006
AK019592	*4930432J01Rik*	-2.19	10.55	0.008
NM_134003	*Zc3h10*	-2.19	12.68	0.046

NM_024188	*Oxct1*	-2.19	15.81	0.018
NM_029794	*D11Ertd636e*	-2.19	10.38	0.015
NM_201365	*BC050210*	-2.19	10.22	0.003
AK053721	*AK053721*	-2.19	9.56	0.007
NM_145713	*Hist1h1d*	-2.19	10.14	0.037
XM_138369	*LOC380824*	-2.19	9.91	0.006
NM_026621	*2810046M22Rik*	-2.19	10.42	0.018
NM_177231	*Arrb1*	-2.20	10.34	0.026
NM_207222	*Lmo3*	-2.20	10.60	0.013
NM_053199	*Igsf4b*	-2.20	9.54	0.006
NM_172553	*Cart1*	-2.20	9.43	0.022
NM_011792	*Bace1*	-2.20	13.42	0.014
NM_011935	*Esrrg*	-2.20	10.03	0.011
AK029248	*5530401A14Rik*	-2.20	8.34	0.001
NM_023258	*Pycard*	-2.20	11.46	0.006
NM_021546	*Apba2bp*	-2.20	11.78	0.021
NM_080464	*Ppp1r3a*	-2.20	9.55	0.023
NM_011199	*Pthr1*	-2.20	17.51	0.010
NM_029649	*Tmem101*	-2.20	13.96	0.035
AK016680	*4933406B17Rik*	-2.20	9.77	0.006
NM_201354	*Gm672*	-2.20	10.88	0.018
NM_178406	*Gpr153*	-2.20	12.60	0.002
NM_008236	*Hes2*	-2.21	10.07	0.006
NM_130864	*Acaa1a*	-2.21	15.53	0.016
NM_153537	*Phldb1*	-2.21	13.62	0.001
NM_031196	*Slc19a1*	-2.21	9.90	0.008
NM_145123	*Crtac1*	-2.21	10.56	0.020
NM_025958	*Cand2*	-2.21	9.78	0.026
AK076376	*Btbd14a*	-2.21	10.52	0.020
NM_007590	*Calm3*	-2.21	15.08	0.006
NM_008797	*Pcx*	-2.21	14.10	0.035
AK140530	*Plcl1*	-2.21	9.88	0.008
U17282	*Atp4a*	-2.21	10.04	0.000
BC098210	*Pla2g4b*	-2.21	10.30	0.017
NM_148946	*Slc8a2*	-2.21	10.36	0.000
NM_147220	*Abca9*	-2.21	9.31	0.031
NM_177118	*A830073O21Rik*	-2.22	11.04	0.020
NM_008591	*Met*	-2.22	10.04	0.005
NM_010725	*Lmx1b*	-2.22	10.26	0.006
AK220521	*Mypn*	-2.22	10.15	0.007
NM_008092	*Gata4*	-2.22	10.28	0.006
XR_004813	*LOC675674*	-2.22	9.92	0.007
NM_172661	*5830434P21Rik*	-2.22	10.49	0.006
NM_007679	*Cebpd*	-2.22	13.16	0.014
NM_177242	*Pptc7*	-2.22	14.84	0.017
NM_016745	*Atp2a3*	-2.22	12.03	0.016
BC009817	*Btn1a1*	-2.22	8.08	0.019
NM_019739	*Foxo1*	-2.22	13.25	0.050
NM_001033344	*Dusp27*	-2.22	9.89	0.022
BC034239	*Chd7*	-2.22	11.35	0.045
NM_008905	*Ppfibp2*	-2.22	9.99	0.016
NM_009139	*Ccl6*	-2.23	9.32	0.029
NM_172588	*Serinc5*	-2.23	11.09	0.006
BC099973	*Rhobtb1*	-2.23	10.96	0.035
NM_007435	*Abcd1*	-2.23	10.29	0.002
NM_007904	*Ednrb*	-2.23	11.54	0.007
NM_207680	*Bcl2l11*	-2.23	11.41	0.007
AK083360	*C920021A13*	-2.23	9.92	0.012
NM_022026	*Aqp9*	-2.23	10.13	0.006

NM_019696	*Cpxm1*	-2.23	13.86	0.023
NM_011522	*Syngr3*	-2.23	11.73	0.007
NM_177373	*Ppfia2*	-2.23	9.96	0.020
NM_001008233	*Plekhn1*	-2.23	11.13	0.013
NM_023402	*Mylc2b*	-2.23	13.40	0.006
NM_001001489	*BC021785*	-2.23	10.44	0.010
NM_147031	*Olfr1122*	-2.23	10.01	0.002
NM_194348	*1810013C15Rik*	-2.23	10.88	0.012
XM_001000569	*Taf1*	-2.23	10.25	0.014
AK035164	*Hmga2*	-2.24	9.68	0.003
NM_009219	*Sstr4*	-2.24	10.14	0.001
XM_903670	*Etnk2*	-2.24	11.24	0.027
NM_013524	*Fut7*	-2.24	10.69	0.003
NM_019999	*Pnkd*	-2.24	10.76	0.014
NM_178235	*Slco1b2*	-2.24	11.12	0.009
BC003498	*4931420C21Rik*	-2.24	13.26	0.033
AK012219	*2700008E08Rik*	-2.24	9.88	0.011
AK038807	*Slit2*	-2.24	9.30	0.011
AK088706	*E430024C06Rik*	-2.24	12.86	0.047
NM_001001885	*Tmem151*	-2.24	12.11	0.041
NM_011749	*Zfp148*	-2.24	13.59	0.046
NM_028852	*Spatc1*	-2.24	10.22	0.000
NM_018766	*Ntsr1*	-2.24	10.28	0.002
NM_009932	*Col4a2*	-2.24	10.77	0.023
NM_008507	*Sh2b3*	-2.25	9.92	0.002
AK038887	*2610307P16Rik*	-2.25	9.50	0.041
NM_001001183	*BC054438*	-2.25	10.28	0.012
BC028487	*Cck*	-2.25	10.39	0.010
NM_021299	*Ak3*	-2.25	15.27	0.018
NM_027874	*Csnk1d*	-2.25	11.92	0.002
NM_008750	*Nxn*	-2.25	12.97	0.006
NM_026633	*9530058B02Rik*	-2.25	11.38	0.008
NM_007421	*Adssl1*	-2.25	11.07	0.041
NM_009330	*Tcf2*	-2.25	11.06	0.014
NM_134197	*V1re8*	-2.25	10.04	0.009
NM_007739	*Col8a1*	-2.26	10.60	0.050
NM_009287	*Stim1*	-2.26	12.95	0.008
NM_177364	*G431001E03Rik*	-2.26	11.12	0.007
NM_031499	*Prp2*	-2.26	11.75	0.015
NM_013530	*Gnb3*	-2.26	10.00	0.021
NM_146764	*Olfr1408*	-2.26	9.74	0.005
NM_021284	*Kras*	-2.27	12.01	0.016
NM_133485	*Ppp1r14c*	-2.27	10.80	0.031
NM_008521	*Ltc4s*	-2.27	10.05	0.013
BX515732	*BX515732*	-2.27	10.62	0.013
NM_147093	*Olfr558*	-2.27	9.78	0.015
NM_008679	*Ncoa3*	-2.27	10.83	0.020
NM_024479	*Wbscr27*	-2.27	13.78	0.010
AK018161	*Dock4*	-2.27	8.70	0.005
BC022221	*Usp53*	-2.27	14.20	0.011
AK041423	*Arhgef18*	-2.27	9.79	0.003
NM_001033227	*Slc5a10*	-2.27	11.17	0.024
NM_144828	*Ppp1r1b*	-2.27	11.12	0.026
NM_172506	*Boc*	-2.28	10.38	0.009
XM_001006035	*LOC673641*	-2.28	9.37	0.020
NM_011143	*Pou4f1*	-2.28	10.61	0.016
NM_032002	*Nrg4*	-2.28	10.99	0.006
XM_899897	*Cenpf*	-2.28	10.33	0.012
NM_001024138	*Gpr139*	-2.28	10.32	0.001

AK150917	*Ctla2b*	-2.28	9.52	0.005
NM_008936	*Prop1*	-2.28	10.54	0.010
NM_007953	*Esrra*	-2.28	10.87	0.035
NM_008457	*Klk1b8*	-2.28	10.01	0.020
NM_009893	*Chrd*	-2.28	11.64	0.023
NM_007495	*Astn1*	-2.28	9.49	0.017
NM_001011662	*Olfr180*	-2.28	9.82	0.005
NM_011379	*Sipa1*	-2.28	10.58	0.000
NM_146395	*Olfr1276*	-2.28	10.34	0.008
AK141695	*Gm98*	-2.28	10.59	0.010
XM_915937	*LOC639490*	-2.28	12.55	0.003
NM_130457	*Cntnap4*	-2.28	9.30	0.005
NM_019923	*Itpr2*	-2.29	13.76	0.029
NM_146293	*Olfr1143*	-2.29	11.31	0.040
NM_011983	*Homer2*	-2.29	10.14	0.015
NM_010616	*Kif12*	-2.29	10.63	0.003
NM_028157	*1700013B16Rik*	-2.29	11.51	0.002
NM_016692	*Incenp*	-2.29	12.86	0.043
NM_207149	*Olfr1010*	-2.29	9.58	0.004
NM_145922	*Kcnc4*	-2.29	10.75	0.013
NM_053082	*Tspan4*	-2.29	16.35	0.002
NM_016741	*Scarb1*	-2.29	15.51	0.010
AK147232	*Lnpep*	-2.29	9.98	0.007
NM_011599	*Tle1*	-2.29	12.09	0.026
NP431243	*NP431243*	-2.30	10.17	0.018
BC048462	*1700001K19Rik*	-2.30	12.00	0.004
NM_019566	*Rhog*	-2.30	15.06	0.008
NM_001001805	*Olfr576*	-2.30	9.70	0.002
NM_201352	*Gdpd5*	-2.30	10.08	0.045
NM_016866	*Stk39*	-2.30	16.45	0.005
NM_001004148	*Slc13a5*	-2.30	11.48	0.005
NM_009358	*Ppp2r5d*	-2.30	14.20	0.032
NM_007709	*Cited1*	-2.30	10.89	0.012
AK052902	*AK052902*	-2.30	10.26	0.007
XM_992182	*Csnk2a2*	-2.30	13.52	0.029
NM_001013378	*Uspl1*	-2.31	13.57	0.024
BC027193	*Fbs1*	-2.31	10.93	0.012
NM_207136	*Olfr1349*	-2.31	10.63	0.011
AK016037	*Rnf121*	-2.31	11.17	0.023
NM_173402	*Rgs12*	-2.31	13.16	0.041
NM_146914	*Olfr5*	-2.31	12.81	0.032
NM_173370	*Cds1*	-2.31	13.27	0.036
AK083796	*AK083796*	-2.31	11.35	0.036
NM_010278	*Gfi1*	-2.31	10.57	0.005
NM_001033360	*Gpr101*	-2.31	10.41	0.005
NM_021718	*Ms4a4b*	-2.32	10.06	0.004
NM_011427	*Snai1*	-2.32	12.61	0.039
NM_026039	*Med18*	-2.32	10.00	0.023
AK046928	*4732479N06Rik*	-2.32	10.34	0.013
NM_177767	*Ogfod1*	-2.32	10.29	0.008
NM_177580	*Baiap2l2*	-2.32	10.30	0.007
BU530348	*AW046674*	-2.32	10.71	0.006
AK036220	*Rbm18*	-2.32	11.76	0.030
NM_027423	*Polr3b*	-2.32	13.01	0.018
NM_172546	*Cnksr3*	-2.32	12.27	0.004
NM_008851	*Pitpnm1*	-2.32	12.97	0.020
NM_017371	*Hpxn*	-2.32	10.59	0.009
NM_011129	*38231*	-2.32	10.52	0.025
NM_007645	*Cd37*	-2.32	9.98	0.001

NM_145229	*AY074887*	-2.32	10.13	0.034
NM_008312	*Htr2c*	-2.32	9.40	0.003
XM_144076	*Lrrc38*	-2.33	11.59	0.011
NM_015739	*Gbx1*	-2.33	10.50	0.007
AK029271	*1110036O03Rik*	-2.33	14.18	0.044
NM_010681	*Lama4*	-2.33	13.65	0.031
AK030181	*4933413A10Rik*	-2.33	16.54	0.007
AJ250689	*AJ250689*	-2.33	10.63	0.011
NM_146827	*Olfr983*	-2.33	9.74	0.019
AK084087	*D130020G16Rik*	-2.33	11.25	0.015
NM_206816	*Olfr128*	-2.33	10.69	0.006
NM_008168	*Grik5*	-2.33	11.20	0.006
AK014401	*Syt11*	-2.33	10.31	0.034
NM_011201	*Ptpn1*	-2.34	14.61	0.015
NM_173069	*Speer2*	-2.34	9.78	0.001
XM_885736	*Fath*	-2.34	10.20	0.014
NM_026230	*H2afy3*	-2.34	13.73	0.001
NM_009374	*Tgm3*	-2.34	10.33	0.005
NM_010433	*Hipk2*	-2.34	10.22	0.000
BC052065	*Rap1gap*	-2.34	12.37	0.002
NAP057058-1	*NAP057058-1*	-2.34	7.89	0.013
NM_031198	*Tcfec*	-2.34	10.42	0.016
BC071254	*BC071254*	-2.35	15.48	0.030
NM_021415	*Cacna1h*	-2.35	10.64	0.005
NM_053256	*Tifp39*	-2.35	11.88	0.037
NM_009525	*Wnt5b*	-2.35	14.41	0.013
XM_139711	*Arid1b*	-2.35	13.26	0.018
AK089219	*AK089219*	-2.35	10.66	0.007
AK084964	*A930033H14Rik*	-2.35	10.30	0.007
NM_011150	*Lgals3bp*	-2.35	12.96	0.048
NAP102554-1	*NAP102554-1*	-2.35	11.10	0.006
NM_010333	*Edg5*	-2.35	10.12	0.012
NM_207683	*Pik3c2g*	-2.36	11.38	0.050
NM_010009	*Cyp27b1*	-2.36	10.06	0.008
NM_009793	*Camk4*	-2.36	10.71	0.023
NM_178257	*Il22ra1*	-2.36	9.71	0.012
AK003646	*Cxx1b*	-2.36	16.35	0.012
NM_024452	*Luzp1*	-2.36	12.50	0.007
NM_145933	*St6gal1*	-2.36	10.98	0.030
NM_009117	*Saa1*	-2.36	11.20	0.001
NM_025326	*0610011I04Rik*	-2.36	12.99	0.015
NM_181751	*Gpr119*	-2.36	10.24	0.004
NM_009844	*Cd19*	-2.37	10.84	0.007
NM_016933	*Ptprcap*	-2.37	12.80	0.002
NM_007564	*Zfp36l1*	-2.37	13.98	0.005
NM_133891	*Slc44a1*	-2.37	12.10	0.005
NAP048481-1	*NAP048481-1*	-2.37	11.55	0.027
AK004474	*1190003M12Rik*	-2.37	10.78	0.001
NM_011121	*Plk1*	-2.38	10.99	0.003
AK169695	*Plcg1*	-2.38	13.15	0.006
NM_008675	*Nbl1*	-2.38	12.41	0.016
NM_134002	*Csnk1g2*	-2.38	14.36	0.034
NM_145387	*Athl1*	-2.38	12.15	0.040
NM_008790	*Pcp2*	-2.38	12.80	0.014
AK041224	*Klre1*	-2.38	9.85	0.001
NM_010899	*Nfatc2*	-2.38	10.24	0.002
NM_001025103	*Efcab4a*	-2.38	10.81	0.011
NM_197990	*1700025G04Rik*	-2.38	14.06	0.048
NM_028195	*Pscd4*	-2.39	12.13	0.011

AK083104	*Extl1*	-2.39	10.68	0.039
NM_133669	*Rp2h*	-2.39	13.47	0.041
NM_001029936	*Specc1*	-2.39	10.26	0.014
NM_178650	*Tbc1d10c*	-2.39	10.26	0.002
NM_175641	*Ltbp4*	-2.39	12.04	0.015
NM_009954	*Bcar1*	-2.39	12.22	0.046
NM_145434	*Nr1d1*	-2.39	10.68	0.005
NM_133781	*Cab39*	-2.39	14.83	0.000
NM_146540	*Olfr1364*	-2.39	10.68	0.017
NM_029568	*Mfap4*	-2.40	10.85	0.005
NM_025573	*Sfrs9*	-2.40	13.99	0.011
NM_015738	*Galr3*	-2.40	12.15	0.022
NM_001004364	*Ddef2*	-2.40	13.48	0.004
NM_008983	*Ptprk*	-2.40	13.19	0.014
NM_009913	*Ccr9*	-2.40	11.77	0.014
NM_011218	*Ptprs*	-2.40	10.43	0.003
NM_030004	*Cryl1*	-2.40	14.01	0.012
AK085110	*R3hdm1*	-2.40	10.50	0.004
NM_001025106	*D4Ertd429e*	-2.41	9.71	0.012
NM_177833	*C230050L11*	-2.41	9.87	0.011
AK084358	*Dleu2*	-2.41	13.92	0.032
NM_026054	*2810474O19Rik*	-2.41	14.84	0.032
AK122491	*Klhl13*	-2.41	10.16	0.015
NM_001002790	*B830017H08Rik*	-2.41	11.66	0.013
NM_146630	*Olfr123*	-2.41	10.02	0.004
NM_080459	*Strc*	-2.42	10.36	0.032
NM_146411	*Olfr462*	-2.42	11.64	0.023
NM_146731	*Olfr599*	-2.42	10.20	0.001
NAP099398-001	*NAP099398-001*	-2.42	12.05	0.040
XM_356184	*1110001D15Rik*	-2.42	10.09	0.002
NM_011206	*Ptpn18*	-2.42	11.39	0.005
NM_134223	*V1ri6*	-2.42	10.83	0.035
NM_010187	*Fcgr2b*	-2.42	10.36	0.006
NM_146776	*Olfr821*	-2.43	10.53	0.014
AK015924	*4930528F23Rik*	-2.43	10.48	0.009
NM_001037910	*A530040E14Rik*	-2.43	10.28	0.031
NM_053167	*Trim9*	-2.43	10.46	0.002
NM_026988	*2610009E16Rik*	-2.43	14.87	0.017
NM_007617	*Cav3*	-2.43	12.32	0.019
NM_175429	*Kctd12b*	-2.43	10.12	0.012
NM_031197	*Slc2a2*	-2.43	10.49	0.007
NM_011346	*Sell*	-2.43	9.34	0.013
NM_025858	*Scotin*	-2.44	16.95	0.006
NM_019643	*Tera*	-2.44	11.06	0.012
AK077971	*2810439F02Rik*	-2.44	9.91	0.009
NM_029620	*Pcolce2*	-2.44	17.40	0.005
NM_001033875	*Ctrc*	-2.44	10.40	0.027
BC004786	*Igh-VJ558*	-2.44	9.67	0.022
BU504102	*BU504102*	-2.44	11.07	0.018
NM_027543	*Gpr173*	-2.44	10.83	0.010
NM_133954	*AA960436*	-2.45	10.80	0.013
NM_134180	*V1rc25*	-2.45	8.48	0.049
NM_008716	*Notch3*	-2.45	10.89	0.016
NM_172522	*Megf11*	-2.45	10.74	0.007
NM_021534	*Pxmp4*	-2.45	14.05	0.024
AK080781	*AK080781*	-2.45	10.43	0.047
NM_013732	*Cart*	-2.45	10.01	0.013
AK007436	*Adamts9*	-2.45	10.15	0.029
AV273577	*AV273577*	-2.45	10.71	0.020

NM_009337	*Tcl1*	-2.45	11.06	0.009
NAP057024-1	*NAP057024-1*	-2.45	12.10	0.001
NM_009918	*Cnga3*	-2.45	9.85	0.002
NM_007650	*Cd5*	-2.46	10.89	0.010
NM_183193	*Foxi2*	-2.46	10.22	0.009
NM_023047	*Dpysl5*	-2.46	11.01	0.009
NM_181405	*Rnpepl1*	-2.46	14.65	0.011
NM_001033147	*1700001L05Rik*	-2.46	10.12	0.005
NM_013605	*Muc1*	-2.47	10.65	0.013
NM_027192	*Ttl*	-2.47	12.24	0.028
NM_019403	*Rnf5*	-2.47	12.69	0.030
NM_181444	*Gprc5a*	-2.47	13.25	0.014
NM_178408	*Arrdc1*	-2.47	11.29	0.012
AK053849	*Gja3*	-2.47	10.51	0.034
NM_172681	*D930015E06Rik*	-2.48	9.81	0.008
NM_019880	*Mtch1*	-2.48	17.27	0.002
NM_011039	*Pax7*	-2.48	10.14	0.001
NM_026865	*1700113I22Rik*	-2.49	11.16	0.009
NM_153577	*AI428936*	-2.49	11.39	0.014
AK018143	*Cgn*	-2.49	10.58	0.001
NM_146899	*Olfr1219*	-2.49	12.63	0.046
BC021475	*2610301G19Rik*	-2.49	10.56	0.007
NM_026685	*0610009B10Rik*	-2.49	11.25	0.015
NM_021551	*Slc22a17*	-2.49	11.10	0.001
NM_172750	*Adprhl1*	-2.49	11.33	0.013
NM_011681	*Scgb1a1*	-2.50	10.55	0.011
AK005861	*1700011E24Rik*	-2.50	10.28	0.016
NM_001005477	*E130309B19Rik*	-2.50	10.00	0.006
NM_027083	*Lyzl6*	-2.50	10.19	0.029
NM_009824	*Cbfa2t3h*	-2.50	10.75	0.045
NM_177084	*Slc9a4*	-2.50	10.04	0.004
NM_026174	*Entpd4*	-2.50	10.89	0.011
NM_183037	*Trim46*	-2.50	10.91	0.008
NAP061954-1	*NAP061954-1*	-2.50	10.96	0.009
NM_011280	*Trim10*	-2.50	9.86	0.005
NM_010257	*Gast*	-2.51	10.88	0.012
AK006075	*4930558C23Rik*	-2.51	9.54	0.005
AK018447	*8430428J23Rik*	-2.51	11.47	0.003
NM_026686	*0610011F06Rik*	-2.51	12.86	0.029
AK008733	*2310010I16Rik*	-2.51	10.23	0.006
NAP108722-1	*NAP108722-1*	-2.51	10.80	0.010
BC100555	*Sox14*	-2.51	10.58	0.010
NM_145539	*Tm4sf4*	-2.51	10.80	0.014
NM_009666	*Amelx*	-2.51	11.14	0.008
AK038653	*9230112J17Rik*	-2.51	10.68	0.010
NM_019436	*Sit1*	-2.51	10.31	0.013
NM_147047	*Olfr618*	-2.51	9.03	0.014
AK147993	*Wdr25*	-2.51	10.16	0.003
NM_018755	*Pgcp*	-2.52	12.92	0.023
AK083771	*2810055G20Rik*	-2.52	10.39	0.003
NM_028539	*1700052K11Rik*	-2.52	12.83	0.025
NM_027539	*Dcamkl2*	-2.52	10.84	0.002
NM_175195	*3110056O03Rik*	-2.52	13.90	0.009
NM_028266	*Col16a1*	-2.52	13.56	0.032
NM_138748	*Ppp2r4*	-2.52	12.04	0.016
NM_146751	*Olfr648*	-2.52	10.24	0.004
NM_207666	*Egfl9*	-2.52	14.76	0.004
NM_172775	*Plxnb1*	-2.52	12.15	0.014
NM_011854	*Oasl2*	-2.52	8.67	0.040

NM_031186	*Ndst3*	-2.53	10.21	0.004
NM_022723	*Scube1*	-2.53	10.66	0.001
NM_009868	*Cdh5*	-2.53	10.43	0.000
NM_001024619	*Lrrc54*	-2.53	11.31	0.017
NM_013484	*C2*	-2.53	10.80	0.016
NM_177595	*Mkx*	-2.53	13.66	0.039
NM_144845	*Ugt3a2*	-2.53	11.02	0.039
NM_001014394	*Gpr113*	-2.53	11.31	0.030
NM_007620	*Cbr1*	-2.54	12.08	0.045
NM_146469	*Olfr1381*	-2.54	10.48	0.010
NM_173397	*D930001I22Rik*	-2.54	12.86	0.012
NM_026815	*Upk1a*	-2.54	10.58	0.014
NM_009799	*Car1*	-2.54	11.06	0.017
BC050125	*Adcy1*	-2.54	10.85	0.005
NM_027180	*Centd2*	-2.54	11.86	0.033
NM_026555	*Rcn3*	-2.54	17.76	0.000
NM_008538	*Marcks*	-2.54	13.28	0.016
NM_011305	*Rxra*	-2.54	13.45	0.042
NM_007872	*Dnmt3a*	-2.54	13.66	0.036
NM_010255	*Gamt*	-2.54	11.99	0.020
AK129139	*Mtss1*	-2.55	12.41	0.001
NM_011175	*Lgmn*	-2.55	14.88	0.006
NM_007502	*Atp1b3*	-2.55	16.52	0.020
NM_010387	*H2-DMb1*	-2.55	15.48	0.012
XM_283804	*9830001H06Rik*	-2.55	12.09	0.016
NM_175667	*Ankrd5*	-2.55	10.60	0.007
NM_011131	*Pold1*	-2.56	11.95	0.042
NM_001033153	*2610203C20Rik*	-2.56	10.49	0.000
NM_175259	*Tmem58*	-2.56	15.32	0.012
AK040949	*Lincr*	-2.56	10.12	0.035
NAP039495-1	*NAP039495-1*	-2.56	10.38	0.006
NM_153114	*Otos*	-2.56	11.70	0.008
NM_133206	*Znrf1*	-2.56	12.32	0.004
NM_133738	*Antxr2*	-2.56	14.71	0.002
NM_011256	*Pitpnm2*	-2.56	10.28	0.011
NM_175026	*AI447904*	-2.56	10.00	0.001
NM_016846	*Rgl1*	-2.57	11.13	0.010
NM_146706	*Olfr401*	-2.57	10.35	0.008
AK044977	*Evc2*	-2.57	10.32	0.009
NM_008566	*Mcm5*	-2.57	11.56	0.008
AK008004	*Txnrd2*	-2.57	12.43	0.048
NM_010118	*Egr2*	-2.57	10.94	0.014
NM_029935	*4631426J05Rik*	-2.58	12.33	0.046
NM_009514	*Vpreb3*	-2.58	10.35	0.001
XM_619795	*Ryr3*	-2.58	10.63	0.003
NM_013770	*Slc25a10*	-2.58	12.89	0.044
BC052391	*Gpr125*	-2.58	15.03	0.022
NM_028118	*Wdsub1*	-2.58	10.55	0.007
NAP059351-1	*NAP059351-1*	-2.58	10.41	0.013
AK081687	*AK081687*	-2.58	10.35	0.018
NM_001014900	*Zmynd12*	-2.59	9.71	0.005
NM_007809	*Cyp17a1*	-2.59	11.36	0.013
AK085511	*Gm166*	-2.59	11.15	0.011
NM_007523	*Bak1*	-2.59	11.86	0.018
NM_008550	*Man2b2*	-2.59	10.91	0.035
NM_145463	*Tmem46*	-2.60	11.92	0.042
NM_007491	*Art5*	-2.60	11.20	0.016
NM_080559	*Sh3bgrl3*	-2.60	15.71	0.044
NM_008171	*Grin2b*	-2.60	11.01	0.026

U26474	*U26474*	-2.60	13.02	0.047
NAP108488-1	*NAP108488-1*	-2.60	10.79	0.001
NM_011977	*Slc27a1*	-2.60	12.09	0.040
NM_007533	*Bckdha*	-2.60	10.68	0.007
NM_010566	*Inpp5d*	-2.60	10.82	0.001
NM_023680	*Tnfrsf22*	-2.60	12.48	0.016
NM_021323	*Usp29*	-2.60	10.25	0.002
NM_030679	*Myh1*	-2.60	10.52	0.008
BC058264	*Tshz1*	-2.60	11.79	0.025
NM_008766	*Slc22a6*	-2.61	10.22	0.012
NM_153505	*Nckap1l*	-2.61	10.75	0.012
NM_010188	*Fcgr3*	-2.62	9.45	0.017
AB041809	*Gfra4*	-2.62	12.42	0.006
NM_009217	*Sstr2*	-2.62	10.96	0.015
NM_028386	*Asphd2*	-2.62	11.37	0.009
NM_008434	*Kcnq1*	-2.62	10.64	0.012
U63712	*Tfam*	-2.62	13.22	0.005
NM_139302	*Sh3glb2*	-2.63	11.82	0.013
NM_146711	*Olfr43*	-2.63	10.99	0.011
NM_008452	*Klf2*	-2.63	12.61	0.007
NAP000142-003	*NAP000142-003*	-2.63	14.56	0.043
XM_148382	*1700026J12Rik*	-2.63	9.64	0.002
NM_146707	*Olfr410*	-2.63	10.20	0.001
NM_008239	*Foxq1*	-2.63	12.83	0.005
NM_013460	*Adra1d*	-2.63	11.53	0.046
NM_008121	*Gja5*	-2.63	10.40	0.005
NM_008113	*Arhgdig*	-2.64	10.84	0.015
XM_975685	*LOC546020*	-2.64	10.81	0.010
NM_146472	*Olfr1384*	-2.64	9.80	0.000
NM_018862	*Agpat1*	-2.64	10.92	0.009
NM_001033981	*F830116E18Rik*	-2.64	10.18	0.003
NM_010567	*Inppl1*	-2.64	13.68	0.011
NM_176912	*Gpr77*	-2.64	10.31	0.038
AK016577	*Ttll11*	-2.64	10.31	0.050
NM_027149	*2310040A13Rik*	-2.64	10.63	0.009
BC016231	*Gtf2h2*	-2.64	12.01	0.028
BC006743	*LOC638575*	-2.64	10.41	0.021
AK002304	*0610007N19Rik*	-2.64	14.98	0.022
NM_011563	*Prdx2*	-2.64	15.62	0.001
NM_010724	*Psmb8*	-2.64	10.39	0.009
NM_183160	*E030010A14Rik*	-2.64	9.99	0.003
NM_025994	*Efhd2*	-2.64	14.94	0.041
AK050314	*AK050314*	-2.65	13.19	0.047
NM_011655	*Tubb5*	-2.65	16.14	0.008
NM_146981	*Olfr1260*	-2.65	10.02	0.006
NM_008026	*Fli1*	-2.65	11.73	0.004
NM_181729	*Muc6*	-2.65	10.72	0.006
NM_207673	*Olfr100*	-2.65	11.47	0.038
AK076297	*Col27a1*	-2.66	12.65	0.023
NM_026950	*Ociad2*	-2.66	11.54	0.005
AK029105	*Itga1*	-2.66	10.99	0.026
NM_029537	*Tmem98*	-2.66	12.97	0.008
AK019590	*4930430O22Rik*	-2.66	10.73	0.005
NM_144816	*Rhbdl1*	-2.66	10.99	0.012
NM_025598	*2700038C09Rik*	-2.66	15.85	0.013
NM_025659	*Abi3*	-2.66	10.80	0.022
NM_177087	*4632412N22Rik*	-2.66	11.17	0.007
NM_177834	*Cpa6*	-2.66	11.34	0.018
NM_146628	*Olfr344*	-2.67	9.87	0.001

XM_131700	*BC039093*	-2.67	13.39	0.047
NM_146759	*Olfr214*	-2.67	10.64	0.013
NM_018771	*Gipc1*	-2.67	13.21	0.039
NM_008136	*Gna-rs1*	-2.67	12.55	0.045
AK220117	*AK220117*	-2.67	11.14	0.046
NM_013753	*X99384*	-2.67	11.66	0.001
NM_029440	*4930434E21Rik*	-2.67	10.61	0.003
AK129235	*Taok2*	-2.68	13.04	0.049
NM_172741	*4931406P16Rik*	-2.68	12.86	0.019
NM_001024931	*D11Bwg0517e*	-2.68	10.23	0.000
NM_011612	*Tnfrsf9*	-2.68	10.31	0.017
NM_010050	*Dio2*	-2.68	12.09	0.034
NM_026515	*2810417H13Rik*	-2.68	12.20	0.035
NM_010864	*Myo5a*	-2.69	14.96	0.002
NM_053267	*Selm*	-2.69	15.85	0.031
AK014173	*Tbkbp1*	-2.69	10.83	0.003
NM_008120	*Gja4*	-2.69	11.55	0.008
NM_021717	*Nrip2*	-2.69	10.43	0.001
NM_023504	*Nkx2-4*	-2.69	11.86	0.027
NM_020516	*Slc16a8*	-2.70	11.07	0.039
NM_007708	*Cit*	-2.70	10.58	0.000
AK140163	*Arsb*	-2.70	10.04	0.017
NM_173396	*Tgif2*	-2.70	12.13	0.028
NM_146230	*Acaa1b*	-2.70	12.66	0.039
NM_181855	*Magea9*	-2.71	10.83	0.009
NM_001008499	*Taar4*	-2.71	10.54	0.002
NM_172869	*Frmd3*	-2.71	11.86	0.028
XM_900104	*Prok1*	-2.71	10.96	0.012
NM_153067	*Mrgpra3*	-2.71	10.09	0.006
NM_001011804	*Olfr1211*	-2.71	10.77	0.001
NM_175145	*Tmem127*	-2.71	14.64	0.003
NM_080456	*Mrps6*	-2.72	15.41	0.003
NM_133199	*Scn4a*	-2.72	12.03	0.015
NM_147109	*Olfr577*	-2.72	10.18	0.003
NM_010315	*Gng2*	-2.72	14.07	0.007
NM_172432	*C030002O17Rik*	-2.73	10.28	0.026
BC052322	*Ptprf*	-2.73	16.60	0.001
NM_009177	*St3gal1*	-2.73	14.06	0.003
NM_197959	*3000004C01Rik*	-2.73	9.53	0.001
AJ250688	*AJ250688*	-2.73	11.08	0.009
AK084405	*AK084405*	-2.74	11.16	0.014
NM_172704	*Dnajc11*	-2.74	9.61	0.001
NM_146614	*Olfr971*	-2.74	11.30	0.044
XM_619279	*Tbc1d4*	-2.74	11.54	0.004
NM_011477	*Sprr2k*	-2.74	13.23	0.005
NM_008047	*Fstl1*	-2.74	12.11	0.021
NM_023598	*Arid5b*	-2.74	10.96	0.001
NM_178593	*Rcsd1*	-2.75	12.63	0.012
NM_133791	*Wwc2*	-2.75	13.18	0.014
NM_207217	*Itfg3*	-2.75	14.97	0.001
NM_172890	*Slc6a11*	-2.76	11.02	0.007
NM_023223	*Cdc20*	-2.76	12.75	0.030
AK013800	*2900078C09Rik*	-2.76	10.25	0.006
BC026458	*Hexim2*	-2.76	12.08	0.013
D86232	*Ly6c*	-2.76	12.71	0.049
NM_010818	*Cd200*	-2.76	14.28	0.006
NM_153100	*Rtp3*	-2.76	10.33	0.014
NM_177194	*A130023I24Rik*	-2.76	9.63	0.006
AK032937	*AK032937*	-2.77	11.66	0.019

NM_178239	*Ndor1*	-2.77	11.50	0.006
NM_172945	*Ankrd13b*	-2.77	10.80	0.006
BC006867	*Tex10*	-2.77	10.60	0.015
NM_008747	*Ntsr2*	-2.77	10.72	0.008
NM_008272	*Hoxc9*	-2.78	12.54	0.012
NM_001013365	*Osm*	-2.78	10.63	0.010
NM_153390	*Pxt1*	-2.78	10.45	0.005
NM_144850	*Rapgef3*	-2.78	13.37	0.046
NM_134213	*V1rh4*	-2.78	9.36	0.014
NM_009852	*Cd6*	-2.78	11.49	0.004
NM_019511	*Ramp3*	-2.78	11.51	0.012
NM_008321	*Id3*	-2.78	12.15	0.008
NM_183390	*Klhl6*	-2.79	10.04	0.011
AK080318	*Fmnl2*	-2.79	11.00	0.008
NM_032398	*Plvap*	-2.79	11.84	0.003
NM_139228	*Rhbdl3*	-2.79	10.86	0.009
NM_178638	*Tmem108*	-2.80	11.19	0.004
XM_142262	*Ptchd1*	-2.80	10.24	0.041
NM_027562	*Clec2g*	-2.80	10.69	0.007
NM_007905	*Phc1*	-2.80	12.36	0.012
NM_019572	*Hdac7a*	-2.80	10.81	0.020
NM_009303	*Syngr1*	-2.80	11.35	0.038
NM_001002927	*Penk1*	-2.81	13.31	0.020
BC028539	*H2afv*	-2.81	16.50	0.010
NM_009233	*Sox1*	-2.81	10.72	0.005
NAP067058-1	*NAP067058-1*	-2.81	10.56	0.005
NM_033217	*Ngfr*	-2.81	11.02	0.005
NM_011519	*Sdc1*	-2.81	11.21	0.013
BC075715	*Dmpk*	-2.81	10.81	0.006
NM_183031	*Ebi2*	-2.81	9.50	0.022
NM_177069	*E330009P21Rik*	-2.81	11.20	0.014
NM_011565	*Tead2*	-2.81	10.68	0.025
NM_008456	*Klk1b5*	-2.81	10.01	0.003
NM_172739	*Grlf1*	-2.82	13.38	0.033
NM_025609	*Map3k7ip1*	-2.82	13.52	0.009
NM_147101	*Olfr549*	-2.82	11.98	0.005
AK199310	*AK199310*	-2.82	12.65	0.045
NM_174849	*9430057O19Rik*	-2.82	11.41	0.009
NM_146202	*BC026432*	-2.82	14.36	0.046
NM_008319	*Icam5*	-2.82	10.99	0.006
NM_030127	*Htra3*	-2.82	14.20	0.007
BI151587	*BI151587*	-2.82	10.47	0.012
NM_008726	*Nppb*	-2.82	10.74	0.000
NM_145712	*Mtnr1b*	-2.82	10.67	0.008
NM_007469	*Apoc1*	-2.83	10.91	0.017
NM_023908	*Slco3a1*	-2.83	10.92	0.011
NM_178745	*6330442E10Rik*	-2.83	12.09	0.011
NM_080561	*2810055G22Rik*	-2.83	11.76	0.013
AK046472	*B230396O12Rik*	-2.83	12.63	0.018
AK083039	*C530036F05Rik*	-2.83	11.07	0.007
BU511373	*H1fx*	-2.83	12.13	0.005
AK007241	*1700122O11Rik*	-2.84	11.27	0.041
NM_026552	*Arpc4*	-2.84	14.94	0.005
AK007164	*1700110M21Rik*	-2.84	10.80	0.001
AK017437	*LOC432600*	-2.84	12.12	0.007
AK048157	*AK048157*	-2.84	8.88	0.008
NM_011925	*Cd97*	-2.84	12.01	0.013
AK004064	*Dysfip1*	-2.85	11.97	0.023
NM_134195	*V1re6*	-2.85	10.53	0.014

NM_175433	*Zfp710*	-2.85	9.81	0.013
NM_031173	*Cacnb1*	-2.86	11.47	0.004
NM_008590	*Mest*	-2.86	13.82	0.011
XM_989061	*4930431J08Rik*	-2.86	10.17	0.021
NM_012020	*Foxl2*	-2.86	10.20	0.006
NM_026530	*E130307M08Rik*	-2.87	13.42	0.045
NM_010858	*Myl4*	-2.87	11.49	0.007
NM_023449	*Slc9a3r2*	-2.88	13.70	0.012
NM_022305	*B4galt1*	-2.88	13.45	0.000
AK041235	*Igh-4*	-2.88	11.03	0.018
NM_175474	*A230106M15Rik*	-2.88	14.45	0.009
NM_021715	*Chst7*	-2.88	10.12	0.013
XM_001001707	*D630033O11Rik*	-2.88	10.13	0.047
NM_009926	*Col11a2*	-2.89	17.36	0.002
NM_007796	*Ctla2a*	-2.89	10.17	0.004
NAP065826-1	*NAP065826-1*	-2.89	10.87	0.003
AK048085	*AK048085*	-2.89	14.36	0.011
XM_984911	*LOC666606*	-2.89	12.41	0.008
NM_182991	*5330410G16Rik*	-2.89	10.94	0.001
AB070542	*AB070542*	-2.89	10.67	0.002
BC011139	*Gck*	-2.90	11.13	0.005
NM_009596	*Abpa*	-2.90	9.70	0.041
NAP057192-1	*NAP057192-1*	-2.90	10.84	0.014
NM_029621	*2410004L22Rik*	-2.90	14.00	0.002
AK053575	*2900027M19Rik*	-2.91	10.68	0.005
NM_007433	*Akp5*	-2.91	10.88	0.004
NP063118	*NP063118*	-2.92	11.64	0.012
NM_146389	*Olfr1350*	-2.92	10.80	0.004
NM_172631	*D18Ertd653e*	-2.92	11.69	0.023
AK077214	*4933433K01Rik*	-2.93	10.95	0.013
NM_007967	*Evx2*	-2.93	11.09	0.011
AK132388	*4930563E22Rik*	-2.93	10.73	0.008
XM_140800	*Dhtkd1*	-2.93	10.28	0.010
NM_183097	*1700067K01Rik*	-2.94	10.82	0.001
NM_153133	*Rdh9*	-2.94	10.59	0.003
NM_172614	*Tmem44*	-2.94	10.34	0.011
NM_007989	*Foxh1*	-2.94	13.81	0.001
NM_026793	*Myct1*	-2.94	10.41	0.009
NM_030266	*Inpp4a*	-2.94	10.79	0.014
BC094937	*Rhoh*	-2.94	9.16	0.010
NM_147080	*Olfr615*	-2.94	10.71	0.010
NM_019540	*Pfpl*	-2.94	10.07	0.005
NM_010715	*Lig1*	-2.94	12.77	0.005
NM_145636	*Il27*	-2.95	11.72	0.010
NAP062461-1	*NAP062461-1*	-2.95	11.12	0.012
NM_020018	*Magea5*	-2.96	10.39	0.001
NM_028968	*Ifitm7*	-2.96	11.80	0.003
NM_019546	*Prodh2*	-2.97	11.02	0.000
NM_021355	*Fmod*	-2.97	12.30	0.028
NM_178421	*Nanos1*	-2.97	10.73	0.048
NM_177383	*Gpr21*	-2.97	11.90	0.015
NM_033509	*Vangl2*	-2.98	11.07	0.033
NAP103733-1	*NAP103733-1*	-2.98	10.81	0.001
NM_011985	*Mmp23*	-2.98	11.37	0.003
AK122561	*Wdr22*	-2.98	11.43	0.019
BY707292	*1700116B05Rik*	-2.99	10.50	0.009
NM_010491	*Iapp*	-2.99	11.03	0.006
NM_134011	*Tbrg4*	-2.99	11.16	0.007
NM_172397	*Limd2*	-2.99	16.70	0.005

AV147182	*AV147182*	-2.99	11.21	0.007
NM_175319	*C330005M16Rik*	-3.00	10.65	0.009
NAP026409-1	*NAP026409-1*	-3.00	11.09	0.007
BC055360	*BC055360*	-3.00	9.91	0.005
NM_019972	*Sort1*	-3.01	11.66	0.006
NM_026964	*1810023B24Rik*	-3.01	14.26	0.048
NM_011937	*Gnpda1*	-3.01	11.23	0.002
AK005616	*1700041C02Rik*	-3.02	12.19	0.004
NM_010097	*Sparcl1*	-3.02	12.69	0.023
NM_146272	*Olfr930*	-3.03	11.76	0.022
AK041395	*AK041395*	-3.03	10.40	0.041
NM_026142	*3632451O06Rik*	-3.03	13.05	0.002
NM_009936	*Col9a3*	-3.03	15.12	0.017
NM_007745	*Cort*	-3.03	11.60	0.009
NM_027406	*Aldh1l1*	-3.03	10.77	0.019
AK042863	*Phf21b*	-3.04	10.87	0.007
NM_146572	*Olfr1009*	-3.05	10.71	0.005
NM_007511	*Atp7b*	-3.05	11.81	0.013
NM_001002268	*Gpr126*	-3.05	13.06	0.008
NM_146606	*Olfr24*	-3.05	11.57	0.017
NM_023179	*Atp6v1g2*	-3.06	11.57	0.005
NM_146079	*Guca1b*	-3.06	12.62	0.001
NAP052224-1	*NAP052224-1*	-3.06	10.78	0.003
NM_026601	*Hyi*	-3.06	15.60	0.046
NM_013680	*Syn1*	-3.06	13.06	0.010
NM_026523	*Nmb*	-3.06	11.44	0.003
NM_016981	*Slc9a1*	-3.06	12.22	0.039
NM_027010	*Crygf*	-3.06	10.33	0.004
NM_153804	*Plekhg3*	-3.06	14.44	0.011
AK084225	*AK084225*	-3.07	10.95	0.010
NM_009696	*Apoe*	-3.07	12.57	0.011
NM_175347	*Srl*	-3.07	10.77	0.019
BC059866	*Shc2*	-3.07	12.82	0.005
NM_176902	*1110014K08Rik*	-3.08	16.08	0.018
NM_010169	*F2r*	-3.08	10.91	0.010
NM_001014995	*1110013L07Rik*	-3.09	11.24	0.010
BC086653	*Lba1*	-3.09	10.86	0.008
NM_174996	*D4Ertd22e*	-3.09	12.85	0.007
XM_146937	*LOC244911*	-3.09	11.02	0.008
NM_145487	*Prr3*	-3.09	10.98	0.001
AK006670	*1700041E20Rik*	-3.10	10.77	0.011
NM_013503	*Drd5*	-3.10	10.10	0.015
BC066804	*Il17rd*	-3.10	11.63	0.002
NM_181390	*Mustn1*	-3.10	11.97	0.006
NM_021306	*Ecel1*	-3.11	11.26	0.003
NM_026436	*Tmem86a*	-3.12	10.71	0.024
NM_007913	*Egr1*	-3.12	13.45	0.008
NM_011808	*Ets1*	-3.12	12.41	0.025
NM_146377	*Olfr127*	-3.12	10.35	0.002
NM_011597	*Tjp2*	-3.13	11.06	0.005
NM_146908	*Olfr1280*	-3.13	10.72	0.002
NM_198612	*BC049816*	-3.13	12.24	0.011
NM_177922	*Mapk15*	-3.13	10.76	0.002
NM_019928	*Klk4*	-3.14	11.21	0.005
NM_183249	*1100001G20Rik*	-3.14	9.99	0.045
NM_010158	*Khdrbs3*	-3.14	13.10	0.005
NM_027102	*Esam1*	-3.14	11.17	0.006
NM_147054	*Olfr584*	-3.15	9.65	0.004
NM_013836	*Tcf20*	-3.15	10.95	0.005

NM_027170	*2310057N15Rik*	-3.15	9.93	0.032
NM_009920	*Cnih2*	-3.15	10.83	0.009
NM_001011820	*Olfr1359*	-3.16	10.50	0.003
NM_029661	*1700027D21Rik*	-3.16	10.96	0.038
NM_010069	*Doc2a*	-3.16	11.12	0.010
NM_011476	*Sprr2j*	-3.16	11.53	0.002
NM_007428	*Agt*	-3.17	12.58	0.005
NM_008792	*Pcsk2*	-3.17	10.48	0.008
AK076954	*AK076954*	-3.17	10.52	0.001
NM_146183	*Zfp428*	-3.18	12.61	0.038
NM_007965	*Evl*	-3.18	13.46	0.003
NM_001012326	*NM_001012326*	-3.18	10.99	0.005
NM_023117	*Cdc25b*	-3.18	11.59	0.012
NM_181856	*Tmc8*	-3.18	11.42	0.005
NM_176848	*Fbxo2*	-3.19	11.95	0.007
AK045558	*Ap1s2*	-3.19	12.94	0.046
NM_001013826	*LOC435391*	-3.19	10.18	0.005
NM_023625	*1300012G16Rik*	-3.20	14.13	0.005
NM_026772	*Cdc42ep2*	-3.20	11.88	0.022
NM_145828	*Xylt2*	-3.20	13.80	0.001
NM_008304	*Sdc2*	-3.20	13.31	0.000
NM_013533	*Gpr162*	-3.20	10.28	0.005
NM_001033141	*1110006O17Rik*	-3.20	10.67	0.006
NM_199200	*BC025575*	-3.21	11.83	0.012
NM_028443	*3110032G18Rik*	-3.21	15.73	0.004
NM_001039376	*Pde4dip*	-3.21	15.32	0.016
NM_201255	*Krt9*	-3.21	10.60	0.013
NM_181344	*C1rl*	-3.22	10.88	0.009
NM_011693	*Vcam1*	-3.22	10.92	0.005
NM_013660	*Sema4d*	-3.23	11.51	0.004
NM_023764	*Tollip*	-3.23	11.74	0.006
NM_145596	*Gatad2a*	-3.24	15.21	0.018
NM_010150	*Nr2f6*	-3.25	13.03	0.015
AK049634	*Ppil4*	-3.25	10.44	0.001
NM_011789	*Apc2*	-3.25	11.77	0.012
BC090989	*Hmgb1-rs17*	-3.25	12.06	0.039
NM_024449	*Sost*	-3.25	11.33	0.007
NM_183251	*1810020D17Rik*	-3.25	12.24	0.009
NM_133223	*Rac3*	-3.26	12.26	0.047
NM_016972	*Slc7a8*	-3.26	11.08	0.003
NM_001033278	*A630055G03Rik*	-3.26	11.17	0.008
NM_013521	*Fpr1*	-3.27	10.74	0.017
NM_007725	*Cnn2*	-3.27	12.50	0.000
NM_175414	*Tspan9*	-3.28	13.05	0.003
AK161480	*4933432I09Rik*	-3.28	11.59	0.005
NM_031159	*Apobec1*	-3.28	10.86	0.013
NM_009261	*Strbp*	-3.28	10.92	0.006
NM_175667	*Ankrd5*	-3.28	10.91	0.009
NM_026716	*Sycn*	-3.28	10.71	0.000
NM_007731	*Col13a1*	-3.28	12.48	0.003
AK007181	*Tmem165*	-3.28	11.50	0.006
NM_029640	*1810044A24Rik*	-3.29	12.92	0.033
NM_009506	*Vegfc*	-3.29	10.16	0.014
NM_010585	*Itpr1*	-3.29	12.11	0.005
NM_207022	*Tas2r118*	-3.29	10.66	0.009
NM_009686	*Apbb2*	-3.29	13.34	0.003
NM_011160	*Prkg1*	-3.29	12.34	0.014
NM_016707	*Bcl11a*	-3.29	10.91	0.014
NM_080448	*Srgap3*	-3.29	9.44	0.011

NAP045112-1	*NAP045112-1*	-3.30	11.30	0.016
NM_011520	*Sdc3*	-3.30	13.83	0.001
NM_025815	*Cpne8*	-3.30	15.18	0.003
NM_145823	*Pitpnc1*	-3.31	12.05	0.007
AK014677	*4833411C07Rik*	-3.31	11.39	0.006
NM_130865	*Dmbx1*	-3.31	11.30	0.020
AK162873	*Abhd1*	-3.32	11.54	0.047
NM_177632	*BC022623*	-3.32	12.59	0.008
NM_008245	*Hhex*	-3.32	9.98	0.032
NM_029780	*Raf1*	-3.32	16.85	0.005
NM_054043	*Msi2*	-3.33	13.77	0.001
NM_011161	*Mapk11*	-3.33	10.85	0.009
NM_010435	*Hira*	-3.34	11.30	0.003
NM_183208	*Rai17*	-3.34	12.95	0.017
NM_175486	*6430571L13Rik*	-3.36	9.92	0.016
NM_021491	*Smpd3*	-3.36	16.48	0.002
NM_181072	*Myo1e*	-3.36	14.39	0.001
NAP043344-1	*NAP043344-1*	-3.36	10.67	0.006
NM_007574	*C1qc*	-3.36	10.56	0.043
NM_009910	*Cxcr3*	-3.37	10.39	0.014
NM_019503	*Fxyd1*	-3.37	13.49	0.010
XM_620746	*Dscaml1*	-3.38	11.10	0.008
NM_019508	*Il17b*	-3.38	11.69	0.011
NM_144955	*Nkx6-1*	-3.39	11.11	0.004
NM_025620	*2210417D09Rik*	-3.39	13.83	0.018
NM_009856	*Cd83*	-3.39	10.70	0.007
NM_016762	*Matn2*	-3.39	13.31	0.005
AK173168	*BC067047*	-3.39	11.51	0.018
NM_007528	*Bcl6b*	-3.39	10.64	0.002
AK020957	*Nuak1*	-3.40	15.02	0.000
AF140220	*Ctnnd1*	-3.40	12.16	0.013
NM_028349	*Sass6*	-3.40	11.36	0.016
AK090157	*AK090157*	-3.40	12.44	0.049
AK152387	*Csnk1a1*	-3.41	13.16	0.007
NM_011896	*Spry1*	-3.41	10.62	0.000
NM_010090	*Dusp2*	-3.41	10.65	0.045
NM_139152	*Asb18*	-3.42	11.20	0.001
NM_198703	*Wnk1*	-3.42	11.14	0.012
NM_029803	*Ifi27*	-3.42	8.44	0.042
NM_026915	*Lyzl4*	-3.43	10.90	0.009
NM_026730	*Gpihbp1*	-3.43	12.91	0.002
NM_175660	*Hist1h2ab*	-3.43	9.27	0.006
NM_001039153	*LOC654470*	-3.44	12.91	0.003
NM_199473	*Col8a2*	-3.44	12.06	0.014
NM_001013384	*5832418A03Rik*	-3.44	11.79	0.041
NM_017377	*B4galt2*	-3.44	11.62	0.007
NM_133721	*Itga9*	-3.44	10.05	0.012
NM_201619	*Nr1h5*	-3.45	9.77	0.006
NM_028188	*Rusc1*	-3.45	12.28	0.004
X96703	*Lasp1*	-3.45	10.59	0.011
NM_009332	*Tcf3*	-3.45	14.93	0.011
NM_026596	*4930591A17Rik*	-3.45	11.26	0.005
AK040404	*Raver2*	-3.46	10.95	0.003
NM_009136	*Scrg1*	-3.46	16.80	0.004
NM_145830	*Ehmt2*	-3.46	13.93	0.003
NM_133649	*Slc12a6*	-3.46	13.43	0.000
BC050790	*1700021F07Rik*	-3.47	11.11	0.032
NM_008520	*Ltbp3*	-3.47	13.63	0.000
NM_015772	*Sall2*	-3.48	10.86	0.035

AK141756	*AK141756*	-3.49	11.27	0.005
AK083675	*2600005C20Rik*	-3.49	10.87	0.002
NM_027702	*4933421I07Rik*	-3.50	10.55	0.006
NM_007776	*Crygd*	-3.50	11.00	0.011
AK010990	*2510022D24Rik*	-3.51	12.11	0.039
NM_147075	*Olfr656*	-3.52	11.43	0.004
NM_009131	*Clec11a*	-3.53	13.58	0.006
NM_053088	*Ifitm5*	-3.53	11.29	0.006
XM_357746	*A230106M20Rik*	-3.54	10.70	0.017
NM_025622	*Lgals2*	-3.54	9.82	0.003
NM_009614	*Adam15*	-3.54	13.77	0.037
NM_175138	*Dnaic1*	-3.54	11.55	0.004
BC031595	*D2Bwg1335e*	-3.54	12.31	0.018
NM_008560	*Mc2r*	-3.55	11.10	0.006
AK041399	*Cep55*	-3.55	10.47	0.014
NM_022000	*Gnas*	-3.55	11.28	0.008
NM_024269	*Arl2bp*	-3.56	13.30	0.001
NM_008695	*Nid2*	-3.56	14.34	0.000
NM_026822	*Sprrl5*	-3.56	10.50	0.001
NM_009123	*Nkx1-2*	-3.57	11.90	0.008
NM_139206	*Centd3*	-3.57	10.78	0.000
NM_009755	*Bmp1*	-3.58	13.51	0.004
NM_019446	*Barhl1*	-3.58	11.95	0.006
NM_010180	*Fbln1*	-3.59	11.54	0.013
NM_010284	*Ghr*	-3.59	12.12	0.001
NM_008557	*Fxyd3*	-3.59	14.31	0.001
NM_145613	*C1qtnf5*	-3.60	11.79	0.009
BC058211	*Sfxn4*	-3.60	11.23	0.005
NM_001008533	*Adora1*	-3.60	11.09	0.009
NM_177813	*C630025C03*	-3.61	10.90	0.007
AK079811	*Galnt11*	-3.63	13.65	0.027
NM_147068	*Olfr166*	-3.63	11.07	0.003
NM_133832	*Rdh10*	-3.63	11.07	0.004
XM_001003960	*Pde11a*	-3.63	11.62	0.047
AI838745	*AI838745*	-3.64	10.79	0.026
NAP057184-1	*NAP057184-1*	-3.64	11.47	0.000
NM_008548	*Man1a*	-3.64	11.95	0.005
AK087910	*D130020L05Rik*	-3.66	12.35	0.005
NM_021417	*1110006O24Rik*	-3.66	12.20	0.005
NM_172145	*2610027C15Rik*	-3.67	13.93	0.000
NM_144898	*Msto1*	-3.67	13.80	0.014
NM_010795	*Mgat3*	-3.67	10.88	0.009
NM_030565	*BC004044*	-3.68	13.15	0.004
AK165186	*Mpped2*	-3.68	13.42	0.001
NM_008131	*Glul*	-3.69	12.60	0.029
BC062815	*Bfsp2*	-3.69	11.23	0.012
NM_022020	*Rbp7*	-3.71	10.47	0.008
NM_172892	*Slc13a4*	-3.71	12.23	0.001
NM_199022	*Shc4*	-3.71	11.20	0.003
NM_172758	*BC031853*	-3.71	12.65	0.026
NM_013565	*Itga3*	-3.71	14.38	0.038
NAP045501-1	*NAP045501-1*	-3.72	11.22	0.009
NM_010758	*Mag*	-3.72	12.87	0.003
NM_009199	*Slc1a1*	-3.74	12.55	0.005
AK146784	*Mark4*	-3.74	13.10	0.032
BC072651	*Heg1*	-3.74	11.60	0.005
NM_031402	*Crispld1*	-3.75	15.70	0.018
AK008094	*Igl-V1*	-3.76	11.61	0.001
NAP038286-1	*NAP038286-1*	-3.76	12.22	0.007

NM_001029890	*2700083E18Rik*	-3.76	10.95	0.012
NM_008677	*Ncf4*	-3.76	11.03	0.016
NM_145409	*Chtf18*	-3.77	12.14	0.028
NM_198164	*Cdc2l6*	-3.77	12.56	0.011
NAP052718-1	*NAP052718-1*	-3.77	9.52	0.003
NM_030244	*Ier5l*	-3.77	11.34	0.045
NM_029956	*Mmab*	-3.78	13.49	0.015
NM_010172	*F7*	-3.79	10.55	0.000
NM_146679	*Olfr1427*	-3.79	11.05	0.021
AK027913	*Mucdhl*	-3.79	12.00	0.004
NM_007630	*Ccnb2*	-3.79	12.06	0.042
NM_010742	*Ly6d*	-3.80	10.31	0.013
AK016817	*Gpr39*	-3.80	10.94	0.018
NM_008388	*Eif3s6*	-3.80	11.21	0.037
NM_008260	*Foxa3*	-3.80	12.90	0.026
NM_011310	*S100a3*	-3.81	11.90	0.007
NM_007662	*Cdh15*	-3.82	11.32	0.015
NM_012065	*Pde6g*	-3.82	13.05	0.038
NM_009423	*Traf4*	-3.82	13.80	0.002
NM_007689	*Chad*	-3.84	13.70	0.003
NM_011471	*Sprr2e*	-3.84	12.35	0.001
NM_009876	*Cdkn1c*	-3.85	16.98	0.000
NM_011906	*Gpr175*	-3.86	11.46	0.005
NM_020507	*Tob2*	-3.86	13.08	0.005
AK165145	*9230115E21Rik*	-3.87	11.41	0.038
AK154331	*9430020K01Rik*	-3.87	13.62	0.004
NM_009008	*Rac2*	-3.87	12.29	0.002
NM_207654	*Efna5*	-3.88	14.40	0.012
NM_144942	*Csad*	-3.88	12.28	0.024
NM_009387	*Tk1*	-3.88	11.00	0.010
NM_080595	*Emid1*	-3.89	11.52	0.044
NM_008791	*Pcp4*	-3.90	12.44	0.029
AK018612	*9130011L11Rik*	-3.90	12.67	0.016
NM_013519	*Foxc2*	-3.91	10.84	0.011
NAP025478-001	*NAP025478-001*	-3.91	12.83	0.009
XM_619639	*Tns1*	-3.91	13.39	0.002
M35662	*Csh1*	-3.92	11.65	0.004
NM_021278	*Tmsb4x*	-3.92	15.51	0.003
NM_153061	*Tiaf2*	-3.92	11.27	0.031
XM_356935	*LOC383229*	-3.92	10.90	0.000
NM_026820	*Ifitm1*	-3.93	11.71	0.000
NM_011356	*Frzb*	-3.93	15.24	0.006
AJ237585	*5730507H05Rik*	-3.93	10.44	0.004
BC025150	*H19*	-3.93	14.15	0.016
NM_007799	*Ctse*	-3.95	11.15	0.015
NM_153507	*Cpne2*	-3.95	12.70	0.019
NAP030873-1	*NAP030873-1*	-3.95	14.10	0.000
AF127140	*Fgfr4*	-3.95	11.77	0.008
NM_175638	*Wnk4*	-3.96	14.42	0.000
AK014999	*4921534H16Rik*	-3.97	11.78	0.001
NM_008619	*Mov10*	-3.98	11.67	0.011
NM_029576	*Rab1b*	-3.98	13.54	0.049
NM_134189	*Galnt10*	-3.98	15.31	0.028
NM_175116	*P2ry5*	-3.99	11.29	0.018
NM_021390	*Sall1*	-4.00	11.37	0.001
NM_010047	*Dgcr6*	-4.01	13.54	0.028
NM_008585	*Mep1a*	-4.02	11.30	0.001
NM_007856	*Dhcr7*	-4.02	13.48	0.006
NM_008381	*Inhbb*	-4.02	11.94	0.004

NM_033478	*Ly6g6d*	-4.03	11.61	0.006
NM_016765	*Ddah2*	-4.03	11.60	0.024
NM_145447	*BC011209*	-4.03	14.70	0.008
AK032589	*Zfhx2as*	-4.04	12.03	0.000
AK089586	*AK089586*	-4.05	11.23	0.003
NM_026931	*1810011O10Rik*	-4.05	11.32	0.007
U89424	*Unc93b1*	-4.06	11.57	0.005
BC013068	*Pcsk5*	-4.07	9.82	0.007
NM_013846	*Ror2*	-4.07	12.08	0.004
AK019803	*4930571C24Rik*	-4.08	11.79	0.000
NM_008728	*Npr3*	-4.08	11.73	0.003
NM_011337	*Ccl3*	-4.08	9.81	0.022
AK030594	*Ube2f*	-4.08	11.65	0.006
NM_054042	*Cd248*	-4.10	12.94	0.001
NM_008859	*Prkcq*	-4.10	11.25	0.009
NP431391	*NP431391*	-4.11	10.43	0.010
NM_146541	*Olfr1361*	-4.12	11.57	0.007
NM_013756	*Defb3*	-4.12	11.56	0.003
NM_134032	*Hoxb2*	-4.13	10.32	0.011
NM_133229	*Dscr6*	-4.14	11.59	0.006
AK038070	*Hivep3*	-4.14	13.47	0.004
NAP014233-001	*NAP014233-001*	-4.14	12.21	0.003
BB643606	*B230311B06Rik*	-4.14	10.79	0.006
NM_138672	*Stab1*	-4.14	10.98	0.002
NM_023844	*Jam2*	-4.15	14.62	0.009
NM_010784	*Mdk*	-4.17	14.72	0.000
NAP050776-1	*NAP050776-1*	-4.17	11.16	0.002
AK017327	*AK017327*	-4.19	11.20	0.011
NM_009345	*Dntt*	-4.20	10.97	0.039
NM_199059	*Trf3*	-4.22	11.17	0.012
AF114382	*Fhl3*	-4.22	11.97	0.006
NM_199307	*Ece1*	-4.24	12.61	0.001
NM_138595	*Gldc*	-4.25	13.47	0.009
NM_153170	*Slc36a2*	-4.25	10.98	0.014
NM_152808	*Slc44a2*	-4.28	15.59	0.000
NM_201374	*BC050196*	-4.28	11.15	0.000
NM_008546	*Mfap2*	-4.29	12.11	0.014
AK138072	*1190002F15Rik*	-4.30	12.04	0.024
NM_183252	*2610017I09Rik*	-4.30	12.82	0.010
NM_008495	*Lgals1*	-4.30	16.58	0.014
NM_010446	*Foxa2*	-4.31	13.58	0.001
AK086235	*Wdr45l*	-4.32	11.46	0.008
NM_146344	*Olfr1495*	-4.33	11.75	0.005
NM_177057	*E230015B07Rik*	-4.34	12.39	0.014
NM_009598	*Ace*	-4.35	11.48	0.000
NM_026754	*1110017I16Rik*	-4.35	15.77	0.009
NM_023824	*Paqr4*	-4.35	11.61	0.003
NM_146459	*Olfr1215*	-4.36	11.46	0.008
NM_025455	*Ccdc28b*	-4.37	12.43	0.033
L20961	*L20961*	-4.38	11.17	0.041
NM_146228	*Als2cl*	-4.39	11.53	0.001
NM_080435	*Adcy4*	-4.40	10.84	0.005
NM_010228	*Flt1*	-4.40	12.72	0.032
NM_178676	*Entpd3*	-4.41	10.91	0.018
NM_021281	*Ctss*	-4.41	11.49	0.004
AK035470	*AK035470*	-4.41	9.44	0.009
NM_207676	*Igsf4a*	-4.42	12.49	0.005
NM_011178	*Prtn3*	-4.42	11.56	0.001
NM_144836	*Slc17a2*	-4.43	11.66	0.002

AK076708	*4930417M19Rik*	-4.44	11.64	0.032
NM_029928	*Ptprb*	-4.45	10.73	0.044
AK009137	*Prei4*	-4.45	14.10	0.009
NAP098046-001	*NAP098046-001*	-4.48	11.67	0.032
NM_026066	*Cmtm5*	-4.50	11.15	0.042
NM_145435	*Pyy*	-4.51	10.75	0.006
AK087208	*Epas1*	-4.53	11.29	0.006
NM_145134	*Spsb4*	-4.53	11.22	0.016
NM_021474	*Efemp2*	-4.54	17.09	0.000
NM_007405	*Adcy6*	-4.55	13.92	0.030
NM_009898	*Coro1a*	-4.56	10.69	0.010
AK033097	*Lmo4*	-4.59	12.63	0.022
NM_010807	*Marcksl1*	-4.61	13.21	0.002
NM_177732	*Slc35d1*	-4.61	13.88	0.011
NM_172257	*Sidt2*	-4.61	15.53	0.000
NM_033590	*Pcdhga7*	-4.62	14.02	0.022
NM_010612	*Kdr*	-4.62	12.23	0.014
NM_175274	*Ttyh3*	-4.62	12.54	0.000
AK032804	*D5Ertd579e*	-4.62	14.73	0.012
NM_011046	*Furin*	-4.63	16.01	0.007
NM_025331	*Gng11*	-4.64	15.35	0.001
NM_023182	*Ctrl*	-4.64	11.37	0.018
NM_023275	*Rhoj*	-4.64	11.65	0.002
AK010524	*2410017I17Rik*	-4.66	13.69	0.016
NM_053078	*D0H4S114*	-4.66	11.09	0.003
NM_028454	*Tm7sf2*	-4.67	13.50	0.009
NM_012043	*Islr*	-4.68	12.54	0.007
NM_019394	*Mia1*	-4.68	17.32	0.001
NM_134216	*V1rh7*	-4.71	12.69	0.040
NM_145562	*9130213B05Rik*	-4.73	13.32	0.014
NM_025735	*Map1lc3a*	-4.74	13.06	0.006
NM_016885	*Emcn*	-4.76	12.17	0.013
NM_010280	*Gfra3*	-4.78	11.20	0.020
NM_139269	*Hrasls3*	-4.81	14.70	0.000
NM_008788	*Pcolce*	-4.82	15.46	0.004
NM_016862	*Vti1a*	-4.82	13.01	0.011
NM_010217	*Ctgf*	-4.82	17.14	0.003
AK040670	*Ccdc32*	-4.85	9.39	0.006
NM_011352	*Sema7a*	-4.86	11.64	0.034
NM_010744	*Tmed1*	-4.86	14.45	0.000
NM_013767	*Csnk1e*	-4.88	14.15	0.017
NM_145100	*Lypd1*	-4.88	10.82	0.015
S74567	*Maf*	-4.89	14.96	0.029
NM_013805	*Cldn5*	-4.90	12.22	0.002
NM_133903	*Spon2*	-4.91	16.61	0.000
NM_010658	*Mafb*	-4.94	14.06	0.012
AK090207	*Slc43a2*	-4.96	11.72	0.012
NM_178183	*Hist1h2ak*	-4.98	13.42	0.024
NM_010135	*Enah*	-5.01	14.46	0.004
NM_009113	*S100a13*	-5.01	15.54	0.022
NM_013869	*Tnfrsf19*	-5.04	10.97	0.001
NM_023910	*Tsc22d4*	-5.04	12.61	0.005
NM_010494	*Icam2*	-5.06	12.58	0.014
AF004109	*Aanat*	-5.07	11.66	0.003
BC090402	*MGC73635*	-5.07	15.39	0.011
NM_013691	*Thbs3*	-5.08	15.96	0.000
NM_027434	*2610304G08Rik*	-5.10	10.90	0.023
NM_026579	*D10Wsu102e*	-5.10	12.96	0.018
NM_025809	*Clec14a*	-5.13	12.13	0.010

NM_001003815	*Epb4.1l1*	-5.16	12.32	0.007
NM_029021	*4833422F24Rik*	-5.17	12.70	0.011
NM_018826	*Irx5*	-5.17	13.58	0.000
NM_021528	*Chst12*	-5.18	13.33	0.007
AK006273	*1700023F06Rik*	-5.19	12.17	0.003
NM_178929	*Kazald1*	-5.20	13.34	0.008
AK044163	*BC019561*	-5.25	12.04	0.000
NM_017372	*Lyzs*	-5.26	14.45	0.023
AK009327	*2310012P17Rik*	-5.28	11.94	0.029
XM_988017	*D930017K21Rik*	-5.29	12.56	0.000
NM_178444	*Egfl7*	-5.34	12.47	0.020
NM_020574	*Kcne3*	-5.35	11.16	0.004
NM_025891	*Smarcd3*	-5.36	11.72	0.007
NM_011526	*Tagln*	-5.38	13.35	0.002
AK164568	*Slc38a2*	-5.42	14.67	0.001
NM_175408	*Tmem139*	-5.54	16.34	0.001
NM_007484	*Rhoc*	-5.56	16.36	0.011
NM_025424	*Nenf*	-5.59	15.66	0.013
NM_013655	*Cxcl12*	-5.60	11.53	0.024
NM_024283	*1500015O10Rik*	-5.62	15.76	0.000
NM_028783	*Robo4*	-5.64	10.80	0.007
NM_011085	*Pik3r1*	-5.65	11.93	0.008
NM_008278	*Hpgd*	-5.65	13.22	0.009
NM_011581	*Thbs2*	-5.66	15.89	0.008
NM_021375	*Rhbg*	-5.68	12.39	0.005
NM_173047	*Cbr3*	-5.69	14.35	0.029
NM_175316	*Slco2b1*	-5.70	13.47	0.002
NM_178182	*Hist1h2ai*	-5.73	12.55	0.014
J05020	*Fcer1g*	-5.73	11.01	0.006
BC051474	*Rasgrp2*	-5.74	10.89	0.006
AK003674	*Cthrc1*	-5.74	12.64	0.026
NR_001579	*Terc*	-5.75	11.93	0.005
NM_022814	*Svep1*	-5.77	13.36	0.001
NM_032610	*Spnb4*	-5.79	13.84	0.005
X82786	*Mki67*	-5.82	12.24	0.010
NM_009689	*Birc5*	-5.82	13.50	0.006
NM_011662	*Tyrobp*	-5.85	12.77	0.007
NM_008535	*Lyl1*	-5.87	11.21	0.003
NM_134257	*Rgs3*	-5.87	13.38	0.001
NM_010917	*Nid1*	-5.88	14.16	0.000
NM_198616	*Ccdc85b*	-5.90	13.53	0.046
NM_026866	*Disp1*	-5.91	11.99	0.000
M18187	*Lst1*	-5.92	12.79	0.012
NM_011340	*Serpinf1*	-5.92	14.53	0.006
NM_009891	*Chat*	-5.94	13.56	0.003
NAP064912-1	*NAP064912-1*	-5.95	12.56	0.008
NM_173371	*H6pd*	-5.99	13.09	0.018
NM_023320	*Plekho1*	-6.00	14.80	0.003
NM_053080	*Aldh1a3*	-6.02	16.47	0.000
NM_010769	*Matn1*	-6.02	15.81	0.004
NM_009931	*Col4a1*	-6.06	13.10	0.001
AK013783	*Usp32*	-6.10	12.69	0.008
NM_009115	*S100b*	-6.13	17.36	0.019
NM_026268	*Dusp6*	-6.14	13.98	0.008
NM_015744	*Enpp2*	-6.22	14.69	0.001
NM_009382	*Thy1*	-6.25	11.11	0.016
NM_183161	*BC019537*	-6.25	11.18	0.045
XM_981889	*Col22a1*	-6.26	14.13	0.008
NM_013775	*Tcl1b2*	-6.36	12.46	0.000

NM_013706	*Cd52*	-6.41	11.69	0.009
NM_028807	*1200009I06Rik*	-6.44	12.74	0.008
NM_019467	*Aif1*	-6.48	10.30	0.025
NM_027063	*1700013G24Rik*	-6.48	12.73	0.002
NM_178192	*Hist1h4a*	-6.51	13.07	0.041
NM_013925	*Adat1*	-6.56	13.92	0.011
NM_009333	*Tcf7l2*	-6.57	15.06	0.000
NM_009180	*St6galnac2*	-6.63	11.33	0.043
NM_024237	*1600015H20Rik*	-6.64	13.92	0.001
NM_052824	*Fxyd2*	-6.71	12.75	0.001
NM_152804	*Plk2*	-6.93	13.25	0.000
NM_013599	*Mmp9*	-7.03	14.25	0.006
AK051569	*Hecw1*	-7.11	13.88	0.002
NM_172633	*Cbln2*	-7.13	12.78	0.000
NM_030744	*Ropn1*	-7.19	14.41	0.012
NM_133654	*Cd34*	-7.20	12.46	0.001
BC055439	*Myl9*	-7.21	12.01	0.001
NM_007866	*Dll3*	-7.22	12.57	0.016
XM_138240	*LOC238395*	-7.25	16.58	0.006
BC043115	*0610010D24Rik*	-7.30	15.72	0.003
NM_011254	*Rbp1*	-7.44	12.90	0.002
NM_009183	*St8sia4*	-7.45	12.54	0.024
NM_025378	*Ifitm3*	-7.48	12.94	0.005
NM_007606	*Car3*	-7.60	12.80	0.010
NM_153127	*Mmrn2*	-7.64	12.24	0.002
NM_172804	*Syt16*	-7.64	13.98	0.000
NM_175661	*Hist1h2af*	-7.66	13.58	0.003
NM_010313	*Gnb5*	-7.72	15.30	0.000
NM_019444	*Ramp2*	-7.73	12.02	0.010
NM_009368	*Tgfb3*	-7.75	14.61	0.000
NAP102845-1	*NAP102845-1*	-7.82	13.92	0.032
AK019581	*AK019581*	-7.82	12.67	0.008
NM_053219	*V1ra4*	-8.13	13.29	0.000
Z11981	*Pvt1*	-8.34	15.59	0.002
NM_025288	*Stfa3*	-8.51	10.97	0.030
AK043656	*Fgd4*	-8.72	13.68	0.027
AK041359	*Tbc1d2*	-8.79	13.78	0.004
NM_012022	*Ppnr*	-8.83	15.12	0.000
BC023356	*Sox10*	-9.02	14.36	0.006
AK014046	*Prr6*	-9.12	16.12	0.009
NM_144883	*5430407P10Rik*	-9.16	14.28	0.022
NM_010110	*Efnb1*	-9.20	14.96	0.024
NM_153525	*Tmem41b*	-9.48	16.18	0.000
BC021944	*2310040A07Rik*	-9.51	14.77	0.001
NM_025508	*Gmpr*	-10.35	15.34	0.001
BU920841	*BU920841*	-10.36	12.04	0.001
BG963265	*BG963265*	-10.36	16.01	0.002
NM_080555	*Ppap2b*	-10.44	14.16	0.004
NM_010740	*Cd93*	-10.58	13.09	0.000
NM_008597	*Mgp*	-10.59	16.31	0.000
NM_009653	*Alas2*	-10.72	12.12	0.005
BC020108	*Arsi*	-10.84	14.05	0.000
NM_146362	*Olfr1312*	-10.87	15.32	0.012
AK090147	*Ihh*	-11.73	15.81	0.000
NM_008218	*Hba-a1*	-12.18	15.61	0.003
NM_145837	*Il17d*	-12.32	15.97	0.000
NM_020258	*Slc37a2*	-13.71	12.64	0.000
AK077026	*Slc9a2*	-14.23	12.06	0.001
XM_485455	*Grrp1*	-14.90	12.71	0.000

NM_022029	*Nrgn*	-15.64	12.20	0.000
NM_008220	*Hbb-b1*	-16.30	16.45	0.001
NM_011987	*Pla2g10*	-27.02	14.83	0.000
