# Supplementary material for: Transcriptional Profiling of Chondrodysplasia Growth Plate Cartilage Reveals Adaptive ER-Stress Networks That Allow Survival but Disrupt Hypertrophy
Source: PLoS One. 2011 Sep 15;6(9):e24600. doi: 10.1371/journal.pone.0024600 (PMC3174197; doi:10.1371/journal.pone.0024600)
Supplement: Table S2 — Cog versus wildtype differentially expressed genes. (PDF) [file pone.0024600.s006.pdf]

Table S2 - Cog versus Wildtype Differentially Expressed Genes

GenBank Accession	GeneName	Fold Diff	A	adj.P.Val
NM_009375	*Tgn*	241.46	11.35	0.000
NM_024440	*Derl3*	54.65	13.73	0.000
NM_021395	*Hyou1*	30.29	14.22	0.000
NM_011631	*Grp94*	19.95	16.49	0.000
NM_022324	*Sdf2l1*	15.42	14.44	0.000
NM_007837	*Chop*	12.88	13.94	0.000
NM_181072	*Myo1e*	12.77	13.42	0.010
AK016003	*4930539N22Rik*	11.29	10.44	0.010
NM_029720	*Creld2*	10.18	17.30	0.000
NM_001033302	*Gm129*	9.96	11.74	0.000
NM_013584	*Lifr*	8.55	11.94	0.014
NM_028170	*1700030K09Rik*	8.44	10.90	0.042
NM_001033228	*Itga1*	8.35	11.64	0.020
NM_009787	*Erp72*	8.11	12.43	0.022
NM_010800	*Bhlhb8*	7.67	10.90	0.002
NM_172121	*Zc3h3*	7.54	10.98	0.029
NM_145564	*Fbxo21*	7.37	11.93	0.031
NM_031179	*Sf3b1*	7.12	10.43	0.008
AK051569	*Hecw1*	7.07	13.88	0.011
NM_001004153	*LOC245128*	6.93	11.59	0.015
AK148928	*P5*	6.80	11.89	0.001
NM_207683	*Pik3c2g*	6.76	11.38	0.007
AK035513	*Pdlim4*	6.71	11.20	0.011
NM_133669	*Rp2h*	6.67	13.47	0.006
AK079811	*Galnt11*	6.55	13.65	0.020
AK087234	*Ulk2*	6.38	15.81	0.001
NM_028799	*Tgm5*	6.35	11.67	0.019
NM_009802	*Car6*	6.30	13.14	0.000
NM_011081	*Piga*	6.20	11.51	0.000
AK028934	*AK028934*	6.17	11.09	0.050
NM_013565	*Itga3*	6.12	14.38	0.037
NM_010111	*Efnb2*	6.11	10.42	0.020
NM_172804	*Syt16*	6.09	13.98	0.004
AK041855	*AK041855*	6.07	13.80	0.022
AK018612	*9130011L11Rik*	6.02	12.67	0.020
BC051947	*2900083I11Rik*	6.01	12.71	0.017
NM_022331	*Herpud1*	5.79	16.66	0.000
NM_009291	*Stra6*	5.72	12.50	0.024
AK035674	*Cdcp1*	5.71	13.79	0.030
NM_144554	*Trib3*	5.68	14.84	0.000
AK089283	*Slc7a11*	5.68	10.43	0.006
XM_888686	*Atf6*	5.53	12.74	0.004
NM_146914	*Olfr5*	5.48	12.81	0.006
NM_138630	*Arhgap4*	5.44	11.34	0.011
AK010524	*2410017I17Rik*	5.44	13.69	0.040
NM_027399	*Steap1*	5.38	15.77	0.000
NM_008006	*Fgf2*	5.36	10.65	0.020
NM_144958	*Eif4a1*	5.34	13.61	0.019
NM_145993	*L3mbtl2*	5.33	11.77	0.015
NP377814	*NP377814*	5.29	10.04	0.014
NM_008929	*Dnajc3*	5.23	14.73	0.000
NM_026184	*Ero1lb*	5.16	10.47	0.003
C80049	*C80049*	5.16	14.29	0.049
NM_013760	*ERdj4*	5.13	12.90	0.003
XM_884648	*Trim55*	5.02	14.73	0.024

NM_022310	*BiP*	4.91	17.53	0.011
NM_172453	*AI449441*	4.85	11.63	0.008
AK122422	*Unc13b*	4.82	10.35	0.015
AK012966	*Mlstd2*	4.81	13.39	0.014
NM_001003815	*Epb4.1l1*	4.77	12.32	0.034
AK033905	*4732418C07Rik*	4.74	11.08	0.050
NM_011819	*Gdf15*	4.73	12.28	0.016
NM_172659	*Slc2a6*	4.67	10.94	0.029
NM_019539	*Cts7*	4.65	9.07	0.038
XM_993224	*LOC245436*	4.63	10.45	0.009
NM_032610	*Spnb4*	4.58	13.84	0.033
NM_175408	*Tmem139*	4.57	16.34	0.009
NM_026373	*Cdk2ap2*	4.57	13.88	0.001
NM_027373	*2600003E23Rik*	4.56	10.49	0.044
NM_175096	*D5Ertd593e*	4.53	12.16	0.032
AK014174	*Chka*	4.51	11.67	0.014
AK044363	*AK044363*	4.47	11.15	0.042
NM_007498	*Atf3*	4.45	14.53	0.038
AK089569	*AK089569*	4.41	10.87	0.010
NM_001037935	*LOC629678*	4.38	11.72	0.023
NM_027533	*Tspan2*	4.37	13.42	0.004
AK085279	*E230002P03Rik*	4.31	12.85	0.023
NM_009803	*Nr1i3*	4.28	11.07	0.028
NM_028264	*Tmem55a*	4.25	14.07	0.039
NM_029548	*Rph3al*	4.23	11.08	0.047
AK054491	*AK054491*	4.19	10.47	0.024
NM_016711	*Tmod2*	4.16	16.18	0.011
NM_008601	*Mitf*	4.14	10.16	0.043
XM_001004952	*9430008C03Rik*	4.11	13.41	0.028
NM_028228	*2610028A01Rik*	4.08	14.87	0.024
NM_053219	*V1ra4*	4.07	13.29	0.007
NM_145464	*Sox21*	4.06	11.35	0.038
AK051944	*AK051944*	4.03	10.23	0.010
NM_028769	*Syvn1*	4.03	16.29	0.003
NM_183208	*Rai17*	4.02	12.95	0.036
NM_013932	*Ddx25*	4.02	15.77	0.041
NM_027893	*Pvrl4*	4.01	11.28	0.041
AK007241	*1700122O11Rik*	3.98	11.27	0.046
NM_029634	*Ihpk2*	3.98	12.70	0.020
NM_023190	*Acin1*	3.96	11.18	0.040
NM_021323	*Usp29*	3.94	12.62	0.045
AK086528	*AK086528*	3.93	11.32	0.024
NM_026929	*Chac1*	3.90	16.09	0.020
NM_025472	*1810032O08Rik*	3.87	13.37	0.042
NM_013745	*Nufip1*	3.86	10.38	0.021
NM_029690	*Arpm1*	3.85	12.13	0.029
AK013613	*2900034E22Rik*	3.85	13.83	0.001
NM_153525	*Tmem41b*	3.83	16.18	0.006
AK017387	*5430433J05Rik*	3.80	12.91	0.030
AK035116	*E2f6*	3.79	9.29	0.010
AJ315551	*Ly6g6e*	3.79	10.73	0.031
XM_912173	*LOC636687*	3.74	14.04	0.034
NM_175329	*Ndg2*	3.68	13.82	0.017
NM_172428	*2310042L06Rik*	3.65	13.70	0.000
NM_198885	*Scx*	3.64	13.00	0.037
AK046472	*B230396O12Rik*	3.63	12.63	0.028
NM_001024468	*Bcat1*	3.63	14.46	0.001
AK006257	*1700023A16Rik*	3.60	12.58	0.028

NM_001039089	*Sel1h*	3.60	11.18	0.044
NM_146085	*Apbb3*	3.60	14.98	0.010
NM_031388	*Usp26*	3.59	10.17	0.009
NM_030018	*Tmem50b*	3.58	13.62	0.006
NM_207269	*D330050I23Rik*	3.56	13.53	0.011
NM_028057	*Cyb5r1*	3.54	14.48	0.004
NM_008860	*Prkcz*	3.54	11.45	0.020
NM_011716	*Wfs1*	3.53	15.01	0.004
NM_172641	*9930023K05Rik*	3.52	9.92	0.022
NM_007905	*Phc1*	3.48	12.36	0.020
NM_001008427	*LOC434179*	3.47	16.81	0.025
AK041235	*Igh-4*	3.47	11.03	0.034
AK083254	*Diap2*	3.45	12.05	0.029
AK044196	*AK044196*	3.44	9.09	0.009
NM_146899	*Olfr1219*	3.44	12.63	0.048
NM_007757	*Cpox*	3.44	14.13	0.000
NM_020013	*Fgf21*	3.42	11.87	0.013
NM_028325	*Zcchc12*	3.42	10.54	0.009
NM_013819	*H2-M3*	3.41	10.49	0.029
NM_019819	*Dusp14*	3.40	14.07	0.037
AK090131	*D330001F17Rik*	3.39	14.42	0.037
NM_011464	*Spint2*	3.39	10.91	0.034
NM_146293	*Olfr1143*	3.38	11.31	0.030
XM_284376	*Cd209g*	3.37	10.68	0.050
AK035387	*9530028C05*	3.36	16.08	0.021
NM_008358	*Il15ra*	3.35	12.26	0.029
NM_010313	*Gnb5*	3.34	15.30	0.021
AK037972	*AK037972*	3.34	10.78	0.042
NM_145433	*Mrm1*	3.33	11.30	0.038
NM_028980	*8430415E04Rik*	3.33	10.33	0.015
NM_008778	*Pak3*	3.32	10.05	0.048
NM_178595	*Ptrh1*	3.31	13.59	0.005
NM_026400	*ERdj3*	3.31	16.47	0.004
NM_152813	*Plcd3*	3.30	11.53	0.023
NM_010001	*Cyp2c37*	3.29	11.79	0.050
XM_143750	*Gabbr2*	3.28	10.13	0.040
NM_153528	*Gramd1c*	3.25	9.95	0.030
NM_177002	*C730048C13Rik*	3.24	10.17	0.028
NM_007850	*Defcr3*	3.23	12.60	0.050
NM_019448	*Dnmt3l*	3.23	11.74	0.048
XM_001002633	*Wdr81*	3.22	14.87	0.014
NM_011178	*Prtn3*	3.20	11.56	0.012
NM_010918	*Nktr*	3.19	13.56	0.008
NM_001039376	*Pde4dip*	3.19	10.48	0.042
AK084144	*AK084144*	3.15	9.17	0.010
NM_026989	*Sfrs11*	3.15	15.41	0.009
NM_001033261	*Psrc2*	3.15	10.37	0.017
NM_011710	*Wars*	3.09	13.51	0.009
AK012623	*2700099C18Rik*	3.08	9.24	0.009
NM_029103	*Armet*	3.08	17.28	0.012
NM_134105	*Txndc11*	3.07	13.58	0.009
NM_178364	*Zfp369*	3.05	11.04	0.019
NM_001037744	*Timm8a2*	3.05	9.42	0.016
NM_007515	*Slc7a3*	3.05	14.10	0.042
NM_008654	*Myd116*	3.04	10.20	0.006
NM_025626	*3110001A13Rik*	3.04	9.28	0.016
AK020958	*Cacng2*	3.02	10.18	0.011
NM_198629	*LOC328644*	3.02	9.65	0.040

AK086294	*Tcfcp2l1*	3.01	10.87	0.009
XM_125867	*Utp20*	3.01	9.45	0.009
NM_007952	*Erp57*	3.00	17.25	0.019
NM_028628	*1110008K04Rik*	2.99	11.80	0.020
NM_029733	*2010005H15Rik*	2.98	17.19	0.016
AK037524	*AK037524*	2.95	9.45	0.041
NM_009883	*Cebpb*	2.95	15.47	0.003
AK033989	*AK033989*	2.94	10.12	0.020
AK085074	*AK085074*	2.94	10.01	0.004
BC078452	*Rbm15b*	2.93	11.91	0.034
BU053129	*BU053129*	2.93	10.22	0.014
NM_025383	*Necap2*	2.93	10.95	0.041
AK145542	*Ndp52*	2.92	10.88	0.020
CA495516	*CA495516*	2.91	11.06	0.003
AK028648	*Arhgef10l*	2.90	10.68	0.018
NM_181584	*Gab3*	2.88	10.14	0.037
BC082308	*Mfhas1*	2.85	13.46	0.040
NM_138758	*Tmlhe*	2.84	13.17	0.046
AF221104	*Kifc5c*	2.84	10.76	0.001
NM_153133	*Rdh9*	2.83	10.59	0.015
NM_134158	*Cd300d*	2.83	9.99	0.020
NM_026822	*Sprrl5*	2.82	10.50	0.017
XM_983620	*6720475J19Rik*	2.82	10.47	0.019
NM_023363	*2810426N06Rik*	2.82	9.82	0.025
NM_026286	*Ftmt*	2.82	10.97	0.028
X98368	*Sox19*	2.82	10.52	0.020
NM_054038	*Scgb3a2*	2.82	12.02	0.044
NM_029587	*1700012A03Rik*	2.81	10.84	0.012
NM_133692	*Pold3*	2.81	11.00	0.041
NM_010425	*Foxd3*	2.80	9.45	0.031
NM_009893	*Chrd*	2.79	11.64	0.035
NM_172713	*Sdad1*	2.76	13.58	0.045
AK043285	*A730081D07Rik*	2.76	12.29	0.020
NM_029770	*Unc5b*	2.76	16.32	0.023
NM_001025102	*2700007P21Rik*	2.75	15.19	0.020
NM_026095	*Snrpd3*	2.74	15.17	0.049
XR_004813	*LOC675674*	2.70	9.92	0.011
BG082943	*BG082943*	2.70	10.15	0.010
XM_150216	*4930422I07Rik*	2.70	10.84	0.007
AK047530	*Usp33*	2.70	11.12	0.019
NM_175162	*Stox2*	2.69	10.92	0.028
NM_201374	*BC050196*	2.69	11.15	0.016
M18209	*Tcea1*	2.68	11.12	0.038
NM_030720	*Gpr84*	2.67	11.47	0.049
BC040823	*Lsm14b*	2.66	12.79	0.048
NM_009701	*Aqp5*	2.65	10.50	0.021
NM_138311	*H1foo*	2.65	10.82	0.009
NM_009913	*Ccr9*	2.65	11.77	0.034
XM_110968	*Dnahc9*	2.65	9.56	0.032
NM_016752	*Slc35b1*	2.65	16.98	0.012
NM_008795	*Pctk3*	2.64	11.71	0.001
X67128	*Tcrb-V13*	2.64	9.46	0.006
NM_026775	*Tmed10*	2.64	14.64	0.020
AK017531	*Herc3*	2.64	11.58	0.011
NM_010324	*Got1*	2.63	14.47	0.015
NM_145477	*Alg12*	2.63	10.91	0.049
NM_028848	*Spata17*	2.63	9.28	0.017
BC055360	*BC055360*	2.62	9.91	0.036

AK017440	*Rian*	2.62	9.29	0.009
NM_016721	*Iqgap1*	2.62	14.47	0.004
NM_025903	*Ifrd2*	2.62	12.22	0.044
NM_133801	*Gtf2f1*	2.62	16.10	0.009
NM_010517	*Igfbp4*	2.61	10.07	0.022
BC062197	*Sirpa*	2.61	15.00	0.031
AK053617	*E130114P18Rik*	2.61	9.69	0.020
NM_173867	*Rcc2*	2.61	14.02	0.008
XM_619361	*LOC545136*	2.60	9.67	0.048
AK028221	*AK028221*	2.59	7.19	0.030
NM_028231	*Kcnmb2*	2.59	9.68	0.019
NM_022024	*Gmfg*	2.59	10.23	0.020
NM_177204	*D330017J20Rik*	2.58	10.12	0.017
XM_205469	*Dmrtb1*	2.57	10.50	0.032
NM_053185	*Col4a6*	2.57	12.07	0.017
NM_029101	*1110014J01Rik*	2.56	11.79	0.006
NM_001033172	*Rab11fip2*	2.56	11.54	0.023
NM_021890	*Fads3*	2.56	12.94	0.005
NM_025445	*Arfgap3*	2.55	14.09	0.001
NM_007791	*Csrp1*	2.54	11.93	0.005
NM_001024708	*LOC436177*	2.53	12.73	0.021
NM_013775	*Tcl1b2*	2.52	12.46	0.050
AK016515	*Myo18b*	2.51	10.17	0.026
NM_008911	*Ppox*	2.51	10.82	0.022
NM_027423	*Polr3b*	2.51	13.01	0.043
BC052715	*Lars*	2.51	16.52	0.002
NM_028195	*Pscd4*	2.50	12.13	0.032
NM_016966	*Phgdh*	2.50	14.83	0.037
NM_026485	*Trabd*	2.49	13.75	0.032
NM_024246	*Tmem79*	2.47	12.07	0.024
NM_011617	*Tnfsf7*	2.46	10.34	0.012
NM_022015	*Tbn*	2.46	12.45	0.034
XM_207492	*LOC280121*	2.45	16.82	0.048
AK036419	*F730031O20Rik*	2.44	10.93	0.017
NM_011263	*Rest*	2.44	9.17	0.031
NM_008591	*Met*	2.44	10.04	0.012
NM_053176	*Hrg*	2.44	10.64	0.030
NM_146828	*Olfr975*	2.44	10.78	0.032
NM_176951	*Xkr5*	2.43	10.27	0.006
NM_172572	*Rhbdf2*	2.43	11.69	0.041
NM_024250	*Phf10*	2.42	16.76	0.049
AK084851	*D430001L07Rik*	2.40	17.64	0.024
NM_177027	*Zcchc7*	2.40	13.22	0.024
NM_009699	*Aqp2*	2.40	12.01	0.041
NM_008153	*Cmklr1*	2.39	10.99	0.034
NM_025587	*Rps21*	2.39	16.34	0.038
NM_080457	*Muc4*	2.37	9.35	0.050
AK172246	*Ipmk*	2.36	8.85	0.040
NM_027696	*Mier1*	2.36	10.61	0.030
NM_175137	*Vars2l*	2.36	10.46	0.014
NM_031499	*Prp2*	2.35	11.75	0.042
BC037635	*Ube2i*	2.34	10.65	0.007
NM_023784	*Yipf7*	2.34	10.13	0.040
XM_911929	*1110008I14Rik*	2.33	10.62	0.022
AK013390	*2810468N07Rik*	2.33	10.72	0.038
AK052843	*AK052843*	2.33	10.70	0.019
NM_172552	*Tdg*	2.33	14.65	0.047
NM_008057	*Fzd7*	2.33	9.65	0.009

NM_172856	*Lass6*	2.33	9.45	0.030
NM_011867	*Slc26a4*	2.32	10.16	0.025
AK030258	*4930556M19Rik*	2.32	10.22	0.004
S63763	*Ptpn6*	2.32	9.89	0.036
NM_026248	*4930430A15Rik*	2.32	9.38	0.008
NM_025454	*Ing5*	2.31	11.64	0.049
AK077979	*Fdps*	2.31	12.05	0.045
AK038962	*Mamdc1*	2.31	11.77	0.038
NM_029629	*Fahd2a*	2.31	9.22	0.008
NM_009235	*Sox15*	2.30	15.10	0.043
NM_139152	*Asb18*	2.29	11.20	0.037
AK144901	*1110014K08Rik*	2.29	15.33	0.005
NM_146487	*Olfr130*	2.28	10.13	0.011
NM_153067	*Mrgpra3*	2.26	10.09	0.050
NM_020598	*Olfr17*	2.26	10.43	0.019
NM_007989	*Foxh1*	2.26	13.81	0.015
NM_008577	*Slc3a2*	2.25	16.97	0.011
NM_008269	*Hoxb6*	2.25	10.47	0.040
NM_009058	*Ralgds*	2.24	11.80	0.050
NM_199021	*Dpp10*	2.24	10.37	0.043
NM_177158	*5830482F20Rik*	2.24	10.73	0.020
NM_029671	*1700034O15Rik*	2.24	11.46	0.017
NM_019998	*Alg2*	2.23	12.42	0.014
NM_146034	*Mgea6*	2.23	11.47	0.044
BC066804	*Il17rd*	2.23	11.63	0.035
NM_013661	*Sema5b*	2.22	12.50	0.020
NM_024231	*Zfpl1*	2.22	15.53	0.041
NM_001039373	*Mtcp1*	2.22	9.97	0.004
NM_011610	*Tnfrsf1b*	2.22	10.91	0.040
NM_145394	*Slc44a3*	2.21	9.05	0.007
NM_007591	*Calr*	2.20	18.11	0.006
NM_018868	*Nol5*	2.18	14.60	0.015
NM_011601	*Tlm*	2.18	12.92	0.048
XM_484069	*XM_484069*	2.17	16.95	0.030
AK051632	*D130061D10Rik*	2.17	9.86	0.031
CA494461	*Mrpl52*	2.17	17.52	0.040
NM_013622	*Oprd1*	2.17	8.86	0.020
AK032396	*1810015A11Rik*	2.16	8.70	0.048
NM_053008	*Olig3*	2.16	10.85	0.030
NM_009988	*Cxadr*	2.16	9.15	0.021
NM_183223	*BC107364*	2.16	8.66	0.035
AK134636	*2610005L07Rik*	2.15	11.49	0.027
NM_173755	*Ube2o*	2.15	12.65	0.037
AK014999	*4921534H16Rik*	2.15	11.78	0.048
NM_001018086	*LOC245297*	2.15	12.24	0.048
NM_013829	*Plcb4*	2.15	9.53	0.033
NM_008686	*Nfe2l1*	2.14	16.78	0.010
AK133661	*Lrrfip1*	2.14	14.17	0.018
NM_001025612	*Snx22*	2.14	11.59	0.032
AK040310	*Rsbn1l*	2.14	12.02	0.048
NM_028533	*1700065D16Rik*	2.13	10.18	0.017
NM_054040	*Tulp4*	2.12	10.68	0.033
AK017340	*AK017340*	2.11	9.62	0.047
NM_019993	*Aldh9a1*	2.11	13.34	0.021
NM_139307	*Vasn*	2.11	12.76	0.017
AK081302	*AK081302*	2.11	9.91	0.018
NM_001008424	*Cdsn*	2.11	12.08	0.041
S79463	*Sema4c*	2.10	10.61	0.020

NM_153518	*Ccdc65*	2.10	10.60	0.003
AK051244	*AK051244*	2.10	8.92	0.017
AK077029	*4921528I07Rik*	2.09	8.03	0.020
NM_021421	*Angel2*	2.09	12.90	0.020
NM_028874	*Snx19*	2.08	11.44	0.033
NM_175520	*Gpr81*	2.08	9.43	0.002
NM_011826	*Hax1*	2.08	16.00	0.012
AK083496	*Ccni*	2.07	9.85	0.045
XM_621686	*LOC231591*	2.07	10.17	0.029
NM_019632	*Napb*	2.07	11.40	0.011
AK220252	*9130229H14Rik*	2.07	14.68	0.040
AK011905	*Ncam1*	2.07	9.70	0.028
AK029875	*AK029875*	2.06	9.08	0.019
NM_033562	*Derl2*	2.06	12.52	0.019
NM_001040005	*D11Ertd759e*	2.05	12.31	0.036
NM_146640	*Olfr1153*	2.05	10.71	0.045
CO796036	*CO796036*	2.05	11.15	0.028
AK032589	*Zfhx2as*	2.04	12.03	0.049
AK002217	*0610005C13Rik*	2.04	10.45	0.046
NM_016748	*Ctps*	2.03	13.39	0.030
XR_002787	*LOC624863*	2.03	17.08	0.006
NM_199322	*Dot1l*	2.03	12.61	0.047
XM_619795	*Ryr3*	2.02	10.63	0.041
NM_011652	*Ttn*	2.01	9.16	0.048
NM_008236	*Hes2*	2.01	10.07	0.041
NM_032002	*Nrg4*	2.01	10.99	0.041
AK038350	*AK038350*	2.00	8.55	0.019
NM_199148	*BC051665*	2.00	10.72	0.041
AK046757	*AK046757*	2.00	9.93	0.019
NM_027568	*4733401H21Rik*	2.00	10.46	0.019
AK140530	*Plcl1*	-2.00	9.88	0.049
NM_011351	*Sema6c*	-2.01	11.09	0.049
NM_027026	*Lrrc46*	-2.01	9.67	0.045
NM_029091	*Klc4*	-2.01	12.61	0.043
NM_020003	*0610031J06Rik*	-2.02	13.18	0.004
NM_178061	*Mobkl2b*	-2.02	10.39	0.019
NM_010077	*Drd2*	-2.02	10.48	0.014
NM_020258	*Slc37a2*	-2.02	12.64	0.041
NM_177394	*A730011L01Rik*	-2.02	11.51	0.017
NM_001033225	*Pnrc1*	-2.03	15.94	0.033
AK017027	*Rnf148*	-2.03	9.91	0.020
NM_145388	*Vmd2l1*	-2.03	11.17	0.020
NM_011417	*Smarca4*	-2.03	13.58	0.005
NM_027251	*2010107G23Rik*	-2.04	9.90	0.048
NM_177682	*AU022870*	-2.04	13.75	0.020
NM_008624	*Mras*	-2.05	12.20	0.040
NM_175123	*1110051M20Rik*	-2.05	10.58	0.010
NM_024243	*Fuca1*	-2.05	12.67	0.036
NM_144832	*BC017643*	-2.06	12.16	0.030
NM_001009549	*Zfp36l3*	-2.06	9.06	0.041
NM_029420	*Giyd2*	-2.07	12.10	0.018
NM_011513	*Surf5*	-2.08	12.75	0.045
NM_023258	*Pycard*	-2.09	11.46	0.030
NM_016921	*Tcirg1*	-2.10	11.27	0.035
NM_138306	*Dgkz*	-2.10	10.56	0.013
BC008259	*Immp1l*	-2.11	15.43	0.020
AK039608	*A430035B10Rik*	-2.11	10.78	0.014
NM_013782	*Ptdss2*	-2.12	10.99	0.039

NM_011924	*Avpr1b*	-2.13	9.79	0.036
NM_008916	*RP23-136K12.4*	-2.15	11.50	0.039
NM_199455	*1700055M20Rik*	-2.15	10.80	0.033
NM_018773	*Scap2*	-2.16	13.98	0.025
NM_025858	*Scotin*	-2.16	14.23	0.034
NM_008492	*Ldhb*	-2.17	13.46	0.015
AK052458	*Depdc5*	-2.17	10.97	0.015
AK081787	*5730601F06Rik*	-2.17	10.09	0.028
NM_176893	*Mink1*	-2.18	11.01	0.028
NM_011808	*Ets1*	-2.18	9.84	0.039
NM_026142	*3632451O06Rik*	-2.18	13.05	0.034
NM_028659	*Eif3s12*	-2.18	11.26	0.018
NM_177330	*Ghsr*	-2.19	10.15	0.043
NM_145608	*BC021891*	-2.19	11.29	0.045
NM_025473	*1810037C20Rik*	-2.19	12.08	0.034
NM_009376	*Ift88*	-2.20	10.34	0.046
NM_013743	*Pdk4*	-2.21	11.65	0.031
NM_133903	*Spon2*	-2.22	13.68	0.041
AJ001373	*Itgb1bp1*	-2.22	12.59	0.049
AK006574	*1700031A10Rik*	-2.23	10.24	0.034
NM_028544	*Rasip1*	-2.24	10.37	0.020
NM_177266	*Gfm2*	-2.24	10.43	0.021
NM_001039167	*D11Bwg0517e*	-2.26	10.78	0.024
BC050924	*Zmym4*	-2.27	10.19	0.012
NM_008369	*Il3ra*	-2.28	12.33	0.023
NM_147004	*Olfr399*	-2.30	10.18	0.047
XM_906012	*LOC236622*	-2.30	11.63	0.047
NM_173370	*Cds1*	-2.30	11.48	0.036
AK042403	*AK042403*	-2.31	9.62	0.040
NM_025573	*Sfrs9*	-2.31	13.99	0.048
NM_020512	*Olfr1507*	-2.31	9.87	0.022
NM_011699	*Lin7c*	-2.31	11.45	0.038
NM_007872	*Dnmt3a*	-2.33	9.40	0.043
NM_145478	*Pim3*	-2.34	10.76	0.005
NM_177301	*Hnrpl*	-2.35	13.78	0.021
NM_001013392	*Rreb1*	-2.35	10.29	0.039
NM_026866	*Disp1*	-2.36	11.99	0.034
NM_016977	*Mc4r*	-2.39	10.69	0.039
XM_899121	*Camsap1l1*	-2.42	11.64	0.020
NM_026730	*Gpihbp1*	-2.42	12.91	0.040
NM_025350	*Cpa1*	-2.42	9.90	0.032
NM_009557	*Zfp46*	-2.43	13.53	0.041
AK050799	*Pank1*	-2.44	9.99	0.029
NM_007478	*Arf3*	-2.45	12.10	0.033
NM_001033199	*AI747448*	-2.46	9.14	0.030
NM_175074	*Hmgn3*	-2.46	11.10	0.045
NM_008294	*Hsd3b4*	-2.46	11.03	0.016
NM_178252	*Snx26*	-2.47	11.12	0.015
NM_010421	*Hexa*	-2.49	15.23	0.041
NM_175752	*Chn1*	-2.52	9.46	0.050
NM_146286	*Olfr981*	-2.53	8.50	0.032
NM_172442	*Dtx4*	-2.53	9.56	0.012
NM_207175	*Olfr239*	-2.53	10.43	0.017
NM_011520	*Sdc3*	-2.54	12.95	0.010
NM_198108	*BC023055*	-2.57	10.81	0.034
NM_007528	*Bcl6b*	-2.57	10.64	0.022
NM_016677	*Hpcal1*	-2.57	11.04	0.049
NM_029571	*Kti12*	-2.58	11.27	0.030

NM_009932	*Col4a2*	-2.58	10.77	0.042
NM_152808	*Slc44a2*	-2.59	15.59	0.001
NM_025399	*Nudt14*	-2.60	11.40	0.035
NM_172588	*Serinc5*	-2.62	11.09	0.010
NM_013643	*Ptpn5*	-2.63	10.18	0.046
NM_001033141	*1110006O17Rik*	-2.63	10.67	0.049
NM_013581	*Cog1*	-2.64	10.64	0.004
NM_021320	*Ntn4*	-2.64	9.86	0.012
NM_026027	*Pfdn1*	-2.64	12.20	0.019
NM_026042	*Ixl*	-2.65	11.35	0.049
AK080318	*Fmnl2*	-2.66	11.00	0.036
NM_054042	*Cd248*	-2.66	11.02	0.026
NM_172639	*5330431N19Rik*	-2.70	13.14	0.015
NM_029045	*4930432K21Rik*	-2.71	9.91	0.002
NM_147110	*Olfr570*	-2.74	11.72	0.048
NM_053074	*Nup62*	-2.76	11.19	0.049
NM_009923	*Cnp1*	-2.77	11.79	0.005
XM_885325	*Akap6*	-2.78	9.33	0.049
NM_008983	*Ptprk*	-2.80	13.19	0.028
AK043457	*D18Bwg0362e*	-2.80	9.08	0.006
NM_009931	*Col4a1*	-2.82	13.10	0.045
NM_031183	*Sp6*	-2.83	11.28	0.022
NM_013864	*Ndrg2*	-2.85	13.91	0.039
NM_007684	*Cetn3*	-2.86	11.91	0.037
NM_175145	*Tmem127*	-2.86	14.64	0.009
NM_030004	*Cryl1*	-2.91	14.01	0.020
NM_007648	*Cd3e*	-2.91	10.98	0.020
NM_134438	*Gpr37l1*	-2.98	12.18	0.041
NM_134226	*V1rj2*	-2.98	9.60	0.033
AK083081	*Sap18*	-3.00	9.51	0.048
NM_013805	*Cldn5*	-3.00	12.22	0.037
NM_138748	*Ppp2r4*	-3.02	12.04	0.028
NM_013884	*Cspg5*	-3.14	11.30	0.045
NM_052824	*Fxyd2*	-3.27	12.75	0.029
NM_007796	*Ctla2a*	-3.36	10.17	0.010
NM_178072	*Glcci1*	-3.38	10.91	0.009
NM_134122	*Nrm*	-3.49	10.62	0.041
NM_026793	*Myct1*	-3.57	10.41	0.017
NM_010050	*Dio2*	-3.60	12.09	0.041
NM_001033293	*Uap1l1*	-3.64	12.40	0.029
NM_029787	*Cyb5r3*	-3.64	13.48	0.012
NM_028243	*Prcp*	-3.87	9.82	0.019
NM_133832	*Rdh10*	-3.94	11.07	0.011
NM_025997	*2610204K14Rik*	-4.01	10.53	0.049
NM_009199	*Slc1a1*	-4.06	12.55	0.017
NM_026865	*1700113I22Rik*	-4.23	11.16	0.004
NM_011798	*Xcr1*	-4.31	11.92	0.010
NM_011338	*Ccl9*	-4.38	11.32	0.030
AK053676	*E130120C16Rik*	-4.72	9.35	0.015
