# Supplementary material for: Transcriptional Profiling of Chondrodysplasia Growth Plate Cartilage Reveals Adaptive ER-Stress Networks That Allow Survival but Disrupt Hypertrophy
Source: PLoS One. 2011 Sep 15;6(9):e24600. doi: 10.1371/journal.pone.0024600 (PMC3174197; doi:10.1371/journal.pone.0024600)
Supplement: Table S3 — Schmid versus wildtype GO analysis. (DOCX) [file pone.0024600.s007.docx]

| **Table S3 - Schmid versus Wildtype GO Analysis** | | |  |  |  |
| --- | --- | --- | --- | --- | --- |
|  |  |  |  |  |  |
| **GO Cluster rank and functional annotation** | | | **DAVID v6.7 Enrichment Score** | **Corrected *p* value** | **Count** |
| **Clusters associated with genes significantly upregulated in Schmid vs Wt:** | | |  |  |  |
| 1 | GO:0031974 | membrane-enclosed lumen | 9.99 | 7.95 E-17 | 111 |
| 2 | GO:0005783 | endoplasmic reticulum | 9.32 | 1.83 E-09 | 74 |
| 3 | GO:0015031 | protein transport | 6.80 | 8.59 E-07 | 58 |
| 4 | GO:0042254 | ribosome biogenesis | 6.60 | 1.14 E-07 | 23 |
| 5 | GO:0005739 | mitochondrion | 6.37 | 1.36 E-09 | 101 |
| 6 | GO:0003743 | translation initiation factor activity | 5.91 | 6.77 E-05 | 14 |
| 7 | SP_PIR_KEYWORDS | stress response | 5.83 | 5.44 E-07 | 14 |
| 8 | GO:0044432 | endoplasmic reticulum part | 5.09 | 1.18 E-05 | 28 |
| 9 | SP_PIR_KEYWORDS | chaperone | 4.93 | 4.52 E-06 | 23 |
| 10 | GO:0005794 | golgi apparatus | 4.18 | 2.16 E-02 | 44 |
| 11 | GO:0006986 | response to unfolded protein | 4.02 | 7.21 E-04 | 12 |
| 12 | GO:0000166 | nucleotide binding | 3.06 | 2.70 E-05 | 97 |
| 13 | GO:0044429 | mitochondrial part | 2.93 | 1.30 E-02 | 37 |
| 14 | GO:0048037 | cofactor binding | 2.68 | 4.87 E-02 | 20 |
| 15 | GO:0006396 | RNA processing | 2.50 | 1.33 E-05 | 42 |
| 16 | GO:0004812 | aminoacyl-tRNA ligase activity | 2.47 | 7.51 E-02 | 8 |
| 17 | GO:0042470 | melanosome | 2.38 | 1.04 E-02 | 12 |
| 18 | GO:0016053 | organic acid biosynthetic process | 2.37 | 1.98 E-02 | 16 |
| 19 | GO:0004386 | helicase activity | 2.24 | 8.40 E-02 | 13 |
| 20 | GO:0008219 | cell death | 2.23 | 2.03 E-01 | 32 |
| 21 | GO:0016864 | intramolecular oxidoreductase activity | 2.23 | 8.11 E-02 | 4 |
| 22 | GO:0005740 | mitochondrial envelope | 1.87 | 2.13 E-01 | 24 |
| 23 | GO:0005758 | mitochondrial intermembrane space | 1.83 | 8.51 E-02 | 6 |
| 24 | GO:0000154 | rRNA modification | 1.81 | 1.37 E-01 | 4 |
| 25 | SP_PIR_KEYWORDS | mitosis | 1.79 | 9.42 E-01 | 8 |
| 26 | GO:0008483 | transaminase activity | 1.74 | 7.73 E-02 | 6 |
| 27 | SP_PIR_KEYWORDS | nad | 1.71 | 2.76 E-01 | 13 |
| 28 | IPR000504 | RNA recognition motif, RNP-1 | 1.67 | 7.42 E-01 | 15 |
| 29 | GO:0043632 | modification-dependent macromolecule catabolic process | 1.63 | 2.57 E-01 | 31 |
| 30 | GO:0006006 | glucose metabolic process | 1.63 | 5.14 E-02 | 15 |
| 31 | GO:0043933 | macromolecular complex subunit organization | 1.61 | 7.91 E-02 | 27 |
| 32 | GO:0008652 | cellular amino acid biosynthetic process | 1.60 | 1.61 E-02 | 9 |
| 33 | GO:0005741 | mitochondrial outer membrane | 1.54 | 2.23 E-01 | 8 |
| 34 | GO:0007005 | mitochondrial organization | 1.50 | 2.84 E-01 | 10 |
| 35 | GO:0016591 | DNA-directed RNA polymerase II, holoenzyme | 1.48 | 8.35 E-02 | 8 |
| 36 | GO:0000287 | magnesium ion binding | 1.47 | 4.62 E-01 | 23 |
| 37 | GO:0050660 | FAD binding | 1.39 | 7.90 E-01 | 6 |
| 38 | GO:0051082 | unfolded protein binding | 1.38 | 6.30 E-02 | 10 |
| 39 | GO:0006541 | glutamine metabolic process | 1.38 | 5.16 E-01 | 4 |
| 40 | GO:0019217 | regulation of fatty acid metabolic process | 1.32 | 5.16 E-01 | 4 |
| **Clusters associated with genes significantly downregulated in Schmid vs Wt:** | | |  |  |  |
| 1 | SP_PIR_KEYWORDS | glycoprotein | 8.08 | 1.50 E-09 | 215 |
| 2 | GO:0031012 | extracellular matrix | 7.07 | 5.70 E-06 | 37 |
| 3 | IPR013091 | EGF calcium-binding | 6.07 | 3.09 E-06 | 17 |
| 4 | GO:0007155 | cell adhesion | 4.25 | 3.76 E-03 | 46 |
| 5 | GO:0030029 | actin filament-based process | 3.01 | 4.08 E-02 | 19 |
| 6 | GO:0001944 | vasculature development | 2.90 | 3.05 E-02 | 24 |
| 7 | GO:0035295 | tube development | 2.69 | 6.46 E-03 | 27 |
| 8 | IPR006020 | phosphotyrosine interaction region | 2.53 | 4.29 E-02 | 8 |
| 9 | GO:0008092 | cytoskeletal protein binding | 2.35 | 3.95 E-02 | 33 |
| 10 | GO:0044420 | extracellular matrix part | 2.30 | 7.94 E-02 | 12 |
| 11 | GO:0060348 | bone development | 2.25 | 3.93 E-01 | 12 |
| 12 | GO:0043009 | chordate embryonic development | 2.18 | 2.43 E-01 | 30 |
| 13 | GO:0000267 | cell fraction | 2.15 | 2.22 E-01 | 39 |
| 14 | GO:0035295 | tube development | 2.14 | 6.46 E-03 | 27 |
| 15 | IPR003129 | laminin-g, thrombospondin-type, N-terminal | 2.13 | 4.44 E-01 | 5 |
| 16 | SP_PIR_KEYWORDS | membrane | 2.05 | 2.30 E-03 | 263 |
| 17 | UP_SEQ_FEATURE | domain:EGF-like 6; calcium-binding | 2.03 | 3.03 E-01 | 6 |
| 18 | GO:0016477 | cell migration | 2.02 | 3.72 E-01 | 19 |
| 19 | GO:0035239 | tube morphogenesis | 2.01 | 1.54 E-01 | 17 |
| 20 | GO:0030136 | clathrin-coated vesicle | 1.84 | 8.02 E-02 | 13 |
| 21 | GO:0007179 | transforming growth factor beta receptor signaling pathway | 1.83 | 2.73 E-01 | 8 |
| 22 | GO:0042802 | identical protein binding | 1.79 | 4.14 E-01 | 21 |
| 23 | UP_SEQ_FEATURE | region of interest: Triple-helical region (COL3) | 1.76 | 8.50 E-01 | 3 |
| 24 | SP_PIR_KEYWORDS | proteoglycan | 1.72 | 4.37 E-01 | 6 |
| 25 | GO:0006928 | cell motion | 1.69 | 3.31 E-01 | 26 |
| 26 | SP_PIR_KEYWORDS | heparin binding | 1.62 | 2.72 E-02 | 9 |
| 27 | mmu04270 | vascular smooth muscle contraction | 1.57 | 1.13 E-01 | 14 |
| 28 | GO:0010817 | regulation of hormone levels | 1.52 | 9.64 E-02 | 15 |
| 29 | mmu04340 | hedgehog signaling pathway | 1.48 | 4.16 E-01 | 7 |
| 30 | GO:0031225 | anchored to membrane | 1.44 | 6.31 E-01 | 14 |
| 31 | GO:0051050 | positive regulation of transport | 1.39 | 5.02 E-01 | 12 |
| 32 | GO:0022604 | regulation of cell morphogenesis | 1.36 | 4.98 E-01 | 10 |
| 33 | UP_SEQ_FEATURE | domain:EGF-like 4 | 1.35 | 4.57 E-01 | 8 |
| 34 | GO:0007218 | neuropeptide signaling pathway | 1.34 | 7.79 E-01 | 7 |
| 35 | IPR001751 | s100/CaBP-9k-type, calcium binding | 1.34 | 8.63 E-01 | 4 |
| 36 | GO:0008015 | blood circulation | 1.32 | 6.01 E-01 | 10 |
