# Supplementary material for: Transcriptional Profiling of Chondrodysplasia Growth Plate Cartilage Reveals Adaptive ER-Stress Networks That Allow Survival but Disrupt Hypertrophy
Source: PLoS One. 2011 Sep 15;6(9):e24600. doi: 10.1371/journal.pone.0024600 (PMC3174197; doi:10.1371/journal.pone.0024600)
Supplement: Table S4 — Cog versus wildtype GO analysis. (DOCX) [file pone.0024600.s008.docx]

| **Table S4 - Cog versus Wildtype GO Analysis** | | |  |  |  |
| --- | --- | --- | --- | --- | --- |
|  |  |  |  |  |  |
| **GO Cluster rank and functional annotation** | | | **DAVID v6.7 Enrichment Score** | **Corrected *p* value** | **Count** |
| **Clusters associated with genes significantly upregulated in Cog vs Wt:** | | |  |  |  |
| 1 | GO:0005783 | endoplasmic reticulum | 7.41 | 7.59 E-06 | 28 |
| 2 | GO:0031974 | membrane-enclosed lumen | 2.85 | 3.45 E-03 | 27 |
| 3 | GO:0005788 | endoplasmic reticulum lumen | 2.58 | 3.18 E-05 | 9 |
| 4 | GO:0006986 | response to unfolded protein | 1.83 | 1.17 E-01 | 6 |
| 5 | GO:0001701 | in utero embryonic development | 1.62 | 4.72 E-01 | 9 |
| 6 | GO:0006457 | protein folding | 1.61 | 8.68 E-01 | 5 |
| 7 | GO:0006784 | ER-nuclear signaling pathway | 1.54 | 3.52 E-01 | 4 |
| 8 | GO:0009309 | amine biosynthetic process | 1.33 | 8.82 E-01 | 4 |
| **Clusters associated with genes significantly downregulated in Cog vs Wt:** | | |  |  |  |
| 1 | SP_PIR_KEYWORDS | lysosome | 1.37 | 8.24 E-01 | 4 |
| 2 | SP_PIR_KEYWORDS | oxidoreductase | 1.32 | 9.08 E-01 | 6 |
