# Supplementary material for: Transcriptional Profiling of Chondrodysplasia Growth Plate Cartilage Reveals Adaptive ER-Stress Networks That Allow Survival but Disrupt Hypertrophy
Source: PLoS One. 2011 Sep 15;6(9):e24600. doi: 10.1371/journal.pone.0024600 (PMC3174197; doi:10.1371/journal.pone.0024600)
Supplement: Table S5 — Cartilage-specific collagens and ECM components in schmid or cog versus wildtype. (DOCX) [file pone.0024600.s009.docx]

| **Table S5 - Cartilage-Specific Collagens and ECM Components in Schmid or Cog versus Wildtype** | | | | | | |
| --- | --- | --- | --- | --- | --- | --- |
|  |  |  |  |  |  |  |
|  |  |  |  |  |  |  |
| **NCBI** | **Gene Symbol** | **A** | **Relative Expression vs Wt (Adj P Value)** | |  |  |
|  |  |  | **Schmid** | **Cog** |  |  |
| **Cartilage Markers** |  |  |  |  |  |  |
| NM_007424 | *Agc1* | 18.80 | -1.12 (0.401) | -1.02 (0.933) |  |  |
| NM_007689 | *Chad* | 13.70 | -3.84 (0.003) | 1.12 (0.839) |  |  |
| NM_031163 | *Col2a1* | 18.52 | -1.34 (0.068) | -1.09 (0.710) |  |  |
| NM_009933 | *Col6a1* | 15.09 | -1.11 (0.687) | -1.99 (0.047) |  |  |
| NM_146007 | *Col6a2* | 12.92 | -1.63 (0.085) | 1.03 (0.954) |  |  |
| XM_897036 | *Col6a3* | 17.09 | 1.45 (0.286) | -1.21 (0.730) |  |  |
| NM_007738 | *Col9a1* | 15.05 | -1.85 (0.175) | -1.74 (0.381) |  |  |
| NM_007741 | *Col9a2* | 17.85 | -2.15 (0.012) | -1.61 (0.168) |  |  |
| NM_009936 | *Col9a3* | 15.12 | -3.03 (0.017) | -1.34 (0.623) |  |  |
| NM_007729 | *Col11a1* | 15.36 | -3.04 (0.058) | 1.88 (0.415) |  |  |
| NM_009926 | *Col11a2* | 17.36 | -2.89 (0.002) | -1.19 (0.627) |  |  |
| NM_016685 | *Comp* | 18.93 | -1.10 (0.342) | 1.02 (0.903) |  |  |
| NM_010217 | *Ctgf* | 17.14 | -4.82 (0.003) | -1.20 (0.769) |  |  |
| NM_007833 | *Dcn* | 14.96 | 1.61 (0.386) | 1.22 (0.834) |  |  |
| NM_007884 | *Epyc* | 14.95 | -3.00 (0.068) | -1.28 (0.798) |  |  |
| NM_021355 | *Fmod* | 12.30 | -2.97 (0.028) | -1.08 (0.928) |  |  |
| NM_016696 | *Gpc1* | 18.08 | -1.77 (0.001) | -1.05 (0.822) |  |  |
| NM_008150 | *Gpc4* | 14.81 | -1.15 (0.362) | 1.21 (0.346) |  |  |
| NM_013500 | *Hapln1* | 18.44 | 1.13 (0.790) | 1.31 (0.347) |  |  |
| NM_008524 | *Lum* | 16.64 | -1.12 (0.823) | 1.12 (0.887) |  |  |
| NM_010769 | *Matn1* | 15.81 | -6.02 (0.004) | -1.36 (0.657) |  |  |
| NM_016762 | *Matn2* | 13.31 | -3.39 (0.005) | -1.35 (0.524) |  |  |
| NM_010770 | *Matn3* | 16.39 | -1.74 (0.379) | 1.03 (0.984) |  |  |
| NM_013592 | *Matn4* | 13.55 | -1.98 (0.379) | 1.02 (0.988) |  |  |
| NM_054077 | *Prelp* | 16.69 | -2.10 (0.147) | 1.37 (0.688) |  |  |
| NM_011519 | *Sdc1* | 11.21 | -2.81 (0.013) | -1.44 (0.489) |  |  |
| NM_008304 | *Sdc2* | 13.31 | -3.20 (0.000) | -1.22 (0.487) |  |  |
| NM_011520 | *Sdc3* | 13.83 | -3.30 (0.001) | -2.54 (0.010) |  |  |
| NM_011521 | *Sdc4* | 14.48 | 1.70 (0.228) | 1.16 (0.848) |  |  |
| **Hypertrophic Cartilage Markers** | | |  |  |  |  |
| NM_007431 | *Akp2* | 17.07 | -2.07 (0.038) | 1.86 (0.156) |  |  |
| NM_009925 | *Col10a1* | 14.20 | 1.01 (0.996) | 2.03 (0.588) |  |  |
| NM_013599 | *Mmp9* | 14.25 | -7.03 (0.006) | -1.58 (0.776) |  |  |
| NM_008607 | *Mmp13* | 13.74 | -2.44 (0.379) | -1.39 (0.852) |  |  |
| NM_008608 | *Mmp14* | 14.07 | -2.65 (0.077) | 1.20 (0.365) |  |  |
| NM_009263 | *Spp1* | 17.96 | 1.41 (0.517) | -1.41 (0.674) |  |  |
| NM_009820 | *Runx2* | 14.92 | -1.55 (0.069) | -1.31 (0.407) |  |  |
