# Supplementary material for: Transcriptional Profiling of Chondrodysplasia Growth Plate Cartilage Reveals Adaptive ER-Stress Networks That Allow Survival but Disrupt Hypertrophy
Source: PLoS One. 2011 Sep 15;6(9):e24600. doi: 10.1371/journal.pone.0024600 (PMC3174197; doi:10.1371/journal.pone.0024600)
Supplement: Table S6 — ER stress sensors, their targets, and downstream pathways. (DOCX) [file pone.0024600.s010.docx]

| **Table S6 - ER Stress Sensors, Their Targets, and Downstream Pathways** | | | | | | | | | |  |
| --- | --- | --- | --- | --- | --- | --- | --- | --- | --- | --- |
|  |  | | |  | | |  |  |  |  |
| **NCBI** | **GeneName** | **A** | | | | **Relative Expression vs Wt (Adj P Value)** | | |  |  |
|  |  |  | | | | **Schmid** | | **Cog** |  |  |
| **Canonical ER Stress Sensors** |  |  | | | |  | |  |  |  |
| NM_001081304 | *Atf6* | 12.74 | | | | 3.03 (0.008) | | 5.53 (0.004) |  |  |
| NM_023913 | *Ire1* | 10.88 | | | | -1.09 (0.736) | | 1.36 (0.333) |  |  |
| NM_010121 | *Perk* | 12.64 | | | | 5.73 (0.048) | | 2.32 (0.469) |  |  |
| **Targets of Canonical ER Stress Sensors** | | | | | |  | |  |  |  |
| **Atf6:** |  |  | | | |  | |  |  |  |
| NM_029103 | *Armet* | 17.28 | | | | 2.99 (0.003) | | 3.08 (0.012) |  |  |
| NM_009722 | *Atp2a2* | 13.09 | | | | 1.53 (0.171) | | 3.12 (0.008) |  |  |
| NM_011631 | *Grp94* | 16.49 | | | | 9.61 (0.000) | | 19.95 (0.000) |  |  |
| NM_027400 | *Lman1* | 16.24 | | | | 1.94 (0.095) | | 2.24 (0.130) |  |  |
| NM_194350 | *Mafa* | 9.42 | | | | 1.00 (0.983) | | -1.18 (0.260) |  |  |
| NM_008725 | *Nppa* | 9.37 | | | | -1.09 (0.418) | | -1.08 (0.619) |  |  |
| NM_011850 | *Nr0b2* | 9.97 | | | | -1.17 (0.246) | | -1.04 (0.855) |  |  |
| NM_008814 | *Pdx1* | 9.73 | | | | -1.46 (0.049) | | 1.17 (0.544) |  |  |
| NM_020275 | *Tnfrsf10b* | 10.19 | | | | 1.62 (0.019) | | 1.23 (0.221) |  |  |
| NM_013842 | *Xbp1* | 10.72 | | | | 2.85 (0.058) | | 1.79 (0.428) |  |  |
| **Ire1:** |  |  | | | |  | |  |  |  |
| NM_022310 | *BiP* | 17.53 | | | | 4.62 (0.003) | | 4.91 (0.011) |  |  |
| NM_013760 | *ERdj4* | 12.90 | | | | 7.88 (0.000) | | 5.13 (0.003) |  |  |
| NM_011952 | *Mapk3* | | 12.37 | | -1.75 (0.042) | | | -1.99 (0.060) |  |  |
| NM_016700 | *Mapk8* | | 8.45 | | 1.10 (0.390) | | | -1.18 (0.295) |  |  |
| NM_008689 | *Nfkb1* | | 11.55 | | 3.72 (0.073) | | | 1.56 (0.429) |  |  |
| NM_019408 | *Nfkb2* | | 10.58 | | -1.36 (0.336) | | | -1.86 (0.156) |  |  |
| NM_015760 | *Nox4* | | 8.82 | | -1.11 (0.583) | | | 1.26 (0.363) |  |  |
| NM_011951 | *Mapk14* | | 12.25 | | 1.19 (0.442) | | | -1.02 (0.958) |  |  |
| NM_013693 | *Tnf* | | 9.89 | | -1.25 (0.121) | | | 1.15 (0.492) |  |  |
| NM_020275 | *Tnfrsf10b* | | 9.86 | | 1.62 (0.019) | | | -1.50 (0.104) |  |  |
| NM_028769 | *Syvn1* | | 16.29 | | 3.00 (0.002) | | | 4.03 (0.003) |  |  |
| **Perk:** |  | |  | |  | | |  |  |  |
| NM_007498 | *Atf3* | | 14.53 | | 8.10 (0.002) | | | 4.45 (0.038) |  |  |
| NM_007837 | *Chop* | | 13.94 | | 23.52 (0.000) | | | 12.88 (0.000) |  |  |
| NM_001005509 | *Eif2a* | | 11.25 | | 5.83 (0.015) | | | 2.05 (0.167) |  |  |
| NM_026114 | *Eif2s1* | | 14.00 | | 1.74 (0.049) | | | 1.25 (0.614) |  |  |
| NM_010481 | *Hspa9* | | 15.48 | | 5.19 (0.004) | | | 1.92 (0.254) |  |  |
| NM_010512 | *Igf1* | | 10.35 | | -1.86 (0.003) | | | 1.42 (0.010) |  |  |
| NM_028782 | *Lonp1* | | 14.20 | | 3.70 (0.000) | | | 1.31 (0.260) |  |  |
| NM_010902 | *Nfe2l2* | | 13.86 | | 2.36 (0.123) | | | 1.31 (0.767) |  |  |
| NM_008689 | *Nfkb1* | | 11.55 | | 3.72 (0.073) | | | 1.56 (0.429) |  |  |
| NM_019408 | *Nfkb2* | | 10.58 | | -1.36 (0.336) | | | -1.86 (0.156) |  |  |
| NM_008654 | *Gadd34* | | 10.20 | | 4.11 (0.000) | | | 3.04 (0.006) |  |  |
| NM_001039521 | *Rrn3* | | 9.66 | | -1.04 (0.760) | | | 1.15 (0.421) |  |  |
| NM_011716 | *Wfs1* | | 15.01 | | 3.76 (0.000) | | | 3.53 (0.004) |  |  |
| **Additional ER Stress Sensors** |  | |  | |  | | |  |  |  |
| NM_030080 | *Aibzip* | | 9.48 | | 2.52 (0.027) | | | -1.15 (0.822) |  |  |
| NM_178661 | *Bbf2h7* | | 13.36 | | 1.21 (0.573) | | | 1.76 (0.205) |  |  |
| NM_145365 | *Crebh* | | 10.12 | | -1.42 (0.379) | | | -2.55 (0.093) |  |  |
| NM_013497 | *Luman* | | 14.80 | | 4.00 (0.003) | | | 1.63 (0.290) |  |  |
| NM_011957 | *Oasis* | | 10.84 | | -1.42 (0.248) | | | -1.90 (0.128) |  |  |
| **Oxidoreductases & Protein Disulphide Isomerases** | | | | |  | | |  |  |  |
| NM_015774 | *Ero1l* | | 14.08 | | 8.47 (0.000) | | | 1.49 (0.283) |  |  |
| NM_026184 | *Ero1lb* | | 10.65 | | 8.15 (0.004) | | | 9.94 (0.011) |  |  |
| NM_007952 | *Erp57* | | 17.25 | | 2.05 (0.028) | | | 3.00 (0.019) |  |  |
| NM_009787 | *Erp72* | | 12.43 | | 16.37 (0.001) | | | 8.11 (0.022) |  |  |
| NM_027959 | *P5* | | 12.87 | | 4.97 (0.008) | | | 6.80 (0.001) |  |  |
| XM_128552 | *PDI* | | 9.75 | | -1.14 (0.406) | | | -1.06 (0.836) |  |  |
| NM_028295 | *Pdir* | | 13.20 | | 1.62 (0.354) | | | 1.88 (0.385) |  |  |
| **Endoplasmic Reticulum Associated Degradation (ERAD)** | | | | |  | | |  |  |  |
| NM_007597 | *Canx* | | 15.48 | | 1.81 (0.109) | | | 2.05 (0.061) |  |  |
| NM_007591 | *Calr* | | 18.11 | | 1.98 (0.003) | | | 2.20 (0.006) |  |  |
| NM_024207 | *Derl1* | | 14.87 | | 4.50 (0.005) | | | 2.70 (0.076) |  |  |
| NM_033562 | *Derl2* | | 12.52 | | 1.55 (0.039) | | | 2.06 (0.019) |  |  |
| NM_024440 | *Derl3* | | 13.73 | | 19.99 (0.000) | | | 54.65 (0.000) |  |  |
| NM_010072 | *Dpm1* | | 10.24 | | 2.45 (0.032) | | | -2.60 (0.074) |  |  |
| NM_010073 | *Dpm2* | | 14.59 | | 1.49 (0.056) | | | -1.03 (0.941) |  |  |
| NM_138677 | *Edem1* | | 12.65 | | 2.90 (0.005) | | | 2.20 (0.059) |  |  |
| NM_145537 | *Edem2* | | 10.49 | | 2.58 (0.056) | | | 2.05 (0.261) |  |  |
| NM_001039644 | *Edem3* | | 13.81 | | 2.67 (0.070) | | | 1.74 (0.450) |  |  |
| NM_022331 | *Herpud1* | | 16.66 | | 4.56 (0.000) | | | 5.79 (0.000) |  |  |
| NM_020586 | *Herpud2* | | 12.02 | | 3.14 (0.004) | | | 1.10 (0.848) |  |  |
| NM_016906 | *Sec61a1* | | 13.57 | | 3.77 (0.059) | | | 2.35 (0.349) |  |  |
| NM_021305 | *Sec61a2* | | 10.49 | | 2.09 (0.182) | | | 1.04 (0.968) |  |  |
| NM_008408 | *Stt3a* | | 10.74 | | 2.83 (0.004) | | | 1.50 (0.273) |  |  |
| NM_024222 | *Stt3b* | | 13.96 | | 2.96 (0.005) | | | 2.16 (0.071) |  |  |
| **Molecular Chaperones** |  | |  | |  | | |  |  |  |
| NM_019794 | *Dnaja2* | | 13.34 | | 1.84 (0.086) | | | -1.19 (0.501) |  |  |
| NM_023646 | *Dnaja3* | | 12.74 | | 3.88 (0.003) | | | 1.35 (0.541) |  |  |
| NM_018808 | *Dnajb1* | | 11.70 | | 2.66 (0.046) | | | -1.15 (0.865) |  |  |
| NM_008299 | *Dnajb3* | | 12.32 | | -1.93 (0.220) | | | -1.20 (0.848) |  |  |
| NM_025926 | *Dnajb4* | | 14.04 | | 1.67 (0.324) | | | -1.73 (0.459) |  |  |
| NM_019874 | *Dnajb5* | | 10.63 | | -2.18 (0.013) | | | 1.17 (0.711) |  |  |
| NM_001037940 | *Dnajb6* | | 14.97 | | 1.39 (0.172) | | | 1.10 (0.825) |  |  |
| NM_019964 | *Dnajb8* | | 10.14 | | -1.67 (0.093) | | | 1.00 (0.993) |  |  |
| NM_020266 | *Dnajb10* | | 11.82 | | -1.05 (0.918) | | | -1.61 (0.349) |  |  |
| NM_019965 | *Dnajb12* | | 12.94 | | -1.15 (0.785) | | | 1.84 (0.358) |  |  |
| NM_007869 | *Dnajc1* | | 14.06 | | 2.01 (0.077) | | | 1.76 (0.276) |  |  |
| NM_009584 | *Dnajc2* | | 9.55 | | 1.89 (0.157) | | | 2.20 (0.198) |  |  |
| NM_016775 | *Dnajc5* | | 10.71 | | 1.97 (0.132) | | | 1.69 (0.242) |  |  |
| NM_177677 | *Dnajc5g* | | 9.85 | | -1.88 (0.008) | | | 1.37 (0.232) |  |  |
| NM_172400 | *Dnajc8* | | 15.01 | | 1.27 (0.533) | | | -1.08 (0.907) |  |  |
| NM_024181 | *Dnajc10* | | 13.32 | | -1.21 (0.247) | | | -1.16 (0.530) |  |  |
| NM_172704 | *Dnajc11* | | 9.61 | | -2.74 (0.001) | | | 1.67 (0.064) |  |  |
| NM_001163026 | *Dnajc13* | | 10.79 | | 3.04 (0.037) | | | -1.20 (0.825) |  |  |
| NM_025384 | *Dnajc15* | | 14.27 | | -1.80 (0.369) | | | -1.50 (0.692) |  |  |
| NM_026400 | *ERdj3* | | 16.47 | | 2.25 (0.006) | | | 3.31 (0.004) |  |  |
| NM_008301 | *Hspa2* | | 12.04 | | 4.79 (0.000) | | | 1.29 (0.478) |  |  |
| NM_008300 | *Hspa4* | | 12.80 | | 3.68 (0.009) | | | -1.11 (0.879) |  |  |
| NM_011020 | *Hspa4l* | | 10.02 | | 1.78 (0.074) | | | -1.07 (0.908) |  |  |
| NM_031165 | *Hspa8* | | 10.14 | | 1.85 (0.170) | | | -1.10 (0.905) |  |  |
| NM_175199 | *Hspa12a* | | 9.09 | | -1.28 (0.084) | | | -1.02 (0.939) |  |  |
| NM_028306 | *Hspa12b* | | 9.79 | | 1.22 (0.489) | | | -2.14 (0.062) |  |  |
| NM_013560 | *Hspb1* | | 13.81 | | 5.27 (0.004) | | | 1.20 (0.801) |  |  |
| NM_030704 | *Hspb8* | | 11.43 | | 5.78 (0.047) | | | 1.00 (1.000) |  |  |
| NM_175111 | *Hspbap1* | | 11.90 | | 1.87 (0.099) | | | 1.61 (0.346) |  |  |
| NM_025486 | *Hspc171* | | 15.71 | | 1.03 (0.899) | | | 1.18 (0.613) |  |  |
| NM_010477 | *Hspd1* | | 16.84 | | 2.90 (0.025) | | | 1.16 (0.841) |  |  |
| NM_013559 | *Hsph1* | | 15.06 | | 2.83 (0.000) | | | 1.50 (0.098) |  |  |
| NM_008929 | *p58IPK* | | 14.73 | | 3.98 (0.000) | | | 5.23 (0.000) |  |  |
| **Autophagy** |  | |  | |  | | |  |  |  |
| NM_172669 | *Ambra1* | | 13.45 | | -1.33 (0.208) | | | 1.22 (0.534) |  |  |
| NM_026402 | *Atg3* | | 13.65 | | 1.10 (0.862) | | | -1.66 (0.435) |  |  |
| NM_174875 | *Atg4a* | | 12.02 | | 1.10 (0.858) | | | -1.55 (0.492) |  |  |
| NM_174874 | *Atg4b* | | 10.47 | | 1.85 (0.048) | | | -1.31 (0.507) |  |  |
| NM_175029 | *Atg4c* | | 10.60 | | 1.30 (0.689) | | | 1.10 (0.935) |  |  |
| NM_153583 | *Atg4d* | | 11.66 | | -1.03 (0.961) | | | -2.13 (0.245) |  |  |
| NM_053069 | *Atg5* | | 13.24 | | 2.58 (0.009) | | | 1.31 (0.525) |  |  |
| NM_028835 | *Atg7* | | 9.96 | | 1.12 (0.770) | | | -1.58 (0.337) |  |  |
| NM_025770 | *Atg10* | | 9.18 | | 1.76 (0.189) | | | -1.21 (0.794) |  |  |
| NM_026217 | *Atg12* | | 12.86 | | -1.07 (0.894) | | | -1.21 (0.730) |  |  |
| NM_029846 | *Atg16l1* | | 13.79 | | -1.37 (0.282) | | | 1.30 (0.541) |  |  |
| AK131109 | *Atg16l2* | | 10.35 | | 1.53 (0.238) | | | 1.08 (0.905) |  |  |
| NM_009741 | *Bcl2* | | 12.57 | | -1.29 (0.487) | | | -1.59 (0.349) |  |  |
| NM_019584 | *Becn1* | | 10.48 | | 1.41 (0.079) | | | -1.18 (0.555) |  |  |
| NM_009826 | *Fip200* | | 10.02 | | 1.73 (0.076) | | | 1.30 (0.548) |  |  |
| NM_019749 | *Gabarap* | | 13.49 | | -1.19 (0.763) | | | -1.12 (0.905) |  |  |
| NM_025735 | *Map1lc3a* | | 13.06 | | -4.74 (0.006) | | | -2.21 (0.186) |  |  |
| NM_026160 | *Map1lc3b* | | 15.85 | | -1.49 (0.177) | | | -1.65 (0.217) |  |  |
| NM_020009 | *Mtor* | | 10.23 | | 2.15 (0.200) | | | -1.32 (0.622) |  |  |
| NM_009469 | *Ulk1* | | 16.44 | | -1.31 (0.442) | | | 1.09 (0.890) |  |  |
| NM_013881 | *Ulk2* | | 15.81 | | -1.75 (0.070) | | | 6.38 (0.001) |  |  |
| AK004783 | *Ulk3* | | 14.79 | | 1.25 (0.660) | | | -1.12 (0.901) |  |  |
| AK016524 | *Ulk4* | | 9.39 | | -1.07 (0.720) | | | 1.15 (0.603) |  |  |
| **Apoptosis** |  | |  | |  | | |  |  |  |
| NM_007527 | *Bax* | | 10.52 | | -1.32 (0.274) | | | -1.36 (0.384) |  |  |
| NM_009741 | *Bcl2* | | 12.57 | | -1.29 (0.487) | | | -1.59 (0.349) |  |  |
| NM_207680 | *Bcl2l11* | | 9.84 | | -2.23 (0.007) | | | -1.78 (0.078) |  |  |
| NM_207680 | *Bim* | | 11.41 | | -2.23 (0.007) | | | -1.78 (0.078) |  |  |
| NM_009883 | *Cebpb* | | 15.47 | | 3.13 (0.000) | | | 2.95 (0.003) |  |  |
| NM_009808 | *Casp12* | | 9.87 | | -1.79 (0.137) | | | -1.04 (0.954) |  |  |
| NM_009810 | *Casp3* | | 10.11 | | 1.01 (0.986) | | | -1.13 (0.852) |  |  |
| NM_015733 | *Casp9* | | 11.99 | | 1.03 (0.969) | | | -1.40 (0.720) |  |  |
| NM_009802 | *Doc1* | | 13.14 | | 62.41 (0.000) | | | 6.30 (0.000) |  |  |
| NM_011858 | *Doc4* | | 11.97 | | 15.20 (0.006) | | | 4.14 (0.183) |  |  |
| NM_009635 | *Doc6* | | 9.84 | | 2.76 (0.020) | | | 1.88 (0.228) |  |  |
| NM_015774 | *Ero1l* | | 14.08 | | 8.47 (0.000) | | | 1.49 (0.283) |  |  |
| NM_020275 | *Tnfrsf10b* | | 9.86 | | 1.62 (0.019) | | | -1.50 (0.104) |  |  |
| NM_175093 | *Trib3* | | 14.84 | | 8.22 (0.000) | | | 5.68 (0.000) |  |  |
