# Supplementary material for: Transcriptional Profiling of Chondrodysplasia Growth Plate Cartilage Reveals Adaptive ER-Stress Networks That Allow Survival but Disrupt Hypertrophy
Source: PLoS One. 2011 Sep 15;6(9):e24600. doi: 10.1371/journal.pone.0024600 (PMC3174197; doi:10.1371/journal.pone.0024600)
Supplement: Table S7 — Wildtype hypertrophic zone gene expression signature. (DOCX) [file pone.0024600.s011.docx]

| **Table S7 - Wildtype Hypertrophic Zone Gene Expression Signature** | | |  |  |
| --- | --- | --- | --- | --- |
|  |  |  |  |  |
| **GenBank Accession** | **GeneName** | **Fold Diff** | **A** | **adj.P.Val** |
| AK090147 | *Ihh* | 102.98 | 15.33 | 0.004 |
| NM_139269 | *Hrasls3* | 101.92 | 11.39 | 0.006 |
| NM_007558 | *Bmp8a* | 85.90 | 13.38 | 0.009 |
| AK090207 | *Slc43a2* | 64.83 | 10.46 | 0.035 |
| NM_053080 | *Aldh1a3* | 62.29 | 14.54 | 0.021 |
| NM_010174 | *Fabp3* | 45.95 | 13.31 | 0.019 |
| NM_029770 | *Unc5b* | 42.06 | 13.40 | 0.023 |
| NM_011987 | *Pla2g10* | 40.77 | 12.14 | 0.011 |
| NM_009621 | *Adamts1* | 36.38 | 12.17 | 0.009 |
| NM_080555 | *Ppap2b* | 34.95 | 12.31 | 0.020 |
| AK032804 | *D5Ertd579e* | 34.80 | 13.19 | 0.039 |
| NM_033314 | *Slco2a1* | 33.67 | 10.80 | 0.008 |
| NM_138595 | *Gldc* | 31.75 | 11.44 | 0.015 |
| NM_013599 | *Mmp9* | 31.62 | 12.32 | 0.031 |
| NM_025282 | *Mef2c* | 27.81 | 14.46 | 0.010 |
| NM_028784 | *F13a1* | 27.44 | 12.73 | 0.023 |
| NM_145489 | *AI661453* | 25.29 | 11.47 | 0.001 |
| AK036214 | *Nebl* | 25.07 | 11.69 | 0.013 |
| NM_172633 | *Cbln2* | 24.37 | 12.15 | 0.020 |
| ENSMUST00000064814 | *ENSMUST00000064814* | 23.84 | 12.52 | 0.008 |
| NM_145980 | *8430408G22Rik* | 23.55 | 11.32 | 0.002 |
| NM_172294 | *Sulf1* | 22.35 | 11.71 | 0.014 |
| NM_009627 | *Adm* | 22.20 | 12.68 | 0.022 |
| AK030769 | *Rab11fip1* | 22.19 | 9.81 | 0.009 |
| AK003929 | *Auh* | 22.17 | 12.59 | 0.010 |
| NM_007731 | *Col13a1* | 21.96 | 11.13 | 0.028 |
| NM_010681 | *Lama4* | 19.83 | 12.25 | 0.011 |
| NM_175638 | *Wnk4* | 19.80 | 13.78 | 0.016 |
| NM_152804 | *Plk2* | 19.75 | 11.14 | 0.016 |
| NM_007930 | *Enc1* | 19.62 | 14.82 | 0.007 |
| NM_172633 | *Cbln2* | 19.61 | 12.55 | 0.023 |
| NM_020583 | *Isg20* | 19.53 | 13.50 | 0.026 |
| AK134633 | *Bean* | 19.40 | 10.13 | 0.006 |
| XM_485455 | *Grrp1* | 18.63 | 12.15 | 0.012 |
| NM_030143 | *Ddit4l* | 18.19 | 13.65 | 0.046 |
| NM_015744 | *Enpp2* | 18.16 | 13.34 | 0.009 |
| NM_009459 | *Ube2h* | 17.94 | 9.32 | 0.023 |
| NM_020258 | *Slc37a2* | 17.62 | 11.46 | 0.031 |
| NM_173370 | *Cds1* | 17.62 | 10.91 | 0.014 |
| NM_145562 | *9130213B05Rik* | 17.30 | 11.93 | 0.006 |
| AK137548 | *Mxd1* | 16.83 | 11.53 | 0.006 |
| NM_008607 | *Mmp13* | 16.80 | 11.52 | 0.024 |
| NM_153127 | *Mmrn2* | 16.75 | 10.99 | 0.030 |
| AK077026 | *Slc9a2* | 16.74 | 10.50 | 0.014 |
| AK144717 | *AK149472* | 16.29 | 10.22 | 0.001 |
| BC010335 | *BC010335* | 15.65 | 13.71 | 0.006 |
| NM_152803 | *Hpse* | 15.65 | 10.67 | 0.011 |
| NM_007669 | *Cdkn1a* | 15.56 | 13.80 | 0.030 |
| NM_008968 | *Ptgis* | 15.41 | 11.82 | 0.006 |
| AK009597 | *2310033K02Rik* | 15.03 | 10.18 | 0.020 |
| NM_013470 | *Anxa3* | 14.96 | 11.91 | 0.009 |
| NAP046356-1 | *NAP046356-1* | 14.77 | 12.84 | 0.020 |
| NM_028943 | *4933405A16Rik* | 14.72 | 14.00 | 0.014 |
| NM_008416 | *Junb* | 14.66 | 12.29 | 0.019 |
| NM_010755 | *Maff* | 14.33 | 10.42 | 0.021 |
| AK129084 | *Scrn1* | 14.20 | 10.03 | 0.021 |
| AK045020 | *Has2* | 14.04 | 13.19 | 0.020 |
| NM_133903 | *Spon2* | 14.03 | 15.32 | 0.007 |
| NM_010228 | *Flt1* | 13.80 | 11.00 | 0.031 |
| NM_175149 | *2310022B05Rik* | 13.55 | 12.09 | 0.006 |
| NM_001025577 | *Maf* | 13.53 | 11.06 | 0.008 |
| XM_920622 | *Rhobtb1* | 13.49 | 11.92 | 0.002 |
| NM_009373 | *Tgm2* | 13.17 | 14.72 | 0.008 |
| NM_133781 | *Cab39* | 12.91 | 12.43 | 0.048 |
| AK052489 | *Palld* | 12.65 | 12.82 | 0.018 |
| NM_007556 | *Bmp6* | 12.59 | 11.80 | 0.017 |
| NM_010494 | *Icam2* | 12.36 | 11.28 | 0.043 |
| AK084358 | *Dleu2* | 12.34 | 12.31 | 0.013 |
| NM_028995 | *Npal3* | 12.32 | 11.30 | 0.010 |
| NM_172925 | *D930047P17Rik* | 12.29 | 11.52 | 0.036 |
| NM_023320 | *Plekho1* | 12.13 | 12.10 | 0.027 |
| AK077574 | *Dsp* | 11.77 | 10.76 | 0.014 |
| NM_016701 | *Nes* | 11.54 | 11.48 | 0.022 |
| NM_013613 | *Nr4a2* | 11.05 | 10.88 | 0.024 |
| NM_016847 | *Avpr1a* | 10.81 | 10.23 | 0.020 |
| NM_010444 | *Nr4a1* | 10.79 | 11.39 | 0.023 |
| NM_011160 | *Prkg1* | 10.76 | 11.20 | 0.024 |
| NM_016719 | *Grb14* | 10.76 | 15.61 | 0.011 |
| NM_020258 | *Slc37a2* | 10.72 | 11.12 | 0.009 |
| NM_021375 | *Rhbg* | 10.62 | 11.58 | 0.037 |
| NM_013517 | *Fcer2a* | 10.58 | 10.51 | 0.018 |
| NM_133781 | *Cab39* | 10.56 | 12.90 | 0.012 |
| NM_010637 | *Klf4* | 10.42 | 14.35 | 0.046 |
| AK047015 | *AK047015* | 10.36 | 11.38 | 0.010 |
| NM_011808 | *Ets1* | 10.09 | 11.33 | 0.008 |
| NM_026866 | *Disp1* | 9.95 | 11.51 | 0.021 |
| NM_017405 | *Lsr* | 9.64 | 11.74 | 0.049 |
| NM_009400 | *Tnfrsf18* | 9.56 | 11.76 | 0.012 |
| NM_008851 | *Pitpnm1* | 9.56 | 12.17 | 0.002 |
| NM_009653 | *Alas2* | 9.51 | 10.99 | 0.013 |
| AK033097 | *Lmo4* | 9.43 | 12.57 | 0.033 |
| NM_011706 | *Trpv2* | 9.43 | 10.65 | 0.027 |
| NM_172647 | *F11r* | 9.23 | 11.27 | 0.011 |
| BC043115 | *0610010D24Rik* | 9.18 | 14.81 | 0.012 |
| NM_027307 | *Golph2* | 9.11 | 12.58 | 0.009 |
| NM_010681 | *Lama4* | 9.10 | 11.18 | 0.016 |
| NM_010864 | *Myo5a* | 9.02 | 13.92 | 0.002 |
| NM_008904 | *Ppargc1a* | 9.00 | 8.51 | 0.031 |
| NM_133234 | *Bbc3* | 8.91 | 13.51 | 0.042 |
| NM_133838 | *Ehd4* | 8.87 | 14.00 | 0.023 |
| NM_024479 | *Wbscr27* | 8.81 | 12.76 | 0.020 |
| NM_011077 | *Phex* | 8.77 | 11.57 | 0.037 |
| NM_010234 | *Fos* | 8.74 | 11.72 | 0.006 |
| NM_010139 | *Epha2* | 8.71 | 13.33 | 0.032 |
| NM_053083 | *Loxl4* | 8.68 | 10.74 | 0.031 |
| XM_001003960 | *Pde11a* | 8.64 | 10.19 | 0.006 |
| NM_144883 | *5430407P10Rik* | 8.63 | 13.86 | 0.006 |
| NM_021604 | *Agrin* | 8.59 | 14.99 | 0.045 |
| NM_172892 | *Slc13a4* | 8.56 | 11.28 | 0.010 |
| NM_026552 | *Arpc4* | 8.45 | 13.81 | 0.006 |
| NM_009177 | *St3gal1* | 8.40 | 12.83 | 0.005 |
| NM_025359 | *Tspan13* | 8.40 | 14.33 | 0.013 |
| NM_027476 | *Zdhhc24* | 8.38 | 10.60 | 0.038 |
| NM_007431 | *Akp2* | 8.36 | 15.76 | 0.048 |
| TC1709674 | *TC1448906* | 8.36 | 11.41 | 0.018 |
| NM_013751 | *Hrasls* | 8.33 | 11.36 | 0.034 |
| NM_008679 | *Ncoa3* | 8.31 | 12.81 | 0.036 |
| NM_008416 | *Junb* | 8.28 | 12.68 | 0.015 |
| AK033738 | *AK033738* | 8.26 | 10.60 | 0.017 |
| XM_193814 | *Col22a1* | 8.26 | 11.67 | 0.003 |
| NM_139297 | *Ugp2* | 8.16 | 15.24 | 0.009 |
| NM_175538 | *E130304F04Rik* | 8.12 | 12.36 | 0.013 |
| NM_011595 | *Timp3* | 8.11 | 13.56 | 0.012 |
| AK088706 | *E430024C06Rik* | 8.06 | 9.93 | 0.047 |
| NM_008135 | *Slc6a9* | 7.97 | 14.17 | 0.039 |
| NM_153068 | *Ehd2* | 7.88 | 11.25 | 0.011 |
| BC023116 | *Cgref1* | 7.86 | 16.88 | 0.012 |
| NM_015753 | *Zfhx1b* | 7.83 | 9.98 | 0.049 |
| NM_007440 | *Alox12* | 7.83 | 10.21 | 0.012 |
| NM_010544 | *Ihh* | 7.82 | 11.93 | 0.023 |
| NM_009396 | *Tnfaip2* | 7.82 | 11.81 | 0.008 |
| NM_138756 | *Slc25a36* | 7.82 | 12.05 | 0.021 |
| NM_181748 | *Gpr120* | 7.80 | 10.45 | 0.006 |
| NM_011272 | *Rln1* | 7.80 | 10.41 | 0.011 |
| NM_008808 | *Pdgfa* | 7.79 | 15.30 | 0.036 |
| NM_175121 | *Slc38a2* | 7.77 | 13.63 | 0.025 |
| NM_010050 | *Dio2* | 7.68 | 11.79 | 0.014 |
| NM_027268 | *Scrn1* | 7.68 | 10.93 | 0.008 |
| NM_201362 | *Ccdc68* | 7.63 | 11.50 | 0.033 |
| NM_021299 | *Ak3* | 7.62 | 13.92 | 0.009 |
| NM_172734 | *Stk38l* | 7.58 | 12.39 | 0.022 |
| NM_019742 | *Tusc2* | 7.48 | 12.77 | 0.034 |
| NM_009333 | *Tcf7l2* | 7.48 | 12.93 | 0.042 |
| NM_009029 | *Rb1* | 7.39 | 12.92 | 0.024 |
| AK129429 | *A630082K20Rik* | 7.38 | 12.62 | 0.017 |
| NM_015783 | *Isg15* | 7.31 | 11.02 | 0.012 |
| NM_007498 | *Atf3* | 7.29 | 11.46 | 0.042 |
| NM_009498 | *Vamp3* | 7.29 | 11.14 | 0.022 |
| NM_010474 | *Hs3st1* | 7.27 | 8.50 | 0.008 |
| AK011803 | *AK011803* | 7.24 | 10.45 | 0.008 |
| TC1605426 | *TC1463322* | 7.24 | 15.32 | 0.021 |
| NM_013767 | *Csnk1e* | 7.23 | 13.59 | 0.022 |
| XM_001003960 | *Pde11a* | 7.20 | 9.89 | 0.002 |
| NM_011212 | *Ptpre* | 7.17 | 11.20 | 0.029 |
| NM_025382 | *Tmem57* | 7.15 | 15.20 | 0.011 |
| NM_011405 | *Slc7a7* | 7.15 | 13.21 | 0.017 |
| AK021262 | *C430049B03Rik* | 7.14 | 12.79 | 0.004 |
| NM_008655 | *Gadd45b* | 7.10 | 14.86 | 0.040 |
| NM_194342 | *Unc84b* | 7.09 | 15.90 | 0.042 |
| NM_001077364 | *Tsc22d3* | 7.05 | 13.54 | 0.034 |
| NM_009831 | *Ccng1* | 7.04 | 13.39 | 0.022 |
| NM_153507 | *Cpne2* | 6.99 | 11.71 | 0.036 |
| NM_009551 | *Zfand5* | 6.98 | 12.04 | 0.017 |
| NM_153779 | *Amid* | 6.97 | 12.47 | 0.040 |
| AK030594 | *Ube2f* | 6.94 | 10.72 | 0.003 |
| NM_133903 | *Spon2* | 6.92 | 13.18 | 0.033 |
| NM_011278 | *Rnf4* | 6.87 | 11.45 | 0.015 |
| AK040092 | *AK040092* | 6.81 | 10.24 | 0.015 |
| NM_053197 | *Sfxn3* | 6.81 | 12.84 | 0.025 |
| NM_013531 | *Gnb4* | 6.77 | 12.10 | 0.017 |
| NM_172532 | *Aldh5a1* | 6.70 | 12.27 | 0.021 |
| NM_025427 | *1190002H23Rik* | 6.67 | 14.47 | 0.024 |
| NM_009368 | *Tgfb3* | 6.65 | 13.51 | 0.036 |
| NM_007936 | *Epha4* | 6.63 | 10.94 | 0.006 |
| NM_020583 | *Isg20* | 6.58 | 11.35 | 0.025 |
| NM_001033270 | *ENSMUST00000057015* | 6.56 | 12.89 | 0.028 |
| NM_172668 | *Lrp4* | 6.54 | 10.79 | 0.038 |
| NM_007722 | *Cmkor1* | 6.43 | 12.96 | 0.037 |
| NM_010128 | *Emp1* | 6.42 | 14.62 | 0.014 |
| AK028745 | *2310043N10Rik* | 6.40 | 15.40 | 0.023 |
| NM_008655 | *Gadd45b* | 6.39 | 12.55 | 0.023 |
| NM_144883 | *5430407P10Rik* | 6.38 | 12.05 | 0.010 |
| BC040811 | *Rbm28* | 6.35 | 10.78 | 0.039 |
| NM_029780 | *Raf1* | 6.29 | 15.55 | 0.005 |
| NM_010019 | *Dapk2* | 6.21 | 11.11 | 0.027 |
| AK079723 | *B3gat3* | 6.18 | 12.59 | 0.038 |
| NM_011198 | *Ptgs2* | 6.17 | 10.30 | 0.019 |
| NM_173870 | *Mgat4a* | 6.16 | 10.39 | 0.020 |
| NM_013473 | *Anxa8* | 6.13 | 16.87 | 0.048 |
| NM_198006 | *6330578E17Rik* | 6.13 | 12.12 | 0.038 |
| NM_027551 | *4631423F02Rik* | 6.12 | 11.43 | 0.031 |
| NM_010135 | *Enah* | 6.10 | 13.44 | 0.033 |
| NM_027238 | *1810054D07Rik* | 6.08 | 9.86 | 0.042 |
| NM_053252 | *G430002G23Rik* | 6.07 | 13.22 | 0.008 |
| NM_001008542 | *Mxi1* | 6.02 | 11.82 | 0.007 |
| NM_013546 | *Hebp1* | 6.01 | 13.77 | 0.024 |
| XM_131770 | *Otud3* | 6.00 | 12.86 | 0.036 |
| NM_009883 | *Cebpb* | 5.94 | 12.27 | 0.012 |
| AK084888 | *AK084888* | 5.94 | 10.45 | 0.021 |
| AK051168 | *AK051168* | 5.94 | 8.81 | 0.002 |
| NM_172935 | *Amdhd2* | 5.93 | 12.67 | 0.012 |
| AK173144 | *Ssh1* | 5.88 | 11.31 | 0.032 |
| NM_145837 | *Il17d* | 5.81 | 16.17 | 0.009 |
| NM_010863 | *Myo1b* | 5.75 | 10.27 | 0.035 |
| NM_181390 | *Mustn1* | 5.73 | 11.35 | 0.033 |
| NM_028071 | *Cotl1* | 5.70 | 14.81 | 0.032 |
| NM_207625 | *Acsl4* | 5.67 | 13.28 | 0.008 |
| NM_133191 | *Eps8l2* | 5.65 | 11.59 | 0.011 |
| NM_011200 | *Ptp4a1* | 5.62 | 14.52 | 0.014 |
| AK154784 | *Gpr160* | 5.60 | 9.16 | 0.006 |
| NM_176902 | *1110014K08Rik* | 5.60 | 13.87 | 0.044 |
| NM_012032 | *Serinc3* | 5.53 | 14.02 | 0.030 |
| AK044870 | *Col6a2* | 5.50 | 11.75 | 0.036 |
| NM_011491 | *Stc2* | 5.44 | 13.33 | 0.006 |
| NM_027294 | *Cmtm8* | 5.42 | 11.98 | 0.015 |
| NM_175316 | *Slco2b1* | 5.40 | 11.07 | 0.013 |
| NM_023628 | *Anxa9* | 5.40 | 9.43 | 0.003 |
| NM_025846 | *Rras2* | 5.40 | 12.18 | 0.012 |
| NM_198164 | *Cdc2l6* | 5.38 | 10.67 | 0.021 |
| NM_010518 | *Igfbp5* | 5.37 | 10.36 | 0.024 |
| NM_021284 | *Kras* | 5.37 | 11.00 | 0.041 |
| NM_178746 | *9130023D20Rik* | 5.34 | 12.10 | 0.022 |
| NM_053100 | *Trim8* | 5.33 | 13.92 | 0.019 |
| NM_008975 | *Ptp4a3* | 5.32 | 12.61 | 0.019 |
| AK043378 | *AK043378* | 5.29 | 7.96 | 0.006 |
| NM_007484 | *Rhoc* | 5.23 | 14.91 | 0.012 |
| NM_027482 | *5730508B09Rik* | 5.19 | 10.20 | 0.031 |
| NM_027288 | *Manba* | 5.17 | 12.27 | 0.011 |
| NM_133349 | *Zfand2a* | 5.17 | 12.30 | 0.040 |
| NM_001042660 | *Smad7* | 5.15 | 10.15 | 0.023 |
| NM_027265 | *2810004A10Rik* | 5.15 | 10.46 | 0.017 |
| NM_019819 | *Dusp14* | 5.12 | 10.74 | 0.034 |
| NM_130448 | *Pcdh18* | 5.08 | 10.26 | 0.038 |
| NM_010499 | *Ier2* | 5.06 | 12.76 | 0.035 |
| NM_026054 | *2810474O19Rik* | 5.05 | 13.68 | 0.006 |
| NM_026629 | *2410066E13Rik* | 4.98 | 10.86 | 0.031 |
| NM_009569 | *Zfpm1* | 4.96 | 11.44 | 0.018 |
| NM_001013753 | *Pcdh17* | 4.95 | 9.59 | 0.006 |
| NM_145439 | *Tmc6* | 4.94 | 13.02 | 0.009 |
| NM_134065 | *Epdr2* | 4.86 | 13.19 | 0.009 |
| ENSMUST00000073524 | *ENSMUST00000073524* | 4.85 | 12.23 | 0.046 |
| NM_011803 | *Klf6* | 4.84 | 15.14 | 0.015 |
| NM_177390 | *Myo1d* | 4.84 | 13.49 | 0.014 |
| NM_010440 | *Hmg20b* | 4.81 | 13.03 | 0.032 |
| NM_054043 | *Msi2* | 4.81 | 12.23 | 0.011 |
| NM_020296 | *Rbms1* | 4.81 | 11.90 | 0.024 |
| NM_011212 | *Ptpre* | 4.80 | 10.68 | 0.042 |
| NM_178804 | *Slit2* | 4.79 | 9.68 | 0.019 |
| NM_013885 | *Clic4* | 4.78 | 15.05 | 0.016 |
| NAP108117-1 | *NAP108117-1* | 4.78 | 11.82 | 0.026 |
| NM_011200 | *Ptp4a1* | 4.77 | 15.46 | 0.012 |
| NM_021890 | *Fads3* | 4.76 | 14.10 | 0.012 |
| NM_026439 | *Ccdc80* | 4.71 | 17.29 | 0.024 |
| NM_144900 | *Atp1a1* | 4.70 | 14.35 | 0.007 |
| AK147452 | *Snx27* | 4.68 | 9.82 | 0.017 |
| AK015225 | *Ptar1* | 4.66 | 9.78 | 0.015 |
| NM_020590 | *Gabarapl1* | 4.65 | 15.77 | 0.009 |
| ENSMUST00000099524 | *ENSMUST00000099524* | 4.63 | 14.05 | 0.034 |
| NM_009876 | *Cdkn1c* | 4.61 | 16.47 | 0.032 |
| NM_172691 | *B230312A22Rik* | 4.60 | 11.91 | 0.015 |
| AK084451 | *AK084451* | 4.59 | 9.84 | 0.033 |
| NM_010705 | *Lgals3* | 4.58 | 15.66 | 0.012 |
| NM_183028 | *Pcmtd1* | 4.55 | 11.58 | 0.029 |
| NM_178111 | *Trp53inp2* | 4.53 | 15.29 | 0.006 |
| NM_009113 | *S100a13* | 4.52 | 14.91 | 0.024 |
| AK090157 | *AK090157* | 4.50 | 9.71 | 0.022 |
| NM_026505 | *Bambi* | 4.48 | 13.77 | 0.022 |
| NM_174848 | *BC043118* | 4.47 | 15.11 | 0.034 |
| NM_012024 | *Ppp2r5e* | 4.44 | 12.18 | 0.025 |
| NM_201369 | *BC037393* | 4.40 | 10.60 | 0.006 |
| NM_021897 | *Trp53inp1* | 4.40 | 11.96 | 0.006 |
| NM_010728 | *Lox* | 4.34 | 11.04 | 0.030 |
| NM_016894 | *Ramp1* | 4.34 | 11.38 | 0.039 |
| NM_019636 | *Tbc1d1* | 4.32 | 10.94 | 0.032 |
| NM_133889 | *Bsdc1* | 4.30 | 12.45 | 0.017 |
| NM_026797 | *Dbndd2* | 4.30 | 13.36 | 0.007 |
| NM_001004364 | *Ddef2* | 4.29 | 12.54 | 0.013 |
| NM_025303 | *Stau2* | 4.29 | 10.79 | 0.019 |
| NM_011201 | *Ptpn1* | 4.24 | 14.38 | 0.027 |
| NM_026454 | *Ube2f* | 4.22 | 13.29 | 0.024 |
| NM_145629 | *Pls3* | 4.21 | 14.39 | 0.003 |
| BC030902 | *D430042O09Rik* | 4.20 | 12.77 | 0.038 |
| XM_139711 | *Arid1b* | 4.19 | 12.93 | 0.036 |
| AK090131 | *D330001F17Rik* | 4.18 | 12.77 | 0.034 |
| NM_008633 | *Mtap4* | 4.16 | 13.54 | 0.011 |
| NM_007521 | *Bach2* | 4.16 | 10.27 | 0.019 |
| NM_026629 | *2410066E13Rik* | 4.11 | 10.03 | 0.027 |
| NAP112463-1 | *NAP112463-1* | 4.10 | 11.19 | 0.013 |
| NM_007929 | *Emp2* | 4.10 | 14.00 | 0.009 |
| AK085945 | *Lama4* | 4.10 | 7.74 | 0.033 |
| NM_213659 | *Stat3* | 4.10 | 15.42 | 0.023 |
| AK017285 | *Clcn5* | 4.09 | 11.80 | 0.013 |
| NM_054043 | *Msi2* | 4.08 | 13.92 | 0.009 |
| AK147510 | *BC026657* | 4.04 | 10.77 | 0.047 |
| NM_025635 | *Zwint* | 4.04 | 13.42 | 0.024 |
| NM_173780 | *Klf8* | 4.01 | 11.19 | 0.022 |
| NM_026221 | *Ppfibp1* | 4.01 | 14.59 | 0.019 |
| NM_011805 | *Dido1* | 4.00 | 11.01 | 0.010 |
| BU920841 | *BU920841* | 4.00 | 12.08 | 0.022 |
| NM_011915 | *Wif1* | 3.95 | 10.61 | 0.010 |
| AK040830 | *Itga1* | 3.93 | 12.15 | 0.008 |
| NM_001037940 | *Dnajb6* | 3.91 | 13.09 | 0.009 |
| NM_016972 | *Slc7a8* | 3.89 | 9.98 | 0.032 |
| NM_153142 | *Slc35e4* | 3.88 | 13.85 | 0.010 |
| NM_025382 | *Tmem57* | 3.88 | 11.20 | 0.023 |
| NM_016898 | *Cd164* | 3.87 | 11.54 | 0.006 |
| NM_198303 | *Eif5b* | 3.87 | 13.32 | 0.025 |
| NM_024289 | *Osbpl5* | 3.86 | 16.07 | 0.016 |
| NM_023908 | *Slco3a1* | 3.85 | 10.78 | 0.046 |
| AK141119 | *BC017647* | 3.85 | 12.44 | 0.009 |
| NM_019491 | *Rala* | 3.84 | 14.11 | 0.035 |
| NM_029342 | *Nhej1* | 3.84 | 13.54 | 0.030 |
| NM_178654 | *Pkn2* | 3.82 | 11.28 | 0.025 |
| NM_009138 | *Ccl25* | 3.82 | 10.85 | 0.010 |
| NM_054078 | *Baz2a* | 3.79 | 11.61 | 0.042 |
| NM_146078 | *Ubr2* | 3.79 | 13.59 | 0.020 |
| BC099973 | *Rhobtb1* | 3.75 | 9.95 | 0.033 |
| AK017143 | *5031425E22Rik* | 3.75 | 10.41 | 0.023 |
| AK033818 | *AK033818* | 3.73 | 8.89 | 0.027 |
| NM_033217 | *Ngfr* | 3.73 | 11.04 | 0.047 |
| L23423 | *Itga7* | 3.73 | 10.53 | 0.015 |
| NM_145100 | *Lypd1* | 3.65 | 10.10 | 0.007 |
| NM_175329 | *Ndg2* | 3.63 | 11.70 | 0.024 |
| NM_001077694 | *Dysf* | 3.62 | 10.35 | 0.022 |
| AK153119 | *AW112010* | 3.62 | 10.36 | 0.007 |
| NM_133688 | *4930469P12Rik* | 3.59 | 12.77 | 0.024 |
| NM_026169 | *1200004M23Rik* | 3.57 | 14.76 | 0.009 |
| NM_009468 | *Dpysl3* | 3.56 | 10.64 | 0.036 |
| NM_199469 | *Nploc4* | 3.56 | 12.65 | 0.020 |
| NM_016736 | *6330412F12Rik* | 3.55 | 12.56 | 0.010 |
| NM_010757 | *Mafk* | 3.54 | 10.68 | 0.013 |
| NM_133206 | *Znrf1* | 3.54 | 12.42 | 0.014 |
| NM_133941 | *Dhx32* | 3.53 | 13.95 | 0.032 |
| AK028475 | *Sep-11* | 3.52 | 13.07 | 0.040 |
| NM_027250 | *2010305A19Rik* | 3.51 | 12.24 | 0.043 |
| XM_895387 | *2210408F21Rik* | 3.51 | 9.65 | 0.020 |
| AK051849 | *Fbxl7* | 3.50 | 9.70 | 0.046 |
| NM_021491 | *Smpd3* | 3.49 | 16.82 | 0.020 |
| NM_008869 | *Pla2g4a* | 3.49 | 9.81 | 0.026 |
| AY015062 | *Lats2* | 3.46 | 11.23 | 0.037 |
| NM_009229 | *Sntb2* | 3.46 | 11.82 | 0.045 |
| AK042386 | *Zfyve26* | 3.45 | 10.05 | 0.032 |
| NM_011670 | *Uchl1* | 3.45 | 10.79 | 0.028 |
| NM_001033144 | *1190007F08Rik* | 3.44 | 12.26 | 0.046 |
| NM_019661 | *Ykt6* | 3.44 | 12.86 | 0.014 |
| AK152407 | *Nmnat1* | 3.43 | 10.18 | 0.022 |
| NM_001042591 | *Arrdc3* | 3.42 | 11.60 | 0.025 |
| NM_007872 | *Dnmt3a* | 3.41 | 9.51 | 0.039 |
| NM_133738 | *Antxr2* | 3.40 | 14.08 | 0.033 |
| NM_026689 | *0610009K11Rik* | 3.36 | 11.65 | 0.031 |
| BC057368 | *Dock6* | 3.36 | 10.23 | 0.017 |
| NM_197986 | *1110007F12Rik* | 3.35 | 10.65 | 0.044 |
| NM_015734 | *Col5a1* | 3.34 | 15.29 | 0.011 |
| NM_019661 | *Ykt6* | 3.32 | 12.80 | 0.018 |
| NM_013642 | *Dusp1* | 3.32 | 11.02 | 0.018 |
| NM_010566 | *Inpp5d* | 3.28 | 10.37 | 0.034 |
| AK033091 | *Pdlim7* | 3.26 | 10.58 | 0.020 |
| NM_001042671 | *Prei4* | 3.23 | 12.27 | 0.039 |
| NM_133240 | *Acot8* | 3.23 | 13.76 | 0.020 |
| AK045240 | *B130050I23Rik* | 3.22 | 10.84 | 0.023 |
| NM_026689 | *0610009K11Rik* | 3.22 | 11.96 | 0.017 |
| NM_030132 | *D530033C11Rik* | 3.20 | 12.09 | 0.033 |
| NM_022327 | *Ralb* | 3.20 | 14.54 | 0.048 |
| NM_027238 | *1810054D07Rik* | 3.20 | 9.54 | 0.031 |
| XM_618787 | *2510012J08Rik* | 3.19 | 13.24 | 0.027 |
| NM_008823 | *Cfp* | 3.19 | 11.66 | 0.039 |
| NM_001037878 | *Tcf25* | 3.19 | 15.74 | 0.038 |
| AF229257 | *Usp29* | 3.19 | 11.12 | 0.049 |
| AK013994 | *AK013994* | 3.18 | 10.45 | 0.036 |
| NM_029956 | *Mmab* | 3.17 | 13.19 | 0.029 |
| NM_175127 | *Fbxo28* | 3.16 | 11.16 | 0.044 |
| NM_007491 | *Art5* | 3.16 | 10.73 | 0.032 |
| NM_021516 | *Mark3* | 3.15 | 12.26 | 0.040 |
| AK038070 | *Hivep3* | 3.14 | 13.05 | 0.009 |
| A_51_P301713 | *A_51_P301713* | 3.13 | 10.58 | 0.023 |
| NM_023395 | *Wfdc1* | 3.12 | 10.40 | 0.014 |
| NM_029688 | *Srxn1* | 3.12 | 12.82 | 0.027 |
| NM_146145 | *Jak1* | 3.11 | 12.34 | 0.032 |
| NM_016714 | *Nup50* | 3.10 | 11.17 | 0.027 |
| AK076900 | *AK076900* | 3.09 | 10.57 | 0.042 |
| XM_887283 | *Map3k10* | 3.07 | 11.52 | 0.009 |
| NM_021547 | *Stard3* | 3.06 | 13.67 | 0.009 |
| AK052455 | *Ddi2* | 3.05 | 10.79 | 0.006 |
| NM_009811 | *Casp6* | 3.05 | 11.85 | 0.011 |
| AK020957 | *Nuak1* | 3.05 | 14.64 | 0.017 |
| NM_025857 | *2310007F21Rik* | 3.05 | 13.37 | 0.007 |
| NM_008186 | *Gtf2h1* | 3.04 | 12.78 | 0.033 |
| AK077970 | *Tbc1d9b* | 3.03 | 10.83 | 0.048 |
| NM_007569 | *Btg1* | 3.01 | 15.63 | 0.024 |
| NM_152234 | *Ubqln1* | 3.00 | 15.02 | 0.025 |
| NM_008580 | *Map3k5* | 2.96 | 11.48 | 0.034 |
| NM_030244 | *Ier5l* | 2.96 | 10.86 | 0.017 |
| NM_133829 | *2210010L05Rik* | 2.94 | 9.91 | 0.034 |
| NM_027769 | *Cpne3* | 2.94 | 10.45 | 0.009 |
| AV121721 | *AV121721* | 2.92 | 12.93 | 0.036 |
| NM_024190 | *Chmp1b* | 2.92 | 11.28 | 0.034 |
| NM_001077705 | *Ptpn6* | 2.91 | 10.92 | 0.036 |
| NM_016775 | *Dnajc5* | 2.91 | 10.93 | 0.038 |
| NM_001033337 | *AW124722* | 2.91 | 10.70 | 0.039 |
| NM_013603 | *Mt3* | 2.90 | 11.42 | 0.031 |
| ENSMUST00000102856 | *AW011752* | 2.89 | 12.70 | 0.019 |
| NM_029492 | *Zdhhc20* | 2.87 | 13.07 | 0.024 |
| BC079880 | *2310066E14Rik* | 2.86 | 13.64 | 0.038 |
| NM_026615 | *2900073H19Rik* | 2.86 | 13.54 | 0.016 |
| NM_013749 | *Tnfrsf12a* | 2.85 | 14.83 | 0.021 |
| NM_133485 | *Ppp1r14c* | 2.85 | 10.83 | 0.049 |
| AK033091 | *Pdlim7* | 2.84 | 10.73 | 0.015 |
| NM_027857 | *Acy3* | 2.84 | 10.44 | 0.041 |
| NM_009132 | *Scin* | 2.84 | 16.13 | 0.017 |
| NM_145836 | *6430527G18Rik* | 2.84 | 10.77 | 0.026 |
| NM_025280 | *Kin* | 2.84 | 13.07 | 0.037 |
| NM_026369 | *Arpc5* | 2.82 | 11.99 | 0.032 |
| BC100534 | *AK052999* | 2.80 | 8.46 | 0.049 |
| NM_009469 | *Ulk1* | 2.80 | 15.01 | 0.016 |
| NM_012057 | *Irf5* | 2.78 | 9.59 | 0.039 |
| NM_008320 | *Irf8* | 2.78 | 10.16 | 0.040 |
| NM_020273 | *Gmeb1* | 2.78 | 12.71 | 0.032 |
| NM_009549 | *Zfp185* | 2.76 | 10.61 | 0.028 |
| NM_007945 | *Eps8* | 2.75 | 10.25 | 0.040 |
| NM_001002764 | *Smg6* | 2.73 | 11.35 | 0.028 |
| NM_010917 | *Nid1* | 2.72 | 14.21 | 0.019 |
| AK020461 | *9430040K09Rik* | 2.71 | 9.31 | 0.031 |
| NM_019586 | *Ube2j1* | 2.70 | 13.25 | 0.050 |
| NM_144871 | *Suv420h1* | 2.69 | 10.68 | 0.035 |
| NM_029342 | *Nhej1* | 2.69 | 11.95 | 0.010 |
| NM_172586 | *Zfp322a* | 2.69 | 13.07 | 0.034 |
| NM_054082 | *Mta3* | 2.68 | 12.33 | 0.018 |
| NM_025286 | *Slc31a2* | 2.68 | 10.48 | 0.008 |
| BC052391 | *Gpr125* | 2.67 | 14.57 | 0.020 |
| NM_013785 | *Ihpk1* | 2.66 | 10.97 | 0.042 |
| NM_175419 | *Actr5* | 2.66 | 13.04 | 0.038 |
| NM_145465 | *Stk24* | 2.65 | 11.41 | 0.006 |
| NM_015736 | *Galnt3* | 2.64 | 9.57 | 0.036 |
| AK048095 | *AK048095* | 2.64 | 15.87 | 0.033 |
| NM_007602 | *Capn5* | 2.63 | 10.71 | 0.046 |
| AK162987 | *AK162987* | 2.63 | 10.49 | 0.036 |
| NM_010278 | *Gfi1* | 2.62 | 10.34 | 0.030 |
| NM_025988 | *Acbd4* | 2.61 | 13.12 | 0.027 |
| NM_177093 | *Lrrc58* | 2.60 | 17.13 | 0.015 |
| NM_139307 | *Vasn* | 2.59 | 12.48 | 0.038 |
| XM_139038 | *Gm288* | 2.58 | 9.60 | 0.017 |
| NM_008410 | *Itm2b* | 2.57 | 15.04 | 0.025 |
| NM_030266 | *Inpp4a* | 2.57 | 10.32 | 0.012 |
| NM_009439 | *Psmd3* | 2.56 | 17.06 | 0.015 |
| D50494 | *D50494* | 2.56 | 10.77 | 0.048 |
| AK080390 | *AK080390* | 2.56 | 8.53 | 0.044 |
| NM_146091 | *5730596K20Rik* | 2.55 | 9.81 | 0.032 |
| NM_029532 | *6330548G22Rik* | 2.55 | 13.06 | 0.021 |
| AK013640 | *2900042K05Rik* | 2.54 | 10.02 | 0.044 |
| NM_008696 | *Map4k4* | 2.53 | 12.19 | 0.039 |
| NM_009643 | *Ahnak* | 2.53 | 15.27 | 0.015 |
| NM_013851 | *Abca8b* | 2.53 | 8.84 | 0.041 |
| NM_013785 | *Ihpk1* | 2.53 | 11.28 | 0.015 |
| AK129095 | *BC039210* | 2.50 | 16.84 | 0.020 |
| NM_020025 | *B3galt2* | 2.50 | 9.51 | 0.030 |
| AK034156 | *D030011O10Rik* | 2.49 | 8.71 | 0.017 |
| BC021311 | *Zswim6* | 2.49 | 10.72 | 0.029 |
| NM_001013378 | *Uspl1* | 2.47 | 12.68 | 0.020 |
| NM_007930 | *Enc1* | 2.45 | 9.75 | 0.025 |
| NM_026951 | *Pex11c* | 2.44 | 12.28 | 0.012 |
| AK087219 | *AK087219* | 2.43 | 8.60 | 0.009 |
| NM_175138 | *Dnaic1* | 2.43 | 10.83 | 0.024 |
| NM_009595 | *AK050127* | 2.43 | 11.96 | 0.012 |
| NM_012010 | *Eif2s3x* | 2.43 | 10.79 | 0.032 |
| NM_025952 | *2610529C04Rik* | 2.42 | 12.02 | 0.030 |
| NM_015736 | *Galnt3* | 2.41 | 10.03 | 0.038 |
| AK147212 | *Zbtb4* | 2.38 | 12.79 | 0.006 |
| AK076954 | *AK076954* | 2.38 | 10.20 | 0.012 |
| NM_009871 | *Cdk5r1* | 2.38 | 10.36 | 0.023 |
| NM_001037170 | *Tomm40l* | 2.37 | 11.32 | 0.030 |
| NM_001033430 | *A630082K20Rik* | 2.37 | 9.82 | 0.038 |
| NM_028894 | *Lonrf3* | 2.37 | 9.65 | 0.009 |
| NM_007457 | *Ap1s1* | 2.36 | 15.72 | 0.037 |
| NM_026217 | *Atg12* | 2.35 | 10.56 | 0.049 |
| AK150140 | *Wasf2* | 2.35 | 15.38 | 0.039 |
| AK081327 | *AK081327* | 2.34 | 9.72 | 0.015 |
| XM_985042 | *Rap1b* | 2.34 | 11.57 | 0.039 |
| BC003498 | *4931420C21Rik* | 2.34 | 12.73 | 0.048 |
| NM_144861 | *BC021395* | 2.32 | 10.71 | 0.050 |
| AK019498 | *Hspb1* | 2.32 | 8.51 | 0.028 |
| TC1666890 | *TC1410591* | 2.32 | 10.75 | 0.030 |
| NM_019873 | *Fkbpl* | 2.32 | 12.35 | 0.030 |
| NM_027242 | *2010007H12Rik* | 2.29 | 11.67 | 0.027 |
| NM_148958 | *Osbpl10* | 2.28 | 11.46 | 0.012 |
| NM_026275 | *Ube2r2* | 2.26 | 13.11 | 0.035 |
| NM_145611 | *Ankrd25* | 2.26 | 9.80 | 0.044 |
| NM_172397 | *Limd2* | 2.26 | 15.70 | 0.023 |
| NM_177595 | *Mkx* | 2.24 | 11.78 | 0.029 |
| NM_144839 | *Ube2e2* | 2.24 | 14.43 | 0.008 |
| NM_019443 | *Ndufa1* | 2.23 | 16.58 | 0.022 |
| AK162420 | *5730508B09Rik* | 2.23 | 10.39 | 0.011 |
| AK171223 | *Arhgef2* | 2.21 | 11.01 | 0.046 |
| NM_001039669 | *A930037G23Rik* | 2.17 | 10.78 | 0.018 |
| NM_001037957 | *Dyrk1b* | 2.15 | 10.44 | 0.037 |
| NM_025647 | *Cmpk* | 2.15 | 12.71 | 0.031 |
| AK032729 | *AK032729* | 2.13 | 12.11 | 0.041 |
| NM_177041 | *Flad1* | 2.12 | 11.70 | 0.016 |
| NM_009154 | *Sema5a* | 2.12 | 8.26 | 0.017 |
| NM_138598 | *D11Wsu99e* | 2.09 | 16.14 | 0.046 |
| XM_992182 | *Csnk2a2* | 2.09 | 13.79 | 0.030 |
| NM_015747 | *Slc20a1* | 2.09 | 12.55 | 0.043 |
| NM_153412 | *Phldb2* | 2.08 | 8.83 | 0.017 |
| AK172517 | *Strn* | 2.06 | 13.49 | 0.020 |
| NM_173431 | *1700047E16Rik* | 2.06 | 9.58 | 0.046 |
| NM_020590 | *Gabarapl1* | 2.05 | 10.39 | 0.046 |
| XM_484715 | *Sft2d3* | 2.05 | 14.84 | 0.033 |
| ENSMUST00000074329 | *ENSMUST00000074329* | 2.04 | 9.89 | 0.010 |
| NM_177390 | *Myo1d* | 2.02 | 9.74 | 0.033 |
| NM_133973 | *Cog4* | 2.02 | 14.04 | 0.008 |
| NM_133962 | *Arhgef18* | 2.00 | 11.83 | 0.049 |
