# Supplementary material for: Transcriptional Profiling of Chondrodysplasia Growth Plate Cartilage Reveals Adaptive ER-Stress Networks That Allow Survival but Disrupt Hypertrophy
Source: PLoS One. 2011 Sep 15;6(9):e24600. doi: 10.1371/journal.pone.0024600 (PMC3174197; doi:10.1371/journal.pone.0024600)
Supplement: Table S8 — Wildtype proliferative zone gene expression signature. (DOCX) [file pone.0024600.s012.docx]

| **Table S8 - Wildtype Proliferative Zone Gene Expression Signature** | | |  |  |
| --- | --- | --- | --- | --- |
|  |  |  |  |  |
| **GenBank Accession** | **GeneName** | **Fold Diff** | **A** | **adj.P.Val** |
| NM_028199 | *Plxdc1* | 89.52 | 12.88 | 0.006 |
| NM_008516 | *Lrrn1* | 28.21 | 11.20 | 0.012 |
| AK145379 | *H19* | 27.07 | 14.82 | 0.018 |
| XM_981216 | *LOC666048* | 24.15 | 10.41 | 0.010 |
| BC039980 | *Megf6* | 19.02 | 11.82 | 0.009 |
| NM_009387 | *Tk1* | 18.55 | 13.26 | 0.024 |
| NM_033075 | *D17H6S56E-5* | 18.53 | 11.73 | 0.019 |
| NM_023284 | *Cdca1* | 18.10 | 11.98 | 0.042 |
| NM_026214 | *Kctd4* | 16.84 | 10.39 | 0.007 |
| NM_007795 | *Ctf1* | 16.34 | 11.01 | 0.029 |
| NM_026066 | *Cmtm5* | 16.12 | 13.66 | 0.025 |
| NM_172301 | *Ccnb1* | 15.76 | 11.24 | 0.036 |
| NM_145741 | *Gdf10* | 15.60 | 13.47 | 0.033 |
| NM_145467 | *Itgbl1* | 15.45 | 9.80 | 0.006 |
| NM_009849 | *Entpd2* | 14.07 | 11.40 | 0.018 |
| NM_019395 | *Fbp1* | 13.98 | 12.03 | 0.039 |
| NM_001033145 | *1190002N15Rik* | 13.37 | 10.81 | 0.023 |
| NM_001033217 | *Prickle1* | 12.89 | 13.66 | 0.005 |
| NM_010656 | *Sspn* | 12.86 | 13.41 | 0.008 |
| NM_207161 | *BC048355* | 12.84 | 10.69 | 0.020 |
| NM_025830 | *Wwp2* | 12.83 | 13.73 | 0.022 |
| NM_016854 | *Ppp1r3c* | 12.74 | 11.40 | 0.005 |
| NM_025311 | *D14Ertd449e* | 12.24 | 13.89 | 0.015 |
| NM_009828 | *Ccna2* | 12.11 | 13.04 | 0.038 |
| NM_001002927 | *Penk1* | 11.99 | 14.44 | 0.009 |
| NM_146208 | *Neil3* | 11.92 | 10.63 | 0.002 |
| NM_146136 | *Slc16a4* | 11.56 | 11.68 | 0.024 |
| NM_009472 | *Unc5c* | 11.50 | 12.01 | 0.031 |
| NM_172484 | *E030049G20Rik* | 11.30 | 12.07 | 0.006 |
| NM_178879 | *C76566* | 11.17 | 13.27 | 0.009 |
| NM_013538 | *Cdca3* | 10.74 | 12.29 | 0.037 |
| NM_019656 | *Tspan6* | 10.74 | 14.45 | 0.022 |
| AK035842 | *AK035842* | 10.37 | 9.87 | 0.002 |
| NM_009696 | *Apoe* | 10.36 | 14.83 | 0.041 |
| NM_024184 | *Asf1b* | 10.30 | 12.39 | 0.046 |
| NM_009104 | *Rrm2* | 10.20 | 11.25 | 0.024 |
| AK147321 | *Gtl2* | 10.19 | 11.45 | 0.015 |
| NM_010726 | *Phyh* | 10.12 | 13.60 | 0.019 |
| NM_026433 | *Tmem100* | 10.12 | 10.19 | 0.015 |
| NM_016718 | *Ninj2* | 10.12 | 12.49 | 0.046 |
| NM_011727 | *Xlr3b* | 10.09 | 11.46 | 0.002 |
| NM_020052 | *Scube2* | 10.08 | 11.09 | 0.019 |
| NM_009369 | *Tgfbi* | 9.89 | 15.28 | 0.037 |
| NM_030198 | *Gins3* | 9.81 | 10.73 | 0.032 |
| NM_026410 | *Cdca5* | 9.73 | 12.33 | 0.024 |
| AK077691 | *Dact1* | 9.67 | 11.55 | 0.017 |
| NM_022316 | *Smoc1* | 9.57 | 10.38 | 0.008 |
| NM_007630 | *Ccnb2* | 9.55 | 13.55 | 0.034 |
| NM_007659 | *Cdc2a* | 9.53 | 10.85 | 0.024 |
| NM_019641 | *Stmn1* | 9.52 | 15.42 | 0.038 |
| NM_153776 | *Tmem121* | 9.48 | 12.41 | 0.039 |
| NM_007557 | *Bmp7* | 9.42 | 13.44 | 0.032 |
| AK085773 | *AK085773* | 9.42 | 9.93 | 0.034 |
| NM_029541 | *6530401D17Rik* | 9.41 | 11.30 | 0.002 |
| NM_007557 | *Bmp7* | 9.38 | 12.66 | 0.006 |
| NM_026214 | *Kctd4* | 9.34 | 10.47 | 0.008 |
| NM_198214 | *Snph* | 9.29 | 10.65 | 0.008 |
| XM_981786 | *Gtl2* | 9.19 | 12.34 | 0.008 |
| NM_016922 | *Gal3st1* | 9.11 | 12.03 | 0.017 |
| NM_007631 | *Ccnd1* | 9.05 | 12.20 | 0.020 |
| AK030381 | *Efcab1* | 9.04 | 11.41 | 0.014 |
| NM_026560 | *Cdca8* | 9.04 | 14.37 | 0.011 |
| AK147421 | *Acsl6* | 8.81 | 9.27 | 0.008 |
| NM_001029838 | *Pknox2* | 8.76 | 8.29 | 0.014 |
| NM_008610 | *Mmp2* | 8.68 | 14.44 | 0.036 |
| NM_013467 | *Aldh1a1* | 8.66 | 10.09 | 0.023 |
| NM_134117 | *AW548124* | 8.57 | 10.91 | 0.013 |
| AK083328 | *Lef1* | 8.55 | 10.97 | 0.025 |
| NM_020259 | *Hhip* | 8.51 | 11.81 | 0.049 |
| NM_028813 | *Vit* | 8.46 | 14.49 | 0.010 |
| NM_054071 | *Fgfrl1* | 8.40 | 13.94 | 0.018 |
| BC030178 | *Dtymk* | 8.37 | 12.40 | 0.022 |
| NM_023209 | *Pbk* | 8.33 | 10.99 | 0.017 |
| NM_021891 | *Fignl1* | 8.31 | 10.53 | 0.008 |
| NM_016662 | *Mxd3* | 8.17 | 12.10 | 0.030 |
| NM_023595 | *Dut* | 8.17 | 14.11 | 0.020 |
| NM_172607 | *Naprt1* | 8.12 | 11.53 | 0.017 |
| NM_016662 | *Mxd3* | 8.12 | 10.38 | 0.014 |
| NM_011284 | *Rpa2* | 8.11 | 11.27 | 0.009 |
| NM_001013368 | *E2f8* | 8.05 | 10.87 | 0.016 |
| NM_011447 | *Sox8* | 8.04 | 9.90 | 0.024 |
| NM_011830 | *Impdh2* | 7.98 | 12.70 | 0.005 |
| NM_198860 | *AI646023* | 7.97 | 12.58 | 0.009 |
| NM_026656 | *Mcoln2* | 7.97 | 11.67 | 0.006 |
| NM_011234 | *Rad51* | 7.87 | 11.03 | 0.024 |
| D00812 | *Rpa2* | 7.82 | 11.80 | 0.007 |
| NM_174870 | *Slc26a1* | 7.72 | 9.98 | 0.039 |
| ENSMUST00000030142 | *ENSMUST00000030142* | 7.65 | 13.67 | 0.019 |
| NM_026282 | *Spbc24* | 7.62 | 10.62 | 0.015 |
| NM_008489 | *Lbp* | 7.50 | 11.10 | 0.027 |
| NM_019391 | *Lsp1* | 7.48 | 11.34 | 0.032 |
| AK086149 | *A730017D01Rik* | 7.48 | 10.42 | 0.004 |
| NM_011170 | *Prnp* | 7.45 | 15.13 | 0.004 |
| NM_020043 | *Nope* | 7.44 | 11.28 | 0.006 |
| NM_009749 | *Bex2* | 7.39 | 11.99 | 0.023 |
| NM_009780 | *C4b* | 7.39 | 11.30 | 0.036 |
| NM_025495 | *Cenpp* | 7.23 | 11.47 | 0.017 |
| NM_008566 | *Mcm5* | 7.09 | 12.49 | 0.031 |
| NM_207650 | *Dtna* | 7.07 | 9.89 | 0.030 |
| NM_029568 | *Mfap4* | 7.01 | 11.72 | 0.039 |
| AK030820 | *Tfdp1* | 7.00 | 12.82 | 0.013 |
| NM_178255 | *Hapln3* | 6.99 | 12.71 | 0.009 |
| NM_178309 | *Brip1* | 6.95 | 10.03 | 0.007 |
| AK034012 | *Synpo* | 6.92 | 12.50 | 0.008 |
| NM_023122 | *Gpm6b* | 6.88 | 11.79 | 0.008 |
| NM_146094 | *Fads1* | 6.85 | 12.38 | 0.032 |
| BB551773 | *BB551773* | 6.82 | 10.98 | 0.040 |
| NM_146178 | *Ccdc106* | 6.78 | 10.71 | 0.029 |
| NM_028109 | *Tpx2* | 6.77 | 11.90 | 0.008 |
| AK043317 | *AK043317* | 6.76 | 10.14 | 0.012 |
| U12147 | *Lama2* | 6.75 | 10.57 | 0.006 |
| NM_178870 | *Hs3st3a1* | 6.70 | 10.64 | 0.014 |
| NM_011383 | *Six5* | 6.69 | 12.42 | 0.026 |
| AK008821 | *Cldn22* | 6.66 | 8.89 | 0.012 |
| XM_981751 | *AA987161* | 6.60 | 9.67 | 0.019 |
| NM_011488 | *Stat5a* | 6.57 | 12.02 | 0.020 |
| BC060266 | *Ddah1* | 6.53 | 11.90 | 0.017 |
| NM_001002272 | *Tro* | 6.49 | 12.64 | 0.018 |
| NM_029653 | *Dapk1* | 6.37 | 10.44 | 0.024 |
| NM_133762 | *Luzp5* | 6.36 | 11.41 | 0.011 |
| NM_012006 | *Acot1* | 6.33 | 13.17 | 0.010 |
| NM_008446 | *Kif4* | 6.32 | 10.65 | 0.032 |
| NM_026752 | *Zfyve21* | 6.31 | 12.54 | 0.009 |
| NM_026115 | *Hat1* | 6.29 | 10.02 | 0.009 |
| NM_016902 | *Nphp1* | 6.28 | 12.68 | 0.023 |
| NM_013658 | *Sema4a* | 6.23 | 10.80 | 0.039 |
| NM_011830 | *Impdh2* | 6.23 | 12.60 | 0.008 |
| NM_010849 | *Myc* | 6.20 | 12.16 | 0.022 |
| NM_013492 | *Clu* | 6.19 | 11.52 | 0.039 |
| NM_007900 | *Ect2* | 6.17 | 12.12 | 0.029 |
| NM_172301 | *Ccnb1* | 6.17 | 10.48 | 0.022 |
| NM_012043 | *Islr* | 6.16 | 15.38 | 0.024 |
| NM_001040691 | *Ung* | 6.15 | 14.33 | 0.023 |
| NM_133719 | *Metrn* | 6.15 | 12.09 | 0.024 |
| NM_001017983 | *Foxred2* | 6.14 | 9.50 | 0.039 |
| NM_178118 | *Dixdc1* | 6.07 | 11.83 | 0.028 |
| NM_207229 | *Plac9* | 6.06 | 16.34 | 0.020 |
| NM_146171 | *2810406C15Rik* | 6.05 | 12.40 | 0.015 |
| AK162525 | *Cybrd1* | 6.04 | 11.40 | 0.006 |
| NM_172961 | *Abat* | 6.02 | 11.14 | 0.008 |
| NM_176979 | *Topbp1* | 5.96 | 11.17 | 0.024 |
| NM_175027 | *Fancb* | 5.91 | 10.17 | 0.020 |
| NM_010849 | *Myc* | 5.87 | 11.72 | 0.018 |
| NM_021891 | *Fignl1* | 5.84 | 10.91 | 0.006 |
| XM_887155 | *Igsf10* | 5.83 | 11.19 | 0.011 |
| NM_011480 | *Srebf1* | 5.78 | 15.76 | 0.017 |
| ENSMUST00000096320 | *ENSMUST00000096320* | 5.77 | 9.97 | 0.029 |
| NM_011045 | *Pcna* | 5.68 | 14.39 | 0.010 |
| NM_025866 | *Cdca7* | 5.68 | 12.06 | 0.015 |
| NM_001029978 | *Tceal3* | 5.68 | 15.33 | 0.017 |
| NM_146248 | *Cchcr1* | 5.63 | 10.73 | 0.013 |
| NM_010247 | *Xrcc6* | 5.62 | 9.54 | 0.008 |
| NM_025676 | *Mcm8* | 5.61 | 9.69 | 0.042 |
| AK173292 | *Nek1* | 5.60 | 12.14 | 0.010 |
| NM_008565 | *Mcm4* | 5.60 | 14.05 | 0.006 |
| NM_001029978 | *1500026B10Rik* | 5.59 | 15.86 | 0.009 |
| NM_013744 | *Zfp354b* | 5.56 | 9.63 | 0.015 |
| NM_027435 | *Atad2* | 5.56 | 11.76 | 0.004 |
| NM_013837 | *Tpst1* | 5.56 | 14.30 | 0.007 |
| NM_009103 | *Rrm1* | 5.55 | 13.11 | 0.023 |
| NM_028763 | *Cbx6* | 5.50 | 12.26 | 0.024 |
| AK220535 | *A230106D06Rik* | 5.48 | 12.71 | 0.020 |
| NM_020510 | *Fzd2* | 5.47 | 13.36 | 0.019 |
| NM_010875 | *Ncam1* | 5.44 | 11.63 | 0.006 |
| NM_009253 | *Serpina3m* | 5.41 | 11.00 | 0.018 |
| NM_001040087 | *Sytl2* | 5.38 | 11.52 | 0.038 |
| NM_015754 | *Rbbp9* | 5.38 | 10.65 | 0.034 |
| NM_133851 | *Nusap1* | 5.36 | 10.97 | 0.036 |
| NM_008921 | *Prim1* | 5.30 | 11.94 | 0.035 |
| NM_018761 | *Ctnnal1* | 5.25 | 12.93 | 0.017 |
| AK031268 | *Odz3* | 5.22 | 11.65 | 0.012 |
| NM_026038 | *2810055F11Rik* | 5.21 | 12.34 | 0.014 |
| NAP029862-1 | *NAP029862-1* | 5.15 | 12.99 | 0.044 |
| NM_178788 | *Dctd* | 5.15 | 10.37 | 0.017 |
| NM_025838 | *1110004B13Rik* | 5.14 | 14.64 | 0.039 |
| NM_172696 | *Inadl* | 5.13 | 9.59 | 0.036 |
| NM_174857 | *Mamdc2* | 5.11 | 12.28 | 0.037 |
| NM_023627 | *Isyna1* | 5.08 | 13.36 | 0.032 |
| NM_010436 | *H2afx* | 5.07 | 15.12 | 0.020 |
| NM_010450 | *Hoxa11* | 5.05 | 12.24 | 0.015 |
| NM_019761 | *Nxt1* | 5.03 | 11.17 | 0.024 |
| NM_010144 | *Ephb4* | 4.97 | 10.25 | 0.033 |
| XM_899897 | *Cenpf* | 4.96 | 11.63 | 0.036 |
| AK138072 | *1190002F15Rik* | 4.95 | 13.94 | 0.010 |
| NM_009371 | *Tgfbr2* | 4.93 | 13.61 | 0.019 |
| NM_001002272 | *Tro* | 4.93 | 12.37 | 0.020 |
| NM_019641 | *Stmn1* | 4.92 | 9.99 | 0.009 |
| NM_010164 | *Eya1* | 4.90 | 10.72 | 0.031 |
| NM_024474 | *Emid2* | 4.88 | 11.83 | 0.020 |
| NM_130450 | *Elovl6* | 4.85 | 13.66 | 0.023 |
| NM_175494 | *Zfp367* | 4.84 | 11.93 | 0.012 |
| Y09632 | *Kif20a* | 4.80 | 12.62 | 0.038 |
| NM_030080 | *Aibzip* | 4.80 | 9.92 | 0.049 |
| NM_008687 | *Nfib* | 4.77 | 9.76 | 0.020 |
| AK135556 | *Cdc26* | 4.75 | 8.53 | 0.006 |
| NM_009870 | *Cdk4* | 4.74 | 13.05 | 0.046 |
| NM_026412 | *D2Ertd750e* | 4.74 | 7.97 | 0.028 |
| NM_199195 | *Bckdhb* | 4.73 | 11.66 | 0.024 |
| NM_028266 | *Col16a1* | 4.73 | 14.25 | 0.009 |
| NM_020599 | *Rlbp1* | 4.70 | 11.61 | 0.007 |
| NM_015781 | *Nap1l1* | 4.67 | 12.80 | 0.014 |
| NM_009079 | *Rpl22* | 4.66 | 14.35 | 0.005 |
| NM_172149 | *Bnip1* | 4.66 | 12.43 | 0.016 |
| NM_029631 | *Abhd14b* | 4.65 | 11.12 | 0.013 |
| NM_023317 | *Nde1* | 4.65 | 12.89 | 0.017 |
| NM_025312 | *Sostdc1* | 4.63 | 10.20 | 0.010 |
| NM_178936 | *Tmem56* | 4.63 | 11.48 | 0.014 |
| NM_022424 | *Fndc4* | 4.62 | 10.85 | 0.048 |
| NM_027230 | *Prkcbp1* | 4.62 | 12.30 | 0.023 |
| NM_001012392 | *U46068* | 4.60 | 10.49 | 0.029 |
| NM_031196 | *Slc19a1* | 4.59 | 13.71 | 0.017 |
| NM_011121 | *Plk1* | 4.58 | 12.20 | 0.010 |
| NM_145379 | *Mrgprf* | 4.58 | 9.75 | 0.036 |
| NM_007671 | *Cdkn2c* | 4.58 | 13.96 | 0.039 |
| X15052 | *Ncam1* | 4.55 | 14.60 | 0.020 |
| NM_010219 | *Fkbp4* | 4.55 | 13.14 | 0.018 |
| NM_019796 | *Syncrip* | 4.54 | 13.14 | 0.024 |
| NM_019976 | *Psrc1* | 4.53 | 9.74 | 0.017 |
| NM_010112 | *Efs* | 4.50 | 13.87 | 0.006 |
| NM_011196 | *Ptger3* | 4.46 | 9.83 | 0.041 |
| U19596 | *Cdkn2c* | 4.43 | 13.70 | 0.032 |
| BC052065 | *Rap1gap* | 4.39 | 12.45 | 0.006 |
| NM_178739 | *Wdr40b* | 4.39 | 10.31 | 0.044 |
| NM_019568 | *Cxcl14* | 4.38 | 10.59 | 0.035 |
| NM_019802 | *Ggcx* | 4.35 | 10.84 | 0.012 |
| NM_024433 | *Mtap* | 4.34 | 11.16 | 0.006 |
| NM_011415 | *Snai2* | 4.34 | 15.28 | 0.031 |
| NM_001012324 | *Ecm2* | 4.33 | 8.87 | 0.047 |
| AK014119 | *AK014119* | 4.32 | 13.00 | 0.006 |
| NM_009451 | *Tubb4* | 4.32 | 11.62 | 0.021 |
| NM_030259 | *BC003324* | 4.31 | 12.38 | 0.010 |
| NM_134122 | *Nrm* | 4.31 | 11.31 | 0.038 |
| NM_010883 | *Ndph* | 4.30 | 10.40 | 0.012 |
| NM_001024920 | *Trp53i13* | 4.30 | 12.73 | 0.006 |
| AK033357 | *E430014L09Rik* | 4.29 | 9.01 | 0.025 |
| NM_033320 | *Glce* | 4.28 | 14.03 | 0.005 |
| NM_008011 | *Fgfr4* | 4.26 | 11.87 | 0.050 |
| NM_011620 | *Tnnt3* | 4.26 | 13.37 | 0.019 |
| NM_007658 | *Cdc25a* | 4.25 | 8.96 | 0.039 |
| NM_008537 | *Amacr* | 4.25 | 12.59 | 0.034 |
| NM_183089 | *2600005O03Rik* | 4.25 | 9.68 | 0.039 |
| NM_133949 | *Ptov1* | 4.23 | 14.20 | 0.024 |
| NM_007633 | *Ccne1* | 4.21 | 12.83 | 0.042 |
| NM_022004 | *Fxyd6* | 4.21 | 15.80 | 0.044 |
| NM_008697 | *Nin* | 4.19 | 12.25 | 0.012 |
| NM_027230 | *Prkcbp1* | 4.19 | 13.67 | 0.006 |
| NM_008826 | *Pfkl* | 4.17 | 13.44 | 0.025 |
| NM_134164 | *Syt12* | 4.17 | 11.05 | 0.046 |
| NM_176073 | *Pgcp* | 4.17 | 13.53 | 0.016 |
| NM_021525 | *Rcl1* | 4.16 | 11.27 | 0.003 |
| XM_485838 | *Klhdc5* | 4.14 | 11.25 | 0.035 |
| NAP071064-1 | *NAP071064-1* | 4.14 | 14.47 | 0.033 |
| NM_008360 | *Il18* | 4.14 | 10.56 | 0.012 |
| NM_027642 | *Phf6* | 4.14 | 11.45 | 0.026 |
| NM_027309 | *Lysmd2* | 4.14 | 10.88 | 0.020 |
| AK220156 | *Lpin2* | 4.13 | 13.84 | 0.015 |
| NM_178873 | *Adck2* | 4.12 | 12.38 | 0.003 |
| NM_011690 | *Vars2* | 4.12 | 11.85 | 0.046 |
| NM_008880 | *Plscr2* | 4.11 | 9.65 | 0.035 |
| AK161741 | *Zhx3* | 4.10 | 13.63 | 0.009 |
| NM_026214 | *Kctd4* | 4.09 | 9.55 | 0.009 |
| NM_029823 | *2900062L11Rik* | 4.09 | 11.01 | 0.037 |
| NM_133706 | *Tmem97* | 4.09 | 13.94 | 0.035 |
| NM_144882 | *2810022L02Rik* | 4.09 | 13.78 | 0.032 |
| NM_009109 | *Ryr1* | 4.09 | 9.57 | 0.019 |
| NM_175009 | *Eny2* | 4.08 | 10.92 | 0.047 |
| NM_026739 | *9530077C05Rik* | 4.08 | 9.37 | 0.039 |
| NM_026613 | *2810027O19Rik* | 4.08 | 10.93 | 0.050 |
| NM_019578 | *Extl1* | 4.07 | 12.18 | 0.018 |
| NM_025833 | *Baiap2l1* | 4.07 | 10.98 | 0.044 |
| NM_001039556 | *E130016E03Rik* | 4.03 | 10.33 | 0.042 |
| NM_009004 | *Kif20a* | 4.03 | 12.23 | 0.026 |
| NM_009361 | *Tfdp1* | 4.03 | 12.35 | 0.041 |
| AK030381 | *Efcab1* | 4.03 | 9.99 | 0.017 |
| BF467941 | *Hist1h4i* | 4.02 | 11.41 | 0.024 |
| NM_145590 | *BC017158* | 4.02 | 12.49 | 0.033 |
| NM_024208 | *Echdc3* | 4.02 | 10.89 | 0.049 |
| AK079287 | *9530083O12Rik* | 4.01 | 11.13 | 0.034 |
| NM_001033158 | *Rasl12* | 4.00 | 12.24 | 0.017 |
| BQ178455 | *TC1519807* | 3.98 | 10.52 | 0.040 |
| NM_001014761 | *Scn2b* | 3.98 | 10.52 | 0.006 |
| NM_013834 | *Sfrp1* | 3.97 | 10.37 | 0.039 |
| AK171439 | *Nr3c1* | 3.97 | 12.85 | 0.009 |
| NM_146136 | *Slc16a4* | 3.97 | 9.86 | 0.008 |
| NM_133994 | *Gstt3* | 3.97 | 12.29 | 0.007 |
| XM_001000710 | *Btbd2* | 3.96 | 12.44 | 0.006 |
| NM_053155 | *Clmn* | 3.95 | 10.13 | 0.042 |
| BC079668 | *Whsc1* | 3.95 | 11.20 | 0.028 |
| NM_012039 | *Zw10* | 3.93 | 11.56 | 0.035 |
| NM_031392 | *Wdr6* | 3.92 | 12.20 | 0.033 |
| NM_026981 | *Dtwd1* | 3.92 | 10.01 | 0.027 |
| NM_027194 | *Tm2d2* | 3.92 | 12.30 | 0.034 |
| NM_008892 | *Pola1* | 3.92 | 11.38 | 0.031 |
| NM_013538 | *Cdca3* | 3.91 | 10.32 | 0.006 |
| NM_198127 | *Abi2* | 3.91 | 11.29 | 0.013 |
| NM_027027 | *Asb9* | 3.91 | 9.76 | 0.012 |
| NM_175245 | *2410129H14Rik* | 3.90 | 11.86 | 0.018 |
| XM_136212 | *Gli2* | 3.89 | 10.25 | 0.012 |
| NM_175473 | *Fras1* | 3.87 | 10.26 | 0.015 |
| NM_134122 | *Nrm* | 3.87 | 11.81 | 0.029 |
| NM_177372 | *Dna2l* | 3.85 | 9.63 | 0.012 |
| NM_016750 | *H2afz* | 3.85 | 15.60 | 0.011 |
| NM_029752 | *Bri3bp* | 3.84 | 12.16 | 0.017 |
| NM_198652 | *6430706D22Rik* | 3.84 | 12.21 | 0.040 |
| NAP121362-001 | *NAP121362-001* | 3.81 | 13.89 | 0.006 |
| NM_029752 | *Bri3bp* | 3.81 | 13.18 | 0.009 |
| NM_025372 | *Tipin* | 3.80 | 14.24 | 0.029 |
| NM_011632 | *Traf3* | 3.80 | 13.25 | 0.009 |
| NM_011620 | *Tnnt3* | 3.80 | 12.59 | 0.013 |
| NM_080462 | *Hnmt* | 3.79 | 9.72 | 0.006 |
| AK077379 | *Dnajc19* | 3.79 | 10.10 | 0.017 |
| NM_011029 | *Rpsa* | 3.78 | 15.51 | 0.027 |
| NM_145148 | *Frmd4b* | 3.78 | 10.64 | 0.020 |
| NM_172952 | *Gphn* | 3.78 | 11.08 | 0.039 |
| NM_011489 | *Stat5b* | 3.77 | 13.36 | 0.010 |
| ENSMUST00000101051 | *ENSMUST00000101051* | 3.76 | 10.67 | 0.024 |
| NM_007633 | *Ccne1* | 3.76 | 10.44 | 0.014 |
| NM_025610 | *Asrgl1* | 3.75 | 13.38 | 0.029 |
| NM_020567 | *Gmnn* | 3.75 | 13.61 | 0.015 |
| NM_009013 | *Rad51ap1* | 3.73 | 9.37 | 0.028 |
| NM_001045864 | *Rdbp* | 3.73 | 14.08 | 0.013 |
| NM_023277 | *Jam3* | 3.73 | 12.20 | 0.022 |
| BC094932 | *Exosc7* | 3.73 | 11.02 | 0.006 |
| NM_027354 | *Wdr51a* | 3.72 | 11.68 | 0.037 |
| NM_012052 | *Rps3* | 3.72 | 16.18 | 0.038 |
| NM_027975 | *2310007D09Rik* | 3.71 | 8.76 | 0.015 |
| NM_026708 | *Tlcd1* | 3.70 | 14.00 | 0.049 |
| NM_019722 | *Arl2* | 3.69 | 11.48 | 0.046 |
| NM_013900 | *Mfi2* | 3.68 | 16.10 | 0.024 |
| NM_199195 | *Bckdhb* | 3.68 | 11.11 | 0.024 |
| NM_021472 | *Rnase4* | 3.67 | 9.47 | 0.040 |
| AK051749 | *Arhgef15* | 3.65 | 9.16 | 0.032 |
| XM_204015 | *Rere* | 3.65 | 9.75 | 0.029 |
| NM_080445 | *B3galt6* | 3.65 | 11.27 | 0.022 |
| NM_028133 | *Egln3* | 3.63 | 9.90 | 0.019 |
| NM_007697 | *Chl1* | 3.62 | 10.98 | 0.007 |
| NM_007632 | *Ccnd3* | 3.60 | 12.78 | 0.014 |
| AK084221 | *AK084221* | 3.60 | 8.33 | 0.024 |
| NM_172149 | *Bnip1* | 3.60 | 12.90 | 0.011 |
| NM_001033599 | *Acsl6* | 3.59 | 10.24 | 0.005 |
| X82786 | *Mki67* | 3.59 | 9.61 | 0.011 |
| NM_026641 | *Ift80* | 3.58 | 11.38 | 0.023 |
| AK160693 | *4930430F08Rik* | 3.58 | 11.76 | 0.045 |
| NM_001033606 | *Acsl3* | 3.57 | 11.28 | 0.036 |
| NM_027930 | *2610016C23Rik* | 3.57 | 9.86 | 0.034 |
| NM_025314 | *Hars2* | 3.56 | 11.62 | 0.025 |
| U83902 | *Mad2l1* | 3.55 | 10.69 | 0.012 |
| NM_010561 | *Ilf3* | 3.54 | 12.97 | 0.034 |
| NM_010065 | *Dnm1* | 3.51 | 11.41 | 0.038 |
| AK033655 | *Sesn3* | 3.51 | 14.11 | 0.017 |
| AK151379 | *Rnf180* | 3.51 | 9.32 | 0.012 |
| NM_054087 | *Slc19a2* | 3.51 | 11.67 | 0.024 |
| NM_028428 | *Fut11* | 3.50 | 10.82 | 0.047 |
| NM_054077 | *Prelp* | 3.50 | 17.55 | 0.031 |
| NM_026404 | *Slc35a4* | 3.49 | 12.05 | 0.021 |
| AK137889 | *Stat5b* | 3.49 | 9.72 | 0.024 |
| NM_177054 | *Casc4* | 3.48 | 12.94 | 0.042 |
| NM_011220 | *Pts* | 3.47 | 11.13 | 0.020 |
| NM_178926 | *AI662250* | 3.47 | 10.82 | 0.042 |
| NM_019647 | *Rpl21* | 3.46 | 10.78 | 0.034 |
| AK048420 | *Fzd2* | 3.46 | 9.51 | 0.029 |
| NM_025636 | *2310079N02Rik* | 3.44 | 12.26 | 0.021 |
| NM_153083 | *Thtpa* | 3.43 | 11.91 | 0.017 |
| NM_025685 | *Col27a1* | 3.43 | 14.59 | 0.022 |
| NM_198161 | *Bhlhb9* | 3.41 | 11.48 | 0.032 |
| NM_028779 | *Ampd2* | 3.41 | 14.66 | 0.017 |
| AK036012 | *AK036012* | 3.41 | 8.40 | 0.020 |
| NM_021790 | *Cenpk* | 3.41 | 10.22 | 0.026 |
| NM_010219 | *Fkbp4* | 3.41 | 12.37 | 0.012 |
| NM_008949 | *Psmc3ip* | 3.40 | 10.10 | 0.021 |
| NM_146236 | *Tceal1* | 3.39 | 11.54 | 0.027 |
| AK141969 | *AU023006* | 3.39 | 9.66 | 0.008 |
| NM_026159 | *Retsat* | 3.38 | 11.02 | 0.043 |
| NM_025900 | *Dek* | 3.38 | 12.61 | 0.017 |
| BC049666 | *6330512M04Rik* | 3.37 | 10.25 | 0.014 |
| NM_177152 | *Lrig3* | 3.36 | 14.18 | 0.008 |
| NM_001079876 | *Gas2l3* | 3.36 | 9.96 | 0.040 |
| NM_028044 | *Cnn3* | 3.36 | 15.14 | 0.006 |
| NM_178269 | *Cenpm* | 3.35 | 10.45 | 0.006 |
| NM_133228 | *Zfp87* | 3.35 | 11.27 | 0.015 |
| NM_172660 | *D2Wsu81e* | 3.35 | 11.35 | 0.009 |
| NM_133948 | *Psip1* | 3.35 | 13.46 | 0.041 |
| NM_173400 | *6230416J20Rik* | 3.34 | 9.54 | 0.048 |
| NM_021463 | *Prps1* | 3.34 | 13.39 | 0.038 |
| NM_172779 | *6330505F04Rik* | 3.32 | 13.35 | 0.017 |
| NM_028680 | *Ift57* | 3.32 | 10.23 | 0.032 |
| AK170682 | *Tmem64* | 3.32 | 12.15 | 0.006 |
| NM_145462 | *D14Ertd500e* | 3.32 | 11.69 | 0.026 |
| NM_027547 | *Prdm5* | 3.31 | 10.74 | 0.022 |
| U89419 | *Rplp0* | 3.31 | 16.01 | 0.020 |
| AK146582 | *Alkbh1* | 3.30 | 11.14 | 0.033 |
| NM_028356 | *Zbtb25* | 3.28 | 11.95 | 0.039 |
| AF155157 | *Ap4b1* | 3.28 | 9.21 | 0.033 |
| AK082964 | *Bbf2h7* | 3.28 | 15.17 | 0.036 |
| AK031301 | *2810429I04Rik* | 3.28 | 8.74 | 0.024 |
| NM_010112 | *Efs* | 3.25 | 14.43 | 0.006 |
| NM_008415 | *Jrk* | 3.24 | 9.28 | 0.028 |
| NM_027230 | *Prkcbp1* | 3.24 | 13.96 | 0.034 |
| NM_021554 | *Mettl9* | 3.23 | 14.99 | 0.002 |
| NM_011993 | *Dpysl4* | 3.23 | 10.30 | 0.014 |
| NM_028022 | *2410008K03Rik* | 3.23 | 9.33 | 0.003 |
| NM_207650 | *Dtna* | 3.22 | 10.10 | 0.047 |
| NM_175283 | *Srd5a1* | 3.21 | 12.35 | 0.040 |
| NM_008949 | *Psmc3ip* | 3.21 | 10.32 | 0.026 |
| NM_173182 | *Fndc3b* | 3.21 | 13.84 | 0.026 |
| NM_178846 | *Gnl3* | 3.21 | 10.88 | 0.021 |
| NM_027295 | *Rab28* | 3.21 | 12.16 | 0.012 |
| NM_175266 | *Epm2aip1* | 3.20 | 10.94 | 0.022 |
| AK164165 | *Cd47* | 3.20 | 10.66 | 0.049 |
| NM_016957 | *Hmgn2* | 3.20 | 14.96 | 0.049 |
| NM_025954 | *1700012G19Rik* | 3.19 | 14.57 | 0.009 |
| NM_013737 | *Pla2g7* | 3.19 | 9.78 | 0.006 |
| NM_013588 | *Lrrc23* | 3.19 | 10.95 | 0.041 |
| AV242843 | *AV242843* | 3.18 | 9.51 | 0.017 |
| NM_026512 | *Bphl* | 3.18 | 13.63 | 0.034 |
| NM_019421 | *Cd320* | 3.17 | 10.12 | 0.006 |
| NM_033354 | *Lztr2* | 3.16 | 11.67 | 0.008 |
| NM_177743 | *C730027P07Rik* | 3.16 | 10.17 | 0.044 |
| NM_009230 | *Soat1* | 3.16 | 13.46 | 0.024 |
| NM_001047436 | *Pard6a* | 3.15 | 10.83 | 0.014 |
| NM_054088 | *Pnpla3* | 3.14 | 9.16 | 0.020 |
| NM_178605 | *D13Wsu177e* | 3.13 | 12.29 | 0.030 |
| NM_030700 | *Maged2* | 3.13 | 14.89 | 0.023 |
| NM_028223 | *3010001K23Rik* | 3.13 | 11.62 | 0.036 |
| AK169756 | *4933439C10Rik* | 3.12 | 10.55 | 0.017 |
| NM_001037756 | *Brms1l* | 3.12 | 11.35 | 0.038 |
| NM_011035 | *Pak1* | 3.11 | 10.44 | 0.012 |
| NM_148925 | *Fyco1* | 3.11 | 9.91 | 0.041 |
| AK122469 | *C530008M17Rik* | 3.11 | 9.96 | 0.009 |
| NM_009533 | *Xrcc5* | 3.11 | 11.33 | 0.022 |
| NM_181075 | *2610524H06Rik* | 3.10 | 12.79 | 0.047 |
| NM_145463 | *Tmem46* | 3.10 | 13.67 | 0.046 |
| BC029621 | *Prpsap1* | 3.10 | 12.87 | 0.027 |
| NM_013746 | *Plekhb1* | 3.09 | 12.56 | 0.018 |
| AK154302 | *Ahcy* | 3.09 | 11.97 | 0.012 |
| NM_009774 | *Bub3* | 3.09 | 14.16 | 0.014 |
| BC062107 | *A430041B07Rik* | 3.07 | 12.06 | 0.009 |
| NM_021504 | *Ngly1* | 3.07 | 11.98 | 0.020 |
| NM_027855 | *0610007C21Rik* | 3.06 | 14.90 | 0.040 |
| NM_172958 | *Mtmr12* | 3.06 | 10.85 | 0.020 |
| NM_010918 | *Nktr* | 3.06 | 13.08 | 0.024 |
| NM_144844 | *Pcca* | 3.06 | 10.14 | 0.046 |
| NM_025411 | *1110049F12Rik* | 3.06 | 13.08 | 0.034 |
| BC025208 | *Cd8b1* | 3.04 | 11.75 | 0.020 |
| NM_173737 | *8430410A17Rik* | 3.04 | 11.41 | 0.046 |
| AK090134 | *Anapc1* | 3.03 | 11.64 | 0.038 |
| NM_027412 | *Ttc9c* | 3.02 | 10.77 | 0.028 |
| NM_025590 | *Acot11* | 3.02 | 10.32 | 0.007 |
| NM_026446 | *Rgs19* | 3.02 | 11.33 | 0.030 |
| NM_134029 | *Nt5m* | 3.02 | 12.22 | 0.034 |
| NM_177861 | *Tmem67* | 3.02 | 9.64 | 0.019 |
| NAP071111-1 | *NAP071111-1* | 3.02 | 15.73 | 0.039 |
| AK165090 | *Pdk1* | 3.01 | 13.53 | 0.042 |
| BC096542 | *Nfib* | 3.01 | 10.03 | 0.040 |
| NM_010620 | *Kif15* | 3.00 | 9.37 | 0.014 |
| NM_024207 | *Derl1* | 3.00 | 14.14 | 0.012 |
| NM_025969 | *1700034H14Rik* | 2.99 | 12.42 | 0.024 |
| AK031720 | *Fem1c* | 2.99 | 10.32 | 0.049 |
| NM_019392 | *Tyro3* | 2.97 | 10.92 | 0.029 |
| NM_145443 | *L2hgdh* | 2.97 | 12.00 | 0.034 |
| NM_021346 | *Zfp318* | 2.96 | 10.74 | 0.042 |
| NM_027420 | *2610034B18Rik* | 2.96 | 9.45 | 0.017 |
| NM_031863 | *Cenpq* | 2.95 | 10.07 | 0.014 |
| NM_023671 | *Clns1a* | 2.95 | 12.68 | 0.014 |
| AK021164 | *Fzd9* | 2.94 | 12.96 | 0.023 |
| NM_023645 | *Kdelc1* | 2.94 | 13.39 | 0.018 |
| NM_008880 | *Plscr2* | 2.94 | 9.35 | 0.034 |
| NM_008722 | *Npm1* | 2.94 | 15.59 | 0.039 |
| AK020443 | *Ube2d3* | 2.94 | 12.04 | 0.015 |
| AK030036 | *4932416H05Rik* | 2.94 | 9.83 | 0.025 |
| NM_021567 | *Pcbp4* | 2.94 | 13.86 | 0.027 |
| NM_028593 | *Cybrd1* | 2.94 | 10.01 | 0.036 |
| NM_013686 | *Tcp1* | 2.93 | 12.95 | 0.038 |
| AK021086 | *C030016K15Rik* | 2.93 | 9.57 | 0.038 |
| NM_028481 | *Ccdc18* | 2.92 | 8.42 | 0.009 |
| NM_011857 | *Odz3* | 2.92 | 12.62 | 0.017 |
| TC1658525 | *TC1500867* | 2.91 | 10.10 | 0.039 |
| NM_172616 | *C330027C09Rik* | 2.91 | 9.75 | 0.024 |
| NM_153552 | *Thoc1* | 2.91 | 9.13 | 0.013 |
| NM_010207 | *Fgfr2* | 2.91 | 9.95 | 0.017 |
| NM_027988 | *Noxo1* | 2.90 | 10.94 | 0.025 |
| NM_009577 | *Zik1* | 2.90 | 10.45 | 0.033 |
| NM_019976 | *Psrc1* | 2.89 | 10.71 | 0.023 |
| AK136463 | *Lass6* | 2.89 | 12.50 | 0.028 |
| NM_025442 | *Alg5* | 2.88 | 14.68 | 0.008 |
| BC045521 | *Polr2e* | 2.88 | 11.85 | 0.029 |
| NM_023475 | *Serhl* | 2.88 | 12.95 | 0.023 |
| NM_025939 | *Paics* | 2.88 | 15.77 | 0.014 |
| NM_024168 | *Tsen34* | 2.87 | 13.48 | 0.034 |
| AK134517 | *AK134517* | 2.87 | 7.97 | 0.006 |
| NM_001039534 | *Pstk* | 2.86 | 12.58 | 0.025 |
| NM_138745 | *Mthfd1* | 2.86 | 12.21 | 0.020 |
| NM_013747 | *Golga5* | 2.85 | 13.39 | 0.015 |
| NM_153419 | *Grwd1* | 2.85 | 10.73 | 0.031 |
| NM_001004140 | *Ckap2* | 2.85 | 9.21 | 0.024 |
| NM_001010937 | *Gjb6* | 2.85 | 10.38 | 0.020 |
| NM_178705 | *Luzp2* | 2.84 | 9.17 | 0.027 |
| NM_019716 | *Orc6l* | 2.84 | 14.20 | 0.016 |
| NM_009031 | *Rbbp7* | 2.83 | 14.54 | 0.014 |
| NM_009360 | *Tfam* | 2.83 | 11.51 | 0.016 |
| BC065162 | *Tdp1* | 2.83 | 12.92 | 0.027 |
| NM_001007577 | *A630018P17Rik* | 2.82 | 10.74 | 0.011 |
| NM_134138 | *Tnfsf5ip1* | 2.82 | 10.13 | 0.040 |
| AV116077 | *B3bp* | 2.82 | 12.87 | 0.014 |
| NM_026240 | *Gramd3* | 2.81 | 12.96 | 0.014 |
| NM_001037812 | *Als2cr4* | 2.81 | 11.52 | 0.029 |
| AF208109 | *Il17rb* | 2.81 | 10.56 | 0.040 |
| NM_025613 | *Cri1* | 2.81 | 13.72 | 0.010 |
| NM_008458 | *Serpina3c* | 2.80 | 9.81 | 0.022 |
| NM_177261 | *Kndc1* | 2.79 | 8.82 | 0.012 |
| NM_019772 | *1110004F10Rik* | 2.79 | 14.32 | 0.042 |
| NM_029365 | *Med25* | 2.79 | 13.73 | 0.030 |
| NM_177382 | *Cyp2r1* | 2.78 | 9.14 | 0.007 |
| NM_019564 | *Htra1* | 2.78 | 15.56 | 0.037 |
| NM_177619 | *Myst2* | 2.78 | 11.93 | 0.025 |
| NM_198322 | *6820416H06Rik* | 2.77 | 9.43 | 0.020 |
| NM_175400 | *Sephs1* | 2.77 | 11.57 | 0.035 |
| NM_022813 | *Scamp2* | 2.77 | 14.55 | 0.024 |
| AK013141 | *2810423A18Rik* | 2.76 | 14.92 | 0.029 |
| NM_145371 | *Eif2b1* | 2.76 | 14.13 | 0.020 |
| NM_025319 | *0610009B22Rik* | 2.75 | 13.06 | 0.025 |
| NM_134040 | *Ddx1* | 2.75 | 13.17 | 0.024 |
| NM_145591 | *BC003267* | 2.75 | 9.57 | 0.031 |
| NM_029607 | *2310003C23Rik* | 2.75 | 10.14 | 0.046 |
| NM_008263 | *Hoxa10* | 2.74 | 10.45 | 0.024 |
| NM_152814 | *Zfp566* | 2.74 | 11.57 | 0.043 |
| NM_018887 | *Cyp39a1* | 2.73 | 10.73 | 0.012 |
| NM_001007578 | *Armcx6* | 2.73 | 12.44 | 0.028 |
| NM_027652 | *D5Wsu178e* | 2.72 | 9.28 | 0.030 |
| NM_175478 | *Lrfn3* | 2.72 | 11.91 | 0.032 |
| NM_026203 | *Ahi1* | 2.72 | 10.94 | 0.029 |
| NM_028410 | *Prkrir* | 2.72 | 12.79 | 0.020 |
| NM_172746 | *Hirip3* | 2.71 | 12.62 | 0.049 |
| NM_133766 | *C920006C10Rik* | 2.71 | 11.01 | 0.049 |
| AK086787 | *Steap2* | 2.71 | 9.67 | 0.023 |
| NM_011494 | *Stk16* | 2.70 | 9.31 | 0.007 |
| NM_021543 | *Pcdh8* | 2.70 | 9.44 | 0.010 |
| NM_019421 | *Cd320* | 2.70 | 13.55 | 0.031 |
| AK168445 | *Gart* | 2.70 | 10.66 | 0.033 |
| NM_020570 | *Xrcc2* | 2.69 | 9.51 | 0.031 |
| NM_009900 | *Clcn2* | 2.69 | 10.73 | 0.036 |
| NM_010066 | *Dnmt1* | 2.69 | 11.74 | 0.045 |
| NM_028713 | *3222401M22Rik* | 2.69 | 13.69 | 0.031 |
| NM_025281 | *Lyar* | 2.69 | 11.76 | 0.007 |
| AK129359 | *Cttnbp2nl* | 2.69 | 13.81 | 0.019 |
| XM_193936 | *Mphosph1* | 2.69 | 9.46 | 0.041 |
| AK148546 | *BC030336* | 2.68 | 11.69 | 0.011 |
| NM_177367 | *Gemin4* | 2.68 | 10.17 | 0.015 |
| NM_026511 | *2810002N01Rik* | 2.68 | 11.51 | 0.029 |
| NM_028131 | *2610510J17Rik* | 2.68 | 9.72 | 0.039 |
| NM_016683 | *Zfp95* | 2.67 | 11.45 | 0.039 |
| NM_025902 | *1500009M05Rik* | 2.67 | 9.43 | 0.040 |
| NM_028108 | *Nat13* | 2.67 | 11.59 | 0.014 |
| AK038685 | *AK038685* | 2.66 | 8.42 | 0.019 |
| NM_019793 | *Tspan3* | 2.66 | 15.91 | 0.020 |
| NM_024198 | *Gpx7* | 2.65 | 15.21 | 0.048 |
| NM_133953 | *Sf3b3* | 2.65 | 12.45 | 0.020 |
| NM_026499 | *Sfrs6* | 2.65 | 11.61 | 0.022 |
| BC060121 | *Phf20* | 2.65 | 9.77 | 0.046 |
| AK049387 | *Rps24* | 2.65 | 11.09 | 0.015 |
| NM_010231 | *Fmo1* | 2.65 | 8.40 | 0.036 |
| NM_017462 | *Polg* | 2.64 | 11.13 | 0.006 |
| AK088451 | *E430016P22Rik* | 2.63 | 12.32 | 0.049 |
| AK052715 | *Chdh* | 2.63 | 10.40 | 0.024 |
| NM_026955 | *2200002K05Rik* | 2.63 | 9.62 | 0.006 |
| NM_026271 | *1110018M03Rik* | 2.63 | 16.61 | 0.025 |
| NM_145919 | *Abhd14a* | 2.63 | 9.90 | 0.039 |
| AK018584 | *Zfp251* | 2.61 | 13.25 | 0.006 |
| BC022619 | *Phr1* | 2.61 | 12.05 | 0.019 |
| NM_020618 | *Smarce1* | 2.61 | 13.31 | 0.031 |
| NM_029086 | *5830415F09Rik* | 2.60 | 9.84 | 0.023 |
| NM_018884 | *Pdzrn3* | 2.60 | 13.95 | 0.040 |
| XM_129027 | *Cep76* | 2.59 | 9.64 | 0.024 |
| NM_023525 | *Cad* | 2.58 | 12.78 | 0.020 |
| NM_019704 | *Tmem115* | 2.58 | 12.88 | 0.038 |
| NM_133976 | *Imp3* | 2.58 | 13.71 | 0.027 |
| AK018344 | *Vamp4* | 2.58 | 11.93 | 0.008 |
| AK010171 | *Bxdc2* | 2.57 | 11.39 | 0.039 |
| NM_145546 | *Gtf2b* | 2.56 | 13.66 | 0.012 |
| NM_001001882 | *Rtel1* | 2.56 | 9.63 | 0.041 |
| NM_027187 | *Rnaseh2a* | 2.55 | 12.32 | 0.049 |
| NM_021790 | *Cenpk* | 2.55 | 9.41 | 0.010 |
| NM_021713 | *Myg1* | 2.55 | 11.88 | 0.006 |
| XM_358903 | *Zfp174* | 2.54 | 8.97 | 0.017 |
| NM_027194 | *Tm2d2* | 2.54 | 14.77 | 0.039 |
| AK129377 | *Mll3* | 2.54 | 11.85 | 0.049 |
| AK082136 | *Prmt3* | 2.54 | 9.72 | 0.032 |
| NM_026758 | *Mphosph6* | 2.54 | 14.33 | 0.006 |
| AK040524 | *Gns* | 2.53 | 8.74 | 0.033 |
| NM_025289 | *Tbrg1* | 2.53 | 14.81 | 0.024 |
| NM_008057 | *Fzd7* | 2.52 | 9.63 | 0.040 |
| NM_001039723 | *BC022593* | 2.52 | 10.62 | 0.038 |
| AK004937 | *Bbs10* | 2.52 | 9.74 | 0.027 |
| NM_172719 | *Gcn1l1* | 2.52 | 12.51 | 0.049 |
| NM_178939 | *Pdrg1* | 2.52 | 16.36 | 0.013 |
| NM_026940 | *1810015A11Rik* | 2.52 | 12.78 | 0.015 |
| NM_010292 | *Gck* | 2.51 | 9.88 | 0.046 |
| NM_028298 | *Zfp655* | 2.51 | 10.92 | 0.029 |
| NM_011291 | *Rpl7* | 2.51 | 16.09 | 0.020 |
| NM_175384 | *Cdca2* | 2.50 | 9.62 | 0.012 |
| NM_009532 | *Xrcc1* | 2.50 | 13.33 | 0.033 |
| NM_148925 | *Fyco1* | 2.50 | 9.76 | 0.015 |
| NM_016792 | *Txnl1* | 2.50 | 8.02 | 0.019 |
| NM_019499 | *Mad2l1* | 2.50 | 10.13 | 0.026 |
| NM_008316 | *Hus1* | 2.50 | 11.14 | 0.034 |
| NM_133854 | *Snapap* | 2.49 | 11.17 | 0.019 |
| NM_008663 | *Myo7a* | 2.49 | 10.07 | 0.043 |
| NM_178788 | *Dctd* | 2.49 | 9.71 | 0.036 |
| NM_025880 | *2410002F23Rik* | 2.48 | 12.54 | 0.015 |
| NM_181595 | *Ppp1r9a* | 2.48 | 11.05 | 0.010 |
| NM_016918 | *Nudt5* | 2.48 | 11.72 | 0.039 |
| NM_027263 | *2610040C18Rik* | 2.47 | 8.96 | 0.022 |
| NM_153540 | *C85492* | 2.47 | 12.69 | 0.014 |
| NM_020591 | *A030009H04Rik* | 2.46 | 9.23 | 0.048 |
| NAP112061-1 | *NAP112061-1* | 2.46 | 15.11 | 0.030 |
| AK009643 | *2310035P21Rik* | 2.46 | 9.99 | 0.038 |
| NM_011991 | *Cops3* | 2.46 | 12.93 | 0.049 |
| NM_145956 | *Brcc3* | 2.45 | 9.32 | 0.017 |
| NM_008563 | *Mcm3* | 2.44 | 9.83 | 0.020 |
| AK144813 | *Tbrg4* | 2.44 | 11.70 | 0.020 |
| NM_013684 | *Tbp* | 2.44 | 9.98 | 0.020 |
| AK004937 | *Bbs10* | 2.44 | 9.32 | 0.015 |
| XM_131720 | *2610002D18Rik* | 2.43 | 10.63 | 0.024 |
| NM_019833 | *B230317C12Rik* | 2.43 | 10.74 | 0.025 |
| NM_016905 | *Galk1* | 2.43 | 15.82 | 0.037 |
| NM_013853 | *Abcf2* | 2.43 | 10.89 | 0.024 |
| NM_001025375 | *Wdr61* | 2.42 | 14.35 | 0.033 |
| AK129078 | *2600005C20Rik* | 2.42 | 10.37 | 0.033 |
| NM_008784 | *Igbp1* | 2.41 | 9.71 | 0.022 |
| NM_011274 | *C80913* | 2.41 | 8.36 | 0.040 |
| NM_031494 | *Zfp275* | 2.41 | 10.85 | 0.038 |
| NM_177370 | *Rhbdd3* | 2.40 | 14.28 | 0.026 |
| NM_146203 | *8030466O12Rik* | 2.40 | 12.49 | 0.021 |
| NM_172814 | *Lrp12* | 2.39 | 14.79 | 0.017 |
| NM_021487 | *Kcne1l* | 2.39 | 9.30 | 0.028 |
| NM_026799 | *Rnasen* | 2.39 | 9.58 | 0.032 |
| NM_023214 | *Slc30a7* | 2.39 | 11.10 | 0.037 |
| NM_009085 | *Rpo1-1* | 2.39 | 15.11 | 0.027 |
| NM_144807 | *Chpt1* | 2.38 | 8.26 | 0.028 |
| NM_026005 | *2610301B20Rik* | 2.38 | 10.93 | 0.016 |
| NM_024192 | *Cuedc2* | 2.36 | 11.21 | 0.039 |
| NM_010122 | *Eif2b4* | 2.36 | 13.82 | 0.022 |
| NM_027930 | *2610016C23Rik* | 2.36 | 9.85 | 0.030 |
| AK165760 | *Med25* | 2.36 | 11.33 | 0.044 |
| NM_001003815 | *Epb4.1l1* | 2.36 | 10.38 | 0.033 |
| NM_021504 | *Ngly1* | 2.36 | 9.17 | 0.037 |
| NM_152801 | *Arhgef6* | 2.36 | 9.91 | 0.021 |
| NM_018831 | *Dclre1a* | 2.35 | 9.61 | 0.027 |
| NM_001001792 | *Zfp239* | 2.35 | 10.13 | 0.022 |
| NM_007465 | *Birc2* | 2.35 | 9.72 | 0.050 |
| NM_138659 | *Prpf8* | 2.35 | 10.70 | 0.010 |
| NM_133676 | *Osgep* | 2.35 | 9.68 | 0.039 |
| NM_010210 | *Fhit* | 2.34 | 9.09 | 0.006 |
| NM_020022 | *Rfc2* | 2.33 | 12.73 | 0.018 |
| NM_030139 | *Zfp449* | 2.33 | 10.10 | 0.030 |
| AK048015 | *Air* | 2.33 | 9.64 | 0.039 |
| NM_025877 | *Slc25a23* | 2.33 | 9.66 | 0.041 |
| AK122311 | *Opa1* | 2.32 | 10.84 | 0.028 |
| NM_009048 | *Reps1* | 2.32 | 9.86 | 0.037 |
| NM_175118 | *0710001B24Rik* | 2.31 | 10.11 | 0.017 |
| NM_175399 | *Exosc4* | 2.31 | 10.83 | 0.031 |
| NM_007832 | *Dck* | 2.30 | 10.03 | 0.021 |
| NM_025343 | *0610042C05Rik* | 2.30 | 12.06 | 0.023 |
| XM_913601 | *Fbxl16* | 2.30 | 10.18 | 0.017 |
| NM_172546 | *Cnksr3* | 2.29 | 9.98 | 0.024 |
| NM_001033300 | *Gmps* | 2.29 | 9.37 | 0.047 |
| NM_025979 | *Mastl* | 2.29 | 9.32 | 0.031 |
| NM_011514 | *Suv39h1* | 2.29 | 10.46 | 0.039 |
| NM_133348 | *Acot7* | 2.29 | 16.21 | 0.022 |
| NM_011697 | *Vegfb* | 2.29 | 10.15 | 0.039 |
| NM_024243 | *Fuca1* | 2.28 | 13.71 | 0.015 |
| NM_016910 | *Ppm1d* | 2.28 | 9.89 | 0.025 |
| NM_028059 | *Zfp654* | 2.26 | 10.52 | 0.050 |
| NM_001042421 | *Kntc1* | 2.26 | 10.52 | 0.025 |
| NM_029851 | *Dync2h1* | 2.26 | 8.95 | 0.022 |
| BC066035 | *Zranb3* | 2.25 | 10.20 | 0.040 |
| NM_028099 | *Dusp11* | 2.25 | 11.30 | 0.033 |
| TC1647922 | *TC1462346* | 2.25 | 10.14 | 0.008 |
| NM_025326 | *0610011I04Rik* | 2.25 | 13.64 | 0.043 |
| NM_153798 | *Polr2b* | 2.25 | 12.78 | 0.014 |
| NM_172993 | *Zfp512* | 2.25 | 9.63 | 0.019 |
| NM_008976 | *Ptpn14* | 2.25 | 10.43 | 0.020 |
| NM_174852 | *Phf12* | 2.24 | 10.57 | 0.034 |
| ENSMUST00000100152 | *ENSMUST00000100152* | 2.24 | 16.97 | 0.034 |
| NM_080553 | *Itpr3* | 2.24 | 14.61 | 0.013 |
| NAP070973-1 | *NAP070973-1* | 2.23 | 15.40 | 0.037 |
| NM_173002 | *Zxdc* | 2.22 | 9.57 | 0.020 |
| NM_029102 | *Glt8d2* | 2.22 | 8.48 | 0.020 |
| NM_019752 | *Htra2* | 2.22 | 15.06 | 0.040 |
| NM_022982 | *Rtn4r* | 2.22 | 9.76 | 0.016 |
| NM_178601 | *Imp4* | 2.21 | 7.93 | 0.020 |
| NM_023709 | *Capn9* | 2.21 | 8.44 | 0.022 |
| NM_199007 | *Sgol2* | 2.21 | 10.29 | 0.018 |
| NM_145223 | *Alms1* | 2.21 | 8.59 | 0.019 |
| NM_010113 | *Egf* | 2.21 | 9.23 | 0.033 |
| NM_148925 | *Fyco1* | 2.21 | 9.67 | 0.024 |
| NM_145955 | *1110007A13Rik* | 2.21 | 10.24 | 0.034 |
| NM_133641 | *Rtkn* | 2.21 | 12.84 | 0.015 |
| NM_146066 | *Gspt1* | 2.20 | 13.22 | 0.034 |
| NM_011542 | *Tcea3* | 2.20 | 9.19 | 0.025 |
| NM_009739 | *Bckdk* | 2.20 | 13.87 | 0.010 |
| NM_172719 | *Gcn1l1* | 2.20 | 10.47 | 0.038 |
| AK140498 | *Sorbs2* | 2.19 | 10.78 | 0.023 |
| NM_025773 | *Ube2w* | 2.19 | 10.82 | 0.048 |
| AK031247 | *Xrn2* | 2.19 | 12.17 | 0.028 |
| ENSMUST00000064258 | *ENSMUST00000064258* | 2.19 | 16.75 | 0.040 |
| BU515288 | *Tmem106b* | 2.18 | 12.14 | 0.016 |
| L26316 | *Dhfr* | 2.18 | 10.19 | 0.038 |
| NM_026067 | *Thex1* | 2.18 | 9.27 | 0.017 |
| NM_010163 | *Ext2* | 2.18 | 15.62 | 0.047 |
| BB426169 | *BB426169* | 2.18 | 9.21 | 0.047 |
| NM_146033 | *Ankmy2* | 2.17 | 10.16 | 0.033 |
| NM_026653 | *Rpa1* | 2.17 | 13.23 | 0.032 |
| NM_021499 | *Wdr8* | 2.17 | 9.88 | 0.027 |
| AK033428 | *D7Wsu128e* | 2.17 | 10.39 | 0.044 |
| NM_145427 | *Atpaf2* | 2.17 | 11.25 | 0.020 |
| NM_025772 | *Dtnbp1* | 2.17 | 11.04 | 0.026 |
| NM_138305 | *Adcy3* | 2.16 | 7.45 | 0.009 |
| NM_144553 | *Dlg7* | 2.16 | 9.02 | 0.023 |
| NM_011494 | *Stk16* | 2.16 | 13.62 | 0.046 |
| NM_001048208 | *AK136576* | 2.16 | 11.77 | 0.030 |
| NM_019946 | *Mgst1* | 2.16 | 9.45 | 0.038 |
| NM_016672 | *Ddc* | 2.15 | 11.03 | 0.018 |
| NM_007891 | *E2f1* | 2.15 | 10.01 | 0.020 |
| NM_030561 | *BC004004* | 2.15 | 15.54 | 0.045 |
| NM_009193 | *Slbp* | 2.14 | 14.01 | 0.025 |
| TC1688953 | *NAP048817-1* | 2.14 | 12.38 | 0.048 |
| BC083072 | *Mib1* | 2.14 | 13.31 | 0.017 |
| NM_212468 | *Ssbp1* | 2.14 | 10.09 | 0.022 |
| NM_019693 | *Bat1a* | 2.13 | 15.31 | 0.023 |
| NM_008234 | *Hells* | 2.13 | 10.03 | 0.047 |
| NM_018813 | *Cpsf3* | 2.13 | 12.72 | 0.024 |
| NM_027829 | *9030607L17Rik* | 2.13 | 11.63 | 0.012 |
| NM_054089 | *Ncoa6ip* | 2.13 | 9.84 | 0.024 |
| NM_133694 | *Fbxl15* | 2.12 | 11.91 | 0.013 |
| NM_198424 | *Tmem142c* | 2.12 | 12.53 | 0.042 |
| NAP026388-1 | *NAP026388-1* | 2.12 | 10.34 | 0.022 |
| NM_021511 | *Rrs1* | 2.12 | 12.37 | 0.039 |
| NM_019791 | *Maged1* | 2.11 | 16.47 | 0.018 |
| NM_026465 | *2010316F05Rik* | 2.11 | 12.78 | 0.044 |
| NM_145959 | *D15Ertd621e* | 2.11 | 14.06 | 0.033 |
| AK083509 | *AK083509* | 2.10 | 6.78 | 0.049 |
| NM_028478 | *Rassf6* | 2.10 | 9.27 | 0.019 |
| NM_013872 | *Pmm1* | 2.09 | 11.60 | 0.017 |
| NM_130885 | *Oxr1* | 2.09 | 10.34 | 0.016 |
| AK080470 | *Srr* | 2.09 | 9.07 | 0.034 |
| NM_144546 | *Zfp119* | 2.08 | 9.11 | 0.017 |
| NM_172578 | *C79407* | 2.08 | 9.49 | 0.042 |
| NM_010829 | *Msh3* | 2.08 | 10.74 | 0.036 |
| AK088929 | *2810403D21Rik* | 2.08 | 10.42 | 0.043 |
| BC067070 | *4932415G12Rik* | 2.07 | 9.74 | 0.037 |
| AK053189 | *AK053189* | 2.07 | 9.36 | 0.013 |
| NM_181423 | *Supv3l1* | 2.07 | 10.85 | 0.011 |
| NM_010322 | *Gnpat* | 2.06 | 13.78 | 0.037 |
| BC075698 | *Mpp5* | 2.06 | 9.15 | 0.046 |
| NM_027464 | *5730469M10Rik* | 2.06 | 12.84 | 0.043 |
| NM_175156 | *2810407A14Rik* | 2.06 | 9.20 | 0.048 |
| AK011905 | *Ncam1* | 2.06 | 9.93 | 0.048 |
| NM_010330 | *Emb* | 2.05 | 8.82 | 0.017 |
| NM_009030 | *Rbbp4* | 2.05 | 8.45 | 0.037 |
| NM_016781 | *Prkag1* | 2.05 | 9.59 | 0.020 |
| NM_010549 | *Il11ra1* | 2.05 | 14.26 | 0.019 |
| NM_033370 | *Copb1* | 2.05 | 13.14 | 0.035 |
| NM_145920 | *Evc2* | 2.04 | 12.68 | 0.028 |
| NM_019717 | *Arl6ip2* | 2.04 | 10.77 | 0.027 |
| NM_030199 | *Zfp623* | 2.03 | 9.33 | 0.045 |
| NM_001033298 | *Gm114* | 2.03 | 13.05 | 0.008 |
| NM_153571 | *AW049829* | 2.02 | 11.01 | 0.046 |
| NM_144852 | *Slc7a4* | 2.02 | 9.92 | 0.028 |
| NM_028505 | *1700040I03Rik* | 2.02 | 12.45 | 0.019 |
| NM_030066 | *Armcx1* | 2.00 | 13.30 | 0.020 |
| XM_001006017 | *LOC382161* | 2.00 | 8.59 | 0.013 |
| NM_146135 | *Pias3* | 2.00 | 9.75 | 0.036 |
