# Supplementary material for: Transcriptional Profiling of Chondrodysplasia Growth Plate Cartilage Reveals Adaptive ER-Stress Networks That Allow Survival but Disrupt Hypertrophy
Source: PLoS One. 2011 Sep 15;6(9):e24600. doi: 10.1371/journal.pone.0024600 (PMC3174197; doi:10.1371/journal.pone.0024600)
Supplement: Table S9 — Wildtype hypertrophic zone gene expression signature GO analysis. (DOCX) [file pone.0024600.s013.docx]

| **Table S9 - Wildtype Hypertrophic Zone Gene Expression Signature GO Analysis** | | |  |  |  |
| --- | --- | --- | --- | --- | --- |
|  |  |  |  |  |  |
| **GO Cluster rank and functional annotation** | | | **DAVID v6.7 Enrichment Score** | **Corrected *p* value** | **Count** |
| 1 | GO:0043068 | positive regulation of programmed cell death | 3.64 | 6.11 E-02 | 19 |
| 2 | GO:0001944 | vasculature development | 2.71 | 5.34 E-02 | 18 |
| 3 | GO:0006796 | phosphate metabolic process | 2.37 | 7.95 E-02 | 38 |
| 4 | GO:0041012 | extracellular matrix | 2.20 | 1.46 E-01 | 17 |
| 5 | GO:0030036 | actin cytoskeleton organization | 1.96 | 2.38 E-01 | 12 |
| 6 | IPR004827 | basic-leucine zipper (bZIP) transcription factor | 1.79 | 1.39 E-01 | 9 |
| 7 | GO:0016791 | phosphatase activity | 1.74 | 3.30 E-01 | 13 |
| 8 | GO:0001501 | skeletal system development | 1.69 | 3.93 E-01 | 15 |
| 9 | GO:0001942 | hair follicle development | 1.47 | 6.18 E-01 | 5 |
| 10 | SP_PIR_KEYWORDS | amino acid transport | 1.46 | 1.91 E-01 | 5 |
| 11 | GO:0006915 | apoptosis | 1.42 | 6.95 E-01 | 19 |
| 12 | GO:0032990 | cell part morphogenesis | 1.40 | 5.49 E-01 | 12 |
| 13 | GO:0021915 | neural tube development | 1.36 | 2.83 E-01 | 8 |
| 14 | GO:0003779 | actin binding | 1.35 | 3.80 E-01 | 16 |
| 15 | IPR001464 | annexin | 1.35 | 7.24 E-01 | 3 |
| 16 | IPR015940 | Ubq-associated/translation elongation factor EF1B, N-terminal, eukaryote | 1.34 | 6.18 E-01 | 5 |
