# Supplementary material for: Transcriptional Profiling of Chondrodysplasia Growth Plate Cartilage Reveals Adaptive ER-Stress Networks That Allow Survival but Disrupt Hypertrophy
Source: PLoS One. 2011 Sep 15;6(9):e24600. doi: 10.1371/journal.pone.0024600 (PMC3174197; doi:10.1371/journal.pone.0024600)
Supplement: Table S10 — Wildtype proliferative zone gene expression signature GO analysis. (DOCX) [file pone.0024600.s014.docx]

| **Table S10 - Wildtype Proliferative Zone Gene Expression Signature GO Analysis** | | |  |  |  |
| --- | --- | --- | --- | --- | --- |
|  |  |  |  |  |  |
| **GO Cluster rank and functional annotation** | | | **DAVID v6.7 Enrichment Score** | **Corrected *p* value** | **Count** |
| 1 | GO:0007049 | cell cycle | 11.44 | 6.54 E-14 | 70 |
| 2 | GO:0006259 | DNA metabolic process | 9.96 | 1.01 E-11 | 52 |
| 3 | GO:0005694 | chromosome | 9.20 | 1.31 E-07 | 41 |
| 4 | GO:0043232 | intracellular non-membrane-bounded organelle | 5.94 | 1.17 E-05 | 109 |
| 5 | GO:0000775 | chromosome, centromeric region | 5.57 | 2.32 E-07 | 21 |
| 6 | GO:0006281 | DNA repair | 5.41 | 3.39 E-04 | 29 |
| 7 | GO:0000777 | condensed chromosome kinetochore | 4.61 | 1.59 E-02 | 9 |
| 8 | SP_PIR_KEYWORDS | cell cycle control | 4.14 | 1.90 E-05 | 9 |
| 9 | GO:0005730 | nucleolus | 4.07 | 2.15 E-04 | 30 |
| 10 | IPR014400 | cyclin, A/B/D/E | 3.85 | 1.37 E-02 | 6 |
| 11 | GO:0051726 | regulation of cell cycle | 3.47 | 1.11 E-02 | 21 |
| 12 | GO:0001882 | nucleoside binding | 3.21 | 1.90 E-02 | 80 |
| 13 | GO:0005657 | replication fork | 2.77 | 7.06 E-04 | 8 |
| 14 | GO:0006270 | DNA replication initiation | 2.62 | 3.00 E-02 | 5 |
| 15 | GO:0000307 | cyclin-dependent protein kinase holoenzyme complex | 2.51 | 6.60 E-02 | 4 |
| 16 | GO:0000075 | cell cycle checkpoint | 2.46 | 3.69 E-02 | 9 |
| 17 | GO:0001923 | nucleoside monophosphate metabolic process | 2.32 | 1.86 E-02 | 10 |
| 18 | GO:0006302 | double-stranded break repair | 2.26 | 3.83 E-04 | 11 |
| 19 | GO:0006350 | transcription | 2.22 | 1.21 E-01 | 87 |
| 20 | GO:0051276 | chromosome organization | 2.22 | 6.84 E-03 | 32 |
| 21 | GO:0000070 | mitotic sister chromatid segregation | 2.01 | 1.71 E-01 | 5 |
| 22 | IPR000910 | high mobility group, HMG1/HMG2 | 1.82 | 7.26 E-01 | 7 |
| 23 | mmu05222 | small cell lung cancer | 1.77 | 6.91 E-02 | 10 |
| 24 | SP_PIR_KEYWORDS | upl conjugation | 1.75 | 3.87 E-02 | 34 |
| 25 | GO:0051052 | regulation of DNA metabolic process | 1.72 | 1.92 E-01 | 8 |
| 26 | GO:0009262 | deoxyribonucleotide metabolic process | 1.69 | 4.40 E-02 | 6 |
| 27 | GO:0046395 | carboxylic acid catabolic process | 1.61 | 3.88 E-01 | 9 |
| 28 | GO:0043933 | macromolecular complex subunit organization | 1.55 | 1.44 E-01 | 25 |
| 29 | GO:0003678 | DNA helicase activity | 1.45 | 2.12 E-02 | 7 |
| 30 | GO:0004518 | nuclease activity | 1.39 | 1.84 E-01 | 13 |
| 31 | mmu00240 | pyrimidine metabolism | 1.37 | 2.15 E-02 | 14 |
| 32 | GO:0015630 | microtubule cytoskeleton | 1.34 | 3.04 E-02 | 30 |
| 33 | IPR003034 | DNA-binding SAP | 1.34 | 9.74 E-01 | 4 |
| 34 | IPR013817 | pre-ATP-grasp fold | 1.33 | 9.68 E-01 | 3 |
| 35 | GO:0034284 | response to monosaccharide stimulus | 1.33 | 6.83 E-01 | 4 |
| 36 | GO:0004540 | ribonuclease activity | 1.31 | 4.24 E-01 | 7 |
| 37 | GO:0042254 | ribosome biogenesis | 1.30 | 1.35 E-01 | 12 |
